# Genome-Wide Transcriptional Effects of the Anti-Cancer Agent Camptothecin 

**DOI:** 10.1371/journal.pone.0078190

**Published:** 2013-10-23

**Authors:** Artur Veloso, Benjamin Biewen, Michelle T. Paulsen, Nathan Berg, Leonardo Carmo de Andrade Lima, Jayendra Prasad, Karan Bedi, Brian Magnuson, Thomas E. Wilson, Mats Ljungman

**Affiliations:** 1 Department of Radiation Oncology, University of Michigan Comprehensive Cancer Center and Translational Oncology Program, University of Michigan, Ann Arbor, Michigan, United States of America; 2 Bioinformatics Program and Department of Computational Medicine and Bioinformatics, University of Michigan, Ann Arbor, Michigan, United States of America; 3 Gustavus Adolphus College, St. Peter, Minnesota, United States of America; 4 University of Sao Paulo, Sao Paulo, Brazil; 5 Department of Environmental Health Sciences, School of Public Health, University of Michigan, Ann Arbor, Michigan, United States of America; 6 Department of Pathology, University of Michigan, Ann Arbor, Michigan, United States of America; 7 Department of Human Genetics, University of Michigan, Ann Arbor, Michigan, United States of America; INSERM, France

## Abstract

The anti-cancer drug camptothecin inhibits replication and transcription by trapping DNA topoisomerase I (Top1) covalently to DNA in a “cleavable complex”. To examine the effects of camptothecin on RNA synthesis genome-wide we used Bru-Seq and show that camptothecin treatment primarily affected transcription elongation. We also observed that camptothecin increased RNA reads past transcription termination sites as well as at enhancer elements. Following removal of camptothecin, transcription spread as a wave from the 5’-end of genes with no recovery of transcription apparent from RNA polymerases stalled in the body of genes. As a result, camptothecin preferentially inhibited the expression of large genes such as proto-oncogenes, and anti-apoptotic genes while smaller ribosomal protein genes, pro-apoptotic genes and p53 target genes showed relative higher expression. Cockayne syndrome group B fibroblasts (CS-B), which are defective in transcription-coupled repair (TCR), showed an RNA synthesis recovery profile similar to normal fibroblasts suggesting that TCR is not involved in the repair of or RNA synthesis recovery from transcription-blocking Top1 lesions. These findings of the effects of camptothecin on transcription have important implications for its anti-cancer activities and may aid in the design of improved combinatorial treatments involving Top1 poisons.

## Introduction

DNA topoisomerase I (Top1) relaxes torsional tension that is generated in the DNA helix as a consequence of replication, transcription and chromatin remodeling [1,2]. The Top1-mediated reaction involves covalent binding to DNA, cleavage of one strand of the DNA helix followed by the passing of the other strand through the break and finally the resealing of the DNA strand break. The anti-cancer drug camptothecin specifically inhibits Top1 [3] by acting prior to the resealing step, effectively trapping Top1 covalently bound to the DNA in a “cleavable complex.” Camptothecin and other Top1 poisons are used for the treatment of ovarian, cervical, colon, pancreatic, lung, breast, prostate and brain cancers [4]. The anti-cancer activity of camptothecin is linked to replication-mediated toxicity [5]. The inhibitory effect of camptothecin on transcription has also been acknowledged to contribute to toxicity in non-dividing cells [6,7]. 

We previously showed that camptothecin-stabilized Top1-DNA complexes retard elongation but not initiation of transcription [8]. In fact, we observed an increased occupancy of RNA polymerase II in the promoter region of the *DHFR* gene correlating to an increased rate of initiation of transcription [8]. In response to transcription elongation blockage, Top1 is targeted for degradation in an ubiquitin-dependent manner [9] and subsequent residual DNA-bound amino acid residues may require the action of tyrosyl-DNA phosphodiesterase 1 (TDP1) for their removal in order for transcription elongation to resume [10]. Blockage of the transcription machinery by Top1 complexes trapped on DNA by camptothecin has been shown to lead to the induction of DNA double strand breaks [6] and the formation of DNA-RNA hybrid structures (R-loops) activating the stress kinase ATM [7,11]. Furthermore, this transcription stress results in activation of the p53 response pathway [12–15] and induction of 53BP1-mediated DNA damage processing [16]. Top2 and PARP1 play overlapping roles to Top1 in non-dividing cells suggesting that the combination of Top1,Top2 and PARP-targeting drugs may be effective in non-dividing tumor cells [15]. 

Following camptothecin reversal, the topoisomerase reaction is completed and transcription complexes are thought to resume elongation. Interestingly, we previously observed that the recovery of RNA synthesis from the *Dhfr* gene in CHO cells following camptothecin removal resumed as a wave in a 5’-3’ direction with no apparent recovery downstream in the gene [8]. This suggests that transcription complexes blocked by trapped Top1 complexes are unable to resume elongation following camptothecin reversal [8]. The Cockayne complementation group B protein (CSB) defective in transcription-coupled repair (TCR) has been suggested to be involved in the repair of covalently DNA-linked Top1 [17]. It was suggested that this was coupled to a slower recovery of total RNA synthesis in CS-B cells correlating to a hypersensitivity to camptothecin exposure [15,17,18]. However, other studies have found no defect in RNA synthesis recovery following CSB knockdown [16].

To analyze the direct effect of camptothecin on transcription genome-wide and explore transcription recovery following drug removal, we used the recently developed Bru-Seq method [19]. This technique is based on the metabolic labeling of RNA using bromouridine (Bru) followed by specific isolation of Bru-labeled nascent RNA, library preparation and deep sequencing [19]. Our results show that the Top1 inhibitor camptothecin causes a preferential inhibition of expression of large genes through blockage of transcription elongation in combination with a lack of recovery of synthesis from RNA polymerases blocked in the body of the genes. Furthermore, we found no defect in RNA synthesis recovery in CS-B cells following camptothecin reversal, suggesting that TCR may not be required for recovery following topo I inhibition reversal.

## Materials and Methods

### Cell lines, camptothecin treatment and Bru-Seq

hTERT immortalized diploid human foreskin fibroblasts (gift from Dr. Mary Davis, Department of Radiation Oncology, University of Michigan) and CS-B fibroblasts (GM00739, Coriell Cell Repository) were grown as monolayers in MEM supplied with 10% fetal bovine serum and antibiotics (Invitrogen). Cells were treated for 45 min with 20 µM camptothecin (Sigma) and labeled for 15 min with 2 mM bromouridine either during the last 15 min of camptothecin treatment or following washout. The Bru-Seq and BruChase-Seq procedures were performed as previously described [19]. In short, total RNA was isolated from the cell samples using TRIzol reagent (Invitrogen) followed by specific isolation of Bru-labeled RNA using anti-BrdU antibodies (BD Biosciences) conjugated to magnetic beads (Dynabeads, Goat anti-Mouse IgG, Invitrogen). The isolated RNA was then converted into a strand-specific DNA library using the Illumina TruSeq Kit (Illumina) as previously described [19]. 

### Illumina Hi-Seq sequencing and data analysis

Sequencing of the cDNA libraries was performed by the staff at the University of Michigan Sequencing Core using the Illumina HiSeq 2000 sequencer. Base calling was performed using Illumina Casava v1.8.2. and read mapping was performed using TopHat, accepting only reads that could be mapped uniquely to the genome. We calculated RPKM values from the Bru-Seq data and plotted the data using a custom-built browser as previously described [19]. 

### Data availability

The primary data used in the analyses has been deposited at NCBI’s Gene Expression Omnibus and is freely available. We have uploaded the original genome mapping BAM files and the derived synthesis lists as BED files. The accession number is GSE48678 and the complete link is http://www.ncbi.nlm.nih.gov/geo/query/acc.cgi?acc=GSE48678. 

## Results

### Camptothecin preferentially inhibits RNA synthesis of large genes

**Figure 1:**
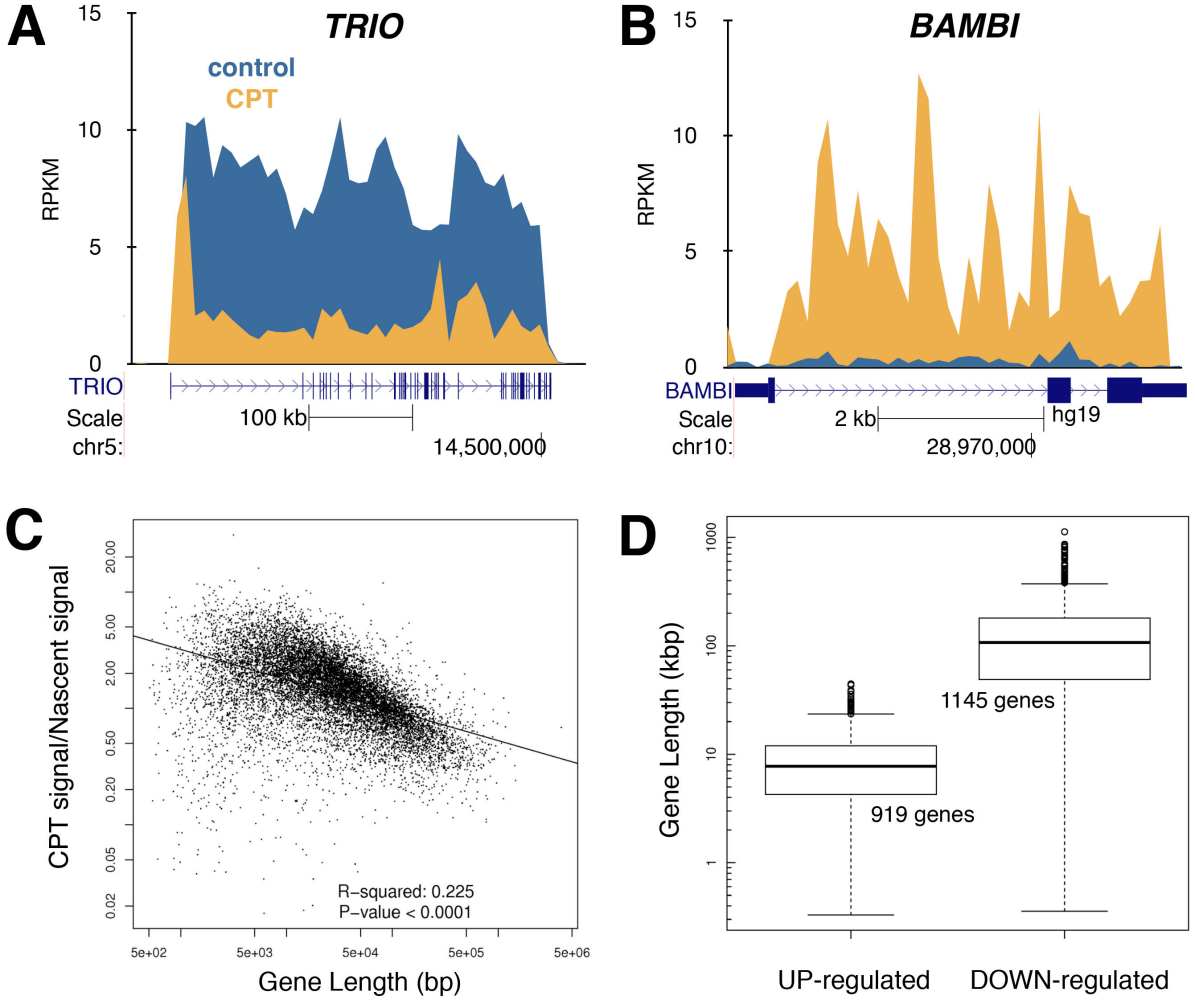
Gene size is a major contributing factor to the effects of camptothecin on RNA synthesis. Human fibroblasts were treated with 20 µM camptothecin for 45 min with 2 mM Bru added during the last 15 min of camptothecin treatment to label nascent RNA followed by Bru-Seq. (**A**), Long genes, such as *TRIO*, exhibit elongation defects, but not transcription initiation, after camptothecin treatment. (**B**), Short genes, such as *BAMBI*, show a relative increase of RNA synthesis following camptothecin treatment. (**C**), Effect of camptothecin on relative transcription as a function of gene size. Ratio of Bru-Seq signal of individual genes in camptothecin-treated over control cells as a function of gene size. Longer genes are inhibited preferentially over shorter genes. (**D**), The median length of genes induced >2-fold by camptothecin (919 genes) is 8,927 bp, whereas genes down-regulated >2-fold (1,145 genes) have a median length of 136,355 bp. The gene maps are from RefSeq Genes (UCSC genome browser).

We challenged human fibroblasts for 45 min with 20 µM camptothecin and labeled RNA with 2 mM Bru for the last 15 min in the presence of camptothecin. The sequencing reads from the nascent Bru-containing RNA mapped throughout genes covering both exons and introns and relative rates of transcription were determined for all genes by summing up the number of reads throughout the genes and dividing it by the length of the gene. The read density was expressed as “reads per thousand base pairs per million reads” (RPKM). Sample statistics can be found in Table S1. Camptothecin treatment inhibited transcription rates of 1142 genes by more than 2-fold while increasing transcription of 919 genes by more than 2-fold (Figures 1 A and B, Figure S1, S2 in File S1 and Table S2). Whether the genes found to have increased relative rates of transcription are truly being synthesized at an absolute higher rate is not determined since the data generated from Bru-Seq represents the distribution of reads rather than absolute expression values. Therefore, when synthesis is reduced in the body of large genes, sequencing reads must accumulate elsewhere (i.e. in small genes and at the beginning of large genes).

The data show an obvious negative correlation between read intensity and gene size following camptothecin treatment (Figure 1C). The median size of the genes with more than a 2-fold decreased relative transcription rates was 107,089 bp. (Figure 1D). The median genomic size of the 919 genes showing increased relative transcription rates following camptothecin treatment was 7,748 bp. These findings are consistent with a mechanism of action whereby camptothecin inhibits transcription elongation without inhibiting transcription initiation [8]. 

### Camptothecin affects transcription termination and expression of ncRNA and enhancer RNA (eRNA)

**Figure 2:**
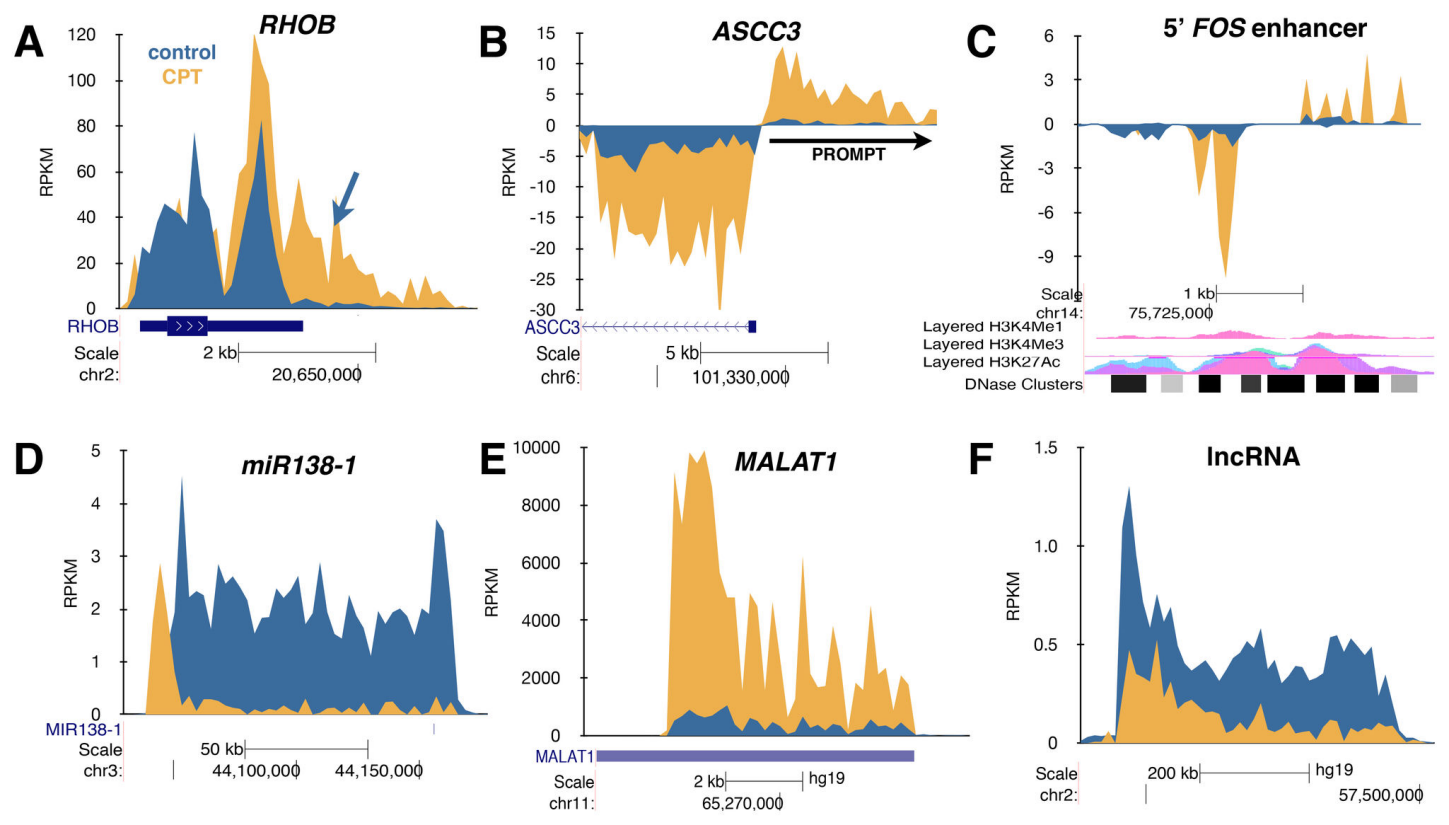
Effect of camptothecin on transcriptional readthrough and synthesis of PROMPTs and eRNA. As in Figure 1, human fibroblasts were treated with 20 µM camptothecin for 45 min with 2 mM Bru added during the last 15 min of camptothecin treatment to label nascent RNA followed by Bru-Seq. (**A**), Transcriptional readthrough of the termination site of the *RHOB* gene induced by camptothecin. (**B**), Enhanced initiation of the *ASCC3* gene and coincident upregulation of divergent upstream PROMPT RNA. (**C**), Enhanced expression of eRNA from the 5’-upstream enhancer of *FOS* by camptothecin. (**D**), Camptothecin inhibits the transcription of the primary transcript of miRNA138-1. (**E**), Camptothecin induces transcription of the ncRNA MALAT1. (F), Camptothecin inhibits the transcription of a very long unannotated ncRNA on chromosome 2. The gene maps are from RefSeq Genes (UCSC genome browser).

For many short genes where no inhibition of elongation was apparent following camptothecin treatment, transcription read-through past the annotated 3’ poly(A) site was prominent (Figure 2A, Figure S3 in File S1). This data supports a role for topoisomerase I in transcription termination in these genes [20]. Alternatively, the increased number of reads beyond the annotated termination sites may result from the induction of alternative poly(A) sites following camptothecin treatment or stabilization of the RNA past the 3’-cleavage site. Many genes in mammalian cells have been shown to generate divergent promoter upstream transcripts (PROMPTs) [19,21]. The expression of some PROMPTs was dramatically enhanced by camptothecin treatment (Figure 2B, Figure S4 A-C in File S1). Furthermore, many divergently transcribed genes showed coordinate initiation enhancement, suggesting that the negative superhelicity expected to accumulate in the wake of transcription in the absence of topoisomerase I activity may enhance transcription initiation (Figure S4 D-F). Finally, camptothecin treatment lead to the production of more enhancer RNA (eRNA) from many known and putative enhancer elements, such as the 5’ *FOS* enhancer (Figure 2C, Figure S5 in File S1). The functional consequence of the enhanced generation of eRNA following camptothecin treatment is not clear since the relative transcription rate of the *FOS* gene was not elevated despite the increase in eRNA generation. In addition to inhibiting the elongation of protein-coding genes, camptothecin inhibited transcription elongation of primary microRNA transcripts (Figure 2D) and enhanced or repressed long non-coding RNAs (lncRNAs) (Figure 2E&F).

### Transcription recovers as a wave from the 5’ end following camptothecin removal

**Figure 3:**
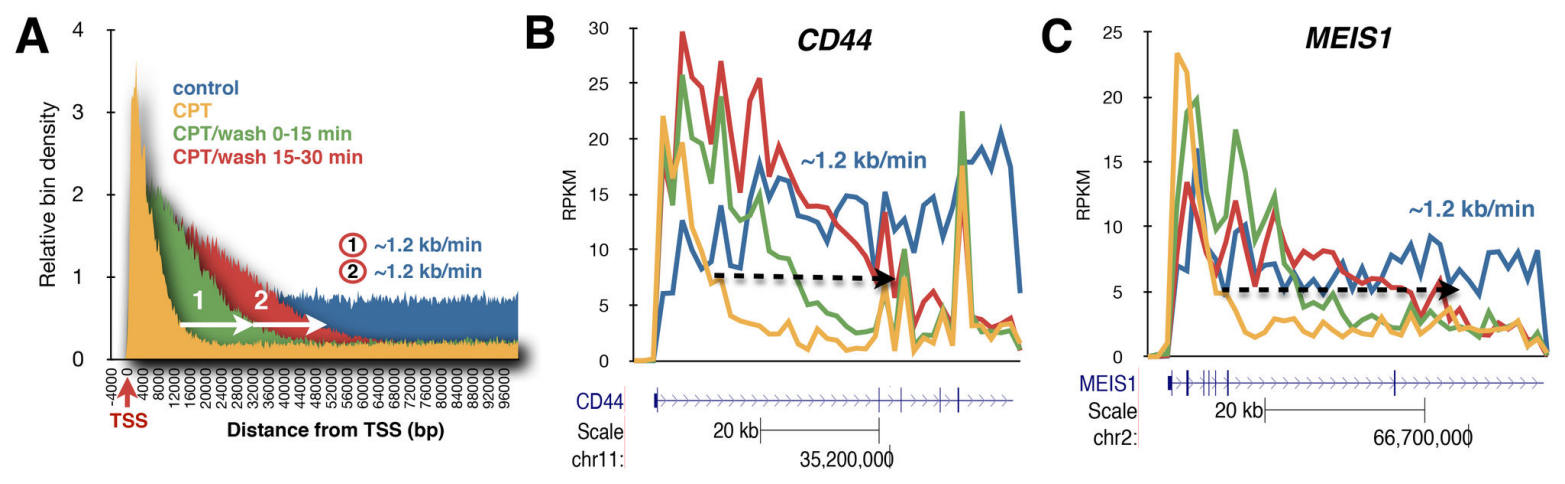
Effect of camptothecin reversal on RNA synthesis. (**A**), Aggregate view of RNA synthesis of genes larger than 100 kb in normal human fibroblasts with the genes aligned by transcriptional start sites (TSS). RNA synthesis recovers as a wave in a 5’-to-3’ direction following camptothecin removal with no apparent recovery of RNA polymerases stalled in the body of the genes. Elongation rates of the recovering transcription wave was estimated to be ~1.2 kb/min. (**B**), Wave of recovery of RNA synthesis can be seen advancing from the 5’-end of the *CD44* gene with no apparent recovery in the body of the gene. The front of the transcription wave extended some 35 kb during the first 30 min recovery resulting in an elongation rate of about 1.2 kb/min. (**C**) Similar elongation rates after camptothecin removal were found for the *MEISE1* gene. Color key: *Blue*, control (30 min Bru labeling); *Yellow*, Bru labeling during the last 15 min of a 45 min camptothecin treatment; *Green*, 45 min camptothecin treatment followed by a drug washout and 15 min of Bru labeling; *Red*, 45 min camptothecin treatment followed by a drug washout, 15 min incubation, and finally 15 min Bru labeling.

The trapping of topoisomerase I on DNA by camptothecin is thought to be a partially reversible event [2]. To explore whether the removal of camptothecin reverses its effects on transcription, we used Bru-Seq to examine the nascent RNA transcriptome in cells following drug washout. To get an aggregate picture of the effect of camptothecin on nascent RNA synthesis of multiple genes, we selected highly expressed genes (RPKM greater than 1) and longer than 100 kb and aligned them by their transcription start sites. We found that camptothecin induced a strong signal above control within the first 10 kb of genes followed by a severe drop in signal below control further downstream (Figure 3A). These results suggest that the inhibition and trapping of topoisomerase I by camptothecin does not inhibit initiation of transcription but strongly inhibits elongation. When camptothecin was washed out and cells were labeled with bromouridine for 15 min in the absence of the drug, recovery of transcription spread from the 5’-end into the gene while no recovery of signal was observed further downstream in the gene. Following washout of the drug and incubation for 15 minutes in drug-free media and then labeling nascent RNA for the following 15 minutes, the transcription wave moved further into the gene in the 3’ direction. Again, no recovery of signal was observed further downstream into the gene. Interestingly, the rate at which transcription wave spreading from the 5’-end and into the body of the genes was approximately 1.1-1.3 kb/min (Figure 3, Figure S6 in File S1). This is slower than the estimated elongation rate of around 2 kb/min in cells under normal conditions [22]. If elongating RNA polymerases collide with trapped topoisomerases, irreversible DNA damage may be induced that would require further processing [6]. It is possible that this reduced elongation rate observed following camptothecin treatment and washout is due to a requirement for a Top1/camptothecin-induced DNA damage repair to take place before elongation can resume.

### No apparent defect in the recovery of RNA synthesis in CS-B cells following camptothecin reversal

**Figure 4:**
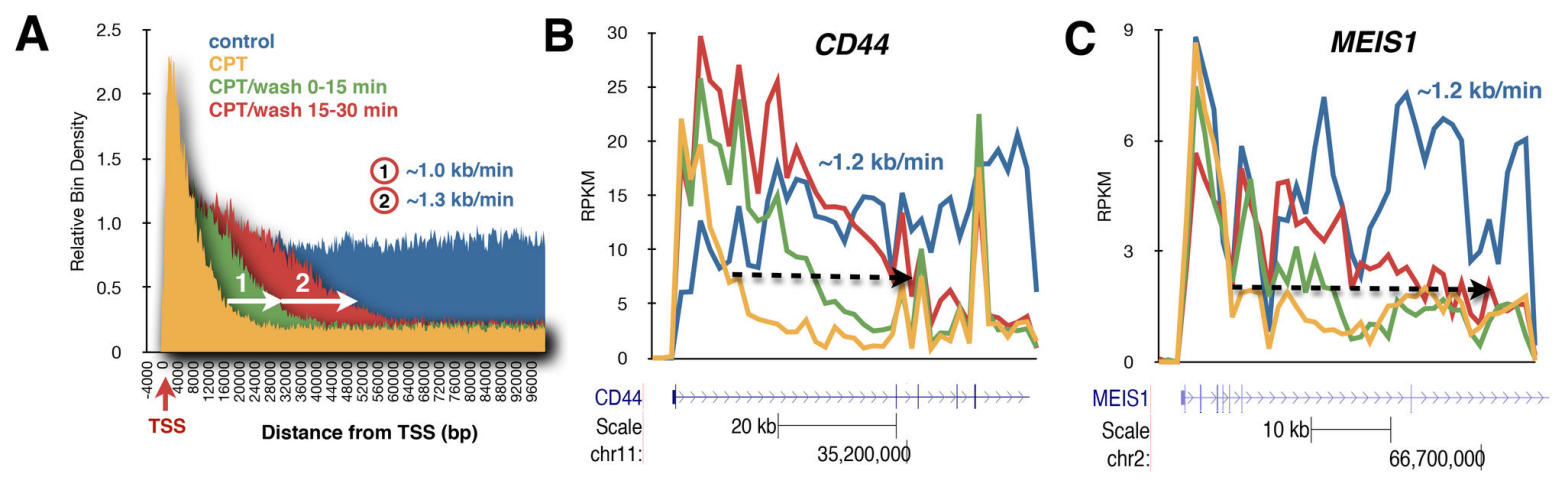
Effect of camptothecin reversal on RNA synthesis in Cockayne syndrome cells. (**A**), Aggregate view of RNA synthesis of genes larger than 100 kb in CS-B cells with the genes lined up by transcriptional start sites (TSS) as in Figure 3. Elongation rates of the recovering transcription wave was estimated to be ~1.0-1.3 kb/min. Individual genes in fibroblasts from a CS-B individual showing similar recovery rates as in fibroblasts from a normal individual for (**B**), *CD44* and (**C**) *MEIS1*. Color key as in Figure 3.

Cells derived from Cockayne syndrome patients are hypersensitive to camptothecin [18]. This hypersensitivity is linked to an enhanced induction of double strand breaks in S-phase as replication forks “collide” with trapped Top1 complexes [18]. Some studies have also shown that the recovery of RNA synthesis is slower in CS cells [15,17,18] while other studies have found no defect in RNA synthesis recovery [16]. Using Bru-Seq we tested whether the recovery of nascent RNA synthesis in CS-B fibroblasts following camptothecin treatment and reversal differed from the recovery in normal human fibroblasts. Analysis of the aggregate transcription signal of genes at least 100 kb or longer showed that CS-B cells recovered RNA synthesis in a wave from the 5’-end of these genes in a similar fashion as the normal fibroblasts (Figure 4A). This was also apparent for the individual genes (Figure 4B&C, Figure S7 in File S1). In addition, no recovery of transcription occurred within the bodies of the genes suggesting that blocked RNA polymerases are not able to resume elongation following camptothecin removal. The apparent normal recovery of RNA synthesis in these CS-B cells was in sharp contrast to the defective recovery of nascent RNA synthesis in these cells following UV-irradiation (unpublished data).

### Camptothecin affected cancer-relevant gene expression

**Figure 5:**
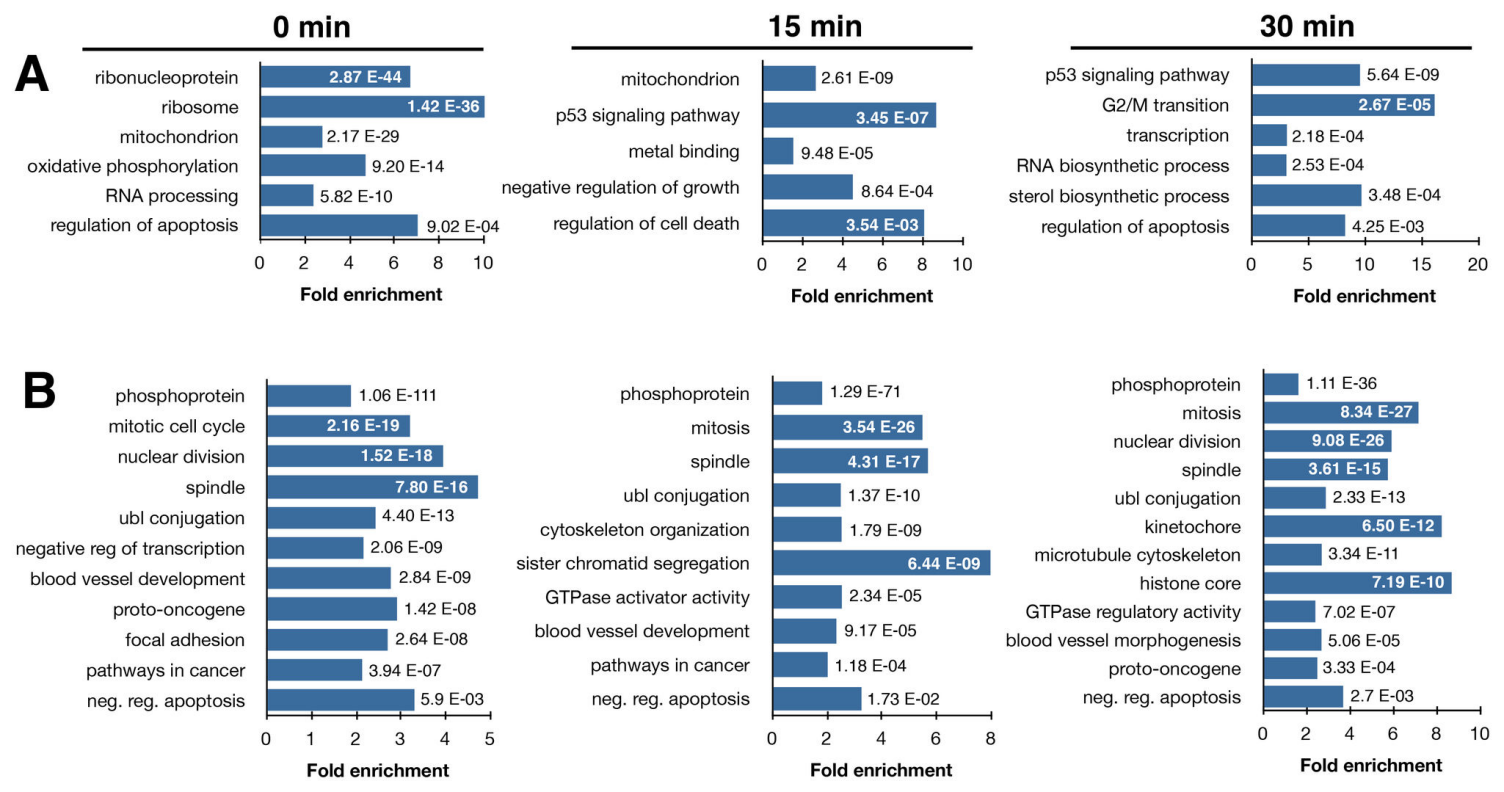
Pathway enrichment for genes following camptothecin treatment and reversal. (**A**) Pathways represented by genes up-regulated at least 2-fold, and (**B**) down-regulated at least 2-fold following camptothecin treatment and recovery. Human fibroblasts were treated with 20 µM camptothecin for 45 minutes and incubated for the last 15 min with 2 mM Bru (“0 min”), incubated for 15 min with Bru following the removal of camptothecin (“15 min”) or incubated for 15 min with Bru following a 45 min treatment, a wash and a 15 min recovery (“30 min”). Enrichment analysis was performed using DAVID (david.abcc.ncifcrf.gov) and the numbers shown represents the p-values for enrichment.

**Figure 6:**
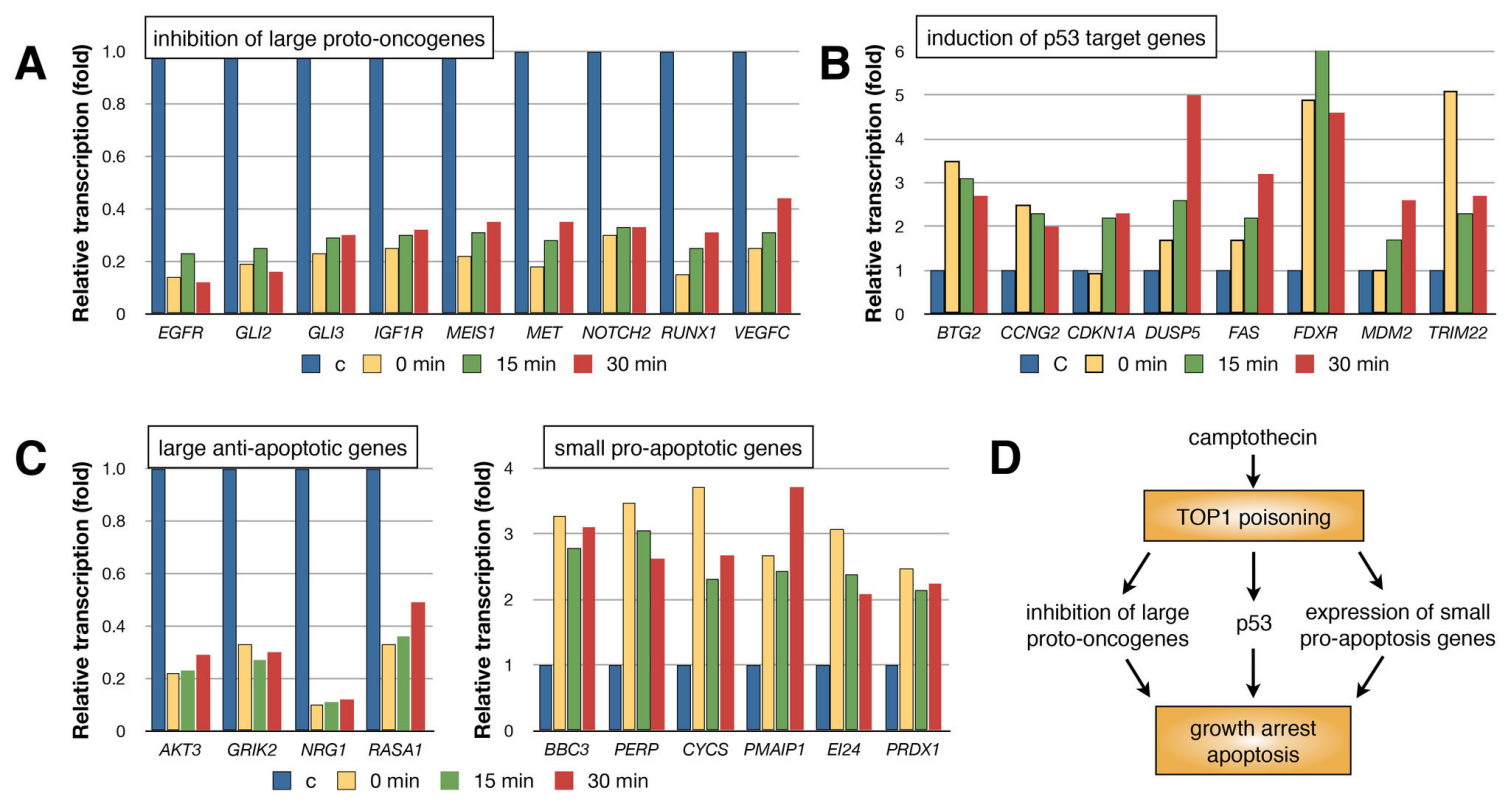
Camptothecin preferentially inhibits large genes such as proto-oncogenes and anti-apoptotic genes, enhances the relative expression of small pro-apoptotic genes and activates the p53 response. (**A**), Examples of large proto-oncogenes inhibited by camptothecin and showing no recovery (or slow recovery) following drug removal. (**B**), Examples of p53 target genes induced following camptothecin treatment. (**C**), Examples of large anti-apoptotic genes showing reduced relative transcription (left) and examples of small pro-apoptotic genes showing enhanced relative transcription following camptothecin treatment (right). (**D**), Model of mechanisms by which camptothecin may induce cell death or inhibit cell growth. Camptothecin triggers a p53 transcriptional response and selectively inhibits large proto-oncogenes and survival genes. The data is color coded where blue represents control (C), yellow represents 15 min Bru-labeling at the end of a 45 min camptothecin treatment with no recovery (“0 min”), green represents drug washout and 15 min Bru-labeling immediately after washout (“15 min”) and finally red represents labeling 15-30 minutes following washout (“30 min”).

Performing DAVID gene enrichment analysis we found that camptothecin-induced genes coding for elements of the ribosome, mitochondrion and the p53 and apoptosis signaling pathways were highly represented (Figure 5, Tables S2-4). The set of genes found to be inhibited shortly after camptothecin treatment was enriched for phosphoproteins, proto-oncogenes, and genes involved in the mitotic cell cycle, ubiquitin conjugation and anti-apoptosis. Some representative large proto-oncogenes inhibited by camptothecin are shown in Figure 6A. It has been shown that blockage of transcription elongation by camptothecin triggers a stress response leading to the rapid accumulation of p53 accompanied by phosphorylation of the Ser15 site and acetylation of the Lys382 site [12]. In support of camptothecin inducing a p53 response in human fibroblasts, we found that camptothecin induced genes in the p53 signaling pathway, including *CDKN1A* (p21), *MDM2, BTG2* and *FAS* (Figure 6B). Some of these genes were induced already during the camptothecin treatment while some genes, like CDKN1A and MDM2, showed induced expression only following reversal of drug treatment. Camptothecin reduced the relative transcription rates of large anti-apoptotic genes and enhanced expression of a set of smaller sized pro-apoptotic genes (Figure 6C). Pro-apoptotic genes are generally more compact compared to anti-apoptotic genes [23], thus agents preferentially reducing expression of large genes by blocking transcription elongation are expected to shift the balance of gene expression in favor of apoptosis. Similar patterns of increased and decreased relative gene expression following camptothecin treatment and reversal were found for CS-B cells (Figure S8 in File S1). There were, however, some differences between normal human fibroblasts and the CS-B cells were observed such as a lack of reduced *GLI2* expression and no induction of the p53-regulated genes *DUSP5*, *FAS*, *MDM2* and *TRIM22* in CS-B cells*.*

## Discussion

Camptothecin and its derivatives are FDA approved anti-cancer drugs used to treat a variety of tumors [4]. They act by trapping topoisomerase I complexes on DNA rather than inhibiting enzymatic function, since RNAi knockdown of Top1 does not reduce cell survival to the same degree as camptothecin treatment [4,24]. In this study, we used Bru-Seq to explore the acute effects of camptothecin on various aspects of transcription and found that camptothecin (i) inhibited elongation of transcription, (ii) stimulated transcriptional read-through past the 3’-end of small genes, (iii) enhanced expression of eRNA from certain enhancer elements (iv) induced the p53 response and (v) shifted the balance of expression of apoptosis-regulatory genes in favor of apoptosis. Importantly, transcription recovered with a reduced elongation rate as a wave from the 5’-end of the gene with no apparent recovery of synthesis from RNA polymerases blocked in the body of the genes. We found no evidence that the recovery of RNA synthesis was different in CS-B fibroblasts which is in sharp contrast to the recovery of RNA synthesis in these cells after UV light (unpublished data). Thus, the mechanisms responsible for the recovery of RNA synthesis following camptothecin removal are fundamentally different from those required following UV-irradiation suggesting that transcription-coupled repair has no major role in the restart of transcription following camptothecin removal. It is therefore conceivable that the observed hypersensitivity of CS-B cells to camptothecin is related to some role of the CSB protein during recovery of replication rather that in the recovery of transcription [18].

The inability of cells to restart transcription from within the body of genes suggests that blocked RNA polymerases are discarded rather than recycled. This will preferentially set back the expression of large genes even following a limited exposure of cells to camptothecin. Interestingly, many proto-oncogenes and anti-apoptotic genes belong to the class of genes preferentially inhibited by camptothecin. The model that emerges is that poisoning of Top1 by camptothecin results in the inhibition of large proto-oncogenes, enhanced expression of small pro-apoptotic genes and activation of the p53 pathway (Figure 6D). Knowledge of the size of the oncogenes that drive carcinogenesis and are important for survival of cancer cells in a given tumor may be used to select patients who would specifically benefit from camptothecin treatment and to rationally combine camptothecin with other treatment modalities. For example, the expression of the large BRCA1-associated RING domain protein 1 gene (BARD1) was reduced by camptothecin. Such suppression would be expected to suppress homologous recombination and thus should lead to increased susceptibility to PARP inhibitors or radiation therapy. Indeed, it has been shown that combining camptothecin with PARP inhibitors or radiotherapy improves tumor control [4,25–27]. 

## Supporting Information

File S1 Supporting Figures.
Figure S1. Large genes inhibited by camptothecin. (A), *SULF1*, (B), *NAV2*, (C), *SH3BP4*, (D), *KCNN2*, (E), *PLCB4* and (F), *TLE4* genes are showing inhibition of transcription elongation following a 45 min treatment of 20 µM camptothecin. Human fibroblast cells were incubated with 2 mM Bru during the last 15 min of camptothecin treatment to label nascent RNA followed by Bru-Seq. The gene maps are from RefSeq Genes (UCSC genome browser). Figure S2. Examples of small genes showing relative higher transcription reads following camptothecin treatment. (A), *RGS2*, (B), *HSPB3*, (C), *FUS*, (D), *TSPYL2*, (E), *CHCHD7* and (F), *TP53* represent genes that are upregulated following a 45 min treatment with 20 µM camptothecin. Human fibroblast cells were incubated with 2 mM Bru during the last 15 min of camptothecin treatment to label nascent RNA followed by Bru-Seq. The gene maps are from RefSeq Genes (UCSC genome browser). Figure S3. Increased transcription readthrough past the 3’-end of short genes. (A), *FZD7*, (B), *MYC*, (C), *CYR61*, (D), *DKK1*, (E), *SSTR1* and (F), *IER5*. Human fibroblast cells were treated with 20 µM camptothecin for 45 min and incubated with 2 mM Bru during the last 15 min of camptothecin treatment to label nascent RNA followed by Bru-Seq. The gene maps are from RefSeq Genes (UCSC genome browser). Figure S4. Effect of camptothecin on divergent upstream transcription. Camptothecin induces divergent upstream promoter transcription at the (A), *CEP78*, (B), *FER* and (C), *RAB33B* genes and increases divergent gene transcription for the (D),*PLAG1* and *CHCHD7*, (E), *CCDC58* and *FAM162A* and (F), *IMMP1L* and *ELP4* genes. Human fibroblast cells were treated with 20 µM camptothecin for 45 min and incubated with 2 mM Bru during the last 15 min of camptothecin treatment to label nascent RNA followed by Bru-Seq. The gene maps are from RefSeq Genes (UCSC genome browser). Figure S5. Effect of camptothecin on putative enhancers defined as regions with high H3K4m1 and H3K27ac while low H3K4m3 histone modifications. Human fibroblast cells were treated with 20 µM camptothecin for 45 min and incubated with 2 mM Bru during the last 15 min of camptothecin treatment to label nascent RNA followed by Bru-Seq. The gene maps, histone mark and DNase hypersensitivity tracks are from ENCODE and RefSeq Genes (UCSC genome browser). Figure S6. Effect of camptothecin reversal on RNA synthesis in normal human fibroblasts. Recovery of RNA synthesis is observed as a wave in a 5’ to 3’ direction following camptothecin removal with no apparent recovery of RNA polymerases stalled in the body of the genes for the (A) *TOP1*, (B) *SMAD3* and (C) *TLE4* genes. Color key: *Blue*, transcription reads in control cells; *Yellow*, transcription reads from cells labeled with Bru during the last 15 min of a 45 min treatment with camptothecin; *Green*, transcription reads from cells labeled for 15 min with Bru following a wash-out of camptothecin after a 45 min treatment; *Red*, transcription reads from cells labeled with Bru 15 min after drug washout following a 45 min treatment. Figure S7. Effect of camptothecin reversal on RNA synthesis in CS-B fibroblasts. Recovery of RNA synthesis is observed as a wave in a 5’ to 3’ direction following camptothecin removal with no apparent recovery of RNA polymerases stalled in the body of the genes for the (A) *TOP1*, (B) *SMAD3* and (C) *TLE4* genes. Color key: *Blue*, transcription reads in control cells; *Yellow*, transcription reads from cells labeled with Bru during the last 15 min of a 45 min treatment with camptothecin; *Green*, transcription reads from cells labeled for 15 min with Bru following a wash-out of camptothecin after a 45 min treatment; *Red*, transcription reads from cells labeled with Bru 15 min after drug washout following a 45 min treatment. Figure S8. Effect of camptothecin on the expression of genes shown in Figure 7 in CS-B cells. (A), large proto-oncogenes inhibited by camptothecin and showing no/slow recovery following drug removal. The *GLI2* gene was unaffected by camptothecin treatment in CS-B cells. (B), p53 target genes where only some were induced in CS-B cells following camptothecin treatment. (C), examples of large anti-apoptotic genes showing reduced relative transcription (leftt) and examples of small pro-apoptotic genes showing enhanced relative transcription in CS-B cells following camptothecin treatment (right). The data is color coded where blue represents control (C), yellow represents 15 min Bru-labeling at the end of a 45 min camptothecin treatment with no recovery (0 min), green represents drug washout and 15 min Bru-labeling immediately after washout (15 min) and finally red represents labeling 15-30 minutes following washout (30 min).
(PDF)

Table S1 **Sample statistics.**
(PDF)

Table S2 **Up and down regulation of genes following CPT treatment.**
(PDF)

Table S3 **Up and down regulation of genes following CPT treatment and 15 min. recovery.**
(PDF)

Table S4 **Up and down regulation of genes following CPT treatment and 30 min recovery.**
(PDF)
