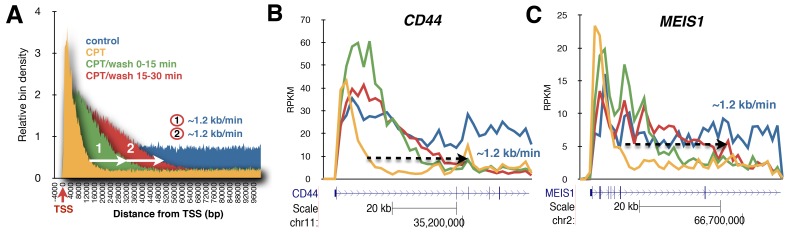# Correction: Genome-Wide Transcriptional Effects of the Anti-Cancer Agent Camptothecin 

**DOI:** 10.1371/annotation/d6d476e2-7c05-43df-bc34-edbda2e15a58

**Published:** 2013-12-16

**Authors:** Artur Veloso, Benjamin Biewen, Michelle T. Paulsen, Nathan Berg, Leonardo Carmo de Andrade Lima, Jayendra Prasad, Karan Bedi, Brian Magnuson, Thomas E. Wilson, Mats Ljungman

Figure 3B and Figure 4B were accidental duplications of the same data. Please see the corrected Figure 3 where panel B has been revised with the correct data: 

**Figure:**