# Supplementary material for: Genome-Wide Transcriptional Effects of the Anti-Cancer Agent Camptothecin
Source: PLoS One. 2013 Oct 23;8(10):e78190. doi: 10.1371/journal.pone.0078190 (PMC3806802; doi:10.1371/journal.pone.0078190)
Supplement: File S1 — Supporting Figures. Figure S1. Large genes inhibited by camptothecin. (A), SULF1, (B), NAV2, (C), SH3BP4, (D), KCNN2, (E), PLCB4 and (F), TLE4 genes are showing inhibition of transcription elongation following a 45 min treatment of 20 µM camptothecin. Human fibroblast cells were incubated with 2 mM Bru during the last 15 min of camptothecin treatment to label nascent RNA followed by Bru-Seq. The gene maps are from RefSeq Genes (UCSC genome browser). Figure S2. Examples of small genes showing relative higher transcription reads following camptothecin treatment. (A), RGS2, (B), HSPB3, (C), FUS, (D), TSPYL2, (E), CHCHD7 and (F), TP53 represent genes that are upregulated following a 45 min treatment with 20 µM camptothecin. Human fibroblast cells were incubated with 2 mM Bru during the last 15 min of camptothecin treatment to label nascent RNA followed by Bru-Seq. The gene maps are from RefSeq Genes (UCSC genome browser). Figure S3. Increased transcription readthrough past the 3’-end of short genes. (A), FZD7, (B), MYC, (C), CYR61, (D), DKK1, (E), SSTR1 and (F), IER5. Human fibroblast cells were treated with 20 µM camptothecin for 45 min and incubated with 2 mM Bru during the last 15 min of camptothecin treatment to label nascent RNA followed by Bru-Seq. The gene maps are from RefSeq Genes (UCSC genome browser). Figure S4. Effect of camptothecin on divergent upstream transcription. Camptothecin induces divergent upstream promoter transcription at the (A), CEP78, (B), FER and (C), RAB33B genes and increases divergent gene transcription for the (D),PLAG1 and CHCHD7, (E), CCDC58 and FAM162A and (F), IMMP1L and ELP4 genes. Human fibroblast cells were treated with 20 µM camptothecin for 45 min and incubated with 2 mM Bru during the last 15 min of camptothecin treatment to label nascent RNA followed by Bru-Seq. The gene maps are from RefSeq Genes (UCSC genome browser). Figure S5. Effect of camptothecin on putative enhancers defined as regions with high H3K4m1 and H3K27ac whil [file pone.0078190.s001.pdf]

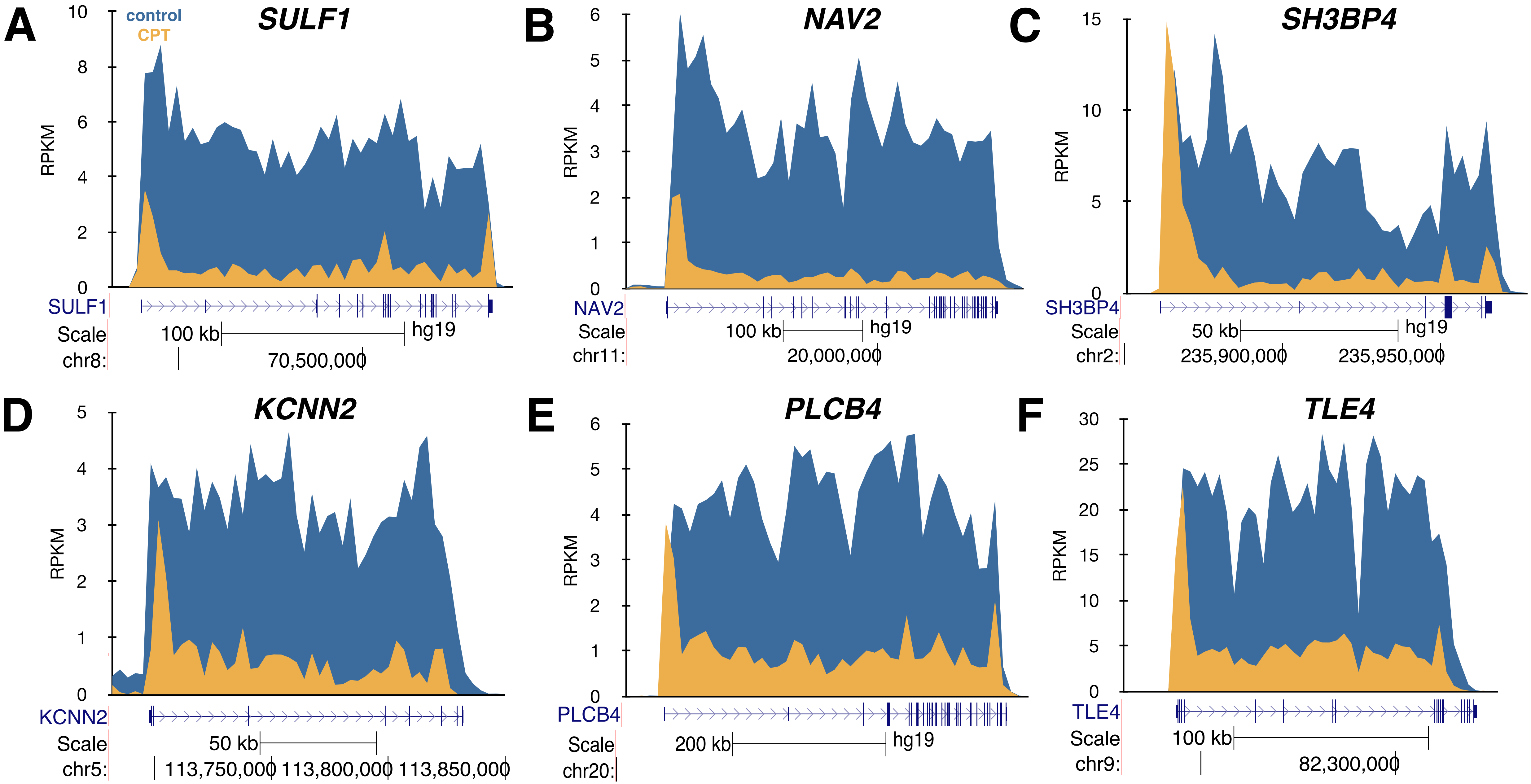

Figure S1. Large genes inhibited by camptothecin. (**A**), *SULF1*, (**B**), *NAV2*, (**C**), *SH3BP4*, (**D**), *KCNN2*, (**E**), *PLCB4* and (**F**), *TLE4* genes are showing inhibition of transcription elongation following a 45 min treatment of 20 μM camptothecin. Human fibroblast cells were incubated with 2 mM Bru during the last 15 min of camptothecin treatment to label nascent RNA followed by Bru-Seq. The gene maps are from RefSeq Genes (UCSC genome browser).

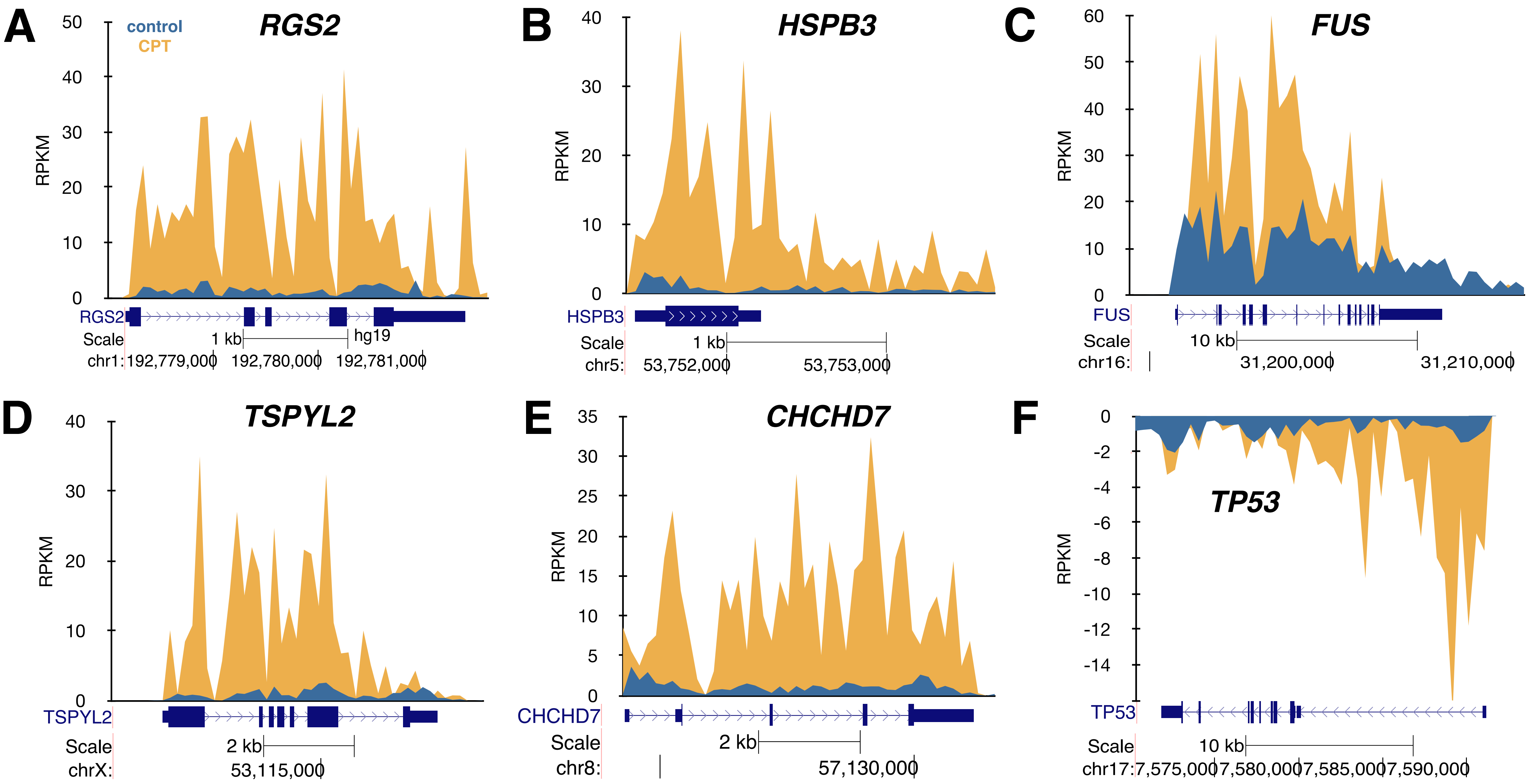

Figure S2. Examples of small genes showing relative higher transcription reads following camptothecin treatment. (**A**), *RGS2*, (**B**), *HSPB3*, (**C**), *FUS*, (**D**), *TSPYL2*, (**E**), *CHCHD7* and (**F**), *TP53* represent genes that are upregulated following a 45 min treatment with 20 μM camptothecin. Human fibroblast cells were incubated with 2 mM Bru during the last 15 min of camptothecin treatment to label nascent RNA followed by Bru-Seq. The gene maps are from RefSeq Genes (UCSC genome browser).

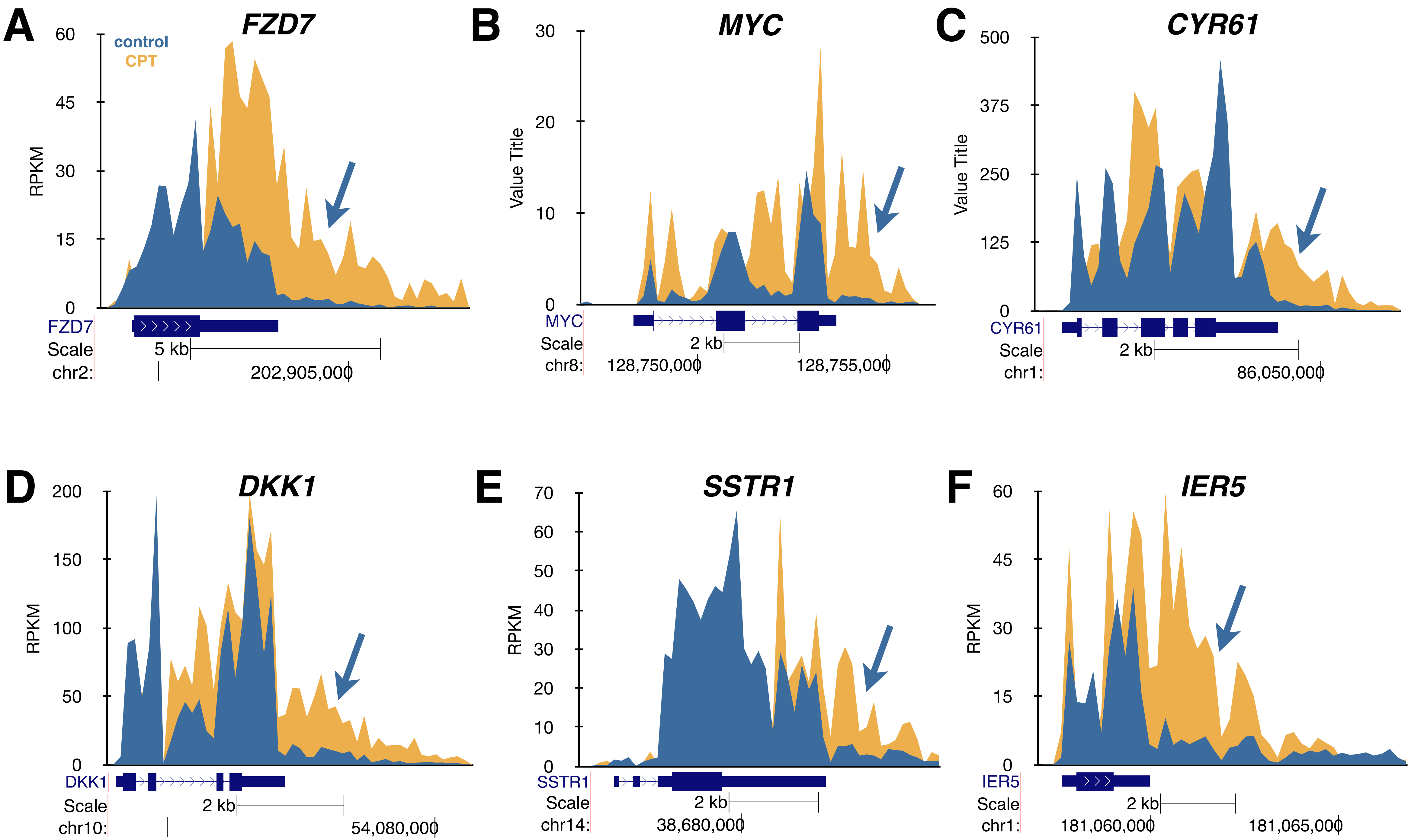

Figure S3. Increased transcription readthrough past the 3'-end of short genes. (**A**), *FZD7*, (**B**), *MYC*, (**C**), *CYR61*, (**D**), *DKK1*, (**E**), *SSTR1* and (**F**), *IER5*. Human fibroblast cells were treated with 20 μM camptothecin for 45 min and incubated with 2 mM Bru during the last 15 min of camptothecin treatment to label nascent RNA followed by Bru-Seq. The gene maps are from RefSeq Genes (UCSC genome browser).

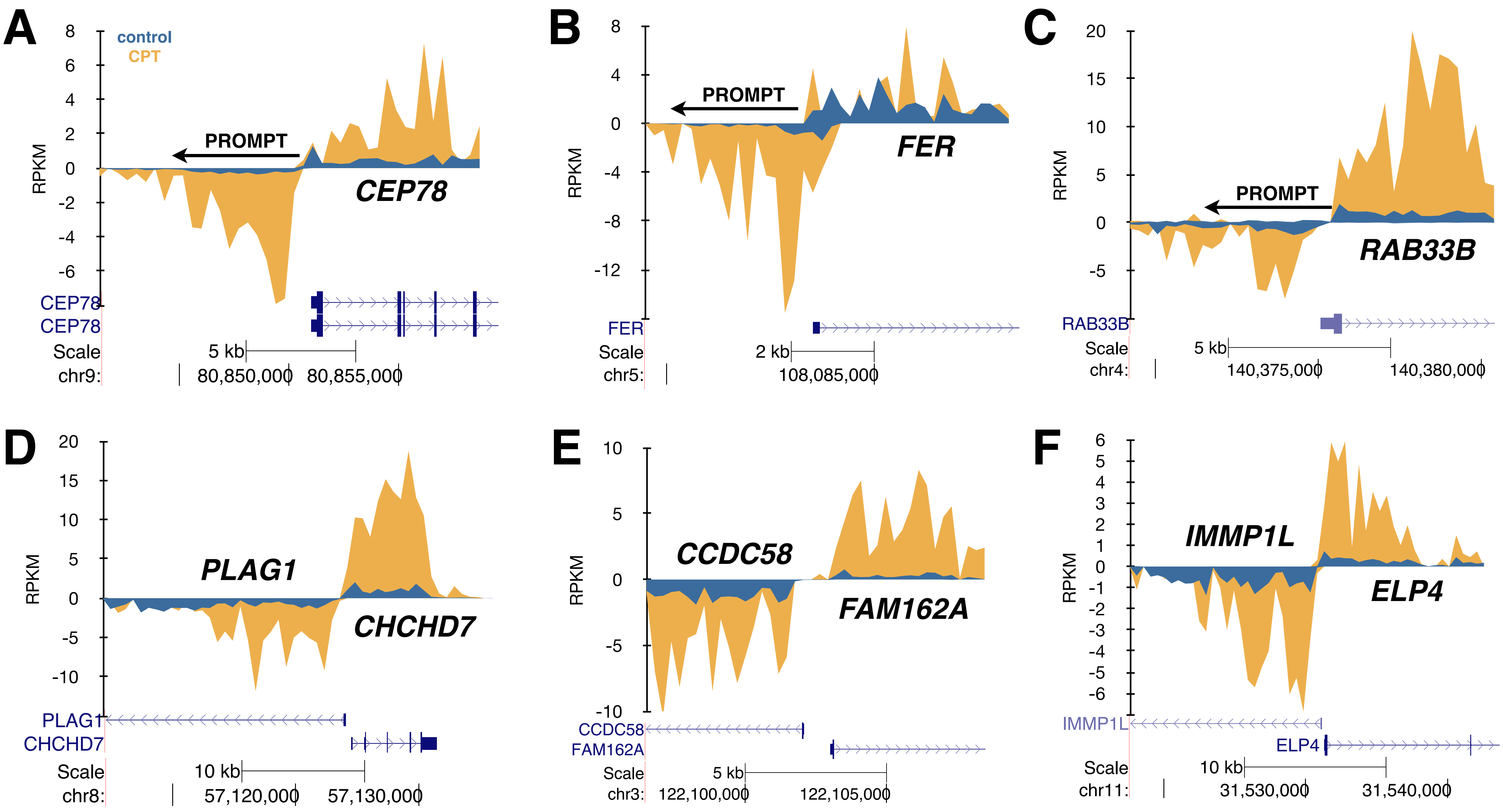

Figure S4. Effect of camptothecin on divergent upstream transcription. Camptothecin induces divergent upstream promoter transcription at the (**A**), *CEP78*, (**B**), *FER* and (**C**), *RAB33B* genes and increases divergent gene transcription for the (**D**),*PLAG1* and *CHCHD7*, (**E**), *CCDC58* and *FAM162A* and (**F**), *IMMP1L* and *ELP4* genes. Human fibroblast cells were treated with 20 μM camptothecin for 45 min and incubated with 2 mM Bru during the last 15 min of camptothecin treatment to label nascent RNA followed by Bru-Seq. The gene maps are from RefSeq Genes (UCSC genome browser).

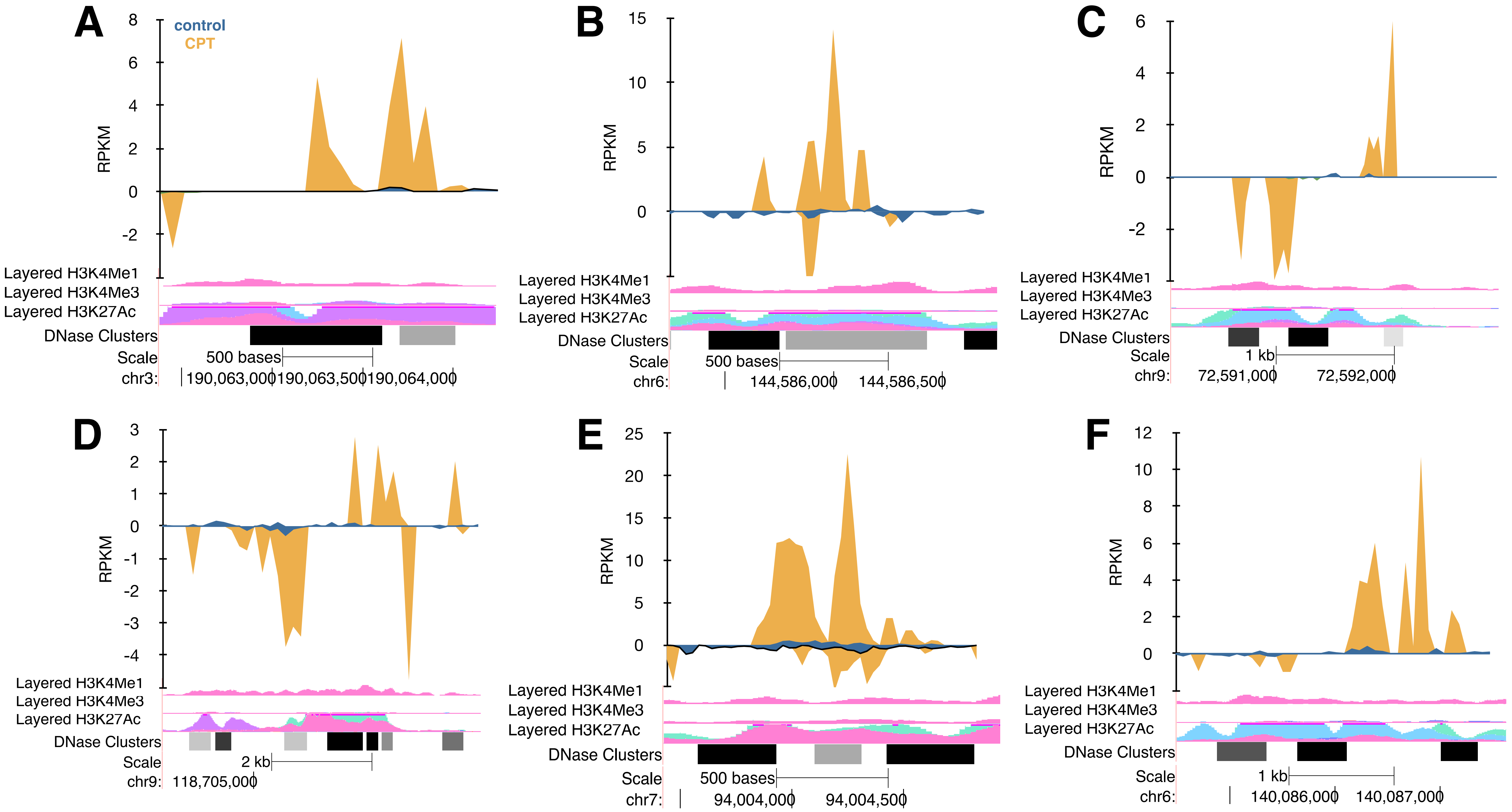

Figure S5. Effect of camptothecin on putative enhancers defined as regions with high H3K4m1 and H3K27ac while low H3K4m3 histone modifications. Human fibroblast cells were treated with 20 µM camptothecin for 45 min and incubated with 2 mM Bru during the last 15 min of camptothecin treatment to label nascent RNA followed by Bru-Seq. The gene maps, histone mark and DNase hypersensitivity tracks are from ENCODE and RefSeq Genes (UCSC genome browser).

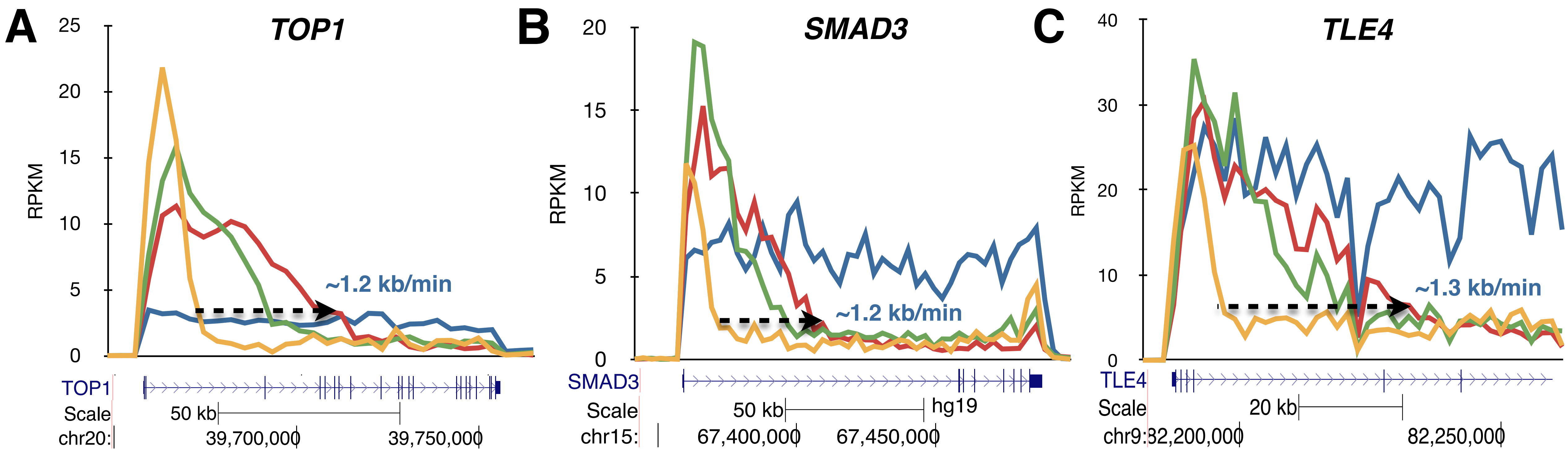

Figure S6. Effect of camptothecin reversal on RNA synthesis in normal human fibroblasts. Recovery of RNA synthesis is observed as a wave in a 5' to 3' direction following camptothecin removal with no apparent recovery of RNA polymerases stalled in the body of the genes for the (**A**) *TOP1*, (**B**) *SMAD3* and (**C**) *TLE4* genes. Color key: *Blue*, transcription reads in control cells; *Yellow*, transcription reads from cells labeled with Bru during the last 15 min of a 45 min treatment with camptothecin; *Green*, transcription reads from cells labeled for 15 min with Bru following a wash-out of camptothecin after a 45 min treatment; *Red*, transcription reads from cells labeled with Bru 15 min after drug washout following a 45 min treatment.

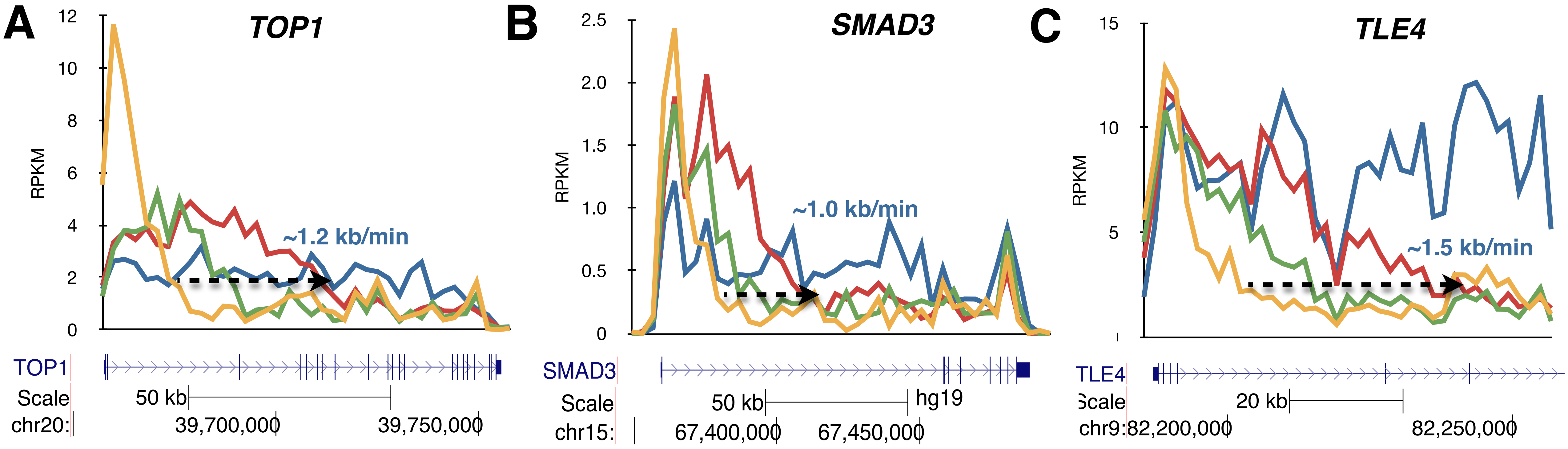

Figure S7. Effect of camptothecin reversal on RNA synthesis in CS-B fibroblasts. Recovery of RNA synthesis is observed as a wave in a 5' to 3' direction following camptothecin removal with no apparent recovery of RNA polymerases stalled in the body of the genes for the (**A**) *TOP1*, (**B**) *SMAD3* and (**C**) *TLE4* genes. Color key: *Blue*, transcription reads in control cells; *Yellow*, transcription reads from cells labeled with Bru during the last 15 min of a 45 min treatment with camptothecin; *Green*, transcription reads from cells labeled for 15 min with Bru following a wash-out of camptothecin after a 45 min treatment; *Red*, transcription reads from cells labeled with Bru 15 min after drug washout following a 45 min treatment.

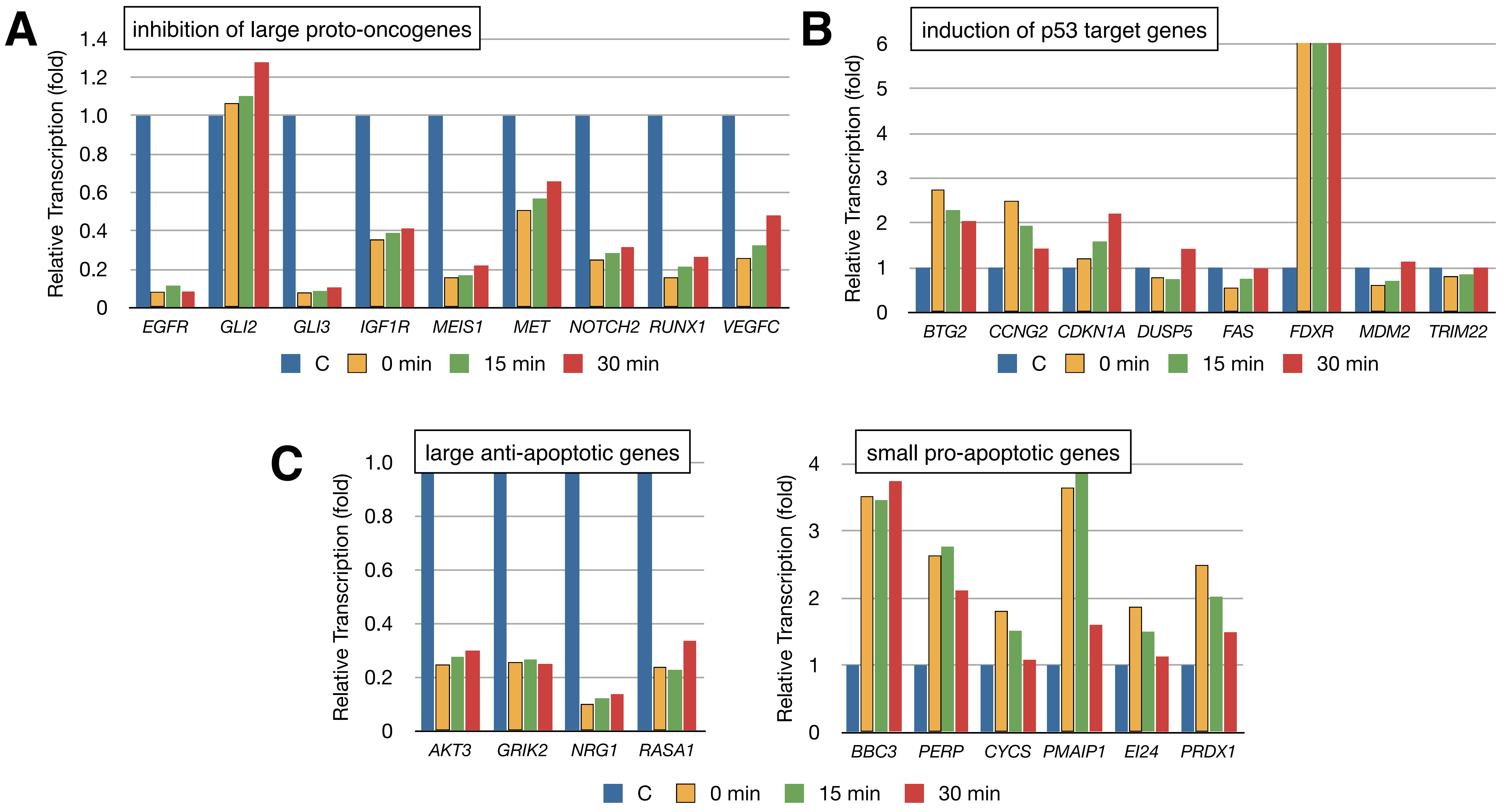

Figure S8. Effect of camptothecin on the expression of genes shown in Figure 7 in CS-B cells. (**A**), large proto-oncogenes inhibited by camptothecin and showing no/slow recovery following drug removal. The *GLI2* gene was unaffected by camptothecin treatment in CS-B cells. (**B**), p53 target genes where only some were induced in CS-B cells following camptothecin treatment. (**C**), examples of large anti-apoptotic genes showing reduced relative transcription (leftt) and examples of small pro-apoptotic genes showing enhanced relative transcription in CS-B cells following camptothecin treatment (right). The data is color coded where blue represents control (C), yellow represents 15 min Bru-labeling at the end of a 45 min camptothecin treatment with no recovery (0 min), green represents drug washout and 15 min Bru-labeling immediately after washout (15 min) and finally red represents labeling 15-30 minutes following washout (30 min).
