# Supplementary material for: Genome-Wide Transcriptional Effects of the Anti-Cancer Agent Camptothecin
Source: PLoS One. 2013 Oct 23;8(10):e78190. doi: 10.1371/journal.pone.0078190 (PMC3806802; doi:10.1371/journal.pone.0078190)
Supplement: Table S1 — Sample statistics. (PDF) [file pone.0078190.s002.pdf]

Table S1. Sample statistics.

run	sample	type	mergedSamples	refSeq	stranded	readCount	mappedReadCount	exon_antisense	intron_antisense	intron_sense	exon_sense	ambiguous
**na**	nf0h3a_3b_4	control	nf0h3a nf0h3b r	hg19	TRUE	171,506,190	123,034,811	476,506	1,615,418	91,749,430	12,825,991	1,847,871
**Run_428**	nfCPT0m1	CPT, 0 min recovery		hg19	TRUE	84,691,817	31,543,500	131,423	523,972	19,443,380	5,755,274	459,586
**Run_428**	nfCPT15m1	CPT, 15 min recovery		hg19	TRUE	76,799,699	40,634,943	208,254	652,892	28,959,558	5,026,714	638,214
**Run_428**	nfCPT30m1	CPT, 30 min recovery		hg19	TRUE	59,058,448	36,041,496	173,590	595,106	26,381,015	3,854,165	564,355
