# Supplementary material for: Genome-Wide Transcriptional Effects of the Anti-Cancer Agent Camptothecin
Source: PLoS One. 2013 Oct 23;8(10):e78190. doi: 10.1371/journal.pone.0078190 (PMC3806802; doi:10.1371/journal.pone.0078190)
Supplement: Table S2 — Up and down regulation of genes following CPT treatment. (PDF) [file pone.0078190.s003.pdf]

Table S2. Up and down regulation of genes following CPT treatment.

Pages 1-14: Table S2A - UP-regulated genes
Pages 15-31: Table S2B - DOWN-regulated genes

Table S2A. Genes UP-regulated >2-fold following 45 min CPT treatment (CPT).

chrom	start	end	name	strand	bp	meanRPKM	meanCount	1_CPT	2_cont	foldChange	log2FoldCha	pAdjusted	significant
chr10	28966423	28971868	**BAMBI**	+	5445	1.366	217	665	68	9.686	3.276	0	1
chr1	192778168	192781407	**RGS2**	+	3239	5.122	482	1404	175	8.002	3	0	1
chr6	31783290	31785719	**HSPA1A**	+	2429	1.145	82	228	33	6.833	2.773	0.002	1
chrX	53111541	53117728	**TSPYL2**	+	6187	3.474	628	1747	255	6.826	2.771	0	1
chr8	57124314	57131176	**CHCHD7**	+	6862	4.007	808	2217	338	6.542	2.71	0	1
chr17	6544221	6547861	**TXNDC17**	+	3640	2.371	255	690	110	6.248	2.643	0	1
chr7	112756772	112758637	**LOC401397**	-	1865	1.777	98	260	44	5.904	2.562	0.003	1
chr5	53751430	53752214	**HSPB3**	+	784	4.354	100	265	45	5.876	2.555	0.004	1
chr11	65265232	65273939	**MALAT1**	+	8707	1086.901	281937	735903	130616	5.634	2.494	0.247	0
chr13	77566058	77576652	**CLN5**	+	10594	2.078	652	1696	304	5.578	2.48	0	1
chrX	37698088	37706889	**DYNLT3**	-	8801	1.657	435	1126	205	5.477	2.453	0	1
chrX	74960372	74962914	**TTC3P1**	-	2542	1.497	114	292	54	5.363	2.423	0.004	1
chr11	62432778	62434923	**METTL12**	+	2145	3.337	211	541	102	5.305	2.407	0.002	1
chr18	57567191	57571538	**PMAIP1**	+	4347	5.3	676	1717	329	5.207	2.381	0	1
chr13	27844463	27847827	**RASL11A**	+	3364	2.705	268	679	131	5.181	2.373	0.002	1
chr9	79000432	79009444	**RFK**	-	9012	1.519	406	1028	198	5.177	2.372	0.001	1
chr18	47007547	47013644	**C18orf32**	-	6097	2.786	505	1280	247	5.171	2.37	0.001	1
chr7	142960521	142966222	**GSTK1**	+	5701	2.214	373	946	182	5.171	2.37	0.001	1
chr17	78109012	78120982	**EIF4A3**	-	11970	5.978	2119	5350	1043	5.129	2.359	0	1
chr19	40928408	40931932	**SERTAD1**	-	3524	8.04	833	2102	410	5.115	2.355	0	1
chr6	97337186	97345767	**NDUFAF4**	-	8581	1.458	373	939	184	5.077	2.344	0.001	1
chr12	120881763	120884215	**TRIAP1**	-	2452	3.526	256	644	127	5.068	2.341	0.002	1
chr9	91605777	91611057	**C9orf47**	+	5280	2.798	438	1099	218	5.03	2.33	0.001	1
chr6	30312905	30314635	**RPP21**	+	1730	1.951	99	248	50	4.945	2.306	0.009	1
chr2	131095815	131099922	**CCDC115**	-	4107	1.084	131	326	66	4.892	2.29	0.007	1
chr17	72858618	72869156	**FDXR**	-	10538	1.797	559	1387	283	4.887	2.289	0.001	1
chr1	110276553	110283660	**GSTM3**	-	7107	2.252	479	1186	244	4.86	2.281	0.001	1
chr2	36581891	36582713	**LOC100288911**	-	822	3.047	73	182	37	4.847	2.277	0.015	1
chr2	220094478	220101391	**ANKZF1**	+	6913	1.85	378	935	193	4.838	2.274	0.002	1
chrX	102883647	102885876	**TCEAL1**	+	2229	2.098	140	344	72	4.736	2.244	0.006	1
chr14	31915242	31926680	**C14orf126**	-	11438	1.342	461	1129	238	4.735	2.243	0.001	1
chr17	40009798	40021629	**KLHL11**	-	11831	1.472	517	1266	268	4.726	2.241	0.001	1
chr2	46844324	46852881	**CRIPT**	+	8557	2.111	542	1324	281	4.707	2.235	0.001	1
chr19	2037469	2051243	**MKNK2**	-	13774	1.899	769	1876	400	4.684	2.228	0.001	1
chr7	108210188	108215294	**DNAJB9**	+	5106	1.219	186	454	97	4.673	2.224	0.005	1
chr5	81569138	81574235	**RPS23**	-	5097	12.85	1961	4768	1025	4.652	2.218	0	1
chrX	102470019	102472128	**BEX4**	+	2109	1.37	85	205	45	4.518	2.176	0.017	1
chr6	144324033	144329867	**HYMAI**	-	5834	2.156	376	902	200	4.493	2.168	0.003	1
chr16	56716381	56718108	**MT1X**	+	1727	1.504	76	184	41	4.492	2.167	0.02	1
chr14	50085406	50087349	**RPL36AL**	-	1943	6.952	403	967	215	4.484	2.165	0.003	1
chr3	160232694	160233024	**SCARNA7**	-	330	15.178	153	367	82	4.463	2.158	0.006	1
chr6	31795511	31798031	**HSPA1B**	+	2520	2.171	163	390	87	4.462	2.158	0.009	1
chr19	48281841	48287943	**SEPW1**	+	6102	3.514	637	1522	342	4.445	2.152	0.002	1
chr10	94449680	94455408	**HHEX**	+	5728	1.001	169	403	91	4.432	2.148	0.01	1
chr12	6619387	6619717	**SCARNA10**	+	330	13.613	138	328	74	4.409	2.14	0.006	1
chrX	20154183	20154531	**SCARNA9L**	-	348	1.151	12	29	6	4.391	2.135	0.071	1
chr8	56980738	56987140	**RPS20**	-	6402	6.376	1223	2904	662	4.383	2.132	0.001	1
chr6	89790428	89794879	**PNRC1**	+	4451	7.801	1032	2445	561	4.355	2.123	0.001	1
chr15	44092618	44094769	**C15orf63**	+	2151	3.067	197	467	107	4.343	2.119	0.008	1
chr19	35225479	35233774	**ZNF181**	+	8295	1.547	383	906	208	4.34	2.118	0.004	1
chr12	6571403	6579843	**VAMP1**	-	8440	3.796	956	2259	522	4.327	2.113	0.001	1
chr17	56160779	56167618	**DYNLL2**	+	6839	6.153	1255	2963	686	4.318	2.11	0.001	1
chr6	99968869	99981059	**LOC100130890**	+	12190	1.87	686	1613	377	4.273	2.095	0.002	1
chr3	42632297	42636490	**SS18L2**	+	4193	2.367	296	693	164	4.229	2.08	0.006	1
chr3	158384202	158390482	**LXN**	-	6280	1.068	201	470	111	4.228	2.08	0.009	1
chr2	55459038	55462989	**RPS27A**	+	3951	6.13	726	1698	403	4.213	2.075	0.002	1
chr5	72848024	72861511	**ANKRA2**	-	13487	1.368	558	1304	309	4.211	2.074	0.002	1
chr10	95653729	95662491	**SLC35G1**	+	8762	1.361	358	837	198	4.21	2.074	0.004	1
chr2	24290453	24299314	**SF3B14**	-	8861	2.31	617	1440	342	4.209	2.073	0.002	1

chr11	22843597	22851382	**SVIP**	-	7785	1.061	245	572	136	4.207	2.073	0.009		1
chr15	40327890	40331389	**SRP14**	-	3499	5.521	583	1356	325	4.166	2.059	0.002		1
chr4	6675820	6677774	**LOC93622**	+	1954	1.487	86	200	48	4.148	2.052	0.025		1
chr6	150385742	150390202	**ULBP3**	-	4460	1.014	134	312	75	4.147	2.052	0.018		1
chr13	111365082	111373421	**ING1**	+	8339	1.927	477	1108	267	4.145	2.051	0.004		1
chr3	180701497	180707562	**DNAJC19**	-	6065	1.57	285	662	159	4.145	2.051	0.007		1
chr2	198364720	198368187	**HSPE1**	+	3467	2.46	254	589	142	4.141	2.05	0.009		1
chr17	8076296	8079714	**TMEM107**	-	3418	2.678	277	643	155	4.131	2.047	0.006		1
chrX	100878119	100882831	**ARMCX3**	+	4712	4.16	592	1371	332	4.127	2.045	0.003		1
chr7	92158086	92166823	**RBM48**	+	8737	2.454	648	1499	364	4.114	2.041	0.002		1
chr1	115110180	115124265	**BCAS2**	-	14085	1.653	702	1624	395	4.112	2.04	0.002		1
chr10	75541807	75543406	**CHCHD1**	+	1599	2.246	108	251	61	4.107	2.038	0.017		1
chr9	35732316	35737005	**CREB3**	+	4689	3.094	433	1001	244	4.102	2.036	0.005		1
chr5	156565450	156569921	**MED7**	-	4471	1.932	261	603	147	4.091	2.032	0.007		1
chr1	153963238	153964631	**RPS27**	+	1393	14.908	622	1430	352	4.059	2.021	0.003		1
chr1	145507556	145513535	**RBM8A**	+	5979	2.31	414	952	234	4.058	2.021	0.005		1
chrX	100600643	100603957	**TIMM8A**	-	3314	1.067	106	244	60	4.057	2.02	0.021		1
chr10	22605311	22609246	**COMMD3**	+	3935	2.428	287	658	163	4.034	2.012	0.008		1
chr18	47340392	47340813	**SCARNA17**	+	421	1.043	13	30	7	4.018	2.007	0.081		1
chr9	33025208	33039062	**DNAJA1**	+	13854	6.356	2650	6069	1510	4.017	2.006	0.002		1
chr1	89517986	89531043	**GBP1**	-	13057	1.012	397	910	226	4.016	2.006	0.006		1
chr3	44771123	44778575	**ZNF501**	+	7452	1.195	267	612	152	4.003	2.001	0.009		1
chr19	40029446	40030838	**EID2**	-	1392	2.729	113	259	65	3.994	1.998	0.023		1
chr17	77806954	77813213	**CBX4**	-	6259	2.737	508	1160	290	3.99	1.996	0.006		1
chr4	186064416	186071538	**SLC25A4**	+	7122	1.346	287	656	164	3.984	1.994	0.009		1
chr7	10971579	10979813	**NDUFA4**	-	8234	4.167	1040	2368	597	3.962	1.986	0.002		1
chr12	121016847	121019201	**POP5**	-	2354	1.663	117	267	67	3.946	1.98	0.022		1
chr3	53919225	53925989	**SELK**	-	6764	2.28	464	1054	267	3.941	1.978	0.005		1
chr15	83654994	83659423	**FAM103A1**	+	4429	1.477	196	445	113	3.926	1.973	0.015		1
chr15	42834719	42841002	**LRRC57**	-	6283	1.81	343	777	198	3.909	1.967	0.008		1
chr10	66585284	66586634	**ANXA2P3**	+	1350	3.288	133	301	77	3.903	1.965	0.021		1
chr4	89617065	89619023	**NAP1L5**	-	1958	1.086	64	145	37	3.877	1.955	0.036		1
chr3	170582664	170588045	**RPL22L1**	-	5381	10.07	1628	3671	947	3.875	1.954	0.003		1
chr13	25670275	25672704	**PABPC3**	+	2429	1.142	84	189	49	3.837	1.94	0.028		1
chr2	86426555	86440477	**MRPL35**	+	13922	1.494	629	1412	368	3.833	1.938	0.004		1
chr1	40974432	40982214	**DEM1**	+	7782	1.333	311	699	182	3.824	1.935	0.011		1
chr4	89442134	89444955	**PIGY**	-	2821	4.16	354	791	208	3.788	1.921	0.009		1
chr12	56660641	56664750	**COQ10A**	+	4109	1.178	143	320	84	3.787	1.921	0.027		1
chrX	69506210	69509798	**PDZD11**	-	3588	1.364	147	328	86	3.781	1.919	0.023		1
chr12	56704212	56710006	**CNPY2**	-	5916	2.41	419	934	247	3.766	1.913	0.009		1
chrX	55478537	55479999	**MAGEH1**	+	1462	1.239	53	119	31	3.748	1.906	0.06		1
chr14	61438166	61447782	**TRMT5**	-	9616	1.41	412	915	244	3.743	1.904	0.008		1
chr10	104613966	104624718	**C10orf32**	+	10752	1.995	651	1446	386	3.741	1.903	0.005		1
chr6	86386724	86388451	**SNHG5**	-	1727	26.254	1368	3037	812	3.739	1.903	0.003		1
chr15	45003684	45010357	**B2M**	+	6673	19.617	3955	8775	2349	3.736	1.901	0.005		1
chr2	120436742	120439694	**TMEM177**	+	2952	1.444	126	281	75	3.731	1.9	0.03		1
chr6	3153901	3157783	**TUBB2A**	-	3882	4.597	534	1185	318	3.726	1.898	0.008		1
chr19	58011308	58019510	**ZNF773**	+	8202	1.433	352	779	209	3.719	1.895	0.012		1
chr7	25158269	25164980	**CYCS**	-	6711	4.62	935	2067	557	3.708	1.891	0.004		1
chr3	23958638	23962332	**RPL15**	+	3694	11.041	1233	2724	737	3.696	1.886	0.004		1
chr15	40985952	40987303	**LOC100505648**	-	1351	2.082	83	185	50	3.693	1.885	0.042		1
chr1	8071778	8086393	**ERRFI1**	-	14615	2.383	1045	2306	624	3.691	1.884	0.005		1
chr2	74682149	74685087	**INO80B**	+	2938	1.062	94	207	56	3.681	1.88	0.036		1
chrX	119759528	119764005	**C1GALT1C1**	-	4477	1.27	172	379	103	3.68	1.88	0.021		1
chr7	30052883	30066268	**FKBP14**	-	13385	1.272	516	1137	309	3.673	1.877	0.007		1
chr12	25348149	25357949	**LYRM5**	+	9800	1.378	409	900	245	3.668	1.875	0.009		1
chr12	6601315	6602471	**MRPL51**	-	1156	5.579	194	427	116	3.661	1.872	0.021		1
chr9	127997126	128003666	**HSPA5**	-	6540	10.245	2032	4455	1224	3.638	1.863	0.004		1
chr14	76117232	76127538	**C14orf1**	-	10306	1.259	390	855	235	3.634	1.862	0.012		1
chr6	153308399	153323925	**MTRF1L**	-	15526	2.59	1217	2665	735	3.625	1.858	0.004		1
chrX	102840418	102842655	**TCEAL4**	+	2237	9.948	674	1475	407	3.619	1.856	0.006		1
chr4	120217573	120225600	**C4orf3**	-	8027	2.207	539	1173	327	3.582	1.841	0.008		1
chr9	132589563	132597572	**C9orf78**	-	8009	1.639	396	861	241	3.571	1.836	0.012		1
chr12	49490922	49504680	**LMBR1L**	-	13758	1.183	489	1063	298	3.565	1.834	0.011		1
chr16	87425800	87438380	**MAP1LC3B**	+	12580	3.866	1462	3175	891	3.561	1.832	0.006		1
chr1	110943876	110950546	**HBXIP**	-	6670	3.562	719	1561	438	3.56	1.832	0.007		1
chr5	98264837	98266713	**LOC100289230**	+	1876	3.241	182	397	111	3.56	1.832	0.027		1
chr5	74063102	74072734	**NSA2**	+	9632	1.783	527	1144	321	3.555	1.83	0.007		1
chr16	57279037	57287545	**ARL2BP**	+	8508	3.542	913	1981	557	3.551	1.828	0.006		1
chr12	53342654	53346685	**KRT18**	+	4031	2.065	254	552	155	3.544	1.826	0.016		1
chr4	71554195	71556268	**UTP3**	+	2073	5.706	360	781	220	3.544	1.825	0.012		1

chr12	6976583	6980110	**TPI1**	+	3527	4	422	915	258	3.541	1.824	0.014	1
chr8	38846326	38854041	**TM2D2**	-	7715	2.672	623	1350	381	3.54	1.824	0.008	1
chr1	203274663	203278729	**BTG2**	+	4066	6.931	835	1808	511	3.535	1.822	0.009	1
chr6	52535883	52551385	**TMEM14A**	+	15502	1.235	581	1257	356	3.525	1.818	0.008	1
chr4	113196781	113207059	**TIFA**	-	10278	1.041	322	695	197	3.52	1.816	0.018	1
chr9	131445933	131458675	**SET**	+	12742	2.51	967	2089	594	3.516	1.814	0.006	1
chr12	107371068	107380929	**MTERFD3**	-	9861	1.946	587	1266	360	3.512	1.812	0.008	1
chr2	176040985	176046490	**ATP5G3**	-	5505	6.104	1018	2195	625	3.509	1.811	0.006	1
chr5	54455983	54463129	**GPX8**	+	7146	6.328	1387	2989	853	3.503	1.809	0.004	1
chr14	24612573	24615855	**PSME2**	-	3282	1.899	187	403	115	3.501	1.808	0.029	1
chr8	97239303	97247862	**UQCRB**	-	8559	2.586	672	1446	413	3.497	1.806	0.008	1
chr1	43849587	43855483	**MED8**	-	5896	5.063	905	1948	557	3.495	1.805	0.006	1
chr16	3348807	3355439	**TIGD7**	-	6632	1.297	259	558	160	3.486	1.801	0.021	1
chr7	42948871	42951689	**C7orf25**	-	2818	1.802	153	330	94	3.48	1.799	0.031	1
chr20	30532757	30539883	**PDRG1**	-	7126	2.041	434	932	268	3.467	1.794	0.017	1
chr9	115804173	115818996	**ZFP37**	-	14823	1.6	718	1539	445	3.458	1.79	0.009	1
chr1	109642814	109643234	**SCARNA2**	+	420	12.641	157	337	97	3.448	1.786	0.041	1
chr12	7053202	7055165	**C12orf57**	+	1963	6.728	395	844	245	3.445	1.785	0.019	1
chr2	207024317	207027653	**EEF1B2**	+	3336	3.855	390	834	242	3.443	1.784	0.015	1
chr12	56435685	56438007	**RPS26**	+	2322	9.992	703	1504	437	3.442	1.783	0.009	1
chr20	58508818	58523702	**C20orf177**	+	14884	1.927	872	1863	542	3.437	1.781	0.007	1
chr4	101107026	101111655	**DDIT4L**	-	4629	1.458	204	436	127	3.425	1.776	0.027	1
chr10	60145175	60155897	**TFAM**	+	10722	2.481	805	1716	501	3.422	1.775	0.009	1
chr2	37423634	37431886	**LOC100505876**	+	8252	2.292	574	1224	358	3.421	1.774	0.011	1
chrX	118920468	118925606	**RPL39**	-	5138	4.73	735	1565	458	3.414	1.771	0.009	1
chr13	108859791	108870716	**LIG4**	-	10925	1.154	384	816	239	3.404	1.767	0.016	1
chr4	140374960	140397069	**RAB33B**	+	22109	1.44	960	2041	600	3.399	1.765	0.009	1
chr5	2746278	2751769	**IRX2**	-	5491	1.701	279	591	174	3.385	1.759	0.028	1
chr11	57471186	57479673	**MED19**	-	8487	1.81	464	985	291	3.382	1.758	0.015	1
chr1	110009099	110024764	**SYPL2**	+	15665	1.469	691	1466	433	3.381	1.758	0.012	1
chr21	38437663	38445458	**PIGP**	-	7795	1.498	356	754	223	3.374	1.754	0.017	1
chr1	43232915	43241413	**C1orf50**	+	8498	1.587	410	867	257	3.369	1.753	0.016	1
chr12	11323779	11325723	**LOC100129361**	+	1944	4.039	238	505	149	3.369	1.752	0.026	1
chr14	90863326	90874619	**CALM1**	+	11293	10.857	3720	7870	2337	3.366	1.751	0.01	1
chr1	156638555	156647189	**NES**	-	8634	1.898	495	1047	311	3.364	1.75	0.015	1
chr12	77252495	77272799	**CSRP2**	-	20304	2.437	1505	3179	946	3.358	1.747	0.007	1
chr5	140560979	140565796	**PCDHB16**	+	4817	1.301	188	397	118	3.357	1.747	0.035	1
chr1	244998638	245008359	**FAM36A**	+	9721	4.113	1217	2570	766	3.355	1.747	0.008	1
chr19	56132106	56135941	**ZNF784**	-	3835	1.093	125	265	79	3.343	1.741	0.05	1
chr12	54104901	54121307	**CALCOCO1**	-	16406	1.191	592	1248	373	3.342	1.741	0.013	1
chr20	54933982	54943718	**FAM210B**	+	9736	1.293	379	798	239	3.338	1.739	0.021	1
chr2	224822120	224832431	**MRPL44**	+	10311	2.961	933	1966	589	3.336	1.738	0.008	1
chr1	25568739	25573985	**C1orf63**	-	5246	17.074	2711	5706	1713	3.331	1.736	0.01	1
chr1	27992571	27998724	**IFI6**	-	6153	1.181	216	455	136	3.328	1.735	0.037	1
chrX	71346960	71351751	**RGAG4**	-	4791	1.166	166	350	105	3.327	1.734	0.045	1
chr6	16129316	16148478	**MYLIP**	+	19162	1.39	793	1669	502	3.324	1.733	0.015	1
chr3	101280711	101285089	**RG9MTD1**	+	4378	3.165	422	887	267	3.324	1.733	0.017	1
chr3	150259779	150264428	**SERP1**	-	4649	6.895	972	2039	616	3.309	1.726	0.01	1
chr1	226250407	226259703	**H3F3A**	+	9296	5.592	1592	3341	1010	3.308	1.726	0.007	1
chr3	58413356	58419579	**PDHB**	-	6223	5.268	1001	2094	636	3.29	1.718	0.009	1
chr20	48599512	48605420	**SNAI1**	+	5908	3.116	554	1159	353	3.284	1.716	0.017	1
chr13	76099349	76111991	**COMMD6**	-	12642	1.897	729	1524	464	3.281	1.714	0.012	1
chr6	17600517	17611950	**FAM8A1**	+	11433	1.045	362	756	230	3.28	1.714	0.023	1
chr5	60453535	60458302	**C5orf43**	-	4767	5.393	782	1634	498	3.278	1.713	0.011	1
chr6	116832807	116839709	**FAM26E**	+	6902	1.552	328	686	209	3.275	1.711	0.021	1
chr7	93551015	93555826	**GNG11**	+	4811	4.219	622	1299	397	3.273	1.71	0.012	1
chr19	47724078	47736023	**BBC3**	-	11945	3.276	1168	2438	745	3.271	1.71	0.013	1
chr3	129149792	129158852	**MBD4**	-	9060	3.53	969	2023	618	3.271	1.71	0.011	1
chrX	75392770	75398033	**CXorf26**	+	5263	2.899	461	962	294	3.263	1.706	0.02	1
chr6	44225902	44233525	**NFKBIE**	-	7623	1.349	307	639	196	3.251	1.701	0.032	1
chrX	102507922	102510121	**TCEAL8**	-	2199	3.865	258	538	165	3.249	1.7	0.029	1
chr7	27135712	27139877	**HOTAIRM1**	+	4165	2.07	258	537	165	3.248	1.7	0.035	1
chr6	31321648	31324989	**HLA-B**	-	3341	2.399	239	498	153	3.247	1.699	0.038	1
chr18	47014850	47018935	**RPL17**	-	4085	11.248	1405	2919	901	3.24	1.696	0.009	1
chr17	7123152	7128585	**ACADVL**	+	5433	5.371	885	1837	567	3.237	1.694	0.012	1
chr3	16298567	16306496	**DPH3**	-	7929	3.106	751	1559	482	3.234	1.693	0.012	1
chr9	111696672	111703237	**FAM206A**	+	6565	1.082	215	445	138	3.231	1.692	0.036	1
chr4	119199916	119200978	**SNHG8**	+	1062	25.882	831	1723	533	3.231	1.692	0.014	1
chr2	170550963	170558218	**PHOSPHO2**	+	7255	1.427	313	649	201	3.226	1.69	0.029	1
chr6	56911383	56920023	**KIAA1586**	+	8640	3.654	961	1991	618	3.221	1.687	0.011	1
chr10	30722949	30750762	**MAP3K8**	+	27813	1.071	898	1860	577	3.22	1.687	0.014	1

chr16	72118755	72128215	**TXNL4B**	-	9460	1.401	399	827	257	3.22	1.687	0.025	1
chr16	50059188	50070999	**TMEM188**	+	11811	1.879	674	1396	433	3.218	1.686	0.015	1
chr9	71394963	71398609	**FAM122A**	+	3646	2.948	323	668	208	3.203	1.679	0.031	1
chr4	53578620	53580305	**SNHG13**	+	1685	6.151	311	643	200	3.202	1.679	0.033	1
chr14	96001322	96011055	**GLRX5**	+	9733	2.054	609	1258	393	3.198	1.677	0.016	1
chr11	59573607	59578345	**MRPL16**	-	4738	2.475	357	737	231	3.19	1.674	0.025	1
chrX	118370210	118378429	**PGRMC1**	+	8219	5.73	1434	2954	927	3.184	1.671	0.011	1
chr3	52739856	52742197	**SPCS1**	+	2341	3.598	254	524	165	3.172	1.665	0.036	1
chr10	44139306	44144326	**ZNF32**	-	5020	1.408	216	444	140	3.171	1.665	0.035	1
chr2	170655388	170668575	**SSB**	+	13187	2.082	839	1723	544	3.168	1.663	0.013	1
chr1	159997461	160001783	**PIGM**	-	4322	1.148	149	307	97	3.166	1.663	0.054	1
chr12	57984941	57997211	**PIP4K2C**	+	12270	1.672	621	1273	404	3.152	1.656	0.019	1
chr5	131817300	131826465	**IRF1**	-	9165	2.505	689	1411	448	3.145	1.653	0.021	1
chr5	43042235	43045370	**LOC153684**	+	3135	1.991	188	385	122	3.145	1.653	0.049	1
chr12	120907659	120936298	**DYNLL1**	+	28639	1.01	879	1798	572	3.141	1.651	0.015	1
chr12	66516848	66524533	**LLPH**	-	7685	3.263	762	1559	496	3.141	1.651	0.016	1
chr19	58125829	58133636	**ZNF134**	+	7807	4.004	951	1946	619	3.14	1.651	0.014	1
chr3	112709799	112720221	**GTPBP8**	+	10422	2.168	691	1414	451	3.136	1.649	0.016	1
chr12	123745539	123756687	**CDK2AP1**	-	11148	2.311	782	1598	510	3.135	1.648	0.017	1
chr2	70523107	70529220	**FAM136A**	-	6113	1.562	289	590	189	3.127	1.645	0.035	1
chr8	23536205	23540450	**NKX3-1**	-	4245	1.095	139	283	90	3.121	1.642	0.065	1
chr15	43477465	43489375	**CCNDBP1**	+	11910	2.93	1062	2167	694	3.12	1.642	0.014	1
chr8	117778741	117786921	**UTP23**	+	8180	3.035	760	1550	496	3.12	1.642	0.015	1
chr9	96208781	96215874	**FAM120AOS**	-	7093	1.599	344	702	225	3.119	1.641	0.031	1
chr3	40351172	40353915	**EIF1B**	+	2743	4.231	348	711	228	3.117	1.64	0.035	1
chr11	93454679	93455032	**SCARNA9**	+	353	2.336	25	52	16	3.115	1.639	0.123	0
chr20	18548072	18550203	**LINC00493**	+	2131	2.585	167	340	109	3.108	1.636	0.052	1
chr15	63445538	63449741	**RPS27L**	-	4203	10.447	1337	2718	876	3.1	1.632	0.014	1
chr8	101162838	101166230	**POLR2K**	+	3392	3.278	343	697	225	3.094	1.63	0.027	1
chr5	139929652	139937678	**SRA1**	-	8026	4.071	998	2024	656	3.083	1.625	0.015	1
chr9	19376253	19380235	**RPS6**	-	3982	18.047	2204	4467	1449	3.082	1.624	0.014	1
chr12	54356095	54362515	**HOTAIR**	-	6420	3.762	735	1489	483	3.08	1.623	0.019	1
chr11	125439297	125454575	**EI24**	+	15278	2.15	994	2012	654	3.073	1.62	0.018	1
chr20	5095598	5107268	**PCNA**	-	11670	4.674	1652	3343	1089	3.069	1.618	0.016	1
chr6	138409641	138428660	**PERP**	-	19019	1.749	1010	2042	666	3.064	1.615	0.017	1
chr2	70485230	70508317	**PCYOX1**	+	23087	1.077	755	1525	498	3.062	1.615	0.02	1
chr14	93651295	93653431	**C14orf109**	+	2136	1.302	83	168	54	3.062	1.615	0.092	1
chr5	137673223	137685418	**FAM53C**	+	12195	4.111	1510	3050	996	3.061	1.614	0.018	1
chr13	21750371	21753220	**MRP63**	+	2849	1.398	120	242	79	3.058	1.613	0.07	1
chr2	120124503	120130122	**DBI**	+	5619	3.374	569	1148	375	3.055	1.611	0.029	1
chr11	112097087	112104695	**PTS**	+	7608	5.864	1365	2754	903	3.05	1.609	0.015	1
chr6	160211491	160219461	**MRPL18**	+	7970	2.242	546	1098	361	3.036	1.602	0.024	1
chr8	125551342	125562227	**NDUFB9**	+	10885	2.61	864	1739	573	3.035	1.602	0.019	1
chr17	37006320	37010053	**RPL23**	-	3733	11.299	1285	2585	851	3.035	1.602	0.016	1
chr14	92582467	92588153	**NDUFB1**	-	5686	2.213	384	773	254	3.034	1.601	0.031	1
chr6	21593971	21598849	**SOX4**	+	4878	1.28	190	382	126	3.03	1.599	0.052	1
chr7	54819939	54826939	**SEC61G**	-	7000	6.199	1327	2668	881	3.029	1.599	0.015	1
chr5	34915819	34925787	**BRIX1**	+	9968	3.448	1050	2110	697	3.027	1.598	0.017	1
chr17	7155371	7163259	**C17orf81**	+	7888	1.426	341	685	226	3.027	1.598	0.037	1
chr1	25548766	25559013	**SYF2**	-	10247	2.469	774	1554	514	3.02	1.594	0.019	1
chr5	140071017	140078890	**HARS2**	+	7873	3.462	827	1660	549	3.019	1.594	0.021	1
chr5	162930230	162946328	**MAT2B**	+	16098	1.936	955	1916	635	3.014	1.592	0.017	1
chr5	159828647	159846168	**SLU7**	-	17521	2.067	1114	2232	741	3.012	1.591	0.015	1
chr1	153946744	153950451	**JTB**	-	3707	3.611	403	809	268	3.012	1.591	0.036	1
chr9	22002901	22009312	**CDKN2B**	-	6411	1.728	335	672	223	3.009	1.589	0.039	1
chr10	75532048	75535976	**FUT11**	+	3928	2.399	283	567	188	3.009	1.589	0.048	1
chr3	58477822	58488087	**KCTD6**	+	10265	1.738	544	1089	362	3.004	1.587	0.027	1
chr1	161123533	161128646	**UFC1**	+	5113	2.327	363	727	242	3.002	1.586	0.034	1
chr17	7486964	7491527	**MPDU1**	+	4563	2.003	276	554	184	3.002	1.586	0.046	1
chr20	5918485	5931173	**TRMT6**	-	12688	1.569	604	1209	403	3	1.585	0.027	1
chr13	21714652	21723224	**SAP18**	+	8572	4.675	1229	2458	819	3	1.585	0.016	1
chr12	108956293	108963160	**ISCU**	+	6867	4.327	900	1801	600	3	1.585	0.021	1
chrX	71492452	71497141	**RPS4X**	-	4689	6.424	921	1842	614	2.998	1.584	0.019	1
chrX	118708498	118718379	**UBE2A**	+	9881	3.487	1052	2101	702	2.991	1.581	0.019	1
chr11	62559597	62572964	**NXF1**	-	13367	4.132	1666	3326	1112	2.989	1.58	0.021	1
chr10	102033712	102046439	**BLOC1S2**	-	12727	2.898	1130	2255	755	2.986	1.578	0.017	1
chr10	99195665	99205768	**EXOSC1**	-	10103	1.246	383	765	256	2.986	1.578	0.035	1
chr2	190526124	190535557	**ASNSD1**	+	9433	3.166	919	1833	614	2.985	1.578	0.018	1
chr1	28905049	28908366	**SNHG12**	-	3317	16.287	1634	3258	1092	2.982	1.576	0.021	1
chr22	39077953	39080766	**TOMM22**	+	2813	2.726	233	465	156	2.982	1.576	0.049	1
chr4	4237268	4249934	**TMEM128**	-	12666	1.435	553	1102	370	2.98	1.575	0.028	1

chr12	88429267	88443937	**C12orf29**	+	14670	1.818	810	1615	542	2.978	1.574	0.023	1
chr8	74888376	74895018	**TMEM70**	+	6642	2.021	407	811	272	2.977	1.574	0.036	1
chr15	49170289	49172380	**EID1**	+	2091	12.323	790	1573	528	2.976	1.573	0.021	1
chr5	44809026	44815618	**MRPS30**	+	6592	2.652	529	1054	354	2.974	1.572	0.031	1
chr19	18496967	18499986	**GDF15**	+	3019	8.882	795	1584	532	2.973	1.572	0.033	1
chr5	140024947	140027370	**NDUFA2**	-	2423	2.156	157	313	105	2.971	1.571	0.069	1
chr4	6717841	6719387	**CNO**	+	1546	2.029	93	186	62	2.961	1.566	0.099	1
chr17	73772514	73775860	**H3F3B**	-	3346	40.926	4162	8267	2794	2.959	1.565	0.027	1
chr17	34842472	34851662	**ZNHIT3**	+	9190	1.637	453	900	304	2.953	1.562	0.037	1
chrX	100910267	100914863	**ARMCX2**	-	4596	1.291	181	359	121	2.953	1.562	0.06	1
chr11	85339621	85347583	**TMEM126B**	+	7962	2.077	502	996	337	2.95	1.561	0.033	1
chr6	29855382	29858856	**HLA-H**	+	3474	1.331	138	275	93	2.95	1.561	0.079	1
chr4	140211070	140223705	**NDUFC1**	-	12635	1.368	531	1052	357	2.945	1.558	0.027	1
chr19	12175545	12188626	**ZNF844**	+	13081	1.654	662	1312	445	2.943	1.557	0.025	1
chr1	115312104	115323308	**SIKE1**	-	11204	1.748	600	1188	404	2.938	1.555	0.027	1
chr12	6875540	6880118	**PTMS**	+	4578	7.3	1009	1998	680	2.937	1.554	0.025	1
chr7	108202670	108210167	**THAP5**	-	7497	1.864	429	848	289	2.93	1.551	0.033	1
chr17	43224683	43229468	**HEXIM1**	+	4785	3.876	561	1109	378	2.93	1.551	0.033	1
chr20	18774692	18776709	**LOC100270804**	+	2017	1.1	68	134	46	2.926	1.549	0.097	1
chr10	28808845	28821283	**LOC220906**	-	12438	3.091	1176	2323	794	2.924	1.548	0.02	1
chr19	44529493	44537262	**ZNF222**	+	7769	1.089	255	505	172	2.924	1.548	0.056	1
chr5	176730834	176733950	**PRELID1**	+	3116	1.642	154	305	104	2.917	1.544	0.074	1
chr11	66432469	66445275	**RBM4B**	-	12806	1.002	391	771	265	2.91	1.541	0.039	1
chr12	56211805	56214959	**ORMDL2**	+	3154	2.949	284	560	192	2.91	1.541	0.048	1
chr12	14927269	14930936	**H2AFJ**	+	3667	3.904	434	855	293	2.909	1.541	0.04	1
chr14	77924372	77935815	**AHSA1**	+	11443	1.875	652	1283	441	2.906	1.539	0.03	1
chr2	233412778	233415226	**TIGD1**	-	2448	1.687	126	247	85	2.901	1.537	0.077	1
chr1	24104875	24114722	**PITHD1**	+	9847	2.25	676	1328	458	2.898	1.535	0.029	1
chr22	19428409	19435755	**C22orf39**	-	7346	2.001	447	878	303	2.895	1.534	0.039	1
chr17	37885408	37886788	**MIEN1**	-	1380	3.289	137	269	93	2.891	1.531	0.084	1
chr4	110736665	110745893	**GAR1**	+	9228	1.801	511	1002	348	2.881	1.526	0.031	1
chrX	54466852	54471731	**TSR2**	+	4879	1.772	263	516	179	2.881	1.527	0.055	1
chr14	23564682	23569665	**C14orf119**	+	4983	2.85	430	843	293	2.878	1.525	0.043	1
chr11	34460471	34493607	**CAT**	+	33136	1.052	1069	2094	727	2.877	1.525	0.023	1
chr4	83814604	83822069	**LOC100499177**	-	7465	4.177	956	1873	651	2.876	1.524	0.024	1
chr2	32248971	32264844	**DPY30**	-	15873	1.103	533	1043	363	2.875	1.524	0.035	1
chr19	58144534	58154147	**ZNF211**	+	9613	1.732	506	991	344	2.875	1.523	0.037	1
chr3	40498800	40503859	**RPL14**	+	5059	5.818	904	1769	615	2.874	1.523	0.024	1
chr11	10772810	10801290	**CTR9**	+	28480	1.804	1579	3090	1076	2.871	1.521	0.021	1
chr6	30029035	30032686	**ZNRD1**	+	3651	1.298	143	281	98	2.87	1.521	0.081	1
chr17	6915735	6917463	**RNASEK**	+	2117	2.132	111	216	75	2.866	1.519	0.098	1
chr12	48541571	48551377	**ASB8**	-	9806	2.115	634	1238	432	2.863	1.518	0.031	1
chr10	133781203	133795435	**BNIP3**	-	14232	2.33	1005	1963	686	2.86	1.516	0.028	1
chr15	66791652	66797193	**RPL4**	-	5541	10.398	1764	3442	1204	2.857	1.515	0.023	1
chr6	72124148	72130448	**LINC00472**	-	6300	3.77	723	1411	494	2.856	1.514	0.031	1
chr5	85913783	85916583	**COX7C**	+	2800	3.247	277	540	189	2.854	1.513	0.056	1
chr17	7571719	7590863	**TP53**	-	19144	1.535	894	1742	611	2.851	1.511	0.029	1
chrX	20004934	20007897	**LOC729609**	-	2963	1.56	140	273	96	2.846	1.509	0.084	1
chr3	186288466	186303589	**DNAJB11**	+	15123	2.181	1007	1959	689	2.842	1.507	0.027	1
chr7	56019610	56023033	**MRPS17**	+	3423	1.166	122	238	83	2.842	1.507	0.081	1
chr2	209100952	209119806	**IDH1**	-	18854	1.353	772	1502	528	2.841	1.506	0.033	1
chr2	37458773	37476303	**C2orf56**	+	17530	1.255	678	1319	465	2.834	1.503	0.028	1
chr14	20915206	20923267	**OSGEP**	-	8061	1.553	380	739	260	2.834	1.503	0.049	1
chr9	104237607	104249475	**C9orf125**	-	11868	2.002	722	1403	495	2.832	1.502	0.033	1
chr19	50431958	50437193	**ATF5**	+	5235	2.96	468	910	321	2.832	1.502	0.046	1
chr8	110346551	110358189	**ENY2**	+	11638	3.44	1223	2375	839	2.83	1.501	0.026	1
chr5	54824669	54830370	**RNF138P1**	-	5701	2.54	443	861	304	2.829	1.501	0.041	1
chrX	101854095	101859085	**ARMCX5**	+	4990	1.234	189	367	129	2.826	1.499	0.066	1
chr6	151773421	151791232	**C6orf211**	+	17811	1.427	779	1512	535	2.824	1.498	0.029	1
chr11	66104803	66112582	**BRMS1**	-	7779	2.107	494	959	339	2.824	1.498	0.046	1
chr11	6701615	6704632	**MRPL17**	-	3017	3.032	276	536	190	2.818	1.495	0.064	1
chr4	15683351	15692070	**FAM200B**	+	8719	4.057	1094	2119	752	2.816	1.494	0.023	1
chr13	103418462	103426149	**C13orf27**	-	7687	1.503	353	684	243	2.815	1.493	0.05	1
chrX	102611379	102613397	**WBP5**	+	2018	7.022	433	839	298	2.814	1.493	0.043	1
chr6	52529198	52533951	**LOC730101**	+	4753	2.975	433	838	298	2.813	1.492	0.043	1
chr9	130209952	130213711	**RPL12**	-	3759	14.196	1619	3131	1114	2.809	1.49	0.03	1
chr6	2833733	2842081	**SERPINB1**	-	8348	3.312	847	1638	583	2.808	1.49	0.029	1
chr1	32799439	32801834	**MARCKSL1**	-	2395	2.548	182	352	125	2.807	1.489	0.092	1
chr7	87834431	87856308	**SRI**	-	21877	1.892	1265	2446	871	2.806	1.489	0.027	1
chr3	111697827	111712210	**ABHD10**	+	14383	1.939	859	1659	592	2.801	1.486	0.028	1
chrX	135044230	135056134	**MMGT1**	-	11904	1.706	620	1199	428	2.801	1.486	0.035	1

chr1	247263263	247267674	**ZNF669**	-	4411	3.422	459	887	316	2.801	1.486	0.046	1
chr17	42253351	42264082	**C17orf65**	-	10731	1.266	411	795	283	2.8	1.485	0.052	1
chr17	48556189	48563336	**RSAD1**	+	7147	1.236	265	512	183	2.797	1.484	0.072	1
chr7	39606002	39612480	**YAE1D1**	+	6478	2.329	465	898	321	2.795	1.483	0.038	1
chr4	159630278	159644552	**PPID**	-	14274	1.689	741	1430	512	2.793	1.482	0.031	1
chr14	78174424	78183941	**SLIRP**	+	9517	2.578	754	1452	522	2.783	1.476	0.031	1
chr13	48611702	48621282	**NUDT15**	+	9580	1.595	466	897	322	2.782	1.476	0.046	1
chr1	154521050	154531120	**UBE2Q1**	-	10070	2.86	880	1693	609	2.78	1.475	0.032	1
chr1	156692412	156697705	**ISG20L2**	-	5293	2.907	468	900	323	2.78	1.475	0.048	1
chr1	150255228	150259501	**C1orf51**	+	4273	2.205	286	550	198	2.774	1.472	0.065	1
chr12	57031958	57039852	**ATP5B**	-	7894	8.232	1983	3810	1374	2.772	1.471	0.03	1
chr19	17326154	17330638	**USE1**	+	4484	1.315	179	343	124	2.772	1.471	0.084	1
chr16	69151911	69166493	**CHTF8**	-	14582	2.608	1166	2241	808	2.771	1.47	0.028	1
chr6	30457182	30461982	**HLA-E**	+	4800	9.043	1311	2517	909	2.769	1.469	0.034	1
chr2	24300302	24308085	**TP53I3**	-	7783	2.282	541	1039	375	2.766	1.468	0.044	1
chr12	91497231	91505542	**LUM**	-	8311	4.955	1256	2409	872	2.761	1.465	0.031	1
chr6	31826828	31830709	**NEU1**	-	3881	3.3	383	734	265	2.76	1.465	0.066	1
chr11	75110561	75116733	**RPS3**	+	6172	9.196	1736	3325	1206	2.757	1.463	0.03	1
chrX	103217199	103220563	**TMSB15B**	+	3364	1.086	112	216	78	2.755	1.462	0.093	1
chr4	152020753	152025804	**RPS3A**	+	5051	9.341	1445	2766	1004	2.753	1.461	0.03	1
chr17	31254927	31268667	**TMEM98**	+	13740	2.077	866	1657	602	2.75	1.46	0.037	1
chr9	35658286	35661500	**CCDC107**	+	3214	2.824	277	530	193	2.749	1.459	0.065	1
chr10	60094738	60130513	**UBE2D1**	+	35775	1.215	1331	2545	926	2.746	1.457	0.03	1
chr16	89284110	89295965	**ZNF778**	+	11855	1.236	443	846	308	2.741	1.455	0.057	1
chr10	45496272	45500777	**ZNF22**	+	4505	4.085	569	1086	396	2.741	1.454	0.037	1
chr3	52570620	52574586	**C3orf78**	+	3966	3.194	385	735	268	2.738	1.453	0.059	1
chr16	69373414	69377013	**NIP7**	+	3599	3.042	334	638	233	2.737	1.452	0.061	1
chr9	104152248	104160919	**MRPL50**	-	8671	1.293	344	657	240	2.736	1.452	0.056	1
chr4	6642444	6644449	**MRFAP1**	+	2005	12.218	747	1426	521	2.735	1.452	0.038	1
chr16	89627064	89633237	**RPL13**	+	6173	6.239	1171	2233	816	2.734	1.451	0.034	1
chr11	118272103	118280562	**ATP5L**	+	8459	3.616	935	1783	652	2.733	1.451	0.034	1
chr8	38120649	38126738	**PPAPDC1B**	-	6089	3.151	581	1108	405	2.733	1.451	0.048	1
chr10	1085963	1095061	**IDI1**	-	9098	3.185	879	1675	614	2.729	1.448	0.039	1
chr2	3622852	3628509	**RPS7**	+	5657	6.605	1148	2187	801	2.729	1.448	0.031	1
chr6	32861952	32871535	**LOC100294145**	+	9583	1.063	312	594	218	2.722	1.445	0.062	1
chr12	44187525	44200178	**TWF1**	-	12653	3.015	1175	2235	822	2.72	1.443	0.03	1
chr2	73956956	73964517	**TPRKB**	-	7561	1.224	285	543	199	2.719	1.443	0.061	1
chr6	42174538	42185633	**MRPS10**	-	11095	2.011	687	1305	481	2.713	1.44	0.037	1
chr1	85715636	85725355	**C1orf52**	-	9719	3.78	1134	2153	794	2.711	1.439	0.03	1
chr11	9481102	9482245	**LOC644656**	-	1143	4.234	147	280	103	2.71	1.438	0.097	1
chr4	84377117	84382929	**MRPS18C**	+	5812	2.65	481	913	337	2.707	1.437	0.039	1
chr1	28562601	28564616	**ATPIF1**	+	2015	6.627	406	770	285	2.697	1.431	0.06	1
chr6	43021766	43027242	**MRPL2**	-	5476	1.783	299	566	210	2.692	1.429	0.067	1
chr19	2269519	2273487	**OAZ1**	+	3968	10.23	1227	2322	862	2.692	1.429	0.04	1
chr8	102209265	102217960	**ZNF706**	-	8695	3.265	880	1663	619	2.687	1.426	0.033	1
chr6	111279762	111289091	**GTF3C6**	+	9329	2.066	591	1116	415	2.686	1.425	0.046	1
chr9	139619045	139622636	**SNHG7**	-	3591	6.551	716	1353	504	2.685	1.425	0.046	1
chr9	125580375	125590935	**PDCL**	-	10560	2.274	732	1383	515	2.684	1.424	0.045	1
chr10	99186026	99193198	**PGAM1**	+	7172	2.859	628	1186	442	2.682	1.423	0.046	1
chr11	22644078	22647387	**FANCF**	-	3309	1.46	146	276	102	2.682	1.424	0.108	0
chr1	46153846	46160108	**TMEM69**	+	6262	1.447	278	525	196	2.68	1.422	0.069	1
chr20	52824501	52836492	**PFDN4**	+	11991	2.276	839	1583	590	2.679	1.422	0.037	1
chr11	116649275	116658739	**ZNF259**	-	9464	2.876	828	1562	583	2.679	1.422	0.044	1
chr8	86126287	86132643	**C8orf59**	-	6356	3.058	592	1118	417	2.677	1.421	0.05	1
chr11	57425215	57429337	**CLP1**	+	4122	1.623	202	382	143	2.675	1.419	0.094	1
chr9	19115758	19127604	**PLIN2**	-	11846	2.878	1050	1980	740	2.674	1.419	0.034	1
chr7	27236498	27239725	**HOXA13**	-	3227	5.394	532	1003	375	2.674	1.419	0.052	1
chr9	33252469	33264759	**BAG1**	-	12290	2.13	795	1499	560	2.673	1.419	0.046	1
chr16	67188088	67193812	**TRADD**	-	5724	1.08	187	354	132	2.673	1.419	0.094	1
chr1	45976706	45988562	**PRDX1**	-	11856	6.78	2472	4658	1743	2.671	1.418	0.035	1
chr19	36236493	36237903	**PSENEN**	+	1410	2.448	104	196	73	2.671	1.417	0.134	0
chr5	33987090	34008220	**AMACR**	-	21130	1.948	1267	2387	894	2.67	1.417	0.033	1
chr15	55473511	55489231	**RSL24D1**	-	15720	2.877	1389	2614	981	2.663	1.413	0.035	1
chr5	149372685	149380730	**TIGD6**	-	8045	1.368	334	628	236	2.663	1.413	0.073	1
chr11	94227152	94232744	**ANKRD49**	+	5592	2.38	410	771	290	2.659	1.411	0.056	1
chr21	35275756	35288158	**ATP5O**	-	12402	2.247	853	1603	603	2.658	1.411	0.042	1
chr1	193065594	193075244	**GLRX2**	-	9650	2.101	617	1159	437	2.653	1.408	0.053	1
chr5	72794249	72801448	**BTF3**	+	7199	5.439	1215	2281	860	2.653	1.407	0.032	1
chr19	58452200	58459077	**ZNF256**	-	6877	2.039	426	800	301	2.65	1.406	0.065	1
chr11	118886421	118889057	**RPS25**	-	2636	6.475	524	983	371	2.65	1.406	0.053	1
chr17	77768175	77770915	**CBX8**	-	2740	1.685	139	261	98	2.647	1.404	0.12	0

chr17	73131343	73150775	**HN1**	-	19432	5.091	3018	5659	2138	2.646	1.404	0.045	1
chrX	102631267	102633001	**NGFRAP1**	+	1734	5.057	268	502	189	2.645	1.404	0.079	1
chr12	112277572	112280706	**C12orf47**	-	3134	1.32	126	236	89	2.644	1.403	0.116	0
chr17	48939586	48945339	**TOB1**	-	5753	7.71	1350	2529	957	2.641	1.401	0.042	1
chr12	53835432	53840427	**PRR13**	+	4995	3.386	516	967	366	2.641	1.401	0.057	1
chr11	111955538	111957522	**TIMM8B**	-	1984	1.41	85	160	60	2.639	1.4	0.134	0
chr17	73034954	73043074	**ATP5H**	-	8120	3.108	767	1434	544	2.633	1.397	0.051	1
chr12	105380097	105388505	**C12orf45**	+	8408	1.437	369	691	262	2.632	1.396	0.068	1
chr5	139712427	139726188	**HBEGF**	-	13761	1.565	654	1222	464	2.629	1.395	0.056	1
chrX	151995870	151999301	**CETN2**	-	3431	1.282	134	252	95	2.628	1.394	0.111	0
chr10	22610138	22620414	**BMI1**	+	10276	1.421	447	835	318	2.627	1.393	0.062	1
chr6	42989384	42997337	**RRP36**	+	7953	2.32	567	1059	403	2.624	1.392	0.052	1
chr6	90341942	90348474	**LYRM2**	-	6532	1.567	316	590	224	2.623	1.391	0.068	1
chr6	13615558	13621127	**NOL7**	+	5569	4.508	768	1434	546	2.623	1.391	0.047	1
chr5	150070351	150080669	**RBM22**	-	10318	3.258	1032	1923	735	2.616	1.387	0.042	1
chr7	56169265	56174187	**CHCHD2**	-	4922	3.433	515	960	367	2.615	1.387	0.061	1
chr6	71998476	72011973	**OGFRL1**	+	13497	3.016	1259	2345	897	2.614	1.386	0.036	1
chr16	81069457	81080951	**ATMIN**	+	11494	1.423	499	928	355	2.611	1.385	0.063	1
chr12	96252708	96260238	**SNRPF**	+	7530	3.634	842	1567	600	2.61	1.384	0.044	1
chr10	99092253	99094458	**FRAT2**	-	2205	1.209	80	149	57	2.6	1.379	0.166	0
chr6	26421618	26430816	**BTN2A3P**	+	9198	1.326	374	695	267	2.596	1.377	0.07	1
chr11	111957570	111966518	**SDHD**	+	8948	2.488	682	1266	488	2.595	1.375	0.053	1
chr1	109606997	109618624	**TAF13**	-	11627	4.239	1518	2814	1086	2.592	1.374	0.04	1
chr20	16710608	16722417	**SNRPB2**	+	11809	2.73	993	1841	710	2.591	1.374	0.043	1
chr9	7796490	7799799	**C9orf123**	-	3309	1.577	157	291	112	2.59	1.373	0.125	0
chr12	54378945	54384062	**HOXC10**	+	5117	1.977	308	572	220	2.589	1.372	0.084	1
chr5	7859271	7869150	**FASTKD3**	-	9879	1.733	524	970	375	2.588	1.372	0.062	1
chr6	75947390	75953644	**COX7A2**	-	6254	3.907	750	1390	537	2.588	1.372	0.05	1
chr5	132202318	132204536	**UQCRQ**	+	2218	4.082	276	511	197	2.586	1.371	0.09	1
chr5	141018868	141030986	**FCHSD1**	-	12118	2.522	933	1727	668	2.583	1.369	0.05	1
chr4	184560788	184580331	**RWDD4**	-	19543	1.08	647	1197	463	2.582	1.369	0.056	1
chr19	49990864	49995564	**RPL13A**	+	4700	16.023	2292	4242	1642	2.582	1.369	0.05	1
chr2	27293341	27294567	**OST4**	-	1226	12.834	477	883	342	2.582	1.368	0.072	1
chr5	68513572	68525985	**MRPS36**	+	12413	1.253	479	886	343	2.579	1.367	0.061	1
chr2	128603839	128615729	**POLR2D**	-	11890	2.011	732	1355	525	2.579	1.367	0.053	1
chr15	101811213	101817700	**SELS**	-	6487	5.484	1092	2019	783	2.578	1.366	0.045	1
chr6	43484776	43489246	**POLR1C**	+	4470	2.712	371	686	266	2.578	1.366	0.075	1
chr8	40010986	40012827	**C8orf4**	+	1841	8.92	507	938	363	2.578	1.367	0.058	1
chr15	45694518	45713616	**SPATA5L1**	+	19098	1.297	758	1399	544	2.569	1.361	0.054	1
chr6	26087508	26095469	**HFE**	+	7961	1.223	299	553	215	2.567	1.36	0.082	1
chr8	118532964	118552501	**MED30**	+	19537	1.424	855	1576	614	2.565	1.359	0.05	1
chr16	18794276	18801656	**RPS15A**	-	7380	8.229	1867	3442	1342	2.565	1.359	0.045	1
chr1	152004981	152009511	**S100A11**	-	4530	19.212	2655	4891	1909	2.562	1.357	0.053	1
chr6	133135707	133138703	**RPS12**	+	2996	27.713	2547	4693	1832	2.561	1.357	0.049	1
chr11	47586981	47595013	**PTPMT1**	+	8032	1.849	455	837	327	2.558	1.355	0.07	1
chr17	26655352	26662495	**IFT20**	-	7143	2.597	564	1039	406	2.558	1.355	0.067	1
chr5	162864576	162872022	**CCNG1**	+	7446	9.804	2250	4135	1621	2.551	1.351	0.046	1
chr2	241499470	241503431	**DUSP28**	+	3961	1.109	133	244	95	2.55	1.35	0.139	0
chr2	27651472	27665124	**NRBP1**	+	13652	4.177	1747	3210	1259	2.549	1.35	0.048	1
chr16	83841592	83846594	**HSBP1**	+	5002	3.463	533	979	384	2.549	1.35	0.063	1
chr2	152104727	152118389	**RBM43**	-	13662	1.357	570	1048	411	2.547	1.349	0.061	1
chrX	119384606	119392251	**ZBTB33**	+	7645	1.802	422	776	304	2.547	1.349	0.073	1
chr17	7138346	7142825	**PHF23**	-	4479	3.363	462	849	333	2.547	1.349	0.07	1
chr19	35168566	35177302	**ZNF302**	+	8736	2.49	664	1220	479	2.545	1.347	0.061	1
chr1	161719580	161726952	**DUSP12**	+	7372	3.726	848	1557	612	2.544	1.347	0.049	1
chr20	45313003	45318276	**TP53RK**	-	5273	3.811	612	1123	441	2.543	1.347	0.066	1
chr10	126085871	126107545	**OAT**	-	21674	3.146	2085	3826	1505	2.541	1.345	0.052	1
chr22	31500762	31503551	**SELM**	-	2789	7.51	632	1157	456	2.535	1.342	0.073	1
chr19	58038692	58052244	**ZNF549**	+	13552	1.368	566	1036	409	2.534	1.341	0.068	1
chr14	21927178	21945132	**RAB2B**	-	17954	1.727	951	1740	688	2.528	1.338	0.054	1
chr1	157094458	157108383	**ETV3**	-	13925	3.545	1517	2776	1098	2.528	1.338	0.048	1
chr17	7143737	7145753	**GABARAP**	-	2016	6.483	395	723	286	2.527	1.338	0.091	1
chr4	78078356	78091213	**CCNG2**	+	12857	4.09	1601	2927	1159	2.525	1.336	0.056	1
chr10	105148808	105156270	**USMG5**	-	7462	2.493	571	1043	413	2.522	1.335	0.065	1
chr10	45471708	45474330	**C10orf10**	-	2622	2.736	216	396	157	2.521	1.334	0.121	0
chr3	4344987	4358949	**SETMAR**	+	13962	1.153	493	901	357	2.519	1.333	0.072	1
chr6	34845554	34855819	**TAF11**	-	10265	2.224	704	1286	510	2.518	1.332	0.057	1
chr13	31032878	31040081	**HMGB1**	-	7203	3.516	780	1423	565	2.517	1.332	0.056	1
chr12	49578582	49582861	**TUBA1A**	-	4279	10.324	1349	2462	978	2.516	1.331	0.054	1
chr4	103998781	104021024	**BDH2**	-	22243	1.312	896	1633	650	2.51	1.328	0.056	1
chr19	57862644	57871265	**ZNF304**	+	8621	2.838	742	1352	538	2.51	1.328	0.068	1

chr12	82746082	82752584	**CCDC59**	-	6502	3.795	761	1386	552	2.509	1.327	0.057	1
chr1	39491966	39500308	**NDUFS5**	+	8342	2.3	590	1074	429	2.505	1.325	0.065	1
chr13	26786904	26796508	**RNF6**	-	9604	5.06	1493	2717	1085	2.504	1.324	0.052	1
chr7	99752042	99756302	**C7orf43**	-	4260	1.304	168	307	122	2.504	1.324	0.134	0
chr6	10695187	10709970	**PAK1IP1**	+	14783	1.43	654	1189	476	2.499	1.321	0.06	1
chr14	104378624	104387903	**C14orf2**	-	9279	2.664	755	1373	549	2.498	1.321	0.065	1
chr17	61896792	61905031	**FTSJ3**	-	8239	1.87	471	855	343	2.494	1.318	0.079	1
chr14	45366506	45376460	**C14orf28**	+	9954	1.523	464	843	338	2.49	1.316	0.079	1
chr16	19513014	19533450	**GDE1**	-	20436	1.417	889	1610	648	2.483	1.312	0.06	1
chr5	114948904	114961876	**TMED7**	-	12972	5.984	2402	4350	1752	2.482	1.311	0.053	1
chr7	43906156	43909145	**MRPS24**	-	2989	4.432	406	735	296	2.479	1.31	0.086	1
chr3	169482397	169482848	**TERC**	-	451	2.016	27	48	19	2.477	1.309	0.285	0
chr3	57541980	57547768	**PDE12**	+	5788	2.928	515	932	376	2.476	1.308	0.084	1
chr5	176728198	176730744	**RAB24**	-	2546	1.335	102	185	74	2.476	1.308	0.178	0
chr1	111991742	112004525	**ATP5F1**	+	12783	3.429	1356	2451	991	2.473	1.306	0.052	1
chr12	56510373	56511616	**RPL41**	+	1243	9.999	385	697	282	2.473	1.306	0.079	1
chr9	107509968	107522403	**NIPSNAP3A**	+	12435	3.246	1251	2261	915	2.472	1.306	0.051	1
chr2	73989324	74007284	**DUSP11**	-	17960	2.553	1417	2559	1036	2.469	1.304	0.053	1
chr11	62619459	62623360	**SNHG1**	-	3901	21.167	2519	4549	1842	2.468	1.304	0.064	1
chr1	42922172	42926086	**PPCS**	+	3914	3.804	456	824	334	2.465	1.302	0.083	1
chr18	33552587	33559250	**C18orf21**	+	6663	4.078	836	1509	612	2.464	1.301	0.062	1
chr8	74202873	74205869	**RPL7**	-	2996	8.066	745	1343	545	2.462	1.3	0.064	1
chr2	237994083	238007489	**COPS8**	+	13406	3.352	1384	2495	1014	2.46	1.298	0.056	1
chr13	114567149	114569805	**LOC100506394**	+	2656	1.433	117	210	85	2.46	1.299	0.149	0
chr9	33624222	33625532	**ANXA2P2**	+	1310	20.547	836	1506	612	2.459	1.298	0.057	1
chr7	77313167	77326662	**LOC100505854**	-	13495	1.244	519	934	380	2.456	1.296	0.073	1
chr18	77724581	77730822	**HSBP1L1**	+	6241	2.178	415	748	305	2.455	1.296	0.092	1
chr11	114271383	114279635	**RBM7**	+	8252	5.961	1524	2742	1118	2.451	1.293	0.053	1
chr1	202909960	202927700	**ADIPOR1**	-	17740	2.452	1335	2400	979	2.45	1.293	0.06	1
chr1	109633402	109639554	**TMEM167B**	+	6152	4.336	823	1479	604	2.448	1.292	0.063	1
chr6	42981840	42989036	**KLHDC3**	+	7196	3.21	704	1265	517	2.447	1.291	0.075	1
chr6	83903031	83906256	**RWDD2A**	+	3225	1.601	159	286	117	2.447	1.291	0.133	0
chr7	99746529	99751833	**C7orf59**	+	5304	2.001	323	581	237	2.445	1.29	0.107	0
chr4	17578926	17609590	**LAP3**	+	30664	1.175	1094	1964	804	2.442	1.288	0.07	1
chr17	10600926	10614875	**C17orf48**	+	13949	3.042	1330	2387	978	2.44	1.287	0.047	1
chr9	37588411	37592636	**TOMM5**	-	4225	3.179	413	741	303	2.439	1.286	0.091	1
chr14	75519927	75530736	**ACYP1**	-	10809	1.041	343	616	252	2.438	1.286	0.104	0
chr11	27515964	27528326	**LIN7C**	-	12362	3.158	1209	2168	889	2.437	1.285	0.056	1
chr7	5632435	5646287	**FSCN1**	+	13852	3.684	1546	2772	1137	2.436	1.284	0.071	1
chr5	40831429	40835387	**RPL37**	-	3958	13.93	1699	3043	1251	2.432	1.282	0.06	1
chr1	156561557	156564091	**APOA1BP**	+	2534	1.797	138	247	101	2.429	1.28	0.164	0
chrX	119004494	119005791	**RNF113A**	-	1297	1.432	56	101	41	2.429	1.281	0.215	0
chr3	196662272	196669464	**NCBP2**	-	7192	2.872	633	1133	467	2.428	1.28	0.077	1
chr11	62327072	62341460	**EEF1G**	-	14388	2.782	1234	2208	910	2.427	1.279	0.06	1
chr10	102737578	102747272	**MRPL43**	-	9694	1.072	317	566	233	2.426	1.278	0.112	0
chr17	40811265	40819024	**TUBG2**	+	7759	1.264	302	540	222	2.424	1.278	0.105	0
chr6	33378772	33384230	**PHF1**	+	5458	2.87	476	851	351	2.424	1.277	0.096	1
chr6	121756744	121770873	**GJA1**	+	14129	1.61	705	1259	520	2.423	1.277	0.067	1
chr14	59939405	59951073	**C14orf149**	-	11668	2.958	1068	1910	788	2.423	1.277	0.06	1
chr11	105878628	105892954	**KIAA1826**	-	14326	1.591	701	1253	517	2.421	1.275	0.072	1
chr5	151122382	151138210	**ATOX1**	-	15828	2.413	1166	2081	861	2.417	1.273	0.069	1
chr16	75681634	75691341	**TERF2IP**	+	9707	4.63	1388	2477	1025	2.416	1.272	0.06	1
chrX	135228860	135293518	**FHL1**	+	14000	1.851	3757	6700	2776	2.414	1.271	0.06	1
chr11	61731756	61735132	**FTH1**	-	3376	21.581	2199	3921	1625	2.413	1.271	0.08	1
chr12	27175482	27182682	**MED21**	+	7200	3.071	681	1214	503	2.412	1.27	0.073	1
chr17	26873724	26879646	**UNC119**	-	5922	1.55	279	497	206	2.412	1.27	0.126	0
chr11	94800055	94804388	**SRSF8**	+	4333	1.672	219	391	162	2.412	1.27	0.139	0
chr3	52232115	52248343	**ALAS1**	+	16228	1.849	920	1640	680	2.41	1.269	0.07	1
chr7	99090853	99097877	**ZNF394**	-	7024	2.464	529	943	391	2.409	1.269	0.09	1
chr9	116135697	116138341	**HDHD3**	-	2644	1.801	144	257	106	2.409	1.268	0.165	0
chr5	80597401	80608965	**ZCCHC9**	+	11564	2.713	969	1725	717	2.407	1.267	0.065	1
chr7	12250847	12276890	**TMEM106B**	+	26043	1.77	1430	2547	1058	2.406	1.267	0.058	1
chr1	85731459	85742587	**BCL10**	-	11128	4.723	1621	2886	1199	2.406	1.267	0.062	1
chr11	118889240	118894385	**TRAPPC4**	+	5145	2.191	345	613	255	2.398	1.262	0.111	0
chr7	12726451	12730558	**ARL4A**	+	4107	1.145	145	258	107	2.397	1.261	0.143	0
chr4	100799494	100815703	**LAMTOR3**	-	16209	2.642	1320	2344	979	2.395	1.26	0.065	1
chr15	65293849	65321977	**MTFMT**	-	28128	1.155	1002	1778	743	2.392	1.258	0.067	1
chr12	42705887	42719932	**ZCRB1**	-	14045	1.2	518	920	384	2.391	1.258	0.089	1
chr4	153457415	153460415	**DKFZP434I0714**	+	3000	2.26	207	368	154	2.39	1.257	0.137	0
chr20	2633177	2639039	**NOP56**	+	5862	6.892	1231	2182	914	2.387	1.255	0.074	1
chr6	10723147	10731362	**TMEM14C**	+	8215	2.68	675	1195	501	2.384	1.254	0.084	1

chrX	62567106	62571218	**SPIN4**	-	4112	1.812	229	406	170	2.384	1.253	0.129	0
chr6	33176285	33180499	**RING1**	+	4214	4.092	529	937	393	2.383	1.253	0.092	1
chr12	124069075	124082688	**TMED2**	+	13613	4.427	1862	3296	1384	2.381	1.252	0.065	1
chr1	3541555	3546694	**TPRG1L**	+	5139	1.505	234	415	174	2.38	1.251	0.144	0
chr1	46769379	46782447	**UQCRH**	+	13068	2.001	806	1426	599	2.378	1.249	0.074	1
chr1	151032150	151040973	**MLLT11**	+	8823	1.215	333	590	248	2.377	1.249	0.099	1
chr4	109571740	109588980	**OSTC**	+	17240	2.749	1473	2605	1096	2.376	1.249	0.061	1
chr7	27220775	27224835	**HOXA11**	-	4060	4.544	557	985	414	2.376	1.249	0.105	0
chr5	134074169	134087850	**CAMLG**	+	13681	1.537	645	1140	480	2.375	1.248	0.086	1
chr13	115079964	115092803	**CHAMP1**	+	12839	4.765	1893	3344	1409	2.373	1.247	0.065	1
chr22	24083771	24093279	**ZNF70**	-	9508	1.1	320	566	238	2.373	1.247	0.117	0
chr3	137879829	137893791	**DBR1**	-	13962	1.56	670	1184	499	2.372	1.246	0.083	1
chr14	74946642	74960084	**NPC2**	-	13442	2.47	1003	1771	746	2.372	1.246	0.088	1
chr12	6980099	6982521	**SPSB2**	-	2422	1.495	110	195	82	2.372	1.246	0.178	0
chr1	146649429	146651528	**PDIA3P**	+	2099	4.523	293	518	218	2.372	1.246	0.116	0
chr13	92000073	92006829	**MIR17HG**	+	6756	1.825	380	672	283	2.37	1.245	0.102	0
chr7	42956461	42971805	**PSMA2**	-	15344	2.781	1330	2347	991	2.369	1.244	0.061	1
chr1	45241245	45244412	**RPS8**	+	3167	9.046	875	1544	652	2.368	1.243	0.083	1
chr7	45022626	45026259	**SNHG15**	-	3633	3.831	426	752	318	2.364	1.241	0.106	0
chr17	49242795	49249105	**NME2**	+	6310	3.795	732	1289	546	2.362	1.24	0.088	1
chr2	68385004	68403094	**PNO1**	+	18090	1.536	856	1509	639	2.361	1.239	0.076	1
chr1	153631144	153634326	**SNAPIN**	+	3182	1.543	151	266	112	2.361	1.239	0.154	0
chr1	45266035	45271667	**PLK3**	+	5632	7.104	1211	2133	904	2.36	1.239	0.086	1
chr5	75911306	75919240	**F2RL2**	-	7934	3.309	802	1412	598	2.359	1.238	0.086	1
chr19	50354415	50363999	**PTOV1**	+	9584	1.217	356	627	266	2.358	1.237	0.12	0
chr15	69745158	69747884	**RPLP1**	+	2726	20.042	1669	2938	1246	2.358	1.237	0.077	1
chr11	94695786	94706776	**CWC15**	-	10990	3.328	1135	1998	847	2.357	1.237	0.066	1
chr17	72766685	72772470	**NAT9**	-	5785	3.475	612	1077	457	2.357	1.237	0.097	1
chr11	68658745	68671303	**MRPL21**	-	12558	1.658	638	1122	476	2.356	1.236	0.092	1
chr17	49231023	49239450	**NME1**	+	8531	2.088	542	954	405	2.353	1.234	0.092	1
chr12	32899477	32908887	**YARS2**	-	9410	1.983	574	1010	429	2.351	1.234	0.092	1
chr3	132373289	132396944	**UBA5**	+	23655	2.121	1548	2718	1157	2.348	1.231	0.072	1
chr10	75002581	75007025	**DNAJC9**	-	4444	2.771	378	664	283	2.348	1.231	0.113	0
chr7	99070514	99085217	**ZNF789**	+	14703	1.189	535	940	400	2.345	1.229	0.099	1
chr12	8071823	8088892	**SLC2A3**	-	17069	2.426	1252	2196	937	2.344	1.229	0.091	1
chr1	54497348	54519111	**TMEM59**	-	21763	2.14	1450	2543	1085	2.343	1.228	0.065	1
chrX	70280787	70288231	**SNX12**	-	7444	1.268	290	508	217	2.342	1.227	0.13	0
chr16	58147496	58163296	**C16orf80**	-	15800	1.756	848	1487	635	2.341	1.227	0.088	1
chr19	13049413	13055304	**CALR**	+	5891	23.458	4248	7448	3181	2.341	1.227	0.091	1
chr4	90033967	90036052	**TIGD2**	+	2085	1.866	120	210	90	2.341	1.227	0.172	0
chrX	51486480	51489326	**GSPT2**	+	2846	1.497	130	228	97	2.339	1.226	0.182	0
chr12	46119502	46121704	**LOC400027**	-	2202	2.887	193	339	145	2.338	1.225	0.159	0
chr6	52842745	52860178	**GSTA4**	-	17433	1.294	696	1220	522	2.337	1.225	0.084	1
chr1	7831328	7841492	**VAMP3**	+	10164	4.801	1499	2626	1123	2.337	1.225	0.077	1
chr3	51422691	51426828	**MANF**	+	4137	2.412	308	539	230	2.337	1.225	0.122	0
chr6	44214848	44221614	**HSP90AB1**	+	6766	14.218	2951	5169	2212	2.336	1.224	0.084	1
chr11	4406126	4414926	**TRIM21**	-	8800	1.612	432	757	324	2.335	1.223	0.118	0
chr6	160199529	160210735	**TCP1**	-	11206	4.166	1434	2509	1076	2.332	1.221	0.077	1
chr19	50162825	50169132	**IRF3**	-	6307	1.102	209	367	157	2.332	1.222	0.169	0
chr3	149456256	149470286	**COMMD2**	-	14030	3.346	1457	2549	1094	2.33	1.22	0.07	1
chr7	19735084	19748660	**TWISTNB**	-	13576	1.897	800	1399	600	2.33	1.22	0.077	1
chrX	77385244	77395179	**TAF9B**	-	9935	1.006	312	546	234	2.33	1.22	0.109	0
chr8	17913924	17942507	**ASAH1**	-	28583	1.11	963	1683	723	2.326	1.218	0.094	1
chr5	52393894	52405598	**MOCS2**	-	11704	1.288	464	812	349	2.326	1.218	0.105	0
chr15	43809805	43823818	**MAP1A**	+	14013	3.592	1554	2715	1167	2.325	1.217	0.074	1
chr16	66965957	66968320	**FAM96B**	-	2363	2.713	195	341	147	2.322	1.215	0.163	0
chr9	130922538	130926207	**C9orf16**	+	3669	1.038	114	198	85	2.321	1.214	0.22	0
chr7	117824085	117844093	**NAA38**	+	20008	2.24	1388	2422	1044	2.319	1.214	0.073	1
chr1	20808883	20812728	**CAMK2N1**	-	3845	6.373	748	1304	563	2.316	1.211	0.097	1
chr12	120875903	120878529	**COX6A1**	+	2626	2.262	180	314	136	2.311	1.208	0.174	0
chr20	4666796	4682234	**PRNP**	+	15438	6.244	2958	5148	2228	2.31	1.208	0.09	1
chr1	211748380	211752099	**SLC30A1**	-	3719	6.949	781	1359	588	2.31	1.208	0.108	0
chr12	121124948	121139667	**MLEC**	+	14719	3.546	1602	2787	1207	2.308	1.207	0.083	1
chr1	112298189	112310199	**DDX20**	+	12010	1.666	618	1075	466	2.306	1.206	0.094	1
chr17	6546632	6554954	**MED31**	-	8322	3.587	912	1586	688	2.306	1.206	0.093	1
chr12	56735381	56754037	**STAT2**	-	18656	3.8	2160	3754	1629	2.305	1.205	0.093	1
chr17	74553845	74561430	**LOC100507246**	+	7585	5.226	1217	2115	917	2.305	1.205	0.084	1
chr21	27096790	27107965	**ATP5J**	-	11175	2.076	716	1244	540	2.304	1.204	0.092	1
chr3	14220227	14239869	**LSM3**	+	19642	1.141	690	1199	520	2.302	1.203	0.094	1
chr11	66610987	66614003	**RCE1**	+	3121	2.274	208	362	157	2.302	1.203	0.166	0
chr1	46016454	46035723	**AKR1A1**	+	19269	1.079	630	1095	475	2.301	1.203	0.114	0

chr22	39925097	39928860	**RPS19BP1**	-	3763	3.029	346	601	262	2.293	1.197	0.141	0
chr12	50505899	50514234	**C12orf62**	+	8335	2.284	577	1000	436	2.291	1.196	0.121	0
chr21	30428647	30446010	**CCT8**	-	17363	2.976	1607	2783	1215	2.29	1.195	0.075	1
chr10	64893006	64914786	**NRBF2**	+	21780	1.791	1212	2096	917	2.285	1.192	0.078	1
chr19	10196805	10203928	**C19orf66**	+	7123	1.463	315	546	239	2.285	1.192	0.153	0
chr22	29190547	29196560	**XBP1**	-	6013	8.33	1546	2674	1170	2.285	1.192	0.083	1
chr9	88879462	88897490	**ISCA1**	-	18028	2.208	1224	2116	927	2.283	1.191	0.088	1
chr12	120634502	120639014	**RPLP0**	-	4512	19.379	2684	4640	2032	2.283	1.191	0.093	1
chr17	6917852	6918055	**RNASEK-C17ORI**	+	734	1.822	10	18	8	2.283	1.191	0.435	0
chr4	79839093	79860582	**PAQR3**	-	21489	1.509	996	1721	754	2.282	1.19	0.092	1
chr15	34376223	34394053	**C15orf24**	-	17830	1.743	971	1679	735	2.282	1.19	0.076	1
chr19	37001588	37019248	**ZNF260**	-	17660	1.231	672	1161	508	2.282	1.19	0.097	1
chr4	57829515	57843826	**NOA1**	-	14311	1.355	595	1028	450	2.28	1.189	0.109	0
chr5	140027383	140042065	**IK**	+	14682	1.693	765	1322	580	2.279	1.188	0.096	1
chr4	2743386	2758103	**TNIP2**	-	14717	1.445	647	1117	491	2.276	1.187	0.115	0
chr2	232319458	232329205	**NCL**	-	9747	16.165	4861	8388	3685	2.276	1.186	0.106	0
chr1	161009040	161015757	**USF1**	-	6717	1.896	388	669	294	2.276	1.187	0.138	0
chrX	68380580	68385365	**PJA1**	-	4785	1.216	177	307	134	2.276	1.187	0.179	0
chr2	190634992	190649097	**ORMDL1**	-	14105	3.017	1321	2280	1002	2.275	1.186	0.079	1
chr19	44598481	44612479	**ZNF224**	+	13998	1.654	711	1226	539	2.275	1.186	0.102	0
chr1	174968570	174981163	**CACYBP**	+	12593	1.898	742	1278	563	2.269	1.182	0.092	1
chr17	8280833	8286565	**RPL26**	-	5732	8.118	1439	2479	1092	2.269	1.182	0.084	1
chr3	180319917	180328918	**TTC14**	+	9001	4.804	1338	2304	1016	2.267	1.181	0.084	1
chr18	3247527	3256234	**MYL12A**	+	8707	13.751	3723	6409	2828	2.266	1.18	0.092	1
chr6	33168602	33172214	**SLC39A7**	+	3612	7.313	810	1394	615	2.265	1.179	0.101	0
chr11	120195837	120204388	**TMEM136**	+	8551	1.777	464	799	353	2.263	1.178	0.13	0
chr7	42971938	42977453	**MRPL32**	+	5515	2.227	379	652	288	2.263	1.178	0.13	0
chr11	11373320	11374904	**CSNK2A1P**	-	1584	1.114	54	93	41	2.263	1.178	0.248	0
chr5	177576464	177580961	**NHP2**	-	4497	1.455	200	343	152	2.262	1.178	0.177	0
chr2	201676268	201688569	**BZW1**	+	12301	5.737	2201	3781	1674	2.259	1.175	0.082	1
chr4	148538538	148556672	**TMEM184C**	+	18134	1.586	894	1536	680	2.258	1.175	0.088	1
chr1	155978838	155990758	**SSR2**	-	11920	3.012	1105	1898	841	2.256	1.174	0.094	1
chr2	71357443	71377232	**MPHOSPH10**	+	19789	2.136	1311	2251	998	2.255	1.173	0.084	1
chr11	5710816	5732093	**TRIM22**	+	21277	3.334	2210	3793	1682	2.254	1.173	0.083	1
chr7	75931874	75933614	**HSPB1**	+	1740	6.86	358	614	273	2.25	1.17	0.168	0
chr2	200820039	200828847	**C2orf47**	+	8808	2.843	775	1328	590	2.249	1.169	0.097	1
chr14	23938897	23947402	**NGDN**	+	8505	2.926	769	1319	586	2.249	1.17	0.098	1
chr2	217363519	217366188	**RPL37A**	+	2669	17.507	1446	2477	1102	2.247	1.168	0.088	1
chr1	43629844	43638241	**EBNA1BP2**	-	8397	3.531	913	1563	697	2.242	1.165	0.098	1
chrX	100663120	100669128	**HNRNPH2**	+	6008	3.49	644	1102	491	2.242	1.165	0.113	0
chr13	52586522	52598826	**ALG11**	+	17258	2.821	1072	1833	818	2.241	1.164	0.094	1
chr1	24018268	24022915	**RPL11**	+	4647	13.589	1951	3338	1489	2.241	1.164	0.092	1
chr6	34385230	34393902	**RPS10**	-	8672	4.53	1216	2079	928	2.239	1.163	0.091	1
chr19	50058967	50083803	**NOSIP**	-	24836	1.092	834	1426	637	2.237	1.161	0.104	0
chrX	54834170	54842445	**MAGED2**	+	8275	2.727	690	1180	527	2.236	1.161	0.117	0
chr8	42691816	42698474	**THAP1**	-	6658	1.09	225	384	172	2.233	1.159	0.159	0
chr17	7788122	7816075	**CHD3**	+	27953	1.712	1478	2522	1130	2.232	1.158	0.094	1
chr3	88198892	88207115	**C3orf38**	+	8223	2.704	692	1181	530	2.23	1.157	0.099	1
chr2	38970740	38978636	**SRSF7**	-	7896	15.205	3713	6334	2840	2.23	1.157	0.105	0
chr12	100594573	100618201	**ACTR6**	+	23628	1.212	889	1516	680	2.229	1.156	0.095	1
chr10	23728197	23731310	**OTUD1**	+	3113	1.688	160	272	122	2.228	1.156	0.204	0
chr14	74960449	74962271	**ISCA2**	+	1822	3.117	172	294	132	2.228	1.156	0.205	0
chr19	8386383	8387280	**RPS28**	+	897	4.012	109	186	83	2.228	1.156	0.238	0
chr15	40845297	40857252	**C15orf57**	-	11955	1.212	448	764	343	2.227	1.155	0.129	0
chr6	19837616	19840915	**ID4**	+	3299	3.896	393	670	301	2.227	1.155	0.145	0
chr19	55964345	55973049	**ISOC2**	-	8704	1.561	413	704	316	2.226	1.155	0.15	0
chr15	78832746	78841563	**PSMA4**	+	8817	3.31	904	1541	692	2.225	1.154	0.097	1
chr6	33540322	33548070	**BAK1**	-	7748	1.823	428	729	328	2.223	1.153	0.152	0
chr22	35777059	35790207	**HMOX1**	+	13148	2.314	916	1560	702	2.221	1.151	0.131	0
chr1	151138497	151142773	**SCNM1**	+	4276	2.391	315	536	241	2.22	1.15	0.151	0
chr7	66452689	66460588	**SBDS**	-	7899	2.267	555	944	425	2.219	1.15	0.117	0
chr8	125487007	125500859	**RNF139**	+	13852	2.893	1242	2112	952	2.218	1.149	0.094	1
chr12	112451151	112461024	**ERP29**	+	9873	2.181	660	1121	506	2.214	1.147	0.123	0
chr7	77423044	77427747	**TMEM60**	-	4703	2.803	404	687	310	2.214	1.146	0.145	0
chr19	39390339	39399534	**NFKBIB**	+	9195	1.115	311	529	239	2.211	1.145	0.172	0
chrX	107327434	107334874	**PSMD10**	-	7440	1.615	373	634	287	2.211	1.145	0.135	0
chr3	48481685	48485537	**CCDC72**	+	3852	3.693	436	740	334	2.21	1.144	0.143	0
chr19	58082933	58090243	**ZNF416**	-	7310	2.054	460	780	353	2.209	1.144	0.141	0
chr3	44690232	44702283	**ZNF35**	+	12051	2.189	814	1380	625	2.207	1.142	0.109	0
chr7	91502020	91510016	**MTERF**	-	7996	3.404	845	1433	649	2.206	1.141	0.101	0
chr8	141521396	141527252	**CHRAC1**	+	5856	2.121	379	643	291	2.205	1.141	0.156	0

chr11	126071988	126081587	**RPUSD4**	-	9599	1.734	514	871	395	2.204	1.14	0.129	0
chr22	42481529	42486888	**NDUFA6**	-	5359	2.801	464	786	356	2.204	1.14	0.134	0
chr9	34646634	34650573	**GALT**	+	3939	2.156	259	439	199	2.204	1.14	0.181	0
chr17	79213110	79215098	**C17orf89**	+	1988	3.457	208	353	160	2.2	1.138	0.203	0
chrX	47001614	47004609	**NDUFB11**	-	2995	1.567	144	244	111	2.199	1.137	0.206	0
chr11	62360674	62369312	**MTA2**	-	8638	3.55	942	1593	725	2.198	1.136	0.113	0
chr16	2012061	2014827	**RPS2**	-	2766	8.143	688	1164	530	2.198	1.136	0.131	0
chr8	144915754	144923146	**NRBP2**	-	7392	1.183	267	452	205	2.196	1.135	0.18	0
chr2	220036618	220041702	**CNPPD1**	-	5084	1.958	302	512	233	2.195	1.134	0.18	0
chr6	31926580	31937532	**SKIV2L**	+	10952	1.43	477	806	367	2.193	1.133	0.15	0
chr11	6632047	6633475	**TAF10**	-	1428	3.621	156	264	120	2.192	1.132	0.229	0
chr13	48807273	48836232	**ITM2B**	+	28959	3.124	2818	4755	2172	2.189	1.131	0.1	1
chr3	185207388	185216845	**TMEM41A**	-	9457	1.723	500	843	385	2.189	1.13	0.141	0
chr2	10580507	10588453	**ODC1**	-	7946	5.14	1260	2127	971	2.189	1.13	0.105	0
chr1	231154703	231175995	**FAM89A**	-	21292	1.006	657	1108	507	2.185	1.127	0.131	0
chrX	135579237	135594503	**HTATSF1**	+	15266	1.413	671	1132	518	2.185	1.128	0.113	0
chrX	118602362	118605359	**SLC25A5**	+	2997	2.677	245	413	189	2.184	1.127	0.188	0
chr11	47600561	47606115	**NDUFS3**	+	5554	2.657	454	765	350	2.183	1.127	0.143	0
chr9	139756570	139760738	**EDF1**	-	4168	2.757	351	591	271	2.183	1.126	0.168	0
chr2	220071537	220074370	**ZFAND2B**	+	2833	1.246	107	180	82	2.181	1.125	0.248	0
chr2	63815742	63834330	**MDH1**	+	18588	2.308	1323	2227	1021	2.18	1.125	0.107	0
chr11	65190268	65194003	**NEAT1**	+	3735	135.366	15732	26484	12148	2.18	1.124	0.199	0
chr3	101399933	101405563	**RPL24**	-	5630	6.336	1111	1869	858	2.178	1.123	0.099	1
chr20	23331372	23335408	**NXT1**	+	4036	1.6	197	331	152	2.178	1.123	0.206	0
chr5	96496570	96519005	**RIOK2**	-	22435	1.933	1356	2282	1048	2.177	1.123	0.094	1
chr22	30163357	30166402	**UQCR10**	+	3045	2.979	278	468	215	2.177	1.123	0.178	0
chr6	31654725	31671137	**ABHD16A**	-	16412	1.373	688	1157	531	2.176	1.122	0.137	0
chr5	34905365	34915780	**RAD1**	-	10415	3.609	1162	1954	898	2.176	1.122	0.107	0
chr17	79648223	79650954	**ARL16**	-	2731	2.223	185	311	143	2.175	1.121	0.214	0
chr2	27587218	27593324	**EIF2B4**	-	6106	1.945	364	613	282	2.174	1.12	0.161	0
chr1	145575987	145586546	**PIAS3**	+	10559	4.035	1306	2194	1009	2.173	1.12	0.116	0
chr2	232573234	232578250	**PTMA**	+	5016	6.401	974	1637	753	2.173	1.12	0.135	0
chr5	177019212	177023099	**TMED9**	+	3887	5.216	622	1046	481	2.173	1.119	0.135	0
chr4	174252526	174255595	**HMGB2**	-	3069	15.467	1474	2477	1140	2.172	1.119	0.1	0
chr6	33257377	33258711	**PFDN6**	+	1334	2.183	88	149	68	2.172	1.119	0.262	0
chr7	141438175	141450257	**SSBP1**	+	12082	3.44	1287	2161	995	2.17	1.118	0.106	0
chr5	133291197	133304406	**C5orf15**	-	13209	2.762	1136	1906	879	2.169	1.117	0.101	0
chr19	36605887	36616849	**TBCB**	+	10962	1.958	657	1103	508	2.169	1.117	0.137	0
chr11	9002122	9025596	**NRIP3**	-	23474	1.2	875	1469	677	2.168	1.117	0.109	0
chr19	33864574	33873592	**CEBPG**	+	9018	2.975	828	1390	641	2.167	1.116	0.12	0
chr1	94352589	94375012	**GCLM**	-	22423	2.107	1477	2476	1144	2.164	1.114	0.097	1
chr17	38077295	38083884	**ORMDL3**	-	6589	1.118	224	376	174	2.164	1.114	0.205	0
chr5	112196884	112228776	**SRP19**	+	31892	1.78	1752	2935	1358	2.162	1.112	0.112	0
chr5	158690088	158713048	**UBLCP1**	+	22960	1.821	1304	2186	1011	2.162	1.113	0.099	1
chr3	39448203	39454032	**RPSA**	+	5829	12.281	2212	3706	1714	2.161	1.112	0.113	0
chr11	77327195	77348851	**CLNS1A**	-	21656	1.69	1138	1905	882	2.159	1.11	0.105	0
chr4	52886860	52904485	**SGCB**	-	17625	3.49	1918	3212	1487	2.159	1.111	0.101	0
chr16	68334517	68344868	**SLC7A6OS**	-	10351	2.162	690	1156	535	2.159	1.11	0.131	0
chr6	83072922	83077133	**TPBG**	+	4211	6.311	808	1353	626	2.159	1.111	0.139	0
chr15	35270541	35280497	**ZNF770**	-	9956	2.781	858	1437	666	2.158	1.11	0.115	0
chr10	99437180	99447015	**AVPI1**	-	9835	2.54	759	1269	588	2.156	1.108	0.144	0
chr12	110929329	110939916	**VPS29**	-	10587	2.43	800	1338	621	2.155	1.108	0.115	0
chr4	7061779	7069800	**GRPEL1**	-	8021	2.428	598	1000	464	2.155	1.107	0.141	0
chr12	4430358	4469190	**C12orf5**	+	38832	2.024	2426	4053	1883	2.152	1.106	0.119	0
chr6	26634610	26659980	**ZNF322**	-	25370	1.621	1273	2127	988	2.152	1.105	0.111	0
chr14	24630421	24635774	**IRF9**	+	5353	4.349	708	1183	549	2.152	1.106	0.146	0
chr14	20757300	20774153	**TTC5**	-	16853	1.022	528	882	410	2.15	1.104	0.15	0
chr11	61556601	61560085	**C11orf10**	-	3484	1.559	167	278	129	2.147	1.102	0.218	0
chr17	46125685	46138907	**NFE2L1**	+	13222	3.743	1523	2541	1183	2.146	1.102	0.12	0
chr12	54058943	54070512	**ATP5G2**	-	11569	3.591	1285	2144	999	2.145	1.101	0.113	0
chr15	85186011	85197521	**WDR73**	-	11510	1.415	501	836	389	2.145	1.101	0.15	0
chr1	33402049	33430286	**RNF19B**	-	28237	1.26	1094	1825	851	2.144	1.101	0.124	0
chr22	41829491	41843027	**TOB2**	-	13536	5.078	2086	3477	1623	2.142	1.099	0.139	0
chr22	51017386	51021428	**CHKB**	-	4042	1.01	124	207	96	2.142	1.099	0.252	0
chr11	17095938	17099220	**RPS13**	-	3282	10.727	1085	1808	844	2.142	1.099	0.121	0
chr15	78441718	78462884	**IDH3A**	+	21166	1.243	805	1342	627	2.14	1.098	0.137	0
chr2	122484520	122494503	**MKI67IP**	-	9983	3.536	1090	1814	848	2.139	1.097	0.121	0
chr4	184415889	184425668	**LOC389247**	-	9779	1.087	333	555	259	2.139	1.097	0.149	0
chr11	818900	825571	**PNPLA2**	+	6671	3.165	638	1062	496	2.139	1.097	0.164	0
chr15	75212616	75230495	**COX5A**	-	17879	1.185	652	1086	508	2.138	1.096	0.139	0
chr15	90627211	90645708	**IDH2**	-	18497	1.433	817	1360	636	2.137	1.096	0.131	0

chr10	85899184	85913311	**GHITM**	+	14127	3.764	1649	2743	1284	2.136	1.095	0.113	0
chr12	118573869	118583390	**PEBP1**	+	9521	3.323	969	1611	754	2.135	1.094	0.135	0
chr13	95226307	95248511	**TGDS**	-	22204	1.769	1213	2016	945	2.132	1.093	0.121	0
chr14	53241910	53258386	**GNPNAT1**	-	16476	2.142	1098	1826	856	2.132	1.093	0.113	0
chr1	35447126	35450948	**ZMYM6NB**	-	3822	1.94	227	378	177	2.131	1.091	0.206	0
chr1	24128366	24151949	**HMGCL**	-	23583	1.001	723	1201	564	2.129	1.09	0.143	0
chr11	114310107	114321000	**REXO2**	+	10893	3.613	1228	2039	958	2.129	1.09	0.109	0
chr4	109541721	109551639	**RPL34**	+	9918	6.963	2135	3545	1665	2.129	1.09	0.121	0
chr17	73937588	73975515	**ACOX1**	-	37927	1.82	2144	3559	1672	2.128	1.09	0.116	0
chr3	32522803	32544403	**CMTM6**	-	21600	2.595	1738	2886	1356	2.128	1.089	0.116	0
chr5	141000442	141016423	**HDAC3**	-	15981	2.151	1062	1763	828	2.127	1.089	0.124	0
chr10	75561668	75571589	**NDST2**	-	9921	1.484	449	745	350	2.126	1.088	0.176	0
chr14	39644386	39652422	**PNN**	+	8036	8.835	2211	3668	1725	2.126	1.088	0.114	0
chr19	58193356	58201169	**ZNF551**	+	7813	2.556	613	1018	479	2.125	1.088	0.149	0
chr3	170606203	170626426	**EIF5A2**	-	20223	1.991	1250	2073	975	2.124	1.087	0.116	0
chr1	115247084	115259515	**NRAS**	-	12431	5.969	2309	3829	1803	2.124	1.087	0.116	0
chr11	111944967	111955874	**C11orf57**	+	10907	2.071	701	1162	547	2.123	1.086	0.134	0
chr3	122130699	122134882	**WDR5B**	-	4183	1.081	140	233	109	2.121	1.085	0.222	0
chr14	20811229	20811570	**RPPH1**	-	341	218.652	2327	3855	1817	2.121	1.085	0.114	0
chr9	136215068	136218280	**RPL7A**	+	3212	9.092	900	1490	703	2.118	1.083	0.134	0
chr4	88812994	88815167	**HSP90AB3P**	+	2173	2.518	170	282	133	2.118	1.083	0.209	0
chr2	20232410	20251789	**LAPTM4A**	-	19379	4.194	2522	4175	1972	2.117	1.082	0.123	0
chr14	77564577	77583630	**KIAA1737**	+	19053	1.145	669	1108	523	2.117	1.082	0.148	0
chr6	28234787	28245980	**ZNF187**	+	11193	2.302	801	1325	626	2.117	1.082	0.128	0
chr20	20015011	20036690	**CRNKL1**	-	21679	1.579	1065	1762	832	2.116	1.081	0.119	0
chr20	33292147	33301237	**TP53INP2**	+	9090	3.825	1057	1749	826	2.116	1.081	0.145	0
chr11	64851693	64855874	**ZFPL1**	+	4181	3.284	420	695	328	2.116	1.081	0.178	0
chr19	13033283	13044558	**FARSA**	-	11275	1.364	469	776	367	2.114	1.08	0.177	0
chr17	27071022	27077976	**TRAF4**	+	6954	5.49	1159	1916	906	2.114	1.08	0.146	0
chrX	48755194	48760422	**PQBP1**	+	5228	1.412	227	376	178	2.113	1.079	0.206	0
chr7	72299951	72307978	**SBDSP1**	+	8027	1.443	358	591	280	2.112	1.078	0.176	0
chrX	101859085	101860581	**ARMCX5-GPRAS**	+	5948	1.597	73	121	57	2.112	1.078	0.281	0
chr9	101978706	101984246	**ALG2**	-	5540	4.917	836	1382	654	2.112	1.078	0.141	0
chr21	37442284	37445462	**CBR1**	+	3178	3.928	381	631	298	2.112	1.079	0.187	0
chr1	153516094	153518282	**S100A4**	-	2188	25.318	1713	2831	1340	2.112	1.079	0.124	0
chr20	42086503	42092244	**SRSF6**	+	5741	20.183	3578	5908	2801	2.109	1.076	0.139	0
chr16	75480922	75498584	**TMEM170A**	-	17662	1.523	828	1365	648	2.104	1.073	0.141	0
chr17	7387697	7417935	**POLR2A**	+	30238	7.034	6535	10775	5122	2.103	1.073	0.176	0
chr19	40946747	40950282	**SERTAD3**	-	3535	7.621	826	1362	648	2.103	1.072	0.145	0
chrX	100805513	100809675	**ARMCX1**	+	4162	2.077	267	440	209	2.102	1.072	0.195	0
chr11	62443971	62446527	**UBXN1**	-	2556	4.073	317	524	249	2.102	1.072	0.204	0
chr11	10533224	10562774	**RNF141**	-	29550	1.666	1536	2531	1204	2.101	1.071	0.116	0
chr13	50571142	50592603	**TRIM13**	+	21461	2.445	1642	2706	1288	2.1	1.07	0.113	0
chr9	101984569	101992901	**SEC61B**	+	8332	4.606	1194	1966	937	2.097	1.069	0.121	0
chrX	77154960	77160881	**COX7B**	+	5921	2.356	433	712	339	2.097	1.068	0.163	0
chr11	67374322	67380012	**NDUFV1**	+	5690	2.226	387	638	304	2.097	1.068	0.19	0
chr1	16340522	16345285	**HSPB7**	-	4763	1.319	192	317	151	2.096	1.067	0.228	0
chr19	39876269	39881679	**PAF1**	-	5410	2.379	394	649	310	2.094	1.066	0.187	0
chr17	7761063	7765600	**CYB5D1**	+	4537	1.114	154	253	121	2.094	1.067	0.249	0
chr10	103911932	103923627	**NOLC1**	+	11695	2.253	814	1337	639	2.093	1.065	0.141	0
chr5	95149552	95158577	**GLRX**	-	9025	4.834	1335	2194	1049	2.092	1.065	0.142	0
chrX	119005733	119010629	**NDUFA1**	+	4896	2.111	319	525	251	2.092	1.065	0.188	0
chr2	54014067	54045956	**ERLEC1**	+	31889	1.9	1884	3094	1480	2.09	1.063	0.125	0
chr12	110872705	110888158	**ARPC3**	-	15453	2.936	1423	2338	1118	2.09	1.064	0.113	0
chr11	85358962	85367597	**TMEM126A**	+	8635	1.934	520	855	409	2.09	1.064	0.149	0
chr6	139349818	139364439	**C6orf115**	+	14621	1.788	799	1313	628	2.089	1.063	0.155	0
chr15	102182048	102192594	**TM2D3**	-	10546	3.699	1202	1974	945	2.089	1.063	0.137	0
chr10	53455245	53459355	**CSTF2T**	-	4110	2.73	346	569	272	2.089	1.063	0.187	0
chr11	74699949	74718743	**NEU3**	+	18794	1.468	847	1392	666	2.088	1.062	0.147	0
chr9	125670334	125675609	**ZBTB6**	-	5275	3.291	541	888	425	2.088	1.062	0.148	0
chr6	26597170	26600277	**ABT1**	+	3107	3.547	338	555	266	2.086	1.061	0.196	0
chr11	89933596	89956532	**CHORDC1**	-	22936	3.161	2243	3679	1764	2.085	1.06	0.135	0
chr15	79603490	79615189	**TMED3**	+	11699	2.702	966	1585	760	2.085	1.06	0.15	0
chr2	70121074	70132368	**SNRNP27**	+	11294	2.755	963	1580	758	2.085	1.06	0.136	0
chr10	112052797	112064707	**SMNDC1**	-	11910	4.511	1681	2755	1323	2.082	1.058	0.119	0
chr1	145438461	145442628	**TXNIP**	+	4167	12.721	1633	2676	1285	2.082	1.058	0.137	0
chr3	149682690	149688741	**PFN2**	-	6051	8.862	1670	2736	1315	2.081	1.057	0.125	0
chr12	112842993	112847443	**RPL6**	-	4450	6.987	967	1585	761	2.081	1.057	0.133	0
chr20	57603732	57607422	**ATP5E**	-	3690	6.392	731	1198	575	2.081	1.057	0.144	0
chr17	4699456	4701790	**PSMB6**	+	2334	3.747	268	439	211	2.08	1.057	0.215	0
chr19	5690271	5691678	**RPL36**	+	1407	6.616	284	466	224	2.08	1.056	0.216	0

chr22	25844053	25857645	**CRYBB2P1**	+	13592	1.851	769	1259	605	2.079	1.056	0.161	0
chr6	64281919	64293489	**PTP4A1**	+	11570	8.414	2993	4901	2357	2.079	1.056	0.148	0
chr19	57980953	57988938	**ZNF772**	-	7985	1.275	314	515	248	2.078	1.055	0.194	0
chr12	8234806	8250373	**NECAP1**	+	15567	2.27	1089	1783	858	2.077	1.055	0.14	0
chr1	52870218	52883992	**PRPF38A**	+	13774	2.262	968	1584	762	2.077	1.054	0.135	0
chr2	3592690	3605940	**RNASEH1**	-	13250	2.627	1081	1770	852	2.077	1.055	0.133	0
chr10	124753196	124768311	**IKZF5**	-	15115	2.211	1042	1705	821	2.076	1.054	0.13	0
chr13	45911303	45915297	**TPT1**	-	3994	16.455	2032	3323	1602	2.075	1.053	0.137	0
chr1	113454469	113498975	**SLC16A1**	-	44506	1.365	1877	3066	1480	2.071	1.05	0.138	0
chr12	51632507	51640501	**DAZAP2**	+	7994	6.042	1481	2419	1168	2.07	1.05	0.146	0
chr1	161195832	161200407	**TOMM40L**	+	4575	1.383	192	314	152	2.069	1.049	0.252	0
chr15	66161796	66184329	**RAB11A**	+	22533	2.924	2046	3338	1615	2.067	1.048	0.135	0
chr15	75192327	75199462	**C15orf17**	-	7135	2.403	523	854	413	2.067	1.047	0.188	0
chr10	77157602	77161513	**ZNF503**	-	3911	1.484	175	285	138	2.067	1.048	0.277	0
chr7	99613218	99635403	**ZKSCAN1**	+	22185	2.918	2013	3284	1589	2.066	1.047	0.134	0
chr6	24650204	24667115	**TDP2**	-	16911	2.495	1303	2126	1029	2.065	1.046	0.139	0
chr1	153634513	153643479	**ILF2**	-	8966	4.365	1215	1982	959	2.065	1.046	0.135	0
chr19	50364459	50370822	**PNKP**	-	6363	1.468	284	463	224	2.065	1.046	0.231	0
chr17	56078279	56084707	**SRSF1**	-	6428	12.749	2550	4158	2014	2.064	1.046	0.137	0
chr19	39881962	39891203	**MED29**	+	9241	1.656	468	764	370	2.063	1.045	0.19	0
chr2	3501689	3523350	**ADI1**	-	21661	2.433	1632	2660	1290	2.062	1.044	0.137	0
chr9	116029289	116037869	**CDC26**	-	8580	1.193	314	512	248	2.062	1.044	0.209	0
chr10	97803158	97820625	**CCNJ**	+	17467	1.291	697	1135	551	2.058	1.041	0.156	0
chrX	24001832	24045303	**KLHL15**	-	43471	1.209	1630	2652	1289	2.057	1.04	0.137	0
chr9	125680377	125693779	**ZBTB26**	-	13402	1.524	635	1034	502	2.057	1.041	0.153	0
chr4	47452810	47465676	**COMMD8**	-	12866	1.808	723	1176	572	2.056	1.04	0.149	0
chr19	36119979	36128587	**RBM42**	+	8608	2.084	547	891	433	2.056	1.04	0.188	0
chr17	61904769	61909387	**PSMC5**	+	4618	6.326	903	1470	715	2.056	1.04	0.147	0
chr17	5377612	5389494	**DERL2**	-	11882	3.278	1209	1966	957	2.055	1.039	0.137	0
chr8	37701397	37707431	**BRF2**	-	6034	2.345	435	707	344	2.054	1.038	0.191	0
chr7	140396480	140406446	**NDUFB2**	+	9966	1.867	575	934	455	2.052	1.037	0.169	0
chr5	89811444	89825401	**LYSMD3**	-	13957	3.235	1406	2283	1113	2.05	1.036	0.135	0
chr20	34287231	34288902	**ROMO1**	+	1671	3.934	201	327	159	2.05	1.036	0.245	0
chr1	202847409	202858385	**RABIF**	-	10976	2.012	678	1101	537	2.049	1.035	0.172	0
chr11	63527363	63536113	**C11orf95**	-	8750	1.171	311	506	247	2.049	1.035	0.231	0
chr2	75873908	75889334	**MRPL19**	+	15426	2.464	1173	1903	929	2.047	1.033	0.147	0
chr7	100797685	100804557	**AP1S1**	+	6872	2.154	451	733	358	2.047	1.034	0.204	0
chr15	77223961	77242601	**RCN2**	+	18640	1.7	985	1597	781	2.045	1.032	0.142	0
chr9	33461350	33473941	**NOL6**	-	12591	1.791	690	1120	547	2.045	1.032	0.177	0
chr7	123181082	123197958	**NDUFA5**	-	16876	1.716	892	1446	708	2.041	1.029	0.157	0
chr2	8822112	8824583	**ID2**	+	2471	18.661	1432	2320	1137	2.041	1.029	0.139	0
chr17	16284366	16286054	**UBB**	+	1688	4.944	257	416	204	2.041	1.03	0.227	0
chr19	12907633	12912694	**PRDX2**	-	5061	1.518	234	379	186	2.038	1.027	0.247	0
chr2	128458596	128461407	**SFT2D3**	+	2811	2.168	187	302	148	2.038	1.027	0.256	0
chr5	140080031	140086239	**ZMAT2**	+	6208	3.497	671	1085	533	2.037	1.027	0.168	0
chr16	56965747	56977793	**HERPUD1**	+	12046	7.685	2871	4642	2281	2.035	1.025	0.151	0
chr8	124260689	124287781	**ZHX1**	-	27092	1.275	1078	1743	857	2.034	1.024	0.139	0
chr19	44416775	44439411	**ZNF45**	-	22636	1.024	721	1165	572	2.034	1.025	0.156	0
chr17	27046999	27051374	**RPL23A**	+	4375	4.745	644	1041	511	2.034	1.024	0.165	0
chr17	39981333	39992523	**NT5C3L**	-	11190	1.061	365	590	290	2.033	1.024	0.21	0
chr22	36622254	36636000	**APOL2**	-	13746	1.518	642	1036	510	2.032	1.023	0.18	0
chr9	37780307	37785067	**EXOSC3**	-	4760	4.641	677	1094	538	2.032	1.023	0.181	0
chr4	155456148	155471585	**PLRG1**	-	15437	2.68	1286	2077	1022	2.031	1.022	0.142	0
chr15	78575577	78591940	**WDR61**	-	16363	1.982	1006	1624	800	2.03	1.021	0.149	0
chr12	69979207	69995357	**CCT2**	+	16150	4.069	2034	3284	1617	2.03	1.021	0.149	0
chr19	55912649	55919325	**UBE2S**	-	6676	5.737	1185	1913	942	2.03	1.021	0.149	0
chr1	167885912	167906307	**BRP44**	-	20395	2.196	1401	2260	1115	2.027	1.019	0.137	0
chr14	35179587	35184029	**CFL2**	-	4442	11.886	1664	2684	1325	2.026	1.019	0.129	0
chrX	40440215	40465888	**ATP6AP2**	+	25673	1.015	810	1306	645	2.025	1.018	0.154	0
chr18	11851388	11854448	**CHMP1B**	+	3060	7.018	653	1054	520	2.025	1.018	0.196	0
chr15	89002708	89010633	**MRPL46**	-	7925	2.004	489	788	390	2.02	1.015	0.194	0
chr3	169490852	169507504	**MYNN**	+	16652	2.88	1498	2410	1194	2.018	1.013	0.141	0
chr17	56422538	56429563	**SUPT4H1**	-	7025	1.182	256	412	204	2.017	1.012	0.234	0
chr3	51991469	52001482	**PCBP4**	-	10013	2.059	631	1015	503	2.015	1.011	0.192	0
chr1	110026560	110035420	**ATXN7L2**	+	8860	2.59	699	1124	557	2.015	1.011	0.195	0
chr20	34129777	34145405	**ERGIC3**	+	15628	1.72	828	1330	660	2.013	1.01	0.173	0
chr2	85569077	85581821	**RETSAT**	-	12744	1.177	459	737	366	2.013	1.009	0.214	0
chr4	184426219	184432249	**ING2**	+	6030	1.942	364	584	290	2.012	1.009	0.205	0
chr1	154929501	154934258	**PYGO2**	-	4757	3.311	480	771	383	2.012	1.009	0.217	0
chr3	119388371	119396243	**COX17**	-	7872	1.393	343	551	274	2.011	1.008	0.196	0
chr2	18735988	18741959	**RDH14**	-	5971	2.175	400	643	319	2.011	1.008	0.21	0

chr19	57901217	57913919	**ZNF548**	+	12702	1.923	750	1203	599	2.009	1.006	0.184	0
chr1	202101976	202113866	**ARL8A**	-	11890	1.733	632	1014	505	2.007	1.005	0.192	0
chr1	173446485	173457946	**PRDX6**	+	11461	10.774	3900	6251	3116	2.006	1.004	0.147	0
chr11	64008412	64011607	**FKBP2**	+	3195	1.237	120	193	96	2.006	1.004	0.303	0
chr4	666224	668127	**ATP5I**	-	1903	2.944	174	278	139	2.004	1.003	0.256	0
chr19	19030483	19039442	**DDX49**	+	8959	1.175	321	515	257	2.003	1.002	0.241	0
chr11	65729159	65747607	**SART1**	+	18448	1.396	797	1277	638	2.001	1.001	0.17	0
**sum**					**8232857**								
**average size**					**8920**								

Table S2B. Genes DOWN-regulated >2-fold following 45 min CPT treatment (CPT).

chrom	start	end	name	strand	bp	meanRPKM	meanCount	1_CPT	2_cont	foldChange	log2FoldCha	pAdjusted	significant
chr6	26271145	26271612	**HIST1H3G**	-	467	11.336	171	6	226	0.026	-5.238216	0	1
chr6	1312674	1314993	**FOXQ1**	+	2319	1.831	133	7	175	0.044	-4.507032	0.001	1
chr11	19372270	20143147	**NAV2**	+	770877	1.901	47753	4304	62236	0.069	-3.853773	0.048	1
chr12	1726221	1756378	**WNT5B**	+	30157	3.972	3936	365	5127	0.071	-3.810714	0	1
chr1	115828536	115880857	**NGF**	-	52321	5.566	9895	941	12880	0.073	-3.773715	0.001	1
chr18	57098170	57364644	**CCBE1**	-	266474	5.065	45435	4738	59000	0.08	-3.638166	0.058	1
chr8	70378858	70573147	**SULF1**	+	194289	5.248	36717	4351	47506	0.092	-3.448683	0.056	1
chr5	64444562	64777704	**ADAMTS6**	-	333142	2.078	24175	2893	31269	0.093	-3.433711	0.023	1
chr14	51955854	52197444	**FRMD6**	+	241590	14.097	117269	15262	151272	0.101	-3.309075	0.435	0
chr13	60239722	60738119	**DIAPH3**	-	498397	1.514	25642	3430	33046	0.104	-3.267888	0.031	1
chr14	73136659	73360809	**DPF3**	-	224150	1.681	12505	1668	16117	0.104	-3.272259	0.006	1
chr8	31497267	32622558	**NRG1**	+	1125291	1.296	49669	6660	64005	0.104	-3.264425	0.105	0
chr16	17196180	17564738	**XYLT1**	-	368558	1.638	20034	2834	25768	0.11	-3.184347	0.021	1
chr13	38210772	38443939	**TRPC4**	-	233167	1.666	13525	1932	17389	0.111	-3.169752	0.012	1
chr10	78629358	79397577	**KCNMA1**	-	768219	2.886	72829	10754	93520	0.115	-3.120337	0.219	0
chr10	49654078	49813138	**ARHGAP22**	-	159060	2.984	15426	2374	19776	0.12	-3.057817	0.015	1
chr12	120123594	120315095	**CIT**	-	191501	1.446	9191	1410	11784	0.12	-3.062199	0.006	1
chr22	46481876	46509808	**MIRLET7BHG**	+	27932	5.437	4899	768	6276	0.122	-3.029847	0.002	1
chr5	113698015	113832197	**KCNN2**	+	134182	3.476	15624	2489	20002	0.124	-3.006013	0.019	1
chr5	126112314	126172712	**LMNB1**	+	60398	1.956	3955	636	5061	0.126	-2.991573	0.002	1
chr1	208195587	208417665	**PLXNA2**	-	222078	1.184	8886	1430	11371	0.126	-2.99076	0.008	1
chr7	83587658	83824217	**SEMA3A**	-	236559	2.401	19773	3186	25302	0.126	-2.989319	0.033	1
chr19	38397867	38699008	**SIPA1L3**	+	301141	1.493	14980	2413	19169	0.126	-2.989599	0.018	1
chr4	157682762	157892546	**PDGFC**	-	209784	3.713	26022	4493	33199	0.135	-2.885262	0.056	1
chr1	94027348	94147385	**BCAR3**	-	120037	7.572	30014	5191	38288	0.136	-2.882673	0.067	1
chr20	52183609	52199636	**ZNF217**	-	16027	8.383	4457	787	5680	0.139	-2.850866	0.004	1
chr7	55086724	55275031	**EGFR**	+	188307	4.235	26168	4682	33330	0.14	-2.831587	0.057	1
chr2	36583369	36778278	**CRIM1**	+	194909	25.278	170222	31017	216624	0.143	-2.804045	0.86	0
chr11	123396527	123493518	**GRAMD1B**	+	96991	1.404	4498	818	5724	0.143	-2.806663	0.004	1
chr1	201617449	201796102	**NAV1**	+	178653	3.391	20394	3715	25953	0.143	-2.804313	0.044	1
chr3	71003864	71633140	**FOXP1**	-	629276	1.369	29341	5382	37327	0.144	-2.793782	0.079	1
chr15	81071711	81243999	**KIAA1199**	+	172288	9.442	55888	10220	71111	0.144	-2.798657	0.207	0
chr10	80828791	81076285	**ZMIZ1**	+	247494	4.635	37377	6868	47547	0.144	-2.791323	0.103	0
chr12	66218239	66360071	**HMGA2**	+	141832	9.784	44384	8247	56430	0.146	-2.774434	0.133	0
chr16	56642477	56643409	**MT2A**	+	932	43.357	1285	239	1634	0.146	-2.772028	0.002	1
chr20	9049700	9461462	**PLCB4**	+	411762	4.461	65275	12107	82998	0.146	-2.777229	0.275	0
chr18	55313658	55470327	**ATP8B1**	-	156669	4.443	23221	4369	29505	0.148	-2.75532	0.056	1
chr3	42132745	42267268	**TRAK1**	+	134523	2.521	11170	2109	14190	0.149	-2.749669	0.018	1
chr16	87863628	87903100	**SLC7A5**	-	39472	5.033	6344	1211	8055	0.15	-2.732687	0.007	1
chr21	36160097	36421595	**RUNX1**	-	261498	4.259	36973	7065	46942	0.151	-2.732	0.115	0
chr9	82186877	82341656	**TLE4**	+	154779	21.798	117591	22664	149234	0.152	-2.719074	0.571	0
chr16	86600856	86602537	**FOXC2**	+	1681	2.462	128	24	162	0.153	-2.707658	0.031	1
chr2	235860627	235964358	**SH3BP4**	+	103731	6.912	23422	4652	29679	0.157	-2.673523	0.061	1
chr14	21668237	21675059	**LOC283624**	-	6822	7.523	1677	335	2125	0.158	-2.663773	0.004	1
chr12	27849427	27850566	**REP15**	+	1139	2.199	85	17	108	0.158	-2.658454	0.068	1
chr2	9346893	9545812	**ASAP2**	+	198919	1.969	12820	2596	16228	0.16	-2.644012	0.027	1
chr1	97543299	98386615	**DPYD**	-	843316	1.512	43859	8887	55517	0.16	-2.643114	0.174	0
chr17	18874380	18908060	**FAM83G**	-	33680	2.306	2507	512	3172	0.161	-2.631214	0.004	1
chr11	33563876	33695646	**C11orf41**	+	131770	1.503	6570	1348	8310	0.162	-2.62383	0.012	1
chr18	55711609	56068772	**NEDD4L**	+	357163	1.427	16557	3399	20943	0.162	-2.623288	0.039	1
chr19	45971252	45978437	**FOSB**	+	7185	19.393	4358	898	5512	0.163	-2.616413	0.006	1
chr17	75277491	75496678	**9-Sep**	+	219187	3.117	22019	4623	27817	0.166	-2.588836	0.062	1
chr7	66018552	66043498	**LOC493754**	-	24946	1.96	1616	338	2042	0.166	-2.591637	0.005	1
chr2	207945528	208030614	**KLF7**	-	85086	7.664	22261	4694	28117	0.167	-2.582525	0.073	1
chr3	192514604	192635950	**MB21D2**	-	121346	4.193	16978	3610	21435	0.168	-2.5699	0.049	1
chr3	156977531	157221415	**VEPH1**	-	243884	1.007	8278	1760	10451	0.168	-2.569334	0.02	1
chr16	81478774	81745367	**CMIP**	+	266593	1.517	13239	2831	16709	0.169	-2.561159	0.033	1
chr14	95883830	95942173	**C14orf49**	-	58343	1.298	2439	522	3077	0.17	-2.558938	0.005	1
chr1	56960418	57045257	**PPAP2B**	-	84839	4.503	12714	2723	16045	0.17	-2.558821	0.033	1
chr5	14143828	14509458	**TRIO**	+	365630	8.003	98323	21057	124078	0.17	-2.558812	0.485	0
chr10	22823765	23003503	**PIP4K2A**	-	179738	2.811	16320	3591	20563	0.175	-2.517561	0.046	1
chr11	12132137	12285331	**MICAL2**	+	153194	4.832	24549	5442	30918	0.176	-2.506254	0.088	1
chr7	130565750	130598069	**LOC646329**	-	32319	40.216	42611	9480	53654	0.177	-2.500747	0.178	0
chr9	36572904	36677679	**MELK**	+	104775	1.407	4912	1108	6180	0.179	-2.47959	0.014	1
chr5	72921982	73237818	**RGNEF**	+	315836	1.232	12815	2892	16123	0.179	-2.478987	0.038	1
chr7	116312458	116438440	**MET**	+	125982	2.838	11942	2706	15020	0.18	-2.472249	0.037	1
chr9	113636053	113800365	**LPAR1**	-	164312	6.525	35699	8200	44866	0.183	-2.451872	0.154	0

chr5	178537851	178772431	**ADAMTS2**	-	234580	2.107	16276	3780	20441	0.185	-2.434774	0.057	1
chr9	126141932	126692417	**DENND1A**	-	550485	1.32	23898	5550	30014	0.185	-2.434845	0.093	1
chr2	121554866	121750229	**GLI2**	+	195363	1.525	9873	2288	12401	0.185	-2.438204	0.031	1
chr18	46065426	46389586	**CTIF**	+	324160	2.737	29545	6939	37080	0.187	-2.417738	0.13	0
chr7	137559724	137686846	**CREB3L2**	-	127122	5.02	21439	5051	26902	0.188	-2.41286	0.089	1
chr5	172741725	172756506	**STC2**	-	14781	16.337	7625	1796	9568	0.188	-2.413577	0.02	1
chr5	9035137	9546233	**SEMA5A**	-	511096	2.318	39400	9343	49419	0.189	-2.403017	0.187	0
chr1	66999824	67210768	**SGIP1**	+	210944	8.001	56589	13409	70982	0.189	-2.404161	0.288	0
chr2	40339285	40739575	**SLC8A1**	-	400290	2.077	29308	6961	36757	0.189	-2.400675	0.149	0
chr19	47421932	47508333	**ARHGAP35**	+	86401	3.266	9324	2226	11690	0.19	-2.39243	0.032	1
chr8	118811601	119124058	**EXT1**	-	312457	12.071	127077	30309	159333	0.19	-2.394191	0.676	0
chr1	240255184	240638489	**FMN2**	+	383305	5.771	76070	18098	95394	0.19	-2.398066	0.414	0
chr19	13945329	13947100	**LOC284454**	-	1771	36.218	2041	487	2559	0.19	-2.393617	0.008	1
chr7	36429431	36493400	**ANLN**	+	63969	4.914	10569	2529	13249	0.191	-2.38907	0.039	1
chr22	46692637	46726707	**GTSE1**	+	34070	1.355	1498	358	1878	0.191	-2.389198	0.008	1
chr14	71996041	72206120	**SIPA1L1**	+	210079	3.134	22371	5348	28045	0.191	-2.390549	0.099	1
chr12	64238540	64541613	**SRGAP1**	+	303073	4.751	48856	11692	61244	0.191	-2.388947	0.254	0
chr5	169290718	169407744	**FAM196B**	-	117026	2.193	8656	2093	10843	0.193	-2.372824	0.033	1
chr18	52889561	53303188	**TCF4**	-	413627	2.958	42907	10362	53755	0.193	-2.375036	0.236	0
chr19	15348300	15391262	**BRD4**	-	42962	3.561	4959	1205	6211	0.194	-2.365763	0.016	1
chr13	30083550	30169825	**SLC7A1**	-	86275	5.274	14975	3634	18755	0.194	-2.367664	0.058	1
chr4	148653452	148993927	**ARHGAP10**	+	340475	1.776	20158	4912	25239	0.195	-2.361076	0.088	1
chr3	29322802	30051886	**RBMS3**	+	729084	1.178	29729	7274	37214	0.195	-2.355018	0.154	0
chr4	114372187	114683083	**CAMK2D**	-	310896	3.341	34955	8569	43750	0.196	-2.352067	0.176	0
chr6	57182421	57513376	**PRIM2**	+	330955	1.633	17874	4375	22373	0.196	-2.354135	0.074	1
chr4	99391517	99579812	**TSPAN5**	-	188295	4.272	26742	6566	33467	0.196	-2.349561	0.125	0
chr9	99148224	99180669	**ZNF367**	-	32445	1.289	1389	341	1738	0.196	-2.348708	0.011	1
chr9	128199672	128469513	**MAPKAP1**	-	269841	1.869	16733	4120	20937	0.197	-2.345278	0.071	1
chr4	150999425	151178608	**DCLK2**	+	179183	10.743	66443	16429	83114	0.198	-2.338861	0.377	0
chr18	25530929	25757445	**CDH2**	-	226516	5.188	40999	10194	51267	0.199	-2.330251	0.233	0
chr4	160188997	160281301	**RAPGEF2**	+	92304	1.871	5998	1494	7499	0.199	-2.327604	0.029	1
chr1	162602227	162750247	**DDR2**	+	148020	4.189	20531	5128	25665	0.2	-2.323065	0.094	1
chr8	25042286	25270619	**DOCK5**	+	228333	4.358	33531	8465	41886	0.202	-2.306839	0.178	0
chr4	16503164	16900424	**LDB2**	-	397260	1.12	15527	3909	19400	0.202	-2.311062	0.082	1
chr17	78518624	78940173	**RPTOR**	+	421549	1.568	21578	5443	26957	0.202	-2.308006	0.097	1
chr2	168810529	169104105	**STK39**	-	293576	1.734	16954	4275	21181	0.202	-2.30861	0.078	1
chr8	128806778	129113499	**PVT1**	+	306721	2.625	26322	6684	32868	0.203	-2.297722	0.127	0
chr9	118916070	119164600	**PAPPA**	+	248530	1.139	9494	2421	11852	0.204	-2.29134	0.043	1
chr4	72053002	72437804	**SLC4A4**	+	384802	1.173	15686	3991	19584	0.204	-2.294622	0.084	1
chr15	67358194	67487533	**SMAD3**	+	129339	6.379	26537	6753	33131	0.204	-2.294524	0.123	0
chr5	148521053	148639999	**ABLIM3**	+	118946	1.775	6950	1782	8673	0.205	-2.282818	0.03	1
chr14	101361106	101373305	**MEG8**	+	12199	2.398	960	246	1198	0.205	-2.283005	0.015	1
chr9	37915894	38069210	**SHB**	-	153316	2.7	13605	3482	16980	0.205	-2.285764	0.061	1
chr5	140787769	140790232	**PCDHGB6**	+	2463	1.336	106	27	133	0.206	-2.280421	0.088	1
chr2	56411257	56613309	**CCDC85A**	+	202052	7.901	56132	14476	70017	0.207	-2.273964	0.346	0
chr8	119201694	119634184	**SAMD12**	-	432490	1.201	17679	4567	22050	0.207	-2.27143	0.094	1
chr4	1873122	1983934	**WHSC1**	+	110812	2.467	9002	2320	11230	0.207	-2.275162	0.039	1
chr9	112810877	112934791	**AKAP2**	+	123914	6.397	25984	6749	32396	0.208	-2.263047	0.133	0
chr10	128594022	129250780	**DOCK1**	+	656758	1.152	24909	6449	31062	0.208	-2.267823	0.127	0
chr19	18367905	18385319	**KIAA1683**	-	17414	1.037	587	152	732	0.208	-2.262121	0.021	1
chr4	20255234	20620788	**SLIT2**	+	365554	3.167	39429	10241	49159	0.208	-2.26301	0.229	0
chr5	95220801	95297775	**ELL2**	-	76974	22.967	61878	16098	77138	0.209	-2.260493	0.38	0
chr17	64298925	64806862	**PRKCA**	+	507937	3.728	63065	16459	78601	0.209	-2.255671	0.363	0
chr9	18474078	18910947	**ADAMTSL1**	+	436869	1.336	18953	4963	23616	0.21	-2.250387	0.091	1
chr20	45838380	45985474	**ZMYND8**	-	147094	1.323	6427	1678	8011	0.21	-2.254821	0.03	1
chr5	137801180	137805004	**EGR1**	+	3824	46.097	5533	1452	6893	0.211	-2.246322	0.021	1
chr1	156495196	156542396	**IQGAP3**	-	47200	1.647	2558	676	3185	0.212	-2.235122	0.017	1
chr12	11182985	11184006	**TAS2R31**	-	1021	1.147	38	10	48	0.213	-2.233204	0.206	0
chr8	131064350	131455906	**ASAP1**	-	391556	6.319	82190	21864	102299	0.214	-2.226155	0.479	0
chr2	27615489	27616443	**FTH1P3**	-	954	1.026	32	8	40	0.214	-2.223442	0.227	0
chr7	139246315	139477693	**HIPK2**	-	231378	1.751	13438	3578	16724	0.214	-2.224642	0.069	1
chr3	151985828	152183569	**MBNL1**	+	197741	8.867	59627	15853	74218	0.214	-2.226975	0.361	0
chr2	225629806	225907330	**DOCK10**	-	277524	4.598	43330	11588	53911	0.215	-2.21794	0.266	0
chr3	127407908	127542051	**MGLL**	-	134143	1.51	6607	1764	8222	0.215	-2.220448	0.032	1
chr10	33466418	33623833	**NRP1**	-	157415	8.389	43935	11776	54655	0.215	-2.214391	0.259	0
chr1	197053256	197115824	**ASPM**	-	62568	3.07	6452	1735	8025	0.216	-2.209507	0.035	1
chr2	66662531	66799891	**MEIS1**	+	137360	6.998	32708	8798	40679	0.216	-2.209012	0.2	0
chr3	27257096	27410912	**NEK10**	-	153816	1.755	8953	2405	11135	0.216	-2.210625	0.047	1
chr1	214776531	214837914	**CENPF**	+	61383	2.445	4986	1347	6199	0.217	-2.201866	0.028	1
chr1	85784167	86044046	**DDAH1**	-	259879	7.92	69782	18852	86759	0.217	-2.20223	0.427	0
chr1	180238797	180243816	**LOC100527964**	-	5019	1.477	238	64	297	0.217	-2.207359	0.053	1

chr11	58389023	58390145	**ZFP91-CNTF**	+	1122	3.468	130	35	162	0.217	-2.206482	0.096	1
chr6	45296053	45518819	**RUNX2**	+	222766	1.772	13177	3564	16381	0.218	-2.200293	0.072	1
chr1	243651534	244006886	**AKT3**	-	355352	1.889	22801	6219	28328	0.22	-2.187482	0.139	0
chr11	58390145	58393205	**CNTF**	+	3060	4.304	427	116	530	0.22	-2.186432	0.034	1
chr13	110959630	111165373	**COL4A2**	+	205743	2.039	13948	3818	17325	0.22	-2.181804	0.078	1
chr10	126676417	126849624	**CTBP2**	-	173207	2.626	14878	4089	18474	0.221	-2.175589	0.08	1
chr17	55333930	55757299	**MSI2**	+	423369	1.318	18495	5086	22965	0.221	-2.174866	0.106	0
chr4	170015406	170192249	**SH3RF1**	-	176843	3.835	22874	6265	28411	0.221	-2.180971	0.139	0
chr5	1253286	1295162	**TERT**	-	41876	1.789	2382	654	2958	0.221	-2.176812	0.016	1
chr16	72816785	73092534	**ZFHX3**	-	275749	2.614	24011	6581	29821	0.221	-2.179967	0.142	0
chr3	63989697	63997917	**LOC100507062**	+	8220	2.628	725	199	900	0.222	-2.172226	0.027	1
chr12	11174270	11175170	**TAS2R19**	-	900	1.541	46	12	57	0.222	-2.170703	0.205	0
chr8	126104082	126379367	**NSMCE2**	+	275285	1.052	9657	2669	11986	0.223	-2.166994	0.057	1
chr2	215593274	215674428	**BARD1**	-	81154	2.271	6117	1702	7589	0.224	-2.156305	0.037	1
chr3	172472297	172539263	**ECT2**	+	66966	2.032	4549	1271	5642	0.225	-2.150311	0.031	1
chr22	50171537	50173958	**LOC90834**	-	2421	2.18	172	48	213	0.225	-2.154718	0.078	1
chr8	59717976	60031767	**TOX**	-	313791	2.74	29188	8130	36207	0.225	-2.154821	0.189	0
chr10	60272903	60588845	**BICC1**	+	315942	2.654	27976	7854	34683	0.226	-2.142629	0.177	0
chr3	187896330	187898596	**FLJ42393**	+	2266	3.129	229	64	285	0.226	-2.14767	0.062	1
chr6	128289923	128841870	**PTPRK**	-	551947	4.193	77393	21704	95956	0.226	-2.144377	0.48	0
chr12	66151800	66220754	**RPSAP52**	-	68954	1.169	2568	719	3184	0.226	-2.145975	0.018	1
chr1	46085715	46089731	**CCDC17**	-	4016	1.067	137	38	170	0.227	-2.139969	0.089	1
chr4	2627158	2734302	**FAM193A**	+	107144	1.597	5635	1585	6985	0.227	-2.139907	0.035	1
chr6	26033319	26033796	**HIST1H2AB**	-	477	2.908	45	12	56	0.228	-2.13494	0.206	0
chr3	11178778	11304939	**HRH1**	+	126161	1.316	5422	1529	6720	0.228	-2.135781	0.033	1
chr12	72666528	73059422	**TRHDE**	+	392894	2.435	32500	9183	40273	0.228	-2.132733	0.218	0
chr6	144612872	145174170	**UTRN**	+	561298	1.823	34443	9729	42681	0.228	-2.133142	0.227	0
chr7	31792631	32338383	**PDE1C**	-	545752	2.915	53883	15273	66753	0.229	-2.127799	0.355	0
chr20	44441254	44445596	**UBE2C**	+	4342	3.45	487	138	604	0.229	-2.1297	0.035	1
chr19	36545782	36596012	**WDR62**	+	50230	1.137	1846	523	2287	0.229	-2.128249	0.019	1
chr22	42556018	42611445	**TCF20**	-	55427	1.073	1969	560	2439	0.23	-2.122752	0.022	1
chr13	97874573	98046374	**MBNL2**	+	171801	6.038	35900	10293	44436	0.232	-2.110066	0.258	0
chr15	40453209	40513337	**BUB1B**	+	60128	1.092	2175	626	2691	0.233	-2.101996	0.024	1
chr7	42000547	42276618	**GLI3**	-	276071	1.344	12222	3525	15121	0.233	-2.100905	0.077	1
chr8	23154409	23261722	**LOXL2**	-	107313	6.271	22095	6376	27334	0.233	-2.100006	0.14	0
chr20	42543491	42698254	**TOX2**	+	154763	1.125	5699	1644	7050	0.233	-2.100405	0.037	1
chr17	13399005	13505244	**HS3ST3A1**	-	106239	1.633	5790	1675	7162	0.234	-2.095506	0.043	1
chr7	127292201	127732659	**SND1**	+	440458	2.56	37437	10821	46309	0.234	-2.097427	0.249	0
chr12	109176465	109251359	**SSH1**	-	74894	8.383	20526	5938	25389	0.234	-2.09603	0.131	0
chr7	92234234	92465941	**CDK6**	-	231707	4.515	35317	10250	43673	0.235	-2.091087	0.246	0
chr5	108083522	108523373	**FER**	+	439851	1.296	19183	5571	23720	0.235	-2.08997	0.135	0
chr5	16662015	16936385	**MYO10**	-	274370	3.306	30302	8796	37471	0.235	-2.090792	0.207	0
chr15	81293294	81296345	**MESDC1**	+	3051	4.203	402	117	496	0.236	-2.08033	0.04	1
chr13	33677271	34250932	**STARD13**	-	573661	1.301	25036	7304	30947	0.236	-2.083057	0.174	0
chr11	109964086	110042566	**ZC3H12C**	+	78480	1.768	4598	1338	5684	0.236	-2.086142	0.035	1
chr6	16299342	16761721	**ATXN1**	-	462379	2.73	41744	12283	51564	0.238	-2.069631	0.282	0
chr14	55034329	55260033	**SAMD4A**	+	225704	4.364	32809	9658	40527	0.238	-2.069022	0.228	0
chr6	33589155	33664348	**ITPR3**	+	75193	1.602	3834	1133	4734	0.239	-2.063003	0.028	1
chr12	96051582	96184536	**NTN4**	-	132954	3.25	14681	4339	18129	0.239	-2.062849	0.111	0
chr5	140782519	140785006	**PCDHGA9**	+	2487	1.555	124	36	154	0.239	-2.06455	0.109	0
chr5	98104998	98132198	**RGMB**	+	27200	25.724	23067	6809	28487	0.239	-2.064756	0.155	0
chr16	70721341	70835061	**VAC14**	-	113720	1.755	6356	1879	7849	0.239	-2.062448	0.04	1
chr4	40812043	41216635	**APBB2**	-	404592	1.613	21704	6423	26797	0.24	-2.060743	0.151	0
chr11	12695968	12966284	**TEAD1**	+	270316	4.319	38880	11519	48001	0.24	-2.058983	0.274	0
chr1	210111537	210337633	**SYT14**	+	226096	1.301	9460	2815	11674	0.241	-2.051795	0.062	1
chr20	39807088	39928739	**ZHX3**	-	121651	2.573	10495	3122	12953	0.241	-2.052368	0.079	1
chr6	157099063	157531913	**ARID1B**	+	432850	1.731	25171	7542	31047	0.243	-2.041377	0.187	0
chr9	16409500	16870786	**BNC2**	-	461286	1.698	26176	7846	32286	0.243	-2.040859	0.191	0
chr15	59428562	59665071	**MYO1E**	-	236509	1.671	12975	3886	16004	0.243	-2.042059	0.091	1
chr9	127019884	127114719	**NEK6**	+	94835	4.115	12557	3769	15486	0.243	-2.038551	0.083	1
chr3	27414213	27498245	**SLC4A7**	-	84032	6.661	19113	5727	23575	0.243	-2.041258	0.149	0
chr9	128509616	128729655	**PBX3**	+	220039	5.186	37197	11198	45863	0.244	-2.033986	0.256	0
chr4	48499379	48782316	**FRYL**	-	282937	1.494	14286	4321	17607	0.245	-2.026769	0.113	0
chr15	99192760	99507759	**IGF1R**	+	314999	1.788	18859	5688	23249	0.245	-2.031145	0.141	0
chr10	91461366	91534700	**KIF20B**	+	73334	1.281	3146	948	3879	0.245	-2.032053	0.035	1
chr3	187871662	188608460	**LPP**	+	736798	2.26	55708	16851	68661	0.245	-2.026655	0.39	0
chr4	7760439	7941653	**AFAP1**	-	181214	2.707	16118	4893	19860	0.246	-2.020824	0.117	0
chr4	81187741	81212171	**FGF5**	+	24430	15.032	12056	3653	14857	0.246	-2.023779	0.088	1
chr15	58983301	58985324	**HSP90AB4P**	-	2023	1.04	70	21	87	0.246	-2.021172	0.191	0
chr4	177604690	177713895	**VEGFC**	-	109205	11.614	41935	12717	51674	0.246	-2.022607	0.3	0
chr12	77415025	77459360	**E2F7**	-	44335	2.476	3595	1092	4429	0.247	-2.01932	0.034	1

chr11	28042162	28129746	**KIF18A**	-	87584	1.843	5451	1656	6717	0.247	-2.019984	0.052	1
chr13	52158483	52336171	**WDFY2**	+	177688	2.415	14167	4338	17444	0.249	-2.007607	0.107	0
chr1	209848669	209849735	**G0S2**	+	1066	5.154	175	54	215	0.25	-1.997457	0.095	1
chr6	158402887	158520207	**SYNJ2**	+	117320	2.512	9560	2955	11762	0.251	-1.992623	0.072	1
chrX	9431334	9687780	**TBL1X**	+	256446	1.124	9492	2935	11678	0.251	-1.991963	0.076	1
chr10	125505151	125651500	**CPXM2**	-	146349	1.617	7703	2386	9475	0.252	-1.988962	0.062	1
chr6	148663728	148873184	**SASH1**	+	209456	2.395	16554	5140	20359	0.253	-1.985589	0.131	0
chr17	61086897	61505067	**TANC2**	+	418170	3.087	44213	13753	54367	0.253	-1.982986	0.347	0
chr6	42192668	42419783	**TRERF1**	-	227115	1.87	13870	4307	17057	0.253	-1.985555	0.105	0
chr11	128328655	128457453	**ETS1**	-	128798	3.937	16570	5177	20368	0.254	-1.975898	0.129	0
chr4	5712923	5816031	**EVC**	+	103108	2.206	7419	2314	9121	0.254	-1.978874	0.062	1
chr6	15246526	15522253	**JARID2**	+	275727	2.141	19215	5996	23621	0.254	-1.977805	0.144	0
chr10	94352824	94415152	**KIF11**	+	62328	1.624	3384	1059	4159	0.255	-1.973337	0.042	1
chr11	95711439	96076344	**MAML2**	-	364905	4.177	51934	16266	63824	0.255	-1.972238	0.397	0
chr5	140800536	140803653	**PCDHGA11**	+	3117	1.948	197	61	242	0.255	-1.971895	0.097	1
chr12	116396380	116714991	**MED13L**	-	318611	3.853	41760	13125	51305	0.256	-1.966784	0.335	0
chr10	134351352	134596984	**INPP5A**	+	245632	1.024	8247	2611	10126	0.258	-1.955056	0.072	1
chr1	165796731	165880855	**UCK2**	+	84124	2.012	5534	1751	6795	0.258	-1.956127	0.053	1
chr10	75910942	76469061	**ADK**	+	558119	1.598	29834	9500	36612	0.259	-1.946229	0.245	0
chr12	2162415	2807115	**CACNA1C**	+	644700	2.616	55275	17558	67847	0.259	-1.950122	0.4	0
chr20	54944444	54967351	**AURKA**	-	22907	1.735	1314	419	1612	0.26	-1.943282	0.035	1
chr9	97488950	97849500	**C9orf3**	+	360550	1.985	23699	7588	29070	0.261	-1.937582	0.196	0
chr13	110801309	110959496	**COL4A1**	-	158187	1.729	9075	2900	11133	0.261	-1.940393	0.084	1
chr3	45636322	45722755	**LIMD1**	+	86433	1.336	3764	1204	4617	0.261	-1.939163	0.043	1
chr1	170633312	170708541	**PRRX1**	+	75229	4.988	12271	3934	15051	0.261	-1.935734	0.103	0
chr9	80335190	80646219	**GNAQ**	-	311029	1.711	17786	5735	21803	0.263	-1.926617	0.157	0
chr2	238395877	238463961	**MLPH**	+	68084	1.703	3715	1202	4553	0.264	-1.920885	0.041	1
chr19	11257830	11266484	**SPC24**	-	8654	1.207	339	109	416	0.264	-1.923006	0.075	1
chr9	100961279	101018003	**TBC1D2**	-	56724	1.837	3366	1088	4126	0.264	-1.922631	0.04	1
chr13	95672082	95953687	**ABCC4**	-	281605	1.132	10387	3373	12725	0.265	-1.91554	0.093	1
chr11	43918852	43921424	**LOC729799**	+	2572	1.759	145	47	178	0.265	-1.918123	0.126	0
chr21	16333555	16437126	**NRIP1**	-	103571	4.096	14308	4647	17528	0.265	-1.915096	0.137	0
chr4	95373037	95589377	**PDLIM5**	+	216340	4.274	31088	10094	38086	0.265	-1.915743	0.268	0
chr15	60639349	60690185	**ANXA2**	-	50836	27.695	46940	15291	57489	0.266	-1.910544	0.373	0
chr3	67410883	67705038	**SUCLG2**	-	294155	1.785	17272	5623	21155	0.266	-1.911651	0.15	0
chr20	30326903	30389603	**TPX2**	+	62700	2.117	4402	1433	5392	0.266	-1.911698	0.055	1
chr14	69340839	69446083	**ACTN1**	-	105244	11.846	40650	13285	49772	0.267	-1.905476	0.321	0
chr6	129898239	130031370	**ARHGAP18**	-	133131	3.811	17279	5658	21152	0.267	-1.902434	0.169	0
chr16	23690200	23701688	**PLK1**	+	11488	3.731	1392	455	1704	0.267	-1.903824	0.036	1
chr1	178062863	178448648	**RASAL2**	+	385785	1.87	24073	7856	29479	0.267	-1.907772	0.214	0
chr19	4909509	4962165	**UHRF1**	+	52656	1.627	2773	907	3395	0.267	-1.903848	0.04	1
chr1	17866329	18024370	**ARHGEF10L**	+	158041	1.033	5270	1726	6451	0.268	-1.901784	0.056	1
chr4	104026962	104119566	**CENPE**	-	92604	1.251	3861	1265	4727	0.268	-1.90071	0.054	1
chr12	11802787	12048325	**ETV6**	+	245538	1.993	16415	5388	20090	0.268	-1.898484	0.155	0
chr10	21823100	22032559	**MLLT10**	+	209459	1.967	13540	4445	16572	0.268	-1.898382	0.125	0
chr5	169010637	169031781	**CCDC99**	+	21144	6.773	4855	1598	5941	0.269	-1.89385	0.066	1
chr16	64980682	65155919	**CDH11**	-	175237	4.218	24707	8119	30236	0.269	-1.896769	0.222	0
chr10	101635333	101769676	**DNMBP**	-	134343	3.249	14504	4768	17750	0.269	-1.896158	0.137	0
chr3	99357453	99515158	**COL8A1**	+	157705	2.598	13962	4611	17079	0.27	-1.888895	0.145	0
chr6	26031816	26032288	**HIST1H3B**	-	472	13.616	210	69	257	0.27	-1.890335	0.112	0
chr8	89049459	89339717	**MMP16**	-	290258	1.095	10694	3533	13081	0.27	-1.888326	0.113	0
chr17	12569206	12670651	**MYOCD**	+	101445	1.422	5140	1695	6288	0.27	-1.890838	0.085	1
chr12	11148560	11150474	**TAS2R20**	-	1914	1.325	85	28	104	0.27	-1.890292	0.21	0
chr4	185615218	185655286	**MLF1IP**	-	40068	1.072	1406	466	1720	0.271	-1.882418	0.04	1
chr11	43702142	43878169	**HSD17B12**	+	176027	1.022	5954	1982	7278	0.272	-1.876641	0.07	1
chr12	27677044	27848497	**PPFIBP1**	+	171453	2.513	14568	4861	17804	0.273	-1.872783	0.15	0
chr1	19665266	19812066	**CAPZB**	-	146800	4.287	20345	6799	24860	0.274	-1.870314	0.177	0
chr11	48002109	48192394	**PTPRJ**	+	190285	1.292	8235	2757	10061	0.274	-1.867489	0.094	1
chr4	1723216	1746905	**TACC3**	+	23689	1.915	1469	491	1795	0.274	-1.869592	0.039	1
chr15	85923870	86292586	**AKAP13**	+	368716	3.104	38148	12807	46595	0.275	-1.863271	0.332	0
chr1	212208918	212278187	**DTL**	+	69269	1.077	2454	825	2997	0.275	-1.860991	0.047	1
chr15	91509267	91537804	**PRC1**	-	28537	2.241	2100	705	2565	0.275	-1.861549	0.045	1
chr3	15708743	15901053	**ANKRD28**	-	192310	3.771	24250	8180	29607	0.276	-1.855685	0.23	0
chr13	98795433	99102023	**FARP1**	+	306590	1.762	17947	6055	21911	0.276	-1.855429	0.176	0
chr6	7107829	7252213	**RREB1**	+	144384	3.115	14668	4945	17909	0.276	-1.856522	0.139	0
chr9	107543283	107690527	**ABCA1**	-	147244	1.298	6036	2039	7368	0.277	-1.853078	0.061	1
chr7	8008422	8128709	**GLCCI1**	+	120287	1.217	4848	1637	5918	0.277	-1.853862	0.065	1
chr3	65339905	66024509	**MAGI1**	-	684604	1.306	29967	10129	36580	0.277	-1.852531	0.278	0
chr12	1100403	1605099	**ERC1**	+	504696	1.057	17873	6053	21814	0.278	-1.849403	0.181	0
chr6	108881025	109005971	**FOXO3**	+	124946	1.926	7953	2694	9707	0.278	-1.849277	0.092	1
chr6	17759413	17987854	**KIF13A**	-	228441	2.874	21937	7435	26772	0.278	-1.848267	0.215	0

chr9	134735498	134955253	**MED27**	-	219755	1.242	8946	3034	10917	0.278	-1.847147	0.097	1
chr7	158424002	158497520	**NCAPG2**	-	73518	1.427	3456	1173	4217	0.278	-1.845776	0.056	1
chr18	2571509	2616634	**NDC80**	+	45125	1.262	1896	643	2314	0.278	-1.84691	0.049	1
chr3	136055998	136471245	**STAG1**	-	415247	1.675	23358	7931	28500	0.278	-1.845254	0.229	0
chr17	76000317	76104916	**TNRC6C**	+	104599	1.039	3602	1223	4396	0.278	-1.845769	0.058	1
chr10	320129	735608	**DIP2C**	-	415479	1.69	22917	7810	27952	0.279	-1.839416	0.212	0
chr1	65730429	65881552	**DNAJC6**	+	151123	1.358	6790	2310	8283	0.279	-1.841926	0.083	1
chr4	123747862	123819390	**FGF2**	+	71528	11.959	28857	9807	35207	0.279	-1.843888	0.277	0
chr15	37183221	37393500	**MEIS2**	-	210279	4.066	28407	9679	34649	0.279	-1.839914	0.266	0
chr6	20100934	20212670	**MBOAT1**	-	111736	1.926	7403	2531	9027	0.28	-1.834588	0.104	0
chr22	18270415	18507325	**MICAL3**	-	236910	1.397	10732	3669	13087	0.28	-1.834655	0.109	0
chr4	119201192	119273922	**PRSS12**	-	72730	2.341	5826	1989	7106	0.28	-1.836395	0.088	1
chr15	64791618	64978266	**ZNF609**	+	186648	1.102	6889	2350	8402	0.28	-1.83814	0.089	1
chr7	132469622	132766828	**CHCHD3**	-	297206	1.068	10416	3568	12699	0.281	-1.831188	0.113	0
chr2	234745485	234763212	**HJURP**	-	17727	6.134	3523	1205	4295	0.281	-1.833887	0.054	1
chr10	129894924	129924468	**MKI67**	-	29544	4.247	4133	1415	5039	0.281	-1.832413	0.062	1
chr3	81538849	81810950	**GBE1**	-	272101	2.478	22553	7750	27487	0.282	-1.826488	0.227	0
chr2	227596032	227663506	**IRS1**	-	67474	2.967	6642	2279	8096	0.282	-1.828479	0.085	1
chr5	156904311	157002783	**ADAM19**	-	98472	2.661	8702	3002	10601	0.283	-1.819941	0.105	0
chr2	231577556	231685790	**CAB39**	+	108234	5.487	19818	6840	24144	0.283	-1.819426	0.206	0
chr8	141668480	142011412	**PTK2**	-	342932	2.263	25898	8929	31554	0.283	-1.821202	0.255	0
chr6	125304513	125404661	**RNF217**	+	100148	1.605	5444	1886	6630	0.284	-1.813714	0.084	1
chr15	41624891	41673248	**NUSAP1**	+	48357	1.367	2189	759	2666	0.285	-1.812681	0.054	1
chr3	64079525	64211131	**PRICKLE2**	-	131606	1.519	6653	2306	8103	0.285	-1.812866	0.089	1
chr17	17584786	17714765	**RAI1**	+	129979	2.003	8383	2905	10208	0.285	-1.81279	0.092	1
chr2	201170603	201346986	**SPATS2L**	+	176383	3.059	18126	6294	22070	0.285	-1.809911	0.196	0
chr9	130267617	130341268	**FAM129B**	-	73651	5.949	14090	4907	17151	0.286	-1.805239	0.141	0
chr6	26199786	26200216	**HIST1H2BF**	+	430	1.254	17	6	20	0.286	-1.805276	0.41	0
chrX	21958714	22012955	**SMS**	+	54241	2.883	5154	1791	6275	0.286	-1.808403	0.073	1
chr22	46067677	46241187	**ATXN10**	+	173510	5.318	30550	10660	37180	0.287	-1.802311	0.292	0
chr20	30193085	30194317	**ID1**	+	1232	150.342	5875	2053	7149	0.287	-1.799888	0.07	1
chr7	27870192	28220437	**JAZF1**	-	350245	2.42	28421	9941	34581	0.287	-1.79845	0.285	0
chr16	86508130	86542466	**LOC400550**	-	34336	2.213	2427	847	2954	0.287	-1.801972	0.047	1
chr5	142657495	142815077	**NR3C1**	-	157582	3.675	19553	6827	23796	0.287	-1.801353	0.216	0
chr12	100967488	101018685	**GAS2L3**	+	51197	1.745	2919	1021	3552	0.288	-1.798123	0.056	1
chr7	104622193	104631612	**LOC100216546**	-	9419	5.051	1572	550	1913	0.288	-1.796845	0.054	1
chr13	47127295	47327175	**LRCH1**	+	199880	1.336	8852	3102	10768	0.288	-1.795444	0.111	0
chr11	9800213	10315754	**SBF2**	-	515541	1.239	21430	7503	26072	0.288	-1.796969	0.228	0
chr4	146681887	146859607	**ZNF827**	-	177720	1.497	8993	3146	10942	0.288	-1.798314	0.121	0
chr8	141541263	141645646	**EIF2C2**	-	104383	1.651	5540	1954	6735	0.29	-1.784893	0.073	1
chr21	26955086	26955536	**LINC00515**	-	450	1.524	21	7	26	0.29	-1.787566	0.398	0
chr5	34656432	34832717	**RAI14**	+	176285	2.862	16678	5889	20275	0.29	-1.783423	0.184	0
chr1	230202955	230417875	**GALNT2**	+	214920	1.679	11830	4179	14380	0.291	-1.782735	0.137	0
chr9	3824127	4300035	**GLIS3**	-	475908	1.077	17279	6120	20998	0.291	-1.778589	0.201	0
chr13	110406183	110438914	**IRS2**	-	32731	2.782	2929	1036	3560	0.291	-1.781059	0.055	1
chr13	76194569	76434006	**LMO7**	+	239437	11.774	95688	33895	116285	0.291	-1.778529	0.701	0
chr8	134467090	134584183	**ST3GAL1**	-	117093	1.901	7158	2535	8699	0.291	-1.77877	0.088	1
chr8	25316512	25365425	**CDCA2**	+	48913	1.634	2637	936	3203	0.292	-1.774298	0.061	1
chr2	151324709	151344180	**RND3**	-	19471	71.169	44572	15791	54165	0.292	-1.778204	0.378	0
chr4	114821439	114900878	**ARSJ**	-	79439	3.329	8918	3172	10833	0.293	-1.771741	0.126	0
chr3	194123402	194188968	**ATP13A3**	-	65566	3.235	6935	2465	8425	0.293	-1.772634	0.094	1
chr12	24962957	25102393	**BCAT1**	-	139436	1.275	6001	2138	7289	0.293	-1.769332	0.097	1
chr2	163027199	163100045	**FAP**	-	72846	2.675	6394	2276	7766	0.293	-1.770642	0.091	1
chr2	30454396	30482899	**LBH**	+	28503	7.263	7079	2522	8598	0.293	-1.769309	0.115	0
chr4	17812524	17846487	**NCAPG**	+	33963	2.475	2802	998	3403	0.293	-1.769475	0.065	1
chr10	855483	931702	**LARP4B**	-	76219	2.554	6387	2278	7757	0.294	-1.767252	0.092	1
chr15	48700502	48937985	**FBN1**	-	237483	5.224	42189	15122	51211	0.295	-1.759756	0.408	0
chr5	125695787	125829853	**GRAMD3**	+	134066	5.988	27360	9803	33212	0.295	-1.760363	0.303	0
chr10	69869249	69971773	**MYPN**	+	102524	1.844	6307	2260	7657	0.295	-1.76047	0.097	1
chr12	78225068	78606790	**NAV3**	+	381722	3.423	44922	16082	54535	0.295	-1.761707	0.435	0
chr3	196769430	197025447	**DLG1**	-	256017	2.182	18816	6762	22834	0.296	-1.75572	0.223	0
chr5	43014830	43018913	**LOC648987**	-	4083	2.182	288	103	350	0.296	-1.75649	0.117	0
chr15	57210832	57580714	**TCF12**	+	369882	2.273	28394	10197	34460	0.296	-1.756786	0.306	0
chr19	56124958	56129907	**ZNF865**	+	4949	1.199	186	66	226	0.296	-1.758157	0.135	0
chr16	4382224	4389598	**GLIS2**	+	7374	2.002	471	169	571	0.297	-1.750852	0.081	1
chr13	114747193	114898095	**RASA3**	-	150902	1.994	9668	3483	11729	0.297	-1.751413	0.116	0
chr4	141786724	142054616	**RNF150**	-	267892	1.911	17824	6422	21625	0.297	-1.751543	0.237	0
chr8	62413114	62627199	**ASPH**	-	214085	7.997	57138	20658	69298	0.298	-1.746104	0.498	0
chr18	8717368	8832775	**CCDC165**	+	115407	2.772	10426	3772	12644	0.298	-1.744729	0.132	0
chr17	16946073	17095962	**MPRIP**	+	149889	2.41	11738	4242	14236	0.298	-1.746438	0.14	0
chr2	65537984	65659656	**SPRED2**	-	121672	1.618	6395	2313	7756	0.298	-1.745587	0.092	1

chr1	103342022	103574052	**COL11A1**	-	232030	6.536	52456	19003	63607	0.299	-1.742902	0.496	0
chr2	27008881	27017455	**CENPA**	+	8574	1.94	536	195	649	0.301	-1.732408	0.082	1
chr14	65877309	66210839	**FUT8**	+	333530	1.423	15914	5809	19283	0.301	-1.730944	0.202	0
chr10	3109711	3178997	**PFKP**	+	69286	1.35	3043	1111	3688	0.301	-1.730302	0.066	1
chr11	122526397	122685187	**UBASH3B**	+	158790	1.402	7225	2636	8754	0.301	-1.731447	0.101	0
chr22	35796115	35820495	**MCM5**	+	24380	1.161	917	335	1111	0.302	-1.729133	0.064	1
chr1	120454175	120612317	**NOTCH2**	-	158142	1.72	9000	3295	10902	0.302	-1.72621	0.13	0
chr7	24836163	25019760	**OSBPL3**	-	183597	3.381	20542	7519	24883	0.302	-1.726493	0.237	0
chr9	35490006	35561895	**RUSC2**	+	71889	4.399	10622	3891	12865	0.302	-1.725204	0.152	0
chr3	57994126	58157982	**FLNB**	+	163856	1.512	8170	2995	9896	0.303	-1.724266	0.12	0
chr3	171757417	172118492	**FNDC3B**	+	361075	3.335	40236	14743	48734	0.303	-1.72485	0.396	0
chr2	189157389	189460652	**GULP1**	+	303263	2.921	30394	11152	36808	0.303	-1.722651	0.345	0
chr2	135011829	135212192	**MGAT5**	+	200363	1.24	8348	3062	10110	0.303	-1.722869	0.132	0
chr1	163291722	163325553	**NUF2**	+	33831	1.707	1897	695	2298	0.303	-1.724513	0.064	1
chr1	214522038	214725024	**PTPN14**	-	202986	3.253	22015	8086	26657	0.303	-1.721013	0.257	0
chr12	50382944	50419307	**RACGAP1**	-	36363	1.724	2085	764	2525	0.303	-1.724718	0.068	1
chr3	114056946	114866127	**ZBTB20**	-	809181	1.185	32198	11821	38991	0.303	-1.721739	0.343	0
chr6	151186814	151423023	**MTHFD1L**	+	236209	2.342	18307	6733	22164	0.304	-1.718799	0.22	0
chr3	123331142	123603149	**MYLK**	-	272007	7.017	61792	22749	74806	0.304	-1.717345	0.515	0
chr1	38158158	38175391	**CDCA8**	+	17233	3.036	1709	630	2068	0.305	-1.714274	0.063	1
chr8	25276773	25282556	**GNRH1**	-	5783	1.29	248	91	300	0.305	-1.710754	0.151	0
chr2	127413683	127454246	**GYPC**	+	40563	1.57	2025	747	2451	0.305	-1.714171	0.056	1
chr3	124481794	124606144	**ITGB5**	-	124350	1.266	5224	1928	6323	0.305	-1.712955	0.096	1
chr1	10535002	10690815	**PEX14**	+	155813	1.371	7006	2583	8480	0.305	-1.714893	0.108	0
chr13	41506054	41593508	**ELF1**	-	87454	1.88	5550	2056	6714	0.306	-1.706982	0.108	0
chr16	67973786	67977376	**LCAT**	-	3590	2.127	242	90	293	0.306	-1.706101	0.134	0
chr2	20448452	20527144	**PUM2**	-	78692	2.964	7788	2886	9423	0.306	-1.707073	0.127	0
chr9	134452156	134612925	**RAPGEF1**	-	160769	1.309	6815	2524	8245	0.306	-1.707797	0.101	0
chr3	16357351	16555222	**RFTN1**	-	197871	1.284	8393	3103	10156	0.306	-1.710596	0.127	0
chr8	100025493	100889808	**VPS13B**	+	864315	1.011	29272	10828	35420	0.306	-1.709789	0.326	0
chr20	37554954	37581703	**FAM83D**	+	26749	2.403	2091	778	2529	0.308	-1.699414	0.065	1
chr3	119540801	119813264	**GSK3B**	-	272463	2.546	23099	8595	27934	0.308	-1.70035	0.274	0
chr10	64926987	65225722	**JMJD1C**	-	298735	2.367	23758	8837	28731	0.308	-1.700903	0.284	0
chr14	92980124	93155334	**RIN3**	+	175210	1.094	6232	2322	7535	0.308	-1.6979	0.099	1
chr17	1963132	2207069	**SMG6**	-	243937	1.513	12086	4504	14613	0.308	-1.697836	0.161	0
chr8	12940871	13372429	**DLC1**	-	431558	2.06	30245	11282	36567	0.309	-1.696517	0.348	0
chr6	33665345	33679504	**C6orf125**	-	14159	1.069	491	184	594	0.31	-1.688014	0.097	1
chr19	10244021	10305755	**DNMT1**	-	61734	2.143	4286	1606	5179	0.31	-1.68897	0.083	1
chr2	197063976	197457335	**HECW2**	-	393359	1.486	19353	7250	23388	0.31	-1.689707	0.24	0
chr5	149887673	149937773	**NDST1**	+	50100	3.165	5168	1934	6246	0.31	-1.690751	0.093	1
chr14	24641233	24649463	**REC8**	+	8230	1.033	268	100	324	0.31	-1.691271	0.126	0
chr15	32907690	32931868	**ARHGAP11A**	+	24178	1.447	1163	436	1405	0.311	-1.686618	0.074	1
chr11	122943032	123066007	**CLMP**	-	122975	2.635	10548	3968	12742	0.311	-1.682939	0.147	0
chr21	38739858	38887679	**DYRK1A**	+	147821	5.173	25488	9585	30789	0.311	-1.683493	0.301	0
chr19	18553472	18632937	**ELL**	-	79465	2.541	6460	2424	7806	0.311	-1.686819	0.098	1
chr5	130977406	131132756	**FNIP1**	-	155350	3.425	17179	6452	20755	0.311	-1.685583	0.205	0
chr16	2479394	2508859	**CCNF**	+	29465	1.406	1331	501	1607	0.312	-1.68021	0.064	1
chr6	27100816	27101314	**HIST1H2AG**	+	498	3.42	56	21	68	0.312	-1.679092	0.327	0
chr12	58120022	58122139	**LOC100130776**	+	2117	1.489	100	37	120	0.312	-1.679432	0.219	0
chr3	30647993	30735633	**TGFBR2**	+	87640	3.259	9466	3567	11433	0.312	-1.680382	0.146	0
chr6	4706392	4955778	**CDYL**	+	249386	1.036	8523	3219	10291	0.313	-1.676483	0.137	0
chr7	2767740	2883959	**GNA12**	-	116219	1.575	5966	2253	7203	0.313	-1.676785	0.103	0
chr1	41492870	41707815	**SCMH1**	-	214945	1.106	7919	2996	9560	0.313	-1.673642	0.136	0
chr7	55538305	55640200	**VOPP1**	-	101895	1.634	5439	2056	6567	0.313	-1.674976	0.099	1
chr11	35160416	35253949	**CD44**	+	93533	23.323	72126	27372	87044	0.314	-1.669035	0.606	0
chr2	15307031	15701454	**NBAS**	-	394423	1.016	13298	5045	16049	0.314	-1.66938	0.19	0
chr15	66994673	67074337	**SMAD6**	+	79664	2.462	6503	2464	7849	0.314	-1.671021	0.12	0
chr6	80714321	80752244	**TTK**	+	37923	1.445	1814	687	2190	0.314	-1.671101	0.075	1
chr17	1958392	1962981	**HIC1**	+	4589	3.687	528	200	637	0.315	-1.668091	0.083	1
chr4	185308875	185395726	**IRF2**	-	86851	1.026	2925	1112	3529	0.315	-1.665908	0.08	1
chr12	60083125	60175408	**SLC16A7**	+	92283	1.349	4198	1594	5066	0.315	-1.668073	0.102	0
chr8	23386362	23430063	**SLC25A37**	+	43701	1.388	1954	741	2358	0.315	-1.668774	0.066	1
chr10	31608100	31818742	**ZEB1**	+	210642	2.237	15726	5980	18974	0.315	-1.665699	0.219	0
chr1	20915443	20945400	**CDA**	+	29957	1.303	1287	490	1552	0.316	-1.66235	0.075	1
chr4	159690181	159827954	**FNIP2**	+	137773	2.013	9050	3447	10918	0.316	-1.663336	0.139	0
chr4	142949181	143767604	**INPP4B**	-	818423	2.388	63777	24277	76944	0.316	-1.664177	0.552	0
chr5	176732500	176739292	**MXD3**	-	6792	1.431	312	119	376	0.316	-1.66004	0.132	0
chr15	40092930	40213093	**GPR176**	-	120163	4.993	20109	7693	24247	0.317	-1.656146	0.268	0
chr19	46386865	46389376	**IRF2BP1**	-	2511	1.415	112	42	135	0.317	-1.65532	0.21	0
chr1	119425665	119532179	**TBX15**	-	106514	1.477	5205	1991	6276	0.317	-1.656004	0.107	0
chr10	114710008	114927436	**TCF7L2**	+	217428	1.228	8774	3356	10580	0.317	-1.656188	0.141	0

chr16	28109315	28223190	**XPO6**	-	113875	3.47	12903	4936	15558	0.317	-1.656317	0.18	0
chr2	122095351	122407052	**CLASP1**	-	311701	1.923	19873	7611	23961	0.318	-1.654534	0.258	0
chr1	197473878	197744623	**DENND1B**	-	270745	1.262	11584	4436	13967	0.318	-1.654434	0.19	0
chr11	72547789	72853143	**FCHSD2**	-	305354	1.965	20310	7775	24488	0.318	-1.655036	0.277	0
chr11	58294343	58345639	**LPXN**	-	51296	4.355	7311	2806	8813	0.318	-1.650985	0.127	0
chr2	238536223	238690290	**LRRFIP1**	+	154067	2.661	13356	5131	16097	0.319	-1.64938	0.187	0
chr17	60556385	60692841	**TLK2**	+	136456	1.479	6751	2592	8137	0.319	-1.650037	0.133	0
chr6	52362199	52441862	**TRAM2**	-	79663	7.598	19899	7649	23982	0.319	-1.648477	0.255	0
chr21	44263203	44299678	**WDR4**	-	36475	1.433	1668	641	2010	0.319	-1.647792	0.066	1
chr1	164528596	164821060	**PBX1**	+	292464	1.037	9983	3851	12027	0.32	-1.643076	0.158	0
chr6	37787306	38122399	**ZFAND3**	+	335093	2.852	31875	12301	38399	0.32	-1.642212	0.366	0
chr17	35441926	35766902	**ACACA**	-	324976	1.095	11554	4471	13915	0.321	-1.637738	0.169	0
chr22	42334740	42343148	**CENPM**	-	8408	2.532	680	263	819	0.321	-1.63743	0.086	1
chr2	218664511	218808796	**TNS1**	-	144285	1.856	8856	3426	10666	0.321	-1.638234	0.151	0
chr2	235401685	235405693	**ARL4C**	-	4008	1.517	194	75	234	0.322	-1.635053	0.173	0
chr15	36871811	37102449	**C15orf41**	+	230638	1.016	7786	3028	9372	0.323	-1.629811	0.144	0
chr2	113495443	113522254	**CKAP2L**	-	26811	1.883	1670	649	2010	0.323	-1.63078	0.083	1
chr6	485137	693109	**EXOC2**	-	207972	1.011	6930	2692	8342	0.323	-1.631678	0.133	0
chr2	192110106	192290115	**MYO1B**	+	180009	5.31	32693	12726	39349	0.323	-1.62852	0.398	0
chr5	119800018	120022964	**PRR16**	+	222946	2.658	19688	7651	23700	0.323	-1.63114	0.268	0
chr10	35535952	35860847	**CCNY**	+	324895	1.079	11509	4493	13848	0.324	-1.623859	0.18	0
chr5	102594441	102614361	**C5orf30**	+	19920	14.966	10179	3976	12246	0.325	-1.622928	0.191	0
chr4	186131283	186285120	**SNX25**	+	153837	1.3	6793	2657	8171	0.325	-1.620328	0.148	0
chr11	121959810	122073770	**MIR100HG**	-	113960	5.694	21249	8330	25556	0.326	-1.617165	0.277	0
chr11	46417963	46612914	**AMBRA1**	-	194951	1.307	8412	3311	10112	0.327	-1.610525	0.153	0
chr8	80831094	80942506	**MRPS28**	-	111412	1.107	4120	1621	4953	0.327	-1.61145	0.112	0
chr12	106457124	106533811	**NUAK1**	-	76687	2.556	6795	2675	8168	0.327	-1.610508	0.164	0
chr9	134305476	134375575	**PRRC2B**	+	70099	2.421	5543	2177	6665	0.327	-1.614032	0.116	0
chr5	86564069	86687743	**RASA1**	+	123674	3.303	13604	5354	16354	0.327	-1.610826	0.217	0
chr5	39371775	39425335	**DAB2**	-	53560	19.306	35049	13805	42130	0.328	-1.609631	0.417	0
chr3	154797435	154901518	**MME**	+	104083	1.766	5865	2309	7050	0.328	-1.609945	0.109	0
chr3	179370932	179507189	**USP13**	+	136257	1.297	5848	2306	7029	0.328	-1.607894	0.129	0
chr18	74069636	74207146	**ZNF516**	-	137510	2.108	9430	3714	11335	0.328	-1.609549	0.156	0
chr15	93443550	93571237	**CHD2**	+	127687	5.757	24420	9650	29343	0.329	-1.604333	0.321	0
chr3	73431651	73674072	**PDZRN3**	-	242421	1.664	13503	5339	16224	0.329	-1.603233	0.222	0
chr1	156182778	156209868	**PMF1**	+	27090	10.707	8851	3501	10634	0.329	-1.602509	0.121	0
chr1	44173217	44396831	**ST3GAL3**	+	223614	1.148	8439	3332	10141	0.329	-1.605446	0.153	0
chr10	69681655	69835103	**HERC4**	-	153448	9.084	45668	18085	54862	0.33	-1.601004	0.468	0
chr4	41362803	41702061	**LIMCH1**	+	339258	1.484	17375	6890	20870	0.33	-1.598732	0.285	0
chr19	13106583	13209610	**NFIX**	+	103027	2.012	6657	2639	7997	0.33	-1.599511	0.124	0
chr12	45609769	45834187	**ANO6**	+	224418	2.366	17736	7046	21299	0.331	-1.595792	0.266	0
chr6	101846860	102517958	**GRIK2**	+	671098	2.156	49924	19836	59953	0.331	-1.595679	0.539	0
chr4	83550689	83720010	**SCD5**	-	169321	1.085	6168	2448	7408	0.331	-1.59716	0.141	0
chr1	2160133	2241652	**SKI**	+	81519	2.568	6691	2661	8035	0.331	-1.594151	0.124	0
chr5	65222383	65376850	**ERBB2IP**	+	154467	5.536	28650	11418	34394	0.332	-1.590795	0.366	0
chr22	24666785	24813708	**SPECC1L**	+	146923	1.622	7877	3138	9456	0.332	-1.591376	0.154	0
chr9	21454266	21559697	**MIR31HG**	-	105431	2.267	7862	3143	9435	0.333	-1.585825	0.153	0
chr3	160117429	160152741	**SMC4**	+	35312	6.257	7327	2930	8793	0.333	-1.585175	0.151	0
chrY	14813159	14972768	**USP9Y**	+	159609	1.567	8316	3322	9980	0.333	-1.58681	0.164	0
chr5	109025155	109203429	**MAN2A1**	+	178274	1.198	7069	2833	8481	0.334	-1.581579	0.148	0
chr4	101944586	102268628	**PPP3CA**	-	324042	1.511	16429	6582	19712	0.334	-1.582328	0.261	0
chr2	46926098	46989927	**SOCS5**	+	63829	14.476	30760	12337	36901	0.334	-1.580591	0.38	0
chr7	116164838	116201239	**CAV1**	+	36401	23.359	28804	11577	34547	0.335	-1.577312	0.38	0
chr11	28129797	28355054	**METTL15**	+	225257	1.131	8573	3441	10283	0.335	-1.579473	0.178	0
chr16	89334034	89556969	**ANKRD11**	-	222935	2.899	20966	8437	25143	0.336	-1.575343	0.282	0
chr4	17844838	18023483	**LCORL**	-	178645	1.431	8442	3410	10120	0.337	-1.569334	0.168	0
chr1	180257351	180472022	**ACBD6**	-	214671	1.386	9851	3989	11805	0.338	-1.565036	0.188	0
chr12	15773074	15942510	**EPS8**	-	169436	2.937	16778	6794	20106	0.338	-1.565227	0.277	0
chr5	153570294	153800543	**GALNT10**	+	230249	1.285	9935	4025	11905	0.338	-1.564514	0.198	0
chr6	3722835	3752246	**PXDC1**	-	29411	7.132	6951	2815	8329	0.338	-1.564753	0.153	0
chr9	116638561	116818875	**ZNF618**	+	180314	1.584	9338	3779	11191	0.338	-1.566015	0.174	0
chrX	122318095	122624766	**GRIA3**	+	306671	1.254	12749	5177	15273	0.339	-1.560823	0.223	0
chr6	143072603	143266338	**HIVEP2**	-	193735	1.468	9428	3833	11293	0.339	-1.558658	0.187	0
chr2	161128661	161350318	**RBMS1**	-	221657	4.733	35340	14335	42341	0.339	-1.562503	0.434	0
chr5	76986994	77072185	**TBCA**	-	85191	2.173	6055	2458	7254	0.339	-1.56079	0.137	0
chr3	176738541	176915048	**TBL1XR1**	-	176507	2.487	14656	5946	17559	0.339	-1.562165	0.247	0
chr7	7222245	7288251	**C1GALT1**	+	66006	1.094	2355	958	2821	0.34	-1.558465	0.097	1
chr16	53737874	54148379	**FTO**	+	410505	1.03	13989	5689	16756	0.34	-1.55825	0.235	0
chr22	43265771	43411184	**PACSIN2**	-	145413	1.455	6920	2826	8284	0.341	-1.55165	0.149	0
chr13	30338544	30424820	**UBL3**	-	86276	2.949	8525	3481	10206	0.341	-1.551729	0.185	0
chr17	8108048	8113883	**AURKB**	-	5835	4.943	929	380	1112	0.342	-1.546214	0.097	1

chr20	30252260	30310656	**BCL2L1**	-	58396	5.664	10683	4378	12784	0.342	-1.54594	0.188	0
chr7	41728600	41742706	**INHBA**	-	14106	2.564	1220	499	1460	0.342	-1.549146	0.113	0
chr17	43003447	43025082	**KIF18B**	-	21635	2.138	1511	618	1809	0.342	-1.549001	0.096	1
chr5	77298149	77590528	**AP3B1**	-	292379	1.489	14593	5994	17459	0.343	-1.54232	0.258	0
chr2	202671197	202758263	**CDK15**	+	87066	1.904	5489	2253	6567	0.343	-1.543474	0.142	0
chr14	75745480	75748937	**FOS**	+	3457	55.672	6055	2488	7244	0.343	-1.541782	0.122	0
chr10	26727265	26856732	**APBB1IP**	+	129467	2.321	9993	4111	11953	0.344	-1.539614	0.202	0
chr7	151832009	152133090	**MLL3**	-	301081	1.27	12729	5237	15227	0.344	-1.539865	0.232	0
chr16	2570362	2580955	**AMDHD2**	+	10593	2.659	886	365	1060	0.345	-1.536722	0.092	1
chr6	20402136	20493945	**E2F3**	+	91809	2.712	8153	3367	9748	0.345	-1.533614	0.171	0
chr18	59482303	59560304	**RNF152**	-	78001	1.493	3885	1604	4645	0.345	-1.533515	0.133	0
chr11	17373308	17398868	**B7H6**	+	25560	1.326	1060	438	1268	0.346	-1.533122	0.085	1
chr2	160175489	160473059	**BAZ2B**	-	297570	1.089	10914	4513	13048	0.346	-1.531668	0.225	0
chr12	6309481	6347437	**CD9**	+	37956	1.641	1990	822	2379	0.346	-1.532806	0.092	1
chr22	23412668	23467221	**GNAZ**	+	54553	1.02	1793	741	2144	0.346	-1.533227	0.094	1
chr22	29999544	30094589	**NF2**	+	95045	1.679	5267	2177	6296	0.346	-1.53185	0.141	0
chr5	171469073	171615346	**STK10**	-	146273	1.522	7214	2981	8625	0.346	-1.53266	0.153	0
chr9	140513443	140730578	**EHMT1**	+	217135	1.399	9837	4079	11756	0.347	-1.527137	0.187	0
chr1	198126107	198291548	**NEK7**	+	165441	6.432	36419	15114	43521	0.347	-1.525812	0.47	0
chr1	178694299	178889237	**RALGPS2**	+	194938	2.177	14208	5891	16981	0.347	-1.52723	0.259	0
chr19	45596430	45650543	**PPP1R37**	+	54113	1.555	2674	1112	3195	0.348	-1.522158	0.096	1
chr1	203595914	203713209	**ATP2B4**	+	117295	7.173	27659	11536	33033	0.349	-1.517699	0.374	0
chr1	235824344	236030220	**LYST**	-	205876	1.256	8320	3465	9939	0.349	-1.520288	0.166	0
chr10	27443752	27475848	**MASTL**	+	32096	1.062	1119	466	1336	0.349	-1.518383	0.108	0
chrX	64887510	64961793	**MSN**	+	74283	4.915	11953	4984	14277	0.349	-1.518313	0.221	0
chr8	27667137	27695349	**PBK**	-	28212	1.628	1510	629	1803	0.349	-1.518479	0.106	0
chr16	3022791	3030540	**PKMYT1**	-	7749	1.905	469	195	560	0.349	-1.520126	0.135	0
chr7	84624871	84751247	**SEMA3D**	-	126376	1.757	7462	3112	8912	0.349	-1.517682	0.186	0
chr1	249104650	249120154	**SH3BP5L**	-	15504	3.408	1705	711	2037	0.349	-1.51871	0.097	1
chr18	43427573	43547305	**EPG5**	-	119732	2.715	10680	4459	12753	0.35	-1.515796	0.21	0
chr1	53971905	54199877	**GLIS1**	-	227972	1.558	11506	4809	13739	0.35	-1.514506	0.21	0
chr3	149235021	149421060	**WWTR1**	-	186039	1.441	8697	3631	10386	0.35	-1.516107	0.178	0
chr13	49550047	49783915	**FNDC3A**	+	233868	2.496	19621	8212	23424	0.351	-1.512132	0.326	0
chr13	21547175	21635722	**LATS2**	-	88547	3.602	10440	4369	12464	0.351	-1.512412	0.206	0
chr2	114737145	114764887	**LOC440900**	+	27742	1.697	1529	640	1826	0.351	-1.511089	0.101	0
chr22	20861885	20941919	**MED15**	+	80034	4.637	12030	5040	14359	0.351	-1.510374	0.218	0
chr6	147525493	147711612	**STXBP5**	+	186119	2.133	13342	5595	15924	0.351	-1.508873	0.26	0
chr11	118766850	118781613	**BCL9L**	-	14763	5.066	2395	1005	2859	0.352	-1.508106	0.1	1
chr3	135684514	135866752	**PPP2R3A**	+	182238	1.953	12002	5043	14322	0.352	-1.505872	0.248	0
chr15	56119121	56285835	**NEDD4**	-	166714	1.29	7228	3047	8622	0.353	-1.500496	0.189	0
chr11	35684352	35830930	**TRIM44**	+	146578	1.293	6267	2642	7475	0.353	-1.500246	0.166	0
chr4	184020462	184241929	**WWC2**	+	221467	2.321	17286	7286	20619	0.353	-1.500608	0.308	0
chr3	185361526	185542827	**IGF2BP2**	-	181301	2.554	15358	6484	18317	0.354	-1.498203	0.277	0
chr4	77356252	77704405	**SHROOM3**	+	348153	1.263	14660	6183	17486	0.354	-1.499646	0.274	0
chr6	158244293	158366109	**SNX9**	+	121816	3.377	13341	5633	15911	0.354	-1.498008	0.237	0
chr3	112323408	112359977	**CCDC80**	-	36569	3.988	5075	2148	6051	0.355	-1.493958	0.188	0
chr2	62900985	63273621	**EHBP1**	+	372636	2.179	27970	11848	33344	0.355	-1.492773	0.427	0
chr9	132427919	132484951	**PRRX2**	+	57032	1.952	3665	1549	4370	0.355	-1.495597	0.134	0
chr1	154947117	154951725	**CKS1B**	+	4608	1.662	252	107	300	0.356	-1.488547	0.22	0
chr15	64457715	64648442	**CSNK1G1**	-	190727	1.106	6984	2960	8325	0.356	-1.491526	0.18	0
chr13	39917028	40177356	**LHFP**	-	260328	1.747	15116	6406	18019	0.356	-1.491888	0.278	0
chr6	163835674	163999628	**QKI**	+	163954	2.779	15272	6475	18204	0.356	-1.491252	0.286	0
chr19	39138266	39221171	**ACTN4**	+	82905	9.648	26185	11147	31197	0.357	-1.484738	0.373	0
chr2	85980908	86018506	**ATOH8**	+	37598	3.457	4247	1808	5060	0.357	-1.484293	0.138	0
chr2	54683453	54898583	**SPTBN1**	+	215130	2.415	17089	7278	20359	0.357	-1.484044	0.295	0
chr8	99466860	99837909	**STK3**	-	371049	1.068	13223	5617	15758	0.357	-1.488013	0.263	0
chr3	11314009	11599139	**ATG7**	+	285130	1.175	11004	4687	13110	0.358	-1.483943	0.226	0
chr19	1275519	1279243	**C19orf24**	+	3724	1.424	166	71	198	0.358	-1.481272	0.227	0
chr11	9160374	9286873	**DENND5A**	-	126499	2.195	9152	3903	10902	0.358	-1.481855	0.208	0
chr19	2164147	2232577	**DOT1L**	+	68430	1.872	4085	1741	4866	0.358	-1.482155	0.124	0
chr2	39476421	39664219	**MAP4K3**	-	187798	2.788	17467	7450	20806	0.358	-1.481581	0.311	0
chr1	46111451	46112357	**RPS15AP10**	-	906	2.467	74	31	88	0.358	-1.480394	0.362	0
chr2	189896640	190044605	**COL5A2**	-	147965	7.404	37559	16049	44729	0.359	-1.478744	0.504	0
chr15	75966662	76005189	**CSPG4**	-	38527	1.826	2241	958	2669	0.359	-1.477197	0.105	0
chr22	31721789	31742249	**PATZ1**	-	20460	1.106	723	309	861	0.359	-1.476702	0.123	0
chr16	84509965	84538288	**KIAA1609**	-	28323	5.032	4629	1983	5511	0.36	-1.474133	0.142	0
chr1	157963062	158065844	**KIRREL**	+	102782	4.085	13767	5900	16390	0.36	-1.474058	0.26	0
chr4	128802015	128820377	**PLK4**	+	18362	1.199	727	312	865	0.361	-1.471432	0.142	0
chr3	11597543	11762220	**VGLL4**	-	164677	2.28	12425	5339	14788	0.361	-1.469767	0.255	0
chr3	105085556	105295757	**ALCAM**	+	210201	4.079	29194	12578	34732	0.362	-1.465279	0.439	0
chr7	23349827	23509995	**IGF2BP3**	-	160168	1.148	6035	2602	7179	0.362	-1.464147	0.17	0

chr16	85645028	85709812	**KIAA0182**	+	64784	1.129	2344	1010	2789	0.362	-1.465213	0.113	0
chr17	38544772	38574202	**TOP2A**	-	29430	3.918	3841	1655	4570	0.362	-1.46509	0.15	0
chr11	842823	867116	**TSPAN4**	+	24293	1.523	1175	506	1399	0.362	-1.467312	0.105	0
chr3	141043054	141168632	**ZBTB38**	+	125578	2.409	10026	4313	11931	0.362	-1.467873	0.227	0
chr11	35639734	35642421	**FJX1**	+	2687	1.073	91	39	108	0.363	-1.462856	0.309	0
chr1	62920396	63153969	**DOCK7**	-	233573	1.317	10174	4405	12098	0.364	-1.457544	0.231	0
chr7	77428108	77586821	**PHTF2**	+	158713	1.476	7905	3424	9399	0.364	-1.456554	0.219	0
chr10	105353783	105615164	**SH3PXD2A**	-	261381	1.748	14743	6382	17530	0.364	-1.457782	0.266	0
chr2	171847332	172087824	**TLK1**	-	240492	1.515	12141	5255	14436	0.364	-1.457725	0.261	0
chr2	75719443	75796848	**FAM176A**	-	77405	1.694	4359	1890	5182	0.365	-1.455114	0.159	0
chr3	108268717	108308491	**KIAA1524**	-	39774	1.607	2101	912	2497	0.365	-1.452586	0.13	0
chr6	33359312	33377699	**KIFC1**	+	18387	2.442	1458	632	1734	0.365	-1.454052	0.119	0
chr12	81191170	81331694	**LIN7A**	-	140524	1.171	5458	2368	6488	0.365	-1.454174	0.174	0
chr4	129730778	129796379	**PHF17**	+	65601	1.453	3134	1358	3726	0.365	-1.455627	0.137	0
chr1	116519118	116612675	**SLC22A15**	+	93557	1.316	3995	1731	4749	0.365	-1.455742	0.139	0
chr7	17830384	17980131	**SNX13**	-	149747	1.366	6732	2917	8003	0.365	-1.455738	0.185	0
chr3	88188261	88193814	**ZNF654**	+	5553	2.007	375	162	445	0.365	-1.452385	0.215	0
chr5	304290	438405	**AHRR**	+	134115	4.45	19304	8395	22941	0.366	-1.450184	0.318	0
chr10	63661012	63856707	**ARID5B**	+	195695	6.854	43863	19087	52122	0.366	-1.44931	0.519	0
chr10	3818187	3827473	**KLF6**	-	9286	52.937	15715	6840	18673	0.366	-1.448714	0.273	0
chr4	152041432	152149182	**SH3D19**	-	107750	1.253	4582	1994	5444	0.366	-1.44911	0.18	0
chr2	69240275	69476459	**ANTXR1**	+	236184	2.095	16235	7085	19285	0.367	-1.444629	0.3	0
chr12	105724413	105765296	**C12orf75**	+	40883	19.969	27513	12005	32682	0.367	-1.44477	0.427	0
chr3	98514813	98620533	**DCBLD2**	-	105720	3.162	10827	4716	12864	0.367	-1.447653	0.227	0
chr4	54851665	54853449	**RPL21P44**	-	1784	1.235	72	31	86	0.367	-1.444407	0.377	0
chr8	124332090	124408705	**ATAD2**	-	76615	1.558	3951	1729	4692	0.368	-1.440398	0.156	0
chr6	26240653	26241021	**HIST1H4F**	+	368	3.623	43	18	51	0.368	-1.44145	0.435	0
chr7	73113534	73134017	**STX1A**	-	20483	1.091	716	313	851	0.368	-1.443411	0.136	0
chr5	180650262	180662808	**TRIM41**	+	12546	1.078	433	189	515	0.368	-1.442164	0.169	0
chr3	52082936	52090461	**DUSP7**	-	7525	4.517	1081	474	1284	0.369	-1.437193	0.116	0
chr17	3627196	3629992	**GSG2**	+	2796	1.678	150	66	179	0.369	-1.439862	0.274	0
chr19	18390562	18392432	**JUND**	-	1870	28.045	1629	714	1934	0.369	-1.437244	0.099	1
chr17	39182278	39183454	**KRTAP1-5**	-	1176	3.597	131	57	155	0.369	-1.438511	0.262	0
chr17	29421944	29704695	**NF1**	+	282751	1.716	16194	7096	19227	0.369	-1.43799	0.318	0
chr8	38758752	38831430	**PLEKHA2**	+	72678	3.092	7424	3253	8814	0.369	-1.438027	0.205	0
chr12	2966846	2986321	**FOXM1**	-	19475	1.42	894	392	1061	0.37	-1.434219	0.134	0
chr7	104756822	105029341	**SRPK2**	-	272519	1.568	14111	6192	16750	0.37	-1.435625	0.285	0
chr10	82214037	82282391	**TSPAN14**	+	68354	2.505	5563	2446	6602	0.37	-1.432502	0.169	0
chr2	173940564	174132737	**ZAK**	+	192173	2.022	12961	5687	15386	0.37	-1.435828	0.28	0
chr6	160390130	160527583	**IGF2R**	+	137453	2.006	8858	3895	10512	0.371	-1.432149	0.205	0
chr9	19290748	19374139	**DENND4C**	+	83391	1.016	2839	1252	3368	0.372	-1.427397	0.155	0
chr16	27325250	27376099	**IL4R**	+	50849	1.361	2222	979	2636	0.372	-1.428411	0.123	0
chr14	24836144	24848810	**NFATC4**	+	12666	2.177	874	385	1036	0.372	-1.425816	0.125	0
chr6	17615268	17706818	**NUP153**	-	91550	2.168	6610	2915	7842	0.372	-1.427628	0.203	0
chr16	71678851	71748704	**PHLPP2**	-	69853	1.128	2628	1158	3118	0.372	-1.428439	0.149	0
chr12	80167342	80329235	**PPP1R12A**	-	161893	4.524	24859	10959	29492	0.372	-1.428168	0.418	0
chr7	47314751	47621742	**TNS3**	-	306991	1.885	18590	8212	22050	0.372	-1.424915	0.317	0
chr11	2423522	2425106	**TSSC4**	+	1584	2.695	135	60	161	0.372	-1.424754	0.281	0
chr6	17393735	17558023	**CAP2**	+	164288	2.035	11234	4978	13319	0.374	-1.419642	0.276	0
chr5	74666927	74807806	**COL4A3BP**	-	140879	2.123	9815	4347	11637	0.374	-1.420538	0.237	0
chr17	76670129	76778376	**CYTH1**	-	108247	1.256	4456	1975	5282	0.374	-1.419315	0.165	0
chr20	62507483	62512243	**LOC100505815**	+	4760	3.032	454	201	538	0.374	-1.418406	0.161	0
chr3	47892179	48130769	**MAP4**	-	238590	2.913	23132	10268	27420	0.374	-1.417061	0.39	0
chrY	7142012	7249588	**PRKY**	+	107576	1.086	3782	1675	4484	0.374	-1.419956	0.147	0
chr1	222841354	222885864	**AIDA**	-	44510	1.153	1694	752	2008	0.375	-1.416845	0.139	0
chr15	31619082	31670102	**KLF13**	+	51020	2.128	3473	1543	4116	0.375	-1.414964	0.138	0
chr14	61201458	61435398	**MNAT1**	+	233940	1.354	10513	4672	12459	0.375	-1.414974	0.258	0
chr11	4115923	4160106	**RRM1**	+	44183	1.173	1719	763	2038	0.375	-1.416857	0.142	0
chr3	149086804	149095568	**TM4SF1**	-	8764	2.861	813	361	964	0.375	-1.413852	0.144	0
chr17	79609348	79615779	**TSPAN10**	+	6431	1.808	364	162	431	0.375	-1.414031	0.179	0
chr12	89981825	90049844	**ATP2B1**	-	68019	2.849	6249	2786	7404	0.376	-1.409839	0.182	0
chr2	10443039	10567743	**HPCAL1**	+	124704	1.117	4436	1976	5255	0.376	-1.410671	0.148	0
chr20	35405844	35492087	**KIAA0889**	-	86243	2.693	7549	3375	8941	0.377	-1.405622	0.207	0
chr1	145611035	145688776	**RNF115**	+	77741	2.206	5706	2546	6760	0.377	-1.40862	0.196	0
chr1	156904631	157015162	**ARHGEF11**	-	110531	1.187	4295	1922	5086	0.378	-1.403309	0.168	0
chr7	134464163	134655480	**CALD1**	+	191317	29.158	191036	85493	226218	0.378	-1.403825	1	0
chr6	26250369	26250835	**HIST1H3F**	-	466	3.77	57	25	68	0.378	-1.40284	0.417	0
chr8	130853715	130952000	**FAM49B**	-	98285	1.569	5121	2300	6061	0.379	-1.397885	0.191	0
chr1	68167148	68299155	**GNG12**	-	132007	3.625	15763	7065	18662	0.379	-1.401259	0.319	0
chr11	118964584	118966177	**H2AFX**	-	1593	6.695	336	150	397	0.379	-1.398359	0.195	0
chr17	47074773	47133507	**IGF2BP1**	+	58734	1.726	3333	1494	3946	0.379	-1.400313	0.158	0

chr8	42273992	42397068	**SLC20A2**	-	123076	2.807	11271	5055	13343	0.379	-1.40025	0.259	0
chr4	79697531	79833341	**BMP2K**	+	135810	1.867	8347	3754	9878	0.38	-1.395865	0.229	0
chr8	116420723	116681228	**TRPS1**	-	260505	1.583	13689	6153	16201	0.38	-1.396762	0.305	0
chr2	43449540	43453745	**ZFP36L2**	-	4205	30.79	4072	1832	4819	0.38	-1.395474	0.141	0
chr8	74207264	74237516	**RDH10**	+	30252	1.612	1652	745	1955	0.381	-1.39149	0.166	0
chr13	99102454	99229396	**STK24**	-	126942	2.784	11416	5148	13505	0.381	-1.391285	0.256	0
chr1	21132784	21503381	**EIF4G3**	-	370597	1.577	19448	8788	23001	0.382	-1.388	0.37	0
chr10	27961802	28034778	**MKX**	-	72976	4.922	11808	5331	13968	0.382	-1.389558	0.277	0
chr12	111890017	112037480	**ATXN2**	-	147463	2.798	13680	6198	16174	0.383	-1.383718	0.309	0
chr10	71029755	71161637	**HK1**	+	131882	1.531	6483	2934	7666	0.383	-1.385595	0.19	0
chr4	57897243	57976539	**IGFBP7**	-	79296	7.443	19387	8781	22922	0.383	-1.384294	0.359	0
chr20	48429249	48508772	**SLC9A8**	+	79523	1.324	3420	1550	4043	0.383	-1.382716	0.157	0
chr13	50556687	50699677	**DLEU2**	-	142990	1.72	8147	3699	9630	0.384	-1.380328	0.24	0
chr6	27833106	27833576	**HIST1H2AL**	+	470	2.607	39	18	46	0.384	-1.380474	0.468	0
chr19	11200037	11244505	**LDLR**	+	44468	4.565	6353	2883	7510	0.384	-1.381025	0.173	0
chr5	71515235	71616084	**MRPS27**	-	100849	1.246	4161	1888	4919	0.384	-1.381091	0.183	0
chr8	19171080	19253729	**SH2D4A**	+	82649	2.316	6330	2870	7483	0.384	-1.382247	0.213	0
chr10	126630691	126676005	**ZRANB1**	+	45314	1.532	2309	1048	2730	0.384	-1.381307	0.161	0
chr6	129204285	129837710	**LAMA2**	+	633425	1.729	36894	16798	43593	0.385	-1.375765	0.535	0
chr19	3359560	3469215	**NFIC**	+	109655	1.828	6443	2931	7613	0.385	-1.37691	0.193	0
chr1	186369703	186370587	**OCLM**	+	884	1.944	56	25	66	0.385	-1.377065	0.435	0
chr10	16632616	16859453	**RSU1**	-	226837	1.591	11894	5405	14057	0.385	-1.378906	0.284	0
chr13	99852678	100038753	**UBAC2**	+	186075	1.029	6238	2835	7372	0.385	-1.378606	0.202	0
chr12	50898767	51142450	**DIP2B**	+	243683	1.014	8166	3724	9646	0.386	-1.372778	0.242	0
chr6	26045638	26046097	**HIST1H3C**	+	459	9.116	135	61	160	0.386	-1.37426	0.318	0
chr4	14113591	14141676	**LOC152742**	+	28085	2.394	2194	1000	2592	0.386	-1.374374	0.149	0
chr9	20344967	20622514	**MLLT3**	-	277547	1.264	11817	5385	13961	0.386	-1.374356	0.306	0
chr10	98741040	98745585	**C10orf12**	+	4545	1.777	266	121	314	0.387	-1.367789	0.266	0
chr18	12991360	13125051	**CEP192**	+	133691	1.458	6404	2923	7564	0.387	-1.37141	0.214	0
chr9	127115751	127121463	**LOC100129034**	+	5712	3.914	716	327	846	0.387	-1.369197	0.158	0
chr6	111620233	111804414	**REV3L**	-	184181	3.122	19032	8699	22476	0.387	-1.369388	0.37	0
chr19	31765850	31840190	**TSHZ3**	-	74340	1.438	3504	1600	4139	0.387	-1.371045	0.17	0
chr10	75183336	75193319	**ZMYND17**	-	9983	1.061	350	160	414	0.387	-1.368614	0.242	0
chr19	19496641	19619741	**GATAD2A**	+	123100	2.126	8441	3864	9966	0.388	-1.366723	0.228	0
chr18	21111462	21166581	**NPC1**	-	55119	3.177	5637	2581	6655	0.388	-1.366291	0.188	0
chr3	150126787	150177615	**TSC22D2**	+	50828	8.786	14934	6835	17633	0.388	-1.367164	0.339	0
chr21	27252860	27543446	**APP**	-	290586	2.189	20862	9582	24622	0.389	-1.361557	0.382	0
chr2	75059781	75120481	**HK2**	+	60700	2.632	5072	2329	5986	0.389	-1.36172	0.172	0
chr8	30241943	30429734	**RBPMS**	+	187791	3.554	21850	10042	25786	0.389	-1.360497	0.39	0
chr5	114938176	114948904	**TMED7-TICAM2**	-	10728	2.061	719	330	848	0.389	-1.36192	0.172	0
chr1	45205489	45233438	**KIF2C**	+	27949	1.612	1461	673	1724	0.39	-1.357181	0.148	0
chr18	12446510	12657912	**SPIRE1**	-	211402	1.053	7380	3396	8708	0.39	-1.35847	0.24	0
chr4	37892719	38140794	**TBC1D1**	+	248075	1.017	8355	3845	9859	0.39	-1.358183	0.252	0
chr12	100430862	100536642	**UHRF1BP1L**	-	105780	1.249	4383	2017	5172	0.39	-1.358444	0.196	0
chr16	84733554	84813527	**USP10**	+	79973	2.149	5630	2592	6642	0.39	-1.357261	0.206	0
chr9	108210314	108311385	**FSD1L**	+	101071	1.162	3867	1783	4562	0.391	-1.35457	0.186	0
chr12	120648241	120703574	**PXN**	-	55333	4.979	8802	4066	10381	0.392	-1.352261	0.231	0
chr12	26111963	26232825	**RASSF8**	+	120862	2.48	9930	4592	11709	0.392	-1.350204	0.278	0
chr4	80822770	80994477	**ANTXR2**	-	171707	1.655	9362	4341	11035	0.393	-1.345905	0.269	0
chr22	40806291	41032690	**MKL1**	-	226399	2.253	17146	7940	20214	0.393	-1.348116	0.375	0
chr6	117803819	117891020	**DCBLD1**	+	87201	3.106	8808	4093	10379	0.394	-1.342284	0.251	0
chr19	6210391	6279959	**MLLT1**	-	69568	1.284	2859	1328	3369	0.394	-1.342681	0.154	0
chr5	140810157	140812789	**PCDHGA12**	+	2632	1.596	132	61	156	0.394	-1.343765	0.313	0
chr15	77400497	77712446	**PEAK1**	-	311949	2.466	26002	12067	30647	0.394	-1.344629	0.468	0
chr11	66099541	66104000	**RIN1**	-	4459	4.955	697	324	822	0.394	-1.342876	0.159	0
chr9	33817181	33920401	**UBE2R2**	+	103220	2.096	7121	3308	8391	0.394	-1.342586	0.237	0
chr22	19023794	19109967	**DGCR2**	-	86173	1.377	3834	1785	4517	0.395	-1.33895	0.177	0
chr4	99182526	99365012	**RAP1GDS1**	+	182486	1.695	10402	4841	12256	0.395	-1.340002	0.304	0
chr16	11439310	11445617	**RMI2**	+	6307	1.037	209	97	247	0.395	-1.338495	0.278	0
chr17	17746821	17875784	**TOM1L2**	-	128963	1.158	4855	2258	5721	0.395	-1.341253	0.195	0
chr2	145141941	145277958	**ZEB2**	-	136017	2.236	10082	4696	11877	0.395	-1.338545	0.285	0
chr12	109038886	109125295	**CORO1C**	-	86409	8.293	23482	10941	27662	0.396	-1.33814	0.418	0
chr19	17186590	17324104	**MYO9B**	+	137514	1.586	7044	3286	8296	0.396	-1.335931	0.225	0
chr3	128444978	128533641	**RAB7A**	+	88663	4.711	13532	6316	15937	0.396	-1.335349	0.31	0
chr11	102706527	102714342	**MMP3**	-	7815	1.499	374	174	440	0.397	-1.332818	0.223	0
chr5	176560079	176727214	**NSD1**	+	167135	1.537	8500	3977	10007	0.397	-1.330995	0.268	0
chr22	36134782	36424585	**RBFOX2**	-	289803	2.686	25800	12065	30379	0.397	-1.332238	0.454	0
chr11	111473169	111597632	**SIK2**	+	124463	1.166	4846	2265	5707	0.397	-1.33315	0.221	0
chr14	61447831	61550451	**SLC38A6**	+	102620	1.184	3892	1821	4583	0.397	-1.331114	0.174	0
chr18	21572736	21715574	**TTC39C**	+	142838	1.895	8852	4142	10422	0.397	-1.331056	0.262	0
chr1	43824625	43828873	**CDC20**	+	4248	3.018	415	194	489	0.398	-1.328682	0.227	0

chr12	51985019	52202299	**SCN8A**	+	217280	1.71	12371	5790	14564	0.398	-1.330788	0.325	0
chr11	27676441	27743605	**BDNF**	-	67164	8.176	18557	8722	21836	0.399	-1.323886	0.407	0
chr6	27100094	27100575	**HIST1H2BJ**	-	481	2.307	36	17	42	0.399	-1.325008	0.52	0
chr2	176987412	176989645	**HOXD9**	+	2233	1.501	107	50	126	0.399	-1.326292	0.359	0
chr22	24236564	24237409	**MIF**	+	845	11.809	316	148	372	0.399	-1.326107	0.232	0
chr19	47634079	47713893	**SAE1**	+	79814	2.022	5262	2472	6192	0.399	-1.324474	0.211	0
chr16	3775055	3930121	**CREBBP**	-	155066	2.645	13431	6325	15800	0.4	-1.320708	0.327	0
chr13	45694630	45858239	**GTF2F2**	+	163609	1.17	6354	2990	7475	0.4	-1.321685	0.242	0
chr19	13261281	13265718	**IER2**	+	4437	12.949	1789	842	2105	0.4	-1.321669	0.136	0
chr14	50885210	50999376	**MAP4K5**	-	114166	2.853	10904	5134	12828	0.4	-1.32112	0.316	0
chr2	159825145	160089170	**TANC1**	+	264025	1.321	11425	5373	13443	0.4	-1.322978	0.301	0
chr8	119935795	119964383	**TNFRSF11B**	-	28588	26.789	24939	11740	29338	0.4	-1.321269	0.434	0
chr15	101715927	101792137	**CHSY1**	-	76210	2.343	5807	2741	6829	0.401	-1.316961	0.22	0
chr17	26369687	26523404	**NLK**	+	153717	2.178	11272	5317	13257	0.401	-1.317993	0.328	0
chr10	133747959	133770053	**PPP2R2D**	+	22094	1.199	858	404	1009	0.401	-1.318917	0.176	0
chr2	37477645	37544222	**PRKD3**	-	66577	1.248	2770	1307	3258	0.401	-1.317732	0.195	0
chr3	10327437	10335133	**GHRLOS**	+	7696	1.097	270	127	317	0.402	-1.314835	0.264	0
chr15	101459459	101610317	**LRRK1**	+	150858	1.326	6485	3065	7625	0.402	-1.314853	0.227	0
chr20	43595119	43708593	**STK4**	+	113474	1.601	5918	2794	6959	0.402	-1.316481	0.223	0
chr4	85590692	85887544	**WDFY3**	-	296852	1.61	15978	7553	18786	0.402	-1.314424	0.375	0
chr16	75327607	75467387	**CFDP1**	-	139780	1.111	5085	2408	5977	0.403	-1.31159	0.218	0
chr9	91150015	91190704	**NXNL2**	+	40689	1.398	1819	861	2138	0.403	-1.312458	0.155	0
chr11	126226095	126284533	**ST3GAL4**	+	58438	1.187	2261	1070	2658	0.403	-1.312456	0.174	0
chr3	156392204	156424557	**TIPARP**	+	32353	17.65	18706	8885	21980	0.404	-1.306654	0.387	0
chr12	98909350	98944157	**TMPO**	+	34807	2.198	2515	1194	2956	0.404	-1.306972	0.186	0
chr13	113139327	113242481	**TUBGCP3**	-	103154	1.423	4816	2284	5660	0.404	-1.308695	0.217	0
chr12	6957971	6960456	**CDCA3**	-	2485	3.442	274	130	322	0.405	-1.305666	0.269	0
chr22	19318223	19419219	**HIRA**	-	100996	1.121	3664	1745	4304	0.405	-1.302261	0.19	0
chr3	8543510	8609806	**LMCD1**	+	66296	1.233	2636	1257	3095	0.406	-1.299877	0.175	0
chr7	6201411	6312242	**CYTH3**	-	110831	2.927	10769	5144	12644	0.407	-1.2974	0.318	0
chr1	175913966	176176370	**RFWD2**	-	262404	1.395	12134	5802	14245	0.407	-1.295835	0.335	0
chr1	28837404	28865708	**RCC1**	+	28304	2.203	2025	970	2377	0.408	-1.293051	0.177	0
chr12	96672038	96794366	**CDK17**	-	122328	2.276	9239	4435	10840	0.409	-1.289403	0.301	0
chr10	73724119	73773322	**CHST3**	+	49203	2.244	3563	1709	4181	0.409	-1.290604	0.191	0
chr5	138089106	138270723	**CTNNA1**	+	181617	2.811	16837	8071	19759	0.409	-1.291581	0.382	0
chr10	95066185	95242074	**MYOF**	-	175889	8.783	50781	24362	59588	0.409	-1.290354	0.641	0
chr6	64356430	64424405	**PHF3**	+	67975	1.659	3729	1788	4376	0.409	-1.291216	0.215	0
chr3	57743173	57914894	**SLMAP**	+	171721	1.014	5780	2776	6781	0.409	-1.288407	0.25	0
chr1	27022521	27108601	**ARID1A**	+	86080	3.223	9155	4401	10739	0.41	-1.286832	0.295	0
chr1	47715810	47779819	**STIL**	-	64009	1.089	2302	1108	2700	0.41	-1.285051	0.195	0
chr2	25016174	25045245	**CENPO**	+	29071	1.49	1397	674	1638	0.411	-1.281489	0.174	0
chr18	657603	673499	**TYMS**	+	15896	2.472	1272	613	1492	0.411	-1.283176	0.177	0
chr10	24872537	25012597	**ARHGAP21**	-	140060	2.76	12867	6221	15082	0.413	-1.277502	0.356	0
chr5	10679341	10761387	**DAP**	-	82046	3.888	10348	5005	12129	0.413	-1.276939	0.301	0
chr9	95375465	95432547	**IPPK**	-	57082	1.495	2753	1332	3227	0.413	-1.275819	0.187	0
chr16	74655296	74700779	**RFWD3**	-	45483	2.5	3714	1799	4353	0.413	-1.274594	0.211	0
chr14	38677203	38682268	**SSTR1**	+	5065	26.959	4358	2107	5108	0.413	-1.277461	0.2	0
chr11	64883874	64885170	**ZNHIT2**	-	1296	1.005	40	19	47	0.413	-1.277397	0.49	0
chr12	46123619	46301819	**ARID2**	+	178200	1.102	6552	3181	7676	0.414	-1.270964	0.277	0
chr20	34995443	35157040	**DLGAP4**	+	161597	1.026	5363	2602	6284	0.414	-1.271992	0.228	0
chr8	117657054	117768062	**EIF3H**	-	111008	2.143	7857	3808	9207	0.414	-1.273739	0.284	0
chr16	55513080	55540586	**MMP2**	+	27506	5.143	4503	2186	5275	0.414	-1.270819	0.203	0
chr5	36876860	37065921	**NIPBL**	+	189061	1.928	12237	5936	14337	0.414	-1.272047	0.359	0
chr9	133589267	133763062	**ABL1**	+	173795	1.361	7804	3796	9140	0.415	-1.267392	0.285	0
chr6	74405507	74538041	**CD109**	+	132534	1.514	6521	3170	7638	0.415	-1.268847	0.251	0
chr1	227177565	227505826	**CDC42BPA**	-	328261	1.812	19940	9683	23358	0.415	-1.270403	0.442	0
chr7	156931654	157062066	**UBE3C**	+	130412	1.76	7529	3658	8819	0.415	-1.269627	0.276	0
chr2	11321777	11484711	**ROCK2**	-	162934	2.946	16134	7867	18889	0.416	-1.263616	0.409	0
chr19	16178316	16213813	**TPM4**	+	35497	9.074	10471	5105	12260	0.416	-1.263803	0.31	0
chr12	104609558	104744062	**TXNRD1**	+	134504	6.673	30254	14728	35429	0.416	-1.266382	0.54	0
chr13	111767623	111958081	**ARHGEF7**	+	190458	1.593	9857	4810	11539	0.417	-1.262232	0.303	0
chr1	246729638	246831884	**CNST**	+	102246	3.425	11608	5662	13590	0.417	-1.263074	0.343	0
chr15	80987651	81047962	**FAM108C1**	+	60311	2.712	5374	2624	6291	0.417	-1.261318	0.248	0
chr12	124808956	125052010	**NCOR2**	-	243054	1.905	14905	7282	17446	0.417	-1.26038	0.355	0
chr12	57449425	57472574	**TMEM194A**	-	23149	1.007	758	370	888	0.417	-1.261692	0.212	0
chr22	41641614	41682216	**RANGAP1**	-	40602	3.102	4062	1986	4754	0.418	-1.259129	0.212	0
chr5	60628099	60841999	**ZSWIM6**	+	213900	1.852	13178	6442	15424	0.418	-1.259597	0.368	0
chr7	32535175	32623779	**AVL9**	+	88604	2.245	6528	3202	7636	0.419	-1.253681	0.27	0
chr6	118781934	119031238	**CEP85L**	-	249304	1.324	10824	5305	12664	0.419	-1.255159	0.329	0
chr4	71859264	71896629	**DCK**	+	37365	1.196	1463	717	1712	0.419	-1.256243	0.196	0
chr7	148504463	148581441	**EZH2**	-	76978	1.331	3368	1651	3940	0.419	-1.254424	0.221	0

chr17	17115526	17140502	**FLCN**	-	24976	2.737	2152	1055	2517	0.419	-1.253665	0.17	0
chr16	86612114	86615304	**FOXL1**	+	3190	3.814	383	187	448	0.419	-1.256239	0.242	0
chr19	39616419	39670046	**PAK4**	+	53627	1.247	2127	1043	2488	0.419	-1.253377	0.178	0
chr13	28712642	28869475	**PAN3**	+	156833	2.197	11445	5609	13390	0.419	-1.255303	0.347	0
chr7	5659671	5821361	**RNF216**	-	161690	1.563	8317	4073	9732	0.419	-1.256667	0.297	0
chr7	98625057	98741743	**SMURF1**	-	116686	2.454	9395	4608	10991	0.419	-1.254073	0.31	0
chr22	50166936	50218452	**BRD1**	-	51516	2.806	4630	2274	5415	0.42	-1.251491	0.218	0
chr15	42191638	42264755	**EHD4**	-	73117	1.603	3784	1859	4426	0.42	-1.251157	0.213	0
chr5	172261222	172379688	**ERGIC1**	+	118466	1.684	6487	3187	7587	0.42	-1.251297	0.26	0
chr5	134670070	134735577	**H2AFY**	-	65507	3.811	8079	3965	9450	0.42	-1.253028	0.278	0
chr15	69706626	69740764	**KIF23**	+	34138	3.277	3690	1813	4315	0.42	-1.251174	0.229	0
chr7	39663151	39747723	**RALA**	+	84572	2.561	7091	3483	8293	0.42	-1.251658	0.277	0
chr18	59854523	59974355	**KIAA1468**	+	119832	1.355	5368	2641	6277	0.421	-1.248801	0.26	0
chr14	71374121	71582099	**PCNX**	+	207978	1.756	12141	5987	14193	0.422	-1.24519	0.362	0
chr5	67511583	67597649	**PIK3R1**	+	86066	3.61	10262	5064	11995	0.422	-1.243948	0.335	0
chr14	77972339	78083110	**SPTLC2**	-	110771	3.069	11378	5611	13301	0.422	-1.24522	0.359	0
chr2	69685126	69870977	**AAK1**	-	185851	2.719	16726	8271	19545	0.423	-1.240598	0.412	0
chr4	56298659	56412997	**CLOCK**	-	114338	1.848	7027	3473	8211	0.423	-1.241215	0.297	0
chr10	86088409	86278276	**FAM190B**	+	189867	1.904	11876	5870	13878	0.423	-1.241387	0.349	0
chr7	27565058	27702620	**HIBADH**	-	137562	1.045	4824	2383	5637	0.423	-1.242074	0.27	0
chr12	88886569	88974250	**KITLG**	-	87681	4.332	12658	6260	14790	0.423	-1.240389	0.374	0
chr19	44235300	44259142	**SMG9**	-	23842	1.294	998	493	1166	0.423	-1.242083	0.2	0
chr19	10828728	10942586	**DNM2**	+	113858	1.601	5871	2909	6858	0.424	-1.237254	0.248	0
chr5	31400601	31532282	**DROSHA**	-	131681	1.041	4498	2229	5255	0.424	-1.23727	0.244	0
chr12	69864128	69973562	**FRS2**	+	109434	2.575	9302	4606	10867	0.424	-1.238273	0.324	0
chr7	4721929	4811074	**FOXK1**	+	89145	1.277	3630	1802	4239	0.425	-1.233583	0.208	0
chr2	174937174	175113365	**OLA1**	-	176191	1.217	7104	3523	8297	0.425	-1.235777	0.297	0
chr21	17102495	17252377	**USP25**	+	149882	1.2	5982	2966	6987	0.425	-1.235894	0.282	0
chr1	109419602	109473044	**GPSM2**	+	53442	1.222	2154	1072	2515	0.426	-1.230127	0.219	0
chr17	14204505	14249492	**HS3ST3B1**	+	44987	1.766	2587	1288	3020	0.426	-1.229491	0.214	0
chr3	110790605	110913016	**PVRL3**	+	122411	3.769	15665	7791	18290	0.426	-1.231189	0.434	0
chr12	8850517	8929787	**RIMKLB**	+	79270	1.259	3263	1623	3809	0.426	-1.230395	0.226	0
chr20	56964174	57026156	**VAPB**	+	61982	2.25	4553	2262	5317	0.426	-1.232736	0.242	0
chr7	21940516	21985542	**CDCA7L**	-	45026	1.187	1728	861	2017	0.427	-1.227949	0.199	0
chr13	42614171	42803891	**DGKH**	+	189720	1.059	6685	3334	7801	0.427	-1.226248	0.301	0
chr5	139505520	139508391	**IGIP**	+	2871	1.814	172	85	201	0.427	-1.228794	0.382	0
chr9	21802634	21865969	**MTAP**	+	63335	1.569	3247	1617	3791	0.427	-1.228892	0.226	0
chr12	118587605	118810750	**TAOK3**	-	223145	1.173	8700	4334	10155	0.427	-1.228257	0.328	0
chr2	85198230	85286595	**KCMF1**	+	88365	4.258	12544	6260	14638	0.428	-1.225487	0.38	0
chr20	34359922	34538288	**PHF20**	+	178366	1.202	7059	3524	8238	0.428	-1.224989	0.296	0
chr6	35310334	35395968	**PPARD**	+	85634	1.781	4937	2471	5759	0.429	-1.220293	0.247	0
chr11	68228185	68382801	**PPP6R3**	+	154616	2.492	12695	6358	14807	0.429	-1.219491	0.374	0
chr18	9708227	9862553	**RAB31**	+	154326	1.358	6857	3434	7998	0.429	-1.219745	0.288	0
chr12	11090852	11091806	**TAS2R14**	-	954	1.155	36	18	42	0.429	-1.222168	0.565	0
chr13	24144508	24250244	**TNFRSF19**	+	105736	1.248	4378	2189	5108	0.429	-1.222173	0.261	0
chr22	20008630	20053447	**C22orf25**	+	44817	1.467	2091	1048	2438	0.43	-1.218417	0.191	0
chr10	50723150	50725167	**PGBD3**	-	2017	2.953	196	98	229	0.43	-1.218925	0.374	0
chr15	90544751	90625432	**ZNF710**	+	80681	1.163	3031	1518	3535	0.43	-1.218653	0.218	0
chr6	100956607	101329224	**ASCC3**	-	372617	2.519	31268	15724	36449	0.431	-1.21294	0.565	0
chr1	91380856	91487812	**ZNF644**	-	106956	2.838	10012	5026	11674	0.431	-1.215875	0.343	0
chr11	94501507	94609918	**AMOTL1**	+	108411	2.779	9801	4934	11423	0.432	-1.211128	0.331	0
chr19	48111452	48206534	**GLTSCR1**	+	95082	1.657	5090	2565	5932	0.432	-1.209367	0.254	0
chr14	63841354	64010079	**PPP2R5E**	-	168725	1.354	7575	3817	8827	0.432	-1.209427	0.32	0
chr12	11126253	11324224	**PRH1-PRR4**	-	197971	1.455	9562	4815	11145	0.432	-1.210823	0.347	0
chr12	14518610	14651697	**ATF7IP**	+	133087	1.708	7587	3831	8839	0.433	-1.206114	0.329	0
chr10	106071898	106093663	**ITPRIP**	-	21765	4.001	2772	1398	3230	0.433	-1.208416	0.206	0
chr8	92967194	93115454	**RUNX1T1**	-	148260	1.028	5034	2539	5866	0.433	-1.207936	0.276	0
chr17	76170159	76183285	**TK1**	-	13126	2.985	1253	632	1460	0.433	-1.207753	0.199	0
chr13	48877882	49056026	**RB1**	+	178144	1.334	7807	3950	9092	0.434	-1.202709	0.317	0
chr15	56382730	56535483	**RFX7**	-	152753	1.685	8549	4318	9960	0.434	-1.205691	0.338	0
chr19	16308664	16346156	**AP1M1**	+	37492	1.989	2364	1198	2753	0.435	-1.200517	0.201	0
chr10	99894380	100004654	**C10orf28**	+	110274	1.502	5446	2760	6342	0.435	-1.20035	0.281	0
chr5	179921416	180005353	**CNOT6**	+	83937	1.075	2969	1504	3457	0.435	-1.200493	0.242	0
chr2	158592957	158732374	**ACVR1**	-	139417	2.063	9374	4754	10915	0.436	-1.199069	0.334	0
chr10	104503726	104576021	**C10orf26**	+	72295	1.033	2442	1240	2843	0.436	-1.197281	0.231	0
chr11	73019662	73080425	**ARHGEF17**	+	60763	2.763	5426	2758	6316	0.437	-1.195283	0.268	0
chr7	140433812	140624564	**BRAF**	-	190752	1.093	6946	3529	8085	0.437	-1.195839	0.323	0
chr8	27950583	28048669	**ELP3**	+	98086	1.033	3324	1692	3868	0.437	-1.192789	0.249	0
chr1	1716724	1822526	**GNB1**	-	105802	4.435	15380	7816	17901	0.437	-1.195428	0.412	0
chr17	28443833	28513486	**NSRP1**	+	69653	2.364	5413	2754	6299	0.437	-1.193793	0.283	0
chr3	126707436	126756235	**PLXNA1**	+	48799	1.057	1634	831	1901	0.437	-1.192858	0.197	0

chr5	131630144	131679899	**SLC22A4**	+	49755	1.582	2524	1283	2937	0.437	-1.19519	0.216	0
chr6	52128811	52149582	**MCM3**	-	20771	1.418	957	488	1113	0.438	-1.189877	0.23	0
chr1	47897806	47900313	**MGC12982**	-	2507	1.58	124	63	144	0.438	-1.190149	0.388	0
chr22	36677322	36784063	**MYH9**	-	106741	10.452	36042	18373	41931	0.438	-1.19043	0.577	0
chr2	128698790	128784869	**SAP130**	-	86079	1.029	2879	1466	3350	0.438	-1.191935	0.233	0
chr2	20400557	20425194	**SDC1**	-	24637	1.458	1145	584	1332	0.438	-1.189583	0.207	0
chr2	196521531	196602426	**SLC39A10**	+	80895	1.173	3105	1583	3612	0.438	-1.190177	0.243	0
chr2	230631929	230786655	**TRIP12**	-	154726	3.104	15842	8065	18434	0.438	-1.192557	0.426	0
chr12	93166284	93323107	**EEA1**	-	156823	1.688	8748	4468	10175	0.439	-1.187217	0.343	0
chr3	129033613	129035120	**H1FX**	-	1507	7.353	345	176	402	0.439	-1.186444	0.277	0
chr12	31944118	31945175	**H3F3C**	-	1057	1.735	58	30	68	0.439	-1.188669	0.501	0
chr6	26027123	26027480	**HIST1H4B**	-	357	23.376	268	137	312	0.439	-1.187914	0.335	0
chr8	42128819	42190171	**IKBKB**	+	61352	1.336	2657	1356	3091	0.439	-1.18871	0.229	0
chr12	53645369	53648190	**MFSD5**	+	2821	2.598	231	118	269	0.439	-1.186409	0.331	0
chr6	143929316	144152322	**PHACTR2**	+	223006	1.704	12649	6455	14714	0.439	-1.188535	0.401	0
chr3	47057897	47205467	**SETD2**	-	147570	2.308	11365	5803	13218	0.439	-1.18747	0.387	0
chrX	123094474	123236505	**STAG2**	+	142031	1.468	6908	3527	8035	0.439	-1.187758	0.321	0
chr8	56792385	56925006	**LYN**	+	132621	1.275	5579	2856	6487	0.44	-1.183563	0.297	0
chr3	8918879	9005159	**RAD18**	-	86280	1.939	5606	2866	6519	0.44	-1.185554	0.313	0
chr2	55199326	55277734	**RTN4**	-	78408	11.19	28958	14825	33669	0.44	-1.183381	0.553	0
chr2	47168312	47303275	**TTC7A**	+	134963	1.624	7155	3658	8321	0.44	-1.185724	0.309	0
chr1	206858364	206907630	**MAPKAPK2**	+	49266	3.695	5853	3003	6803	0.441	-1.179624	0.277	0
chr7	44836240	44842716	**PPIA**	+	6476	7.282	1523	781	1770	0.441	-1.179876	0.221	0
chr18	56530060	56653709	**ZNF532**	+	123649	2.127	8747	4481	10168	0.441	-1.182134	0.354	0
chr17	65821779	65980494	**BPTF**	+	158715	1.517	8027	4126	9327	0.442	-1.176613	0.349	0
chr7	148395932	148498202	**CUL1**	+	102270	2.923	9783	5027	11368	0.442	-1.177046	0.354	0
chr15	22892683	23003603	**CYFIP1**	+	110920	1.732	6198	3185	7202	0.442	-1.176973	0.286	0
chr4	123091757	123283914	**KIAA1109**	+	192157	1.018	6509	3345	7563	0.442	-1.176694	0.326	0
chr3	38207025	38296979	**OXSR1**	+	89954	3.491	10345	5313	12022	0.442	-1.178076	0.367	0
chr4	139085247	139163503	**SLC7A11**	-	78256	1.673	4429	2276	5146	0.442	-1.176916	0.309	0
chr3	105377108	105587887	**CBLB**	-	210779	1.274	8962	4613	10412	0.443	-1.174417	0.363	0
chr19	4675243	4723855	**DPP9**	-	48612	1.846	2866	1474	3330	0.443	-1.175767	0.226	0
chr2	182321618	182402468	**ITGA4**	+	80850	2.109	5766	2966	6699	0.443	-1.175176	0.332	0
chr11	12399025	12556903	**PARVA**	+	157878	4.347	22549	11615	26193	0.443	-1.173138	0.5	0
chr7	29959718	30029905	**SCRN1**	-	70187	2.993	6872	3533	7985	0.443	-1.176233	0.312	0
chr8	37553300	37556396	**ZNF703**	+	3096	4.953	469	241	545	0.443	-1.17368	0.235	0
chr19	41725107	41767671	**AXL**	+	42564	7.009	9708	5004	11276	0.444	-1.171937	0.349	0
chr9	99212413	99253618	**HABP4**	+	41205	2.007	2714	1399	3152	0.444	-1.171511	0.251	0
chr8	9413444	9639856	**TNKS**	+	226412	1.477	11047	5693	12831	0.444	-1.172395	0.381	0
chr15	63334837	63364113	**TPM1**	+	29276	19.667	19095	9840	22181	0.444	-1.172557	0.476	0
chr2	61414589	61697849	**USP34**	-	283260	1.719	16103	8308	18701	0.444	-1.170497	0.444	0
chr10	62538088	62554610	**CDK1**	+	16522	2.966	1602	827	1860	0.445	-1.168298	0.237	0
chr11	61891444	61920635	**INCENP**	+	29191	1.46	1363	705	1583	0.445	-1.167333	0.218	0
chr11	119205236	119208022	**RNF26**	+	2786	4.525	398	205	462	0.445	-1.168478	0.282	0
chr8	8175257	8239257	**SGK223**	-	64000	2.645	5734	2965	6657	0.445	-1.166973	0.339	0
chr2	85360582	85537511	**TCF7L1**	+	176929	1.031	5937	3070	6892	0.445	-1.166584	0.297	0
chr15	50849351	50979012	**TRPM7**	-	129661	1.811	7709	3983	8952	0.445	-1.16835	0.335	0
chr1	249132529	249143714	**ZNF672**	+	11185	1.1	391	202	455	0.445	-1.168998	0.293	0
chr19	1911888	1913446	**ADAT3**	+	1558	1.899	92	48	107	0.446	-1.165885	0.436	0
chr4	126237566	126414087	**FAT4**	+	176521	1.107	6625	3433	7689	0.446	-1.163293	0.356	0
chr12	115108058	115121969	**TBX3**	-	13911	32.554	14519	7519	16852	0.446	-1.164317	0.397	0
chr4	2932287	2936586	**MFSD10**	-	4299	1.715	231	120	268	0.447	-1.160299	0.338	0
chr17	79523912	79604138	**NPLOC4**	-	80226	3.517	9108	4728	10568	0.447	-1.160366	0.339	0
chr1	223889294	223963720	**CAPN2**	+	74426	5.051	12227	6358	14183	0.448	-1.157402	0.388	0
chr1	179923907	180084015	**CEP350**	+	160108	1.346	7161	3719	8308	0.448	-1.159447	0.342	0
chr19	42788816	42799949	**CIC**	+	11133	1.516	533	277	619	0.448	-1.159898	0.26	0
chr18	2916991	3011945	**LPIN2**	-	94954	1.878	5813	3019	6744	0.448	-1.159156	0.301	0
chr12	79985744	80084790	**PAWR**	-	99046	2.113	7086	3689	8218	0.449	-1.155308	0.365	0
chr11	8714898	8932498	**ST5**	-	217600	1.825	12930	6730	14996	0.449	-1.155911	0.4	0
chr12	97301000	97347469	**NEDD1**	+	46469	3.396	5403	2820	6264	0.45	-1.151454	0.353	0
chr3	14444105	14530857	**SLC6A6**	+	86752	4.018	11216	5854	13003	0.45	-1.151261	0.368	0
chr20	33890368	33999945	**UQCC**	-	109577	1.275	4547	2373	5271	0.45	-1.151422	0.281	0
chr7	97910978	97922275	**BRI3**	+	11297	10.444	3718	1945	4309	0.451	-1.147569	0.239	0
chr11	64844926	64851615	**CDCA5**	-	6689	2.468	527	276	611	0.451	-1.147806	0.279	0
chr13	41129800	41240734	**FOXO1**	-	110934	1.013	3816	1994	4423	0.451	-1.149423	0.321	0
chr13	50018428	50069139	**SETDB2**	+	50711	1.114	1830	956	2121	0.451	-1.149428	0.24	0
chr17	411907	618096	**VPS53**	-	206189	1.035	6930	3619	8034	0.451	-1.150385	0.321	0
chr19	43671894	43690688	**PSG5**	-	18794	3.148	1982	1038	2297	0.452	-1.145269	0.279	0
chr6	27775976	27776445	**HIST1H2AI**	+	469	1.561	24	12	28	0.453	-1.143054	0.665	0
chr2	20817563	20850864	**HS1BP3**	-	33301	1.37	1429	749	1655	0.453	-1.143377	0.21	0
chr16	577855	604636	**SOLH**	+	26781	1.003	847	445	981	0.453	-1.141077	0.232	0

chr5	154092461	154197163	**LARP1**	+	104702	1.933	6536	3436	7569	0.454	-1.139075	0.315	0
chr16	88781745	88851372	**PIEZO1**	-	69627	2.128	4696	2470	5438	0.454	-1.138548	0.266	0
chr3	149530474	149679925	**RNF13**	+	149451	1.261	6231	3278	7215	0.454	-1.137797	0.337	0
chr14	103398715	103523742	**CDC42BPB**	-	125027	2.109	8512	4488	9854	0.455	-1.134625	0.344	0
chr8	67474409	67525480	**MYBL1**	-	51071	2.014	3402	1790	3939	0.455	-1.137228	0.289	0
chr9	91003296	91093622	**SPIN1**	+	90326	2.977	8846	4659	10242	0.455	-1.136236	0.369	0
chr13	33078642	33083532	**CG030**	-	4890	4.1	655	345	758	0.456	-1.133889	0.291	0
chr13	53029494	53050763	**CKAP2**	+	21269	3.02	2121	1119	2455	0.456	-1.133712	0.274	0
chr13	32605436	32870776	**FRY**	+	265340	1.112	9924	5242	11485	0.456	-1.131532	0.412	0
chr11	27387507	27494334	**LGR4**	-	106827	1.289	4578	2416	5299	0.456	-1.13315	0.318	0
chr1	173900351	173962210	**RC3H1**	-	61859	1.821	3762	1986	4354	0.456	-1.132215	0.309	0
chr18	43753987	43846955	**C18orf25**	+	92968	1.992	6134	3242	7098	0.457	-1.130681	0.339	0
chr1	180167169	180169859	**FLJ23867**	+	2690	1.017	87	46	101	0.457	-1.129082	0.487	0
chr15	41271078	41408340	**INO80**	-	137262	1.505	6812	3600	7882	0.457	-1.13047	0.345	0
chr15	48431628	48470558	**MYEF2**	-	38930	1.063	1361	720	1574	0.457	-1.128309	0.268	0
chr3	8792094	8811300	**OXTR**	-	19206	28.373	18022	9523	20855	0.457	-1.130802	0.489	0
chr3	145787227	145879282	**PLOD2**	-	92055	3.263	10298	5445	11916	0.457	-1.129851	0.441	0
chr8	26149006	26230195	**PPP2R2A**	+	81189	2.934	7840	4149	9070	0.457	-1.128249	0.359	0
chr4	119643977	119757326	**SEC24D**	-	113349	5.52	20810	10995	24082	0.457	-1.131049	0.522	0
chr9	137533651	137736688	**COL5A1**	+	203037	2.068	13579	7189	15710	0.458	-1.127815	0.415	0
chr3	33038099	33138694	**GLB1**	-	100595	1.056	3439	1820	3979	0.458	-1.127891	0.274	0
chr13	24995068	25086948	**PARP4**	-	91880	1.549	4622	2447	5347	0.458	-1.127462	0.297	0
chr12	121200312	121342155	**SPPL3**	-	141843	1.166	5444	2886	6296	0.458	-1.12502	0.326	0
chr8	41786996	41909505	**KAT6A**	-	122509	1.821	7367	3913	8519	0.459	-1.12224	0.359	0
chr21	26934456	26947480	**MIR155HG**	+	13024	4.336	1850	982	2139	0.459	-1.122292	0.268	0
chr4	37592421	37687999	**RELL1**	-	95578	1.019	3174	1683	3671	0.459	-1.124604	0.277	0
chr6	10396915	10419797	**TFAP2A**	-	22882	13.311	9910	5261	11460	0.459	-1.12326	0.379	0
chr1	210001311	210030910	**DIEXF**	+	29599	3.393	3258	1733	3766	0.46	-1.119635	0.277	0
chr5	15500304	15939900	**FBXL7**	+	439596	1.755	25779	13717	29800	0.46	-1.119385	0.577	0
chr12	13043955	13066600	**GPRC5A**	+	22645	7.299	5411	2877	6255	0.46	-1.120381	0.323	0
chr1	39547088	39952810	**MACF1**	+	405722	1.896	25461	13552	29430	0.46	-1.118782	0.566	0
chr11	85668213	85780923	**PICALM**	-	112710	4.915	18531	9863	21421	0.46	-1.11888	0.515	0
chr21	45285115	45407475	**AGPAT3**	+	122360	1.2	4708	2507	5442	0.461	-1.118206	0.287	0
chr12	105567074	105630008	**APPL2**	-	62934	6.758	14098	7509	16294	0.461	-1.117714	0.457	0
chr2	118673053	118771739	**CCDC93**	-	98686	1.482	4738	2525	5476	0.461	-1.117045	0.301	0
chr11	47487488	47574792	**CELF1**	-	87304	2.995	8525	4539	9853	0.461	-1.11808	0.364	0
chr20	34213952	34236846	**CPNE1**	-	22894	2.631	1949	1038	2253	0.461	-1.117539	0.258	0
chr1	156084460	156109878	**LMNA**	+	25418	20.635	16658	8875	19252	0.461	-1.117199	0.435	0
chr4	39824482	39979576	**PDS5A**	-	155094	1.681	8587	4572	9926	0.461	-1.118302	0.377	0
chr13	33160563	33352158	**PDS5B**	+	191595	1.025	6548	3486	7569	0.461	-1.118363	0.36	0
chr8	38854504	38962779	**ADAM9**	+	108275	4.923	17562	9374	20292	0.462	-1.114096	0.489	0
chr3	5163929	5222601	**ARL8B**	+	58672	3.216	6037	3224	6975	0.462	-1.113005	0.31	0
chr2	32582095	32843965	**BIRC6**	+	261870	2.134	18580	9916	21469	0.462	-1.114339	0.51	0
chr13	45006278	45150701	**TSC22D1**	-	144423	1.599	7579	4049	8756	0.462	-1.112659	0.361	0
chr5	138940750	139008018	**UBE2D2**	+	67268	1.905	4220	2254	4875	0.462	-1.112599	0.313	0
chr15	69096159	69099440	**ANP32A-IT1**	-	3281	1.228	129	69	150	0.463	-1.111645	0.454	0
chr5	171288555	171433877	**FBXW11**	-	145322	2.07	9925	5308	11464	0.463	-1.110911	0.402	0
chr10	98592016	98724198	**LCOR**	+	132182	2.387	10483	5604	12109	0.463	-1.111586	0.417	0
chr5	52856464	52979171	**NDUFS4**	+	122707	1.684	6827	3652	7885	0.463	-1.110224	0.359	0
chr18	46446222	46477081	**SMAD7**	-	30859	7.905	7935	4245	9165	0.463	-1.110219	0.359	0
chr7	5346422	5463177	**TNRC18**	-	116755	1.401	5257	2811	6073	0.463	-1.111085	0.304	0
chr19	39897486	39900045	**ZFP36**	+	2559	49.718	3955	2115	4568	0.463	-1.110335	0.248	0
chr12	70636773	70748773	**CNOT2**	+	112000	2.624	9720	5213	11223	0.464	-1.106316	0.404	0
chr10	79550548	79686348	**DLG5**	-	135800	1.385	6071	3254	7010	0.464	-1.107188	0.325	0
chr3	193853930	193856401	**HES1**	+	2471	3.14	241	129	279	0.464	-1.107588	0.35	0
chr11	3696239	3819022	**NUP98**	-	122783	2.371	9566	5124	11047	0.464	-1.108157	0.395	0
chr3	141205925	141331197	**RASA2**	+	125272	2.496	10259	5498	11845	0.464	-1.107245	0.402	0
chr1	9294862	9331394	**H6PD**	+	36532	1.176	1368	735	1579	0.465	-1.10376	0.246	0
chr16	70557690	70611571	**SF3B3**	+	53881	2.491	4383	2351	5060	0.465	-1.105479	0.31	0
chr12	111843751	111889427	**SH2B3**	+	45676	3.042	4412	2368	5094	0.465	-1.104647	0.279	0
chr20	37590980	37668366	**DHX35**	+	77386	1.312	3323	1787	3835	0.466	-1.101495	0.298	0
chr2	42396489	42559688	**EML4**	+	163199	1.079	5752	3095	6638	0.466	-1.100742	0.338	0
chr7	130628918	130793562	**FLJ43663**	-	164644	3.208	17195	9250	19844	0.466	-1.101167	0.479	0
chr1	198777131	198906558	**LOC100131234**	-	129427	1.748	7538	4055	8699	0.466	-1.101177	0.384	0
chr13	22066827	22178355	**EFHA1**	-	111528	1.263	4681	2524	5400	0.467	-1.097161	0.34	0
chr15	52599479	52821247	**MYO5A**	-	221768	1.172	8575	4621	9894	0.467	-1.098314	0.39	0
chr6	3077057	3115421	**RIPK1**	+	38364	2.177	2717	1464	3134	0.467	-1.098475	0.282	0
chr16	24930711	25026675	**ARHGAP17**	-	95964	2.843	8893	4803	10257	0.468	-1.094618	0.382	0
chr19	10982252	11033448	**CARM1**	+	51196	1.196	1942	1048	2240	0.468	-1.09581	0.247	0
chr10	74451888	74647452	**MCU**	+	195564	1.256	8140	4397	9388	0.468	-1.094207	0.392	0
chr9	136207754	136214972	**MED22**	-	7218	1.514	346	186	399	0.468	-1.095356	0.341	0

chr5	38938021	39074501	**RICTOR**	-	136480	2.145	9658	5215	11139	0.468	-1.094761	0.408	0
chr2	224839764	224904036	**SERPINE2**	-	64272	1.39	2819	1521	3252	0.468	-1.095937	0.253	0
chr17	3907738	4046253	**ZZEF1**	-	138515	1.378	6222	3356	7178	0.468	-1.096412	0.342	0
chr12	52626953	52642709	**KRT7**	+	15756	1.252	673	364	776	0.469	-1.090834	0.365	0
chr7	151163097	151217010	**RHEB**	-	53913	3.714	6554	3547	7556	0.469	-1.090974	0.356	0
chr15	68346571	68480404	**PIAS1**	+	133833	2.28	10100	5472	11643	0.47	-1.089167	0.422	0
chr10	88516395	88684945	**BMPR1A**	+	168550	1.543	8657	4699	9977	0.471	-1.086189	0.41	0
chr4	1205227	1242908	**CTBP1**	-	37681	1.975	2353	1277	2712	0.471	-1.086517	0.254	0
chr7	157129709	157210133	**DNAJB6**	+	80424	2.877	7481	4061	8621	0.471	-1.0861	0.359	0
chr11	126293395	126870766	**KIRREL3**	-	577371	1.67	32094	17434	36980	0.471	-1.084845	0.641	0
chr5	43602790	43705668	**NNT**	+	102878	1.091	3659	1987	4217	0.471	-1.085442	0.309	0
chr1	179068461	179198819	**ABL2**	-	130358	2.82	12016	6539	13842	0.472	-1.081916	0.435	0
chr3	130569368	130735555	**ATP2C1**	+	166187	2.385	13193	7171	15200	0.472	-1.083678	0.469	0
chr20	40031169	40247133	**CHD6**	-	215964	1.475	10485	5698	12080	0.472	-1.084045	0.424	0
chr2	241375114	241407495	**GPC1**	+	32381	1.516	1558	847	1795	0.472	-1.08324	0.254	0
chrX	48779302	48815648	**OTUD5**	-	36346	1.181	1380	751	1590	0.472	-1.081882	0.269	0
chr12	132195634	132284282	**SFSWAP**	+	88648	2.087	6012	3270	6926	0.472	-1.082693	0.345	0
chr3	124944512	125094198	**ZNF148**	-	149686	2.17	10827	5887	12473	0.472	-1.08327	0.443	0
chr19	926036	972803	**ARID3A**	+	46767	1.295	1918	1044	2209	0.473	-1.080558	0.255	0
chr2	153574406	153617767	**ARL6IP6**	+	43361	1.193	1683	916	1939	0.473	-1.080673	0.282	0
chr5	52285155	52390609	**ITGA2**	+	105454	1.806	6247	3404	7194	0.473	-1.079684	0.36	0
chr10	90973325	91011660	**LIPA**	-	38335	2.538	3161	1723	3641	0.473	-1.078634	0.301	0
chr2	128056244	128100805	**MAP3K2**	-	44561	1.258	1891	1030	2178	0.473	-1.080608	0.329	0
chr2	162164785	162268226	**PSMD14**	+	103441	1.922	6553	3573	7546	0.473	-1.078688	0.373	0
chr1	193091087	193223942	**CDC73**	+	132855	2.294	10051	5488	11573	0.474	-1.076326	0.425	0
chr12	122755980	122907116	**CLIP1**	-	151136	1.724	8586	4682	9887	0.474	-1.07851	0.402	0
chr20	42740336	42816218	**JPH2**	-	75882	1.082	2704	1475	3114	0.474	-1.078183	0.309	0
chr6	86159301	86205509	**NT5E**	+	46208	3.958	5963	3255	6865	0.474	-1.076432	0.35	0
chr11	3108345	3186582	**OSBPL5**	-	78237	1.037	2591	1414	2983	0.474	-1.077072	0.274	0
chr5	133861797	133918918	**PHF15**	+	57121	1.276	2315	1264	2665	0.474	-1.07621	0.264	0
chr22	22273791	22307250	**PPM1F**	-	33459	1.2	1280	699	1473	0.474	-1.076263	0.264	0
chr8	61429468	61536203	**RAB2A**	+	106735	4.451	15683	8559	18058	0.474	-1.077025	0.495	0
chr18	47901391	47920538	**SKA1**	+	19147	1.089	678	370	780	0.474	-1.075557	0.319	0
chr22	26921713	26986089	**TPST2**	-	64376	1.497	3133	1709	3608	0.474	-1.078023	0.301	0
chr21	30671219	30734217	**BACH1**	+	62998	5.858	12148	6642	13983	0.475	-1.073871	0.452	0
chr11	65543377	65547822	**DKFZp761E198**	-	4445	3.271	457	250	526	0.475	-1.073134	0.319	0
chr2	68405988	68479651	**PPP3R1**	-	73663	3.317	8094	4429	9316	0.475	-1.072787	0.404	0
chr9	2015341	2193623	**SMARCA2**	+	178282	1.152	6786	3712	7811	0.475	-1.073423	0.38	0
chr14	31363004	31495607	**STRN3**	-	132603	2.029	8890	4862	10233	0.475	-1.073541	0.412	0
chr15	70946892	71055850	**UACA**	-	108958	5.137	18477	10094	21272	0.475	-1.075461	0.525	0
chr2	181845111	181928150	**UBE2E3**	+	83039	3.56	9697	5305	11161	0.475	-1.072908	0.416	0
chr2	175424301	175547627	**WIPF1**	-	123326	1.774	7265	3971	8363	0.475	-1.074525	0.394	0
chr21	43406939	43430496	**ZNF295**	-	23557	1.979	1494	817	1720	0.475	-1.073484	0.27	0
chr7	45039344	45116069	**CCM2**	+	76725	1.297	3215	1759	3700	0.476	-1.072213	0.299	0
chr5	80715671	81047072	**SSBP2**	-	331401	1.162	12974	7106	14930	0.476	-1.071009	0.493	0
chr7	115850546	115898837	**TES**	+	48291	2.844	4669	2558	5372	0.476	-1.070387	0.383	0
chr7	98476112	98610866	**TRRAP**	+	134754	1.371	5993	3284	6896	0.476	-1.070296	0.349	0
chrX	108884563	108976621	**ACSL4**	-	92058	1.855	5621	3090	6464	0.478	-1.06488	0.364	0
chr17	33914281	34053436	**AP2B1**	+	139155	1.923	8793	4837	10112	0.478	-1.063823	0.41	0
chr8	142138719	142205900	**DENND3**	+	67181	4.05	8671	4762	9973	0.478	-1.066344	0.372	0
chr12	96588206	96661606	**ELK3**	+	73400	1.635	3925	2157	4515	0.478	-1.065642	0.33	0
chr12	65563350	65642141	**LEMD3**	+	78791	1.204	3120	1715	3588	0.478	-1.064969	0.324	0
chr7	100026412	100031749	**MEPCE**	+	5337	3.416	576	317	663	0.478	-1.063459	0.308	0
chr20	49126890	49201086	**PTPN1**	+	74196	3.241	7807	4290	8979	0.478	-1.065592	0.38	0
chr1	78030189	78148343	**ZZZ3**	-	118154	3.389	13172	7238	15150	0.478	-1.065498	0.468	0
chr5	102465256	102538909	**PPIP5K2**	+	73653	1.331	3242	1785	3727	0.479	-1.061861	0.335	0
chr1	31404352	31538564	**PUM1**	-	134212	2.85	12600	6935	14489	0.479	-1.062951	0.466	0
chr12	3068477	3149842	**TEAD4**	+	81365	1.432	3758	2068	4322	0.479	-1.063006	0.311	0
chr1	35734567	35887545	**ZMYM4**	+	152978	1.481	7454	4106	8570	0.479	-1.0615	0.394	0
chr6	75794041	75915623	**COL12A1**	-	121582	8.97	36710	20238	42200	0.48	-1.060145	0.689	0
chr3	14989235	15090780	**NR2C2**	+	101545	1.323	4412	2433	5071	0.48	-1.059591	0.345	0
chr7	155437202	155574179	**RBM33**	+	136977	2.329	10430	5756	11987	0.48	-1.058234	0.431	0
chr7	102781716	102782850	**RPL19P12**	-	1134	2.555	91	50	105	0.48	-1.059141	0.5	0
chr1	155305051	155532324	**ASH1L**	-	227273	1.313	9942	5494	11425	0.481	-1.056248	0.45	0
chr6	56322784	56507694	**DST**	-	184910	6.833	41902	23173	48145	0.481	-1.054966	0.707	0
chr7	44421964	44530385	**NUDCD3**	-	108421	1.416	4951	2738	5689	0.481	-1.054886	0.337	0
chr12	42719946	42842422	**PPHLN1**	+	122476	1.323	5306	2932	6097	0.481	-1.056103	0.358	0
chr17	74380689	74383941	**SPHK1**	+	3252	8.736	891	493	1024	0.481	-1.055186	0.272	0
chr12	114791734	114846247	**TBX5**	-	54513	4.968	8882	4910	10206	0.481	-1.055567	0.415	0
chr11	101981191	102104154	**YAP1**	+	122963	3.482	14088	7792	16187	0.481	-1.054799	0.486	0
chr6	53132195	53213977	**ELOVL5**	-	81782	3.385	8990	4976	10327	0.482	-1.053399	0.406	0

chr3	194406621	194409766	**FAM43A**	+	3145	12.743	1243	687	1428	0.482	-1.054108	0.246	0
chr9	132250938	132275965	**LOC100506190**	+	25027	1.009	808	447	928	0.482	-1.052867	0.301	0
chr11	74459912	74553458	**RNF169**	+	93546	1.593	4908	2716	5639	0.482	-1.053997	0.359	0
chr1	36805224	36851485	**STK40**	-	46261	1.633	2422	1341	2782	0.482	-1.052697	0.289	0
chr3	46963219	47018270	**CCDC12**	-	55051	2.955	5249	2910	6029	0.483	-1.050909	0.343	0
chr2	31456879	31491260	**EHD3**	+	34381	1.032	1123	623	1290	0.483	-1.04942	0.275	0
chr8	143738873	143751401	**JRK**	-	12528	3.168	1256	697	1443	0.483	-1.049668	0.272	0
chr9	127420714	127460907	**MIR181A2HG**	+	40193	2.899	3946	2187	4533	0.483	-1.050896	0.38	0
chr17	17714662	17740325	**SREBF1**	-	25663	3.095	2492	1382	2862	0.483	-1.049688	0.269	0
chr6	37225547	37300746	**TBC1D22B**	+	75199	1.126	2778	1541	3191	0.483	-1.050082	0.325	0
chr10	75757871	75879914	**VCL**	+	122043	5.991	24062	13342	27636	0.483	-1.050555	0.583	0
chr8	54628114	54755850	**ATP6V1H**	-	127736	1.669	6908	3839	7931	0.484	-1.04685	0.376	0
chr2	149402559	149545136	**EPC2**	+	142577	1.94	9067	5039	10410	0.484	-1.046545	0.423	0
chr18	9546791	9614600	**PPP4R1**	-	67809	3.377	7539	4187	8656	0.484	-1.047754	0.404	0
chr1	167905796	168045083	**DCAF6**	+	139287	1.993	9246	5148	10612	0.485	-1.043467	0.448	0
chr1	184760165	184943682	**FAM129A**	-	183517	2.36	14383	8004	16510	0.485	-1.044432	0.507	0
chr5	147763545	147822399	**FBXO38**	+	58854	2.042	3949	2198	4533	0.485	-1.044336	0.347	0
chr1	182992594	183114727	**LAMC1**	+	122133	4.967	19924	11096	22867	0.485	-1.043164	0.552	0
chr10	89623194	89728532	**PTEN**	+	105338	3.437	11999	6679	13772	0.485	-1.043925	0.475	0
chr4	106067031	106200960	**TET2**	+	133929	1.341	5980	3329	6864	0.485	-1.04385	0.398	0
chr9	111777414	111882225	**C9orf5**	-	104811	1.872	6446	3594	7396	0.486	-1.041054	0.388	0
chr6	27858092	27858570	**HIST1H3J**	-	478	3.627	55	30	63	0.486	-1.041598	0.595	0
chr2	11886739	11967533	**LPIN1**	+	80794	1.57	4041	2253	4637	0.486	-1.04152	0.315	0
chr5	71403117	71505397	**MAP1B**	+	102280	6.247	21075	11755	24182	0.486	-1.040625	0.571	0
chr4	120133781	120216673	**USP53**	+	82892	7.478	20440	11390	23457	0.486	-1.042251	0.563	0
chr2	238232654	238322850	**COL6A3**	-	90196	10.679	30622	17114	35125	0.487	-1.037296	0.605	0
chr3	155588324	155655520	**GMPS**	+	67196	1.805	3969	2217	4552	0.487	-1.037506	0.347	0
chr9	6413150	6507051	**UHRF2**	+	93901	2.231	6899	3855	7914	0.487	-1.037693	0.402	0
chr11	58346586	58389023	**ZFP91**	+	42437	5.099	7041	3935	8076	0.487	-1.037335	0.389	0
chr6	106632351	106773695	**ATG5**	-	141344	1.618	7588	4249	8701	0.488	-1.033825	0.425	0
chr15	91073197	91188577	**CRTC3**	+	115380	1.379	5206	2916	5969	0.488	-1.033644	0.369	0
chr2	233562014	233725289	**GIGYF2**	+	163275	1.633	8745	4893	10030	0.488	-1.035523	0.426	0
chr3	172348434	172429008	**NCEH1**	-	80574	2.729	7146	3999	8195	0.488	-1.035087	0.391	0
chr16	50186828	50269219	**PAPD5**	+	82391	1.597	4331	2424	4967	0.488	-1.034727	0.36	0
chr16	53088944	53361414	**CHD9**	+	272470	1.827	16405	9204	18805	0.489	-1.030729	0.53	0
chr8	105501458	105601252	**LRP12**	-	99794	1.885	6164	3459	7066	0.489	-1.03064	0.388	0
chr11	65837823	66012218	**PACS1**	+	174395	1.204	6905	3870	7917	0.489	-1.032456	0.404	0
chr5	102201526	102366808	**PAM**	+	165282	3.369	18507	10374	21219	0.489	-1.032302	0.561	0
chr9	127115743	127177721	**PSMB7**	-	61978	3.512	7042	3946	8074	0.489	-1.032938	0.384	0
chr2	17845078	17935096	**SMC6**	-	90018	1.055	3125	1753	3582	0.489	-1.031177	0.345	0
chr13	27131839	27263082	**WASF3**	+	131243	1.002	4315	2418	4947	0.489	-1.032622	0.358	0
chr4	99799606	99851786	**EIF4E**	-	52180	2.495	4268	2397	4892	0.49	-1.029172	0.359	0
chr14	31028328	31089046	**G2E3**	+	60718	2.654	5296	2977	6069	0.49	-1.027699	0.38	0
chr4	3076407	3245687	**HTT**	+	169280	1.227	6783	3808	7775	0.49	-1.02955	0.395	0
chr2	32092893	32235698	**MEMO1**	-	142805	1.569	7393	4152	8473	0.49	-1.028844	0.418	0
chr8	103264501	103424917	**UBR5**	-	160416	2.662	14167	7966	16234	0.491	-1.027113	0.516	0
chr7	149244244	149321881	**ZNF767**	-	77637	1.426	3573	2010	4094	0.491	-1.026281	0.333	0
chr2	239335625	239360891	**ASB1**	+	25266	3.835	3107	1749	3559	0.492	-1.024569	0.318	0
chr18	77439800	77514510	**CTDP1**	+	74710	1.122	2679	1510	3069	0.492	-1.022874	0.308	0
chr10	104847773	104953063	**NT5C2**	-	105290	2.859	10045	5659	11507	0.492	-1.023765	0.473	0
chr11	59522531	59573355	**STX3**	+	50824	3.588	5800	3266	6645	0.492	-1.024711	0.35	0
chr5	68462836	68474070	**CCNB1**	+	11234	3.98	1460	824	1672	0.493	-1.020701	0.325	0
chr10	76586378	76792639	**KAT6B**	+	206261	1.732	11890	6714	13616	0.493	-1.01988	0.498	0
chr2	178257470	178408564	**AGPS**	+	151094	1.059	5323	3011	6094	0.494	-1.017189	0.402	0
chr15	77713242	77777945	**HMG20A**	+	64703	1.155	2482	1404	2842	0.494	-1.016571	0.356	0
chr1	35899090	36023037	**KIAA0319L**	-	123947	1.028	4179	2362	4784	0.494	-1.018381	0.365	0
chr9	5629118	5776556	**KIAA1432**	+	147438	3.036	14952	8451	17119	0.494	-1.018306	0.539	0
chr5	6714717	6757161	**PAPD7**	+	42444	3.315	4512	2549	5166	0.494	-1.018686	0.342	0
chr7	66205642	66276448	**RABGEF1**	+	70806	2.504	5769	3262	6604	0.494	-1.017414	0.38	0
chr14	99864082	99947226	**SETD3**	-	83144	1.018	2759	1560	3158	0.494	-1.016872	0.337	0
chr4	10075962	10118573	**WDR1**	-	42611	11.654	15892	8991	18192	0.494	-1.016792	0.497	0
chr18	9136750	9285983	**ANKRD12**	+	149233	3.016	14773	8371	16907	0.495	-1.014141	0.517	0
chr1	220321609	220445843	**RAB3GAP2**	-	124234	1.657	6758	3830	7734	0.495	-1.013783	0.412	0
chr10	102106771	102124588	**SCD**	+	17817	3.932	2165	1227	2478	0.495	-1.013749	0.269	0
chr14	103243815	103377837	**TRAF3**	+	134022	1.038	4530	2567	5185	0.495	-1.013808	0.365	0
chr9	139971952	139978990	**UAP1L1**	+	7038	4.656	1022	578	1170	0.495	-1.015284	0.279	0
chr12	19592607	19675173	**AEBP2**	+	82566	1.356	3677	2086	4207	0.496	-1.011662	0.362	0
chr6	170615843	170714237	**FAM120B**	+	98394	2.011	6421	3642	7347	0.496	-1.012322	0.391	0
chr14	73741917	73925286	**NUMB**	-	183369	1.378	8303	4713	9500	0.496	-1.011298	0.436	0
chr13	111293756	111358480	**CARS2**	-	64724	2.792	5745	3267	6570	0.497	-1.007743	0.359	0
chr2	135809834	135928279	**RAB3GAP1**	+	118445	2.759	10830	6159	12387	0.497	-1.007892	0.486	0

chr22	22311402	22337147	**TOP3B**	-	25745	1.35	1102	626	1261	0.497	-1.008646	0.301	0
chr17	76792964	76836969	**USP36**	-	44005	3.898	5470	3109	6258	0.497	-1.009236	0.358	0
chr6	87865268	87973406	**ZNF292**	+	108138	2.642	9368	5327	10714	0.497	-1.007948	0.454	0
chr17	80200536	80231594	**CSNK1D**	-	31058	6.309	6279	3574	7181	0.498	-1.006364	0.379	0
chr19	3506294	3536755	**FZR1**	+	30461	2.05	1973	1123	2256	0.498	-1.005857	0.296	0
chr5	65018022	65125111	**NLN**	+	107089	1.101	3926	2236	4489	0.498	-1.005054	0.388	0
chr9	77703397	77762114	**OSTF1**	+	58717	1.057	2024	1153	2314	0.498	-1.004562	0.337	0
chr11	82692477	82782884	**RAB30**	-	90407	1.364	4056	2309	4639	0.498	-1.006187	0.375	0
chr2	114647536	114716167	**ACTR3**	+	68631	9.867	22389	12773	25595	0.499	-1.002711	0.606	0
chr1	200708685	200829831	**CAMSAP2**	+	121146	2	8005	4569	9151	0.499	-1.001823	0.447	0
chr11	120894802	120960354	**TBCEL**	+	65552	1.162	2493	1422	2850	0.499	-1.002441	0.347	0
chr9	125703287	125867147	**RABGAP1**	+	163860	1.383	7448	4259	8511	0.5	-0.998771	0.434	0
chr1	112162404	112256101	**RAP1A**	+	93697	2.66	8210	4693	9382	0.5	-0.999415	0.448	0
**sum**					**1.6E+08**								
**average size**					**136355**								
