# Supplementary material for: Genome-Wide Transcriptional Effects of the Anti-Cancer Agent Camptothecin
Source: PLoS One. 2013 Oct 23;8(10):e78190. doi: 10.1371/journal.pone.0078190 (PMC3806802; doi:10.1371/journal.pone.0078190)
Supplement: Table S3 — Up and down regulation of genes following CPT treatment and 15 min. recovery. (PDF) [file pone.0078190.s004.pdf]

Table S3. Up and down regulation of genes following CPT treatment and 15 min recovery.

Pages 1-6: Table S3A - UP-regulated genes
Pages 7-19: Table S3B - DOWN-regulated genes

Table S3A. Genes UP-regulated >2-fold following 45 min CPT treatment,wash and 15 min Bru (CPT+15m).

chrom	start	end	name	strand	bp	meanRPKM	meanCount	1_CPT15m	2_cont	foldChange	log2FoldCha	pAdjusted	significant
chrX	53111541	53117728	**TSPYL2**	+	6187	4.09	696	1973	270	7.285	2.865	0	1
chr17	72858618	72869156	**FDXR**	-	10538	2.349	689	1855	300	6.17	2.625	0	1
chr19	2037469	2051243	**MKNK2**	-	13774	2.518	963	2578	424	6.074	2.603	0	1
chr1	110009099	110024764	**SYPL2**	+	15665	1.894	852	2030	459	4.423	2.145	0.002	1
chr1	89517986	89531043	**GBP1**	-	13057	1.148	434	1019	239	4.259	2.09	0.006	1
chr13	77566058	77576652	**CLN5**	+	10594	1.871	575	1337	321	4.161	2.057	0.005	1
chr2	36581891	36582713	**LOC100288911**	-	822	2.941	69	159	39	4.005	2.002	0.053	1
chr15	44092618	44094769	**C15orf63**	+	2151	3.134	196	446	113	3.922	1.972	0.022	1
chrX	135228860	135293518	**FHL1**	+	64658	2.604	4993	11184	2930	3.817	1.932	0.006	1
chrX	20154183	20154531	**SCARNA9L**	-	348	1.141	11	26	7	3.78	1.918	0.151	0
chr6	16129316	16148478	**MYLIP**	+	19162	1.612	894	1983	531	3.73	1.899	0.01	1
chr8	57124314	57131176	**CHCHD7**	+	6862	2.98	600	1325	358	3.698	1.887	0.011	1
chr12	77252495	77272799	**CSRP2**	-	20304	2.798	1674	3696	1000	3.695	1.886	0.006	1
chr1	192778168	192781407	**RGS2**	+	3239	3.231	306	669	185	3.606	1.85	0.026	1
chr18	47007547	47013644	**C18orf32**	-	6097	2.367	427	923	261	3.528	1.819	0.019	1
chr1	8071778	8086393	**ERRFI1**	-	14615	2.453	1057	2248	661	3.402	1.766	0.014	1
chr7	142960521	142966222	**GSTK1**	+	5701	1.828	308	651	193	3.364	1.75	0.036	1
chr9	91605777	91611057	**C9orf47**	+	5280	2.324	364	763	231	3.3	1.722	0.033	1
chr6	121756744	121770873	**GJA1**	+	14129	2.046	864	1807	549	3.29	1.718	0.016	1
chr12	6571403	6579843	**VAMP1**	-	8440	3.47	868	1815	552	3.286	1.717	0.019	1
chr20	10618331	10654694	**JAG1**	-	36363	1.71	1845	3835	1182	3.245	1.698	0.016	1
chr14	80663867	80697397	**DIO2**	-	33530	1.472	1466	3047	939	3.243	1.697	0.016	1
chr12	53342654	53346685	**KRT18**	+	4031	2.1	255	528	164	3.209	1.682	0.04	1
chr6	12290528	12297427	**EDN1**	+	6899	1.064	223	461	144	3.197	1.677	0.039	1
chr14	31915242	31926680	**C14orf126**	-	11438	1.129	388	798	251	3.171	1.665	0.034	1
chr10	95653729	95662491	**SLC35G1**	+	8762	1.233	323	662	210	3.153	1.657	0.044	1
chr17	7123152	7128585	**ACADVL**	+	5433	5.632	915	1859	600	3.096	1.63	0.024	1
chr15	65204100	65251041	**ANKDD1A**	+	46941	1.258	1758	3565	1155	3.085	1.625	0.023	1
chr19	50162825	50169132	**IRF3**	-	6307	1.362	253	513	166	3.077	1.622	0.074	1
chr4	140374960	140397069	**RAB33B**	+	22109	1.458	962	1944	635	3.062	1.615	0.027	1
chr1	203274663	203278729	**BTG2**	+	4066	6.844	819	1653	541	3.051	1.609	0.037	1
chr6	138409641	138428660	**PERP**	-	19019	1.873	1066	2149	704	3.05	1.609	0.025	1
chr6	71998476	72011973	**OGFRL1**	+	13497	3.513	1431	2881	947	3.04	1.604	0.02	1
chr1	47073386	47082563	**MOB3C**	-	9177	2.078	569	1142	378	3.015	1.592	0.04	1
chr9	35732316	35737005	**CREB3**	+	4689	2.767	387	776	258	3.004	1.587	0.054	1
chr14	24683142	24685276	**MDP1**	-	2134	1.059	67	135	45	2.995	1.582	0.141	0
chr6	72124148	72130448	**LINC00472**	-	6300	4.145	782	1563	522	2.992	1.581	0.033	1
chr5	92919042	92929786	**NR2F1**	+	10744	2.092	671	1327	453	2.93	1.551	0.045	1
chr17	73772514	73775860	**H3F3B**	-	3346	43.289	4355	8554	2955	2.894	1.533	0.041	1
chr6	21593971	21598849	**SOX4**	+	4878	1.336	196	386	133	2.894	1.533	0.089	1
chr6	99968869	99981059	**LOC100130890**	+	12190	1.592	587	1154	399	2.893	1.533	0.042	1
chr11	66432469	66445275	**RBM4B**	-	12806	1.068	412	809	280	2.887	1.53	0.059	1
chr10	60094738	60130513	**UBE2D1**	+	35775	1.336	1442	2828	979	2.886	1.529	0.031	1
chr2	220094478	220101391	**ANKZF1**	+	6913	1.449	300	586	204	2.867	1.519	0.082	1
chr6	97337186	97345767	**NDUFAF4**	-	8581	1.102	286	559	195	2.863	1.517	0.073	1
chr19	12460184	12476475	**ZNF442**	-	16291	1.054	524	1021	358	2.847	1.509	0.045	1
chr6	52535883	52551385	**TMEM14A**	+	15502	1.168	549	1067	377	2.832	1.502	0.048	1
chr5	43042235	43045370	**LOC153684**	+	3135	2.007	188	366	129	2.825	1.498	0.112	0
chr14	103592663	103603776	**TNFAIP2**	+	11113	1.52	505	976	348	2.8	1.485	0.069	1
chr17	6544221	6547861	**TXNDC17**	+	3640	1.527	169	326	116	2.791	1.481	0.108	0
chr6	17600517	17611950	**FAM8A1**	+	11433	1.021	352	680	243	2.789	1.48	0.079	1
chr8	95938199	95961615	**TP53INP1**	-	23416	4.53	3188	6137	2205	2.782	1.476	0.047	1
chr19	47724078	47736023	**BBC3**	-	11945	3.203	1141	2197	789	2.782	1.476	0.052	1
chr2	176972083	176974316	**HOXD11**	+	2233	1.823	122	235	84	2.778	1.474	0.142	0
chrX	37698088	37706889	**DYNLT3**	-	8801	1.161	312	599	217	2.762	1.466	0.076	1
chr1	110276553	110283660	**GSTM3**	-	7107	1.714	371	712	257	2.762	1.466	0.071	1
chr12	54393876	54397120	**HOXC9**	+	3244	1.108	108	208	75	2.759	1.464	0.15	0
chr4	17578926	17609590	**LAP3**	+	30664	1.329	1222	2336	851	2.744	1.456	0.051	1
chr6	116832807	116839709	**FAM26E**	+	6902	1.503	317	606	221	2.743	1.456	0.078	1

chr10	30722949	30750762	**MAP3K8**	+	27813	1.046	877	1674	611	2.74	1.454	0.054	1
chr12	54104901	54121307	**CALCOCO1**	-	16406	1.139	567	1082	395	2.74	1.454	0.061	1
chr11	10772810	10801290	**CTR9**	+	28480	1.874	1628	3102	1137	2.728	1.448	0.039	1
chr19	48281841	48287943	**SEPW1**	+	6102	2.823	518	988	362	2.726	1.447	0.076	1
chr9	79000432	79009444	**RFK**	-	9012	1.101	300	571	210	2.722	1.445	0.098	1
chr5	72848024	72861511	**ANKRA2**	-	13487	1.132	467	889	327	2.72	1.444	0.062	1
chr5	53751430	53752214	**HSPB3**	+	784	2.9	68	129	47	2.717	1.442	0.204	0
chr12	49490922	49504680	**LMBR1L**	-	13758	1.085	451	857	315	2.715	1.441	0.08	1
chr1	231154703	231175995	**FAM89A**	-	21292	1.191	765	1452	537	2.704	1.435	0.061	1
chr11	34460471	34493607	**CAT**	+	33136	1.085	1096	2076	769	2.699	1.433	0.046	1
chr10	90694830	90751147	**ACTA2**	-	56317	2.135	3720	7047	2611	2.699	1.432	0.04	1
chr13	108859791	108870716	**LIG4**	-	10925	1.08	360	681	253	2.688	1.427	0.083	1
chr1	115110180	115124265	**BCAS2**	-	14085	1.379	593	1120	417	2.685	1.425	0.062	1
chr5	131817300	131826465	**IRF1**	-	9165	2.448	674	1272	475	2.677	1.421	0.076	1
chr11	62432778	62434923	**METTL12**	+	2145	2.359	153	288	107	2.675	1.419	0.147	0
chr10	133781203	133795435	**BNIP3**	-	14232	2.393	1029	1937	726	2.668	1.416	0.059	1
chr11	62559597	62572964	**NXF1**	-	13367	4.142	1668	3140	1178	2.666	1.415	0.058	1
chr17	78109012	78120982	**EIF4A3**	-	11970	4.313	1563	2942	1104	2.665	1.414	0.054	1
chr21	28208605	28217728	**ADAMTS1**	-	9123	86.381	23617	44312	16718	2.651	1.406	0.207	0
chr10	112257624	112271302	**DUSP5**	+	13678	8.38	3422	6418	2423	2.648	1.405	0.079	1
chr5	141018868	141030986	**FCHSD1**	-	12118	2.719	998	1872	707	2.646	1.404	0.06	1
chr5	156565450	156569921	**MED7**	-	4471	1.6	219	411	155	2.638	1.399	0.112	0
chr20	54933982	54943718	**FAM210B**	+	9736	1.202	354	658	253	2.602	1.379	0.115	0
chr1	85109389	85156240	**SSX2IP**	-	46851	2.956	4253	7899	3038	2.6	1.379	0.062	1
chr6	44225902	44233525	**NFKBIE**	-	7623	1.273	291	541	208	2.598	1.377	0.14	0
chr6	52529198	52533951	**LOC730101**	+	4753	3.023	439	812	315	2.578	1.366	0.096	1
chr3	4344987	4358949	**SETMAR**	+	13962	1.24	527	975	378	2.578	1.366	0.09	1
chrX	153686622	153701985	**PLXNA3**	+	15363	1.467	675	1247	484	2.576	1.365	0.102	0
chr1	33402049	33430286	**RNF19B**	-	28237	1.463	1254	2316	900	2.572	1.363	0.067	1
chr13	27844463	27847827	**RASL11A**	+	3364	1.89	192	352	138	2.539	1.344	0.17	0
chr4	89181531	89205888	**PPM1K**	-	24357	1.101	822	1504	594	2.529	1.339	0.076	1
chr17	11880761	11900689	**ZNF18**	-	19928	1.047	636	1162	460	2.525	1.336	0.093	1
chr9	130922538	130926207	**C9orf16**	+	3669	1.145	125	229	90	2.523	1.335	0.23	0
chr9	33025208	33039062	**DNAJA1**	+	13854	5.184	2201	4013	1597	2.513	1.329	0.069	1
chr10	64893006	64914786	**NRBF2**	+	21780	1.994	1336	2435	969	2.512	1.329	0.065	1
chr5	162930230	162946328	**MAT2B**	+	16098	1.866	925	1685	671	2.509	1.327	0.071	1
chr16	56716381	56718108	**MT1X**	+	1727	1.145	59	108	43	2.506	1.325	0.264	0
chr17	6915735	6917463	**RNASEK**	+	1728	2.109	110	200	80	2.501	1.323	0.218	0
chr19	35225479	35233774	**ZNF181**	+	8295	1.2	303	552	220	2.5	1.322	0.14	0
chr12	107371068	107380929	**MTERFD3**	-	9861	1.714	523	952	380	2.5	1.322	0.087	1
chr14	61438166	61447782	**TRMT5**	-	9616	1.196	355	645	258	2.5	1.322	0.112	0
chr7	30052883	30066268	**FKBP14**	-	13385	1.091	449	816	327	2.497	1.32	0.105	0
chr13	76099349	76111991	**COMMD6**	-	12642	1.738	674	1225	491	2.494	1.319	0.088	1
chr15	45694518	45713616	**SPATA5L1**	+	19098	1.356	791	1436	576	2.493	1.318	0.088	1
chr17	10600926	10614875	**C17orf48**	+	13949	3.266	1417	2571	1033	2.49	1.316	0.058	1
chr17	43224683	43229468	**HEXIM1**	+	4785	3.775	549	996	400	2.485	1.313	0.113	0
chr17	27071022	27077976	**TRAF4**	+	6954	6.277	1317	2385	960	2.482	1.312	0.095	1
chr11	9481102	9482245	**LOC644656**	-	1143	4.294	150	271	109	2.481	1.311	0.189	0
chr16	50059188	50070999	**TMEM188**	+	11811	1.735	628	1137	458	2.479	1.31	0.098	1
chr19	58011308	58019510	**ZNF773**	+	8202	1.217	303	549	221	2.478	1.309	0.15	0
chr5	33987090	34008220	**AMACR**	-	21130	1.989	1293	2339	945	2.476	1.308	0.072	1
chr18	3262110	3278282	**MYL12B**	+	16172	8.675	4348	7856	3178	2.471	1.305	0.079	1
chr4	110736665	110745893	**GAR1**	+	9228	1.764	502	908	367	2.47	1.305	0.102	0
chr6	151773421	151791232	**C6orf211**	+	17811	1.408	771	1390	565	2.456	1.297	0.089	1
chr10	45471708	45474330	**C10orf10**	-	2622	2.86	226	408	166	2.454	1.295	0.186	0
chr14	20915206	20923267	**OSGEP**	-	8061	1.527	375	675	275	2.449	1.292	0.141	0
chr14	45366506	45376460	**C14orf28**	+	9954	1.6	487	875	357	2.446	1.291	0.123	0
chr17	40009798	40021629	**KLHL11**	-	11831	1.066	385	690	283	2.436	1.284	0.141	0
chr1	145507556	145513535	**RBM8A**	+	5979	1.84	337	604	248	2.432	1.282	0.145	0
chr2	3501689	3523350	**ADI1**	-	21661	2.787	1852	3315	1364	2.43	1.281	0.085	1
chr18	57567191	57571538	**PMAIP1**	+	4347	3.604	473	847	349	2.428	1.28	0.149	0
chr8	126442562	126450644	**TRIB1**	+	8082	10.307	2546	4541	1881	2.413	1.271	0.098	1
chr6	31926580	31937532	**SKIV2L**	+	10952	1.583	526	939	389	2.413	1.271	0.14	0
chr2	62423261	62451866	**B3GNT2**	+	28605	1.687	1469	2619	1086	2.41	1.269	0.101	0
chr8	38846326	38854041	**TM2D2**	-	7715	2.297	545	971	403	2.409	1.269	0.118	0
chr10	102033712	102046439	**BLOC1S2**	-	12727	2.743	1078	1920	798	2.405	1.266	0.085	1
chr7	5632435	5646287	**FSCN1**	+	13852	3.874	1628	2897	1205	2.404	1.265	0.107	0
chr15	90627211	90645708	**IDH2**	-	18497	1.603	908	1615	673	2.398	1.262	0.105	0
chr1	154521050	154531120	**UBE2Q1**	-	10070	2.802	868	1538	644	2.387	1.255	0.104	0
chr13	45967453	45992516	**SLC25A30**	-	25063	1.444	1112	1969	826	2.382	1.252	0.101	0
chr11	125439297	125454575	**EI24**	+	15278	1.992	932	1649	692	2.381	1.251	0.108	0

chr12	25348149	25357949	**LYRM5**	+	9800	1.15	348	616	259	2.375	1.248	0.148	0
chr17	7387697	7417935	**POLR2A**	+	30238	7.876	7283	12869	5421	2.374	1.247	0.149	0
chr17	56160779	56167618	**DYNLL2**	+	6839	4.653	975	1721	726	2.371	1.246	0.108	0
chr20	3648619	3662738	**ADAM33**	-	14119	1.316	566	999	421	2.369	1.244	0.143	0
chr16	81069457	81080951	**ATMIN**	+	11494	1.433	504	890	376	2.367	1.243	0.142	0
chrX	70521627	70525204	**ITGB1BP2**	+	3577	1.174	130	229	97	2.364	1.241	0.218	0
chr17	77806954	77813213	**CBX4**	-	6259	2.175	413	728	308	2.363	1.241	0.171	0
chr5	137673223	137685418	**FAM53C**	+	12195	3.802	1414	2490	1055	2.361	1.239	0.111	0
chr17	72744838	72765499	**SLC9A3R1**	+	20661	1.209	754	1327	563	2.358	1.237	0.148	0
chr21	30428647	30446010	**CCT8**	-	17363	3.194	1719	3026	1284	2.357	1.237	0.089	1
chr11	818900	825571	**PNPLA2**	+	6671	3.497	705	1240	526	2.355	1.236	0.15	0
chr12	4430358	4469190	**C12orf5**	+	38832	2.231	2663	4679	1992	2.349	1.232	0.108	0
chr19	11033443	11039357	**YIPF2**	-	5914	1.008	181	318	135	2.347	1.231	0.229	0
chr10	75561668	75571589	**NDST2**	-	9921	1.64	495	868	371	2.34	1.226	0.162	0
chr11	65190268	65194003	**NEAT1**	+	3735	148.077	17135	30024	12839	2.338	1.226	0.214	0
chr6	32861952	32871535	**LOC100294145**	+	9583	1.039	307	538	230	2.331	1.221	0.171	0
chr1	24128366	24151949	**HMGCL**	-	23583	1.103	796	1392	597	2.331	1.221	0.132	0
chr12	56704212	56710006	**CNPY2**	-	5794	1.962	349	610	262	2.328	1.219	0.173	0
chr17	31254927	31268667	**TMEM98**	+	13740	2.019	849	1484	637	2.327	1.219	0.129	0
chr4	78078356	78091213	**CCNG2**	+	12857	4.149	1633	2851	1226	2.325	1.217	0.117	0
chr14	90863326	90874619	**CALM1**	+	11293	9.424	3289	5739	2472	2.322	1.215	0.112	0
chr12	54356095	54362515	**HOTAIR**	-	6420	3.431	680	1185	511	2.319	1.213	0.131	0
chr12	123745539	123756687	**CDK2AP1**	-	11148	2.088	717	1251	539	2.318	1.213	0.13	0
chr9	115804173	115818996	**ZFP37**	-	14823	1.362	624	1085	470	2.307	1.206	0.134	0
chr7	25158269	25164980	**CYCS**	-	6711	3.776	782	1360	589	2.307	1.206	0.127	0
chr14	96001322	96011055	**GLRX5**	+	9733	1.829	551	958	416	2.305	1.205	0.139	0
chr11	5710816	5732093	**TRIM22**	+	21277	3.558	2355	4090	1777	2.301	1.202	0.104	0
chr1	35441299	35444307	**LOC653160**	-	3008	1.514	138	240	104	2.293	1.197	0.269	0
chr17	7138346	7142825	**PHF23**	-	4479	3.372	466	808	352	2.292	1.197	0.157	0
chr9	107509968	107522403	**NIPSNAP3A**	+	12435	3.301	1277	2210	966	2.287	1.193	0.107	0
chr19	37001588	37019248	**ZNF260**	-	17660	1.3	710	1228	538	2.283	1.191	0.134	0
chr11	105878628	105892954	**KIAA1826**	-	14326	1.629	721	1245	547	2.275	1.186	0.138	0
chr9	34610491	34612101	**C9orf23**	-	1610	1.027	50	86	38	2.267	1.181	0.357	0
chr12	56735381	56754037	**STAT2**	-	18656	3.974	2269	3904	1724	2.264	1.179	0.14	0
chr7	10971579	10979813	**NDUFA4**	-	8234	3.23	830	1428	631	2.262	1.178	0.119	0
chr4	57829515	57843826	**NOA1**	-	14311	1.424	627	1078	477	2.26	1.177	0.157	0
chr1	185265521	185286461	**IVNS1ABP**	-	20940	5.29	3442	5913	2618	2.259	1.175	0.126	0
chr16	19513014	19533450	**GDE1**	-	20436	1.425	900	1544	685	2.253	1.172	0.136	0
chr5	159828647	159846168	**SLU7**	-	17521	1.877	1027	1762	782	2.251	1.171	0.115	0
chr11	67374322	67380012	**NDUFV1**	+	5690	2.424	422	724	322	2.248	1.169	0.196	0
chr19	58144534	58154147	**ZNF211**	+	9613	1.613	478	819	364	2.247	1.168	0.174	0
chr17	7571719	7590863	**TP53**	-	19144	1.435	847	1450	646	2.242	1.165	0.145	0
chr4	79839093	79860582	**PAQR3**	-	21489	1.578	1045	1788	797	2.242	1.165	0.14	0
chr12	6875540	6880118	**PTMS**	+	4578	6.713	943	1613	720	2.239	1.163	0.15	0
chrX	100910267	100914863	**ARMCX2**	-	4596	1.182	168	288	128	2.239	1.163	0.245	0
chr8	134249413	134309547	**NDRG1**	-	60134	1.044	1925	3290	1470	2.237	1.162	0.144	0
chr4	4237268	4249934	**TMEM128**	-	12666	1.308	512	875	391	2.237	1.162	0.17	0
chr4	103998781	104021024	**BDH2**	-	22243	1.307	900	1539	688	2.237	1.161	0.139	0
chr1	27992571	27998724	**IFI6**	-	6153	1.013	189	323	144	2.235	1.16	0.278	0
chr9	22002901	22009312	**CDKN2B**	-	6411	1.566	309	528	236	2.234	1.16	0.217	0
chr16	67968406	67970780	**PSMB10**	-	2374	1.487	107	184	82	2.229	1.157	0.305	0
chr14	24734743	24740803	**RABGGTA**	-	6060	1.381	255	436	195	2.228	1.156	0.246	0
chr12	108956293	108963160	**ISCU**	+	6867	3.921	830	1415	635	2.228	1.156	0.151	0
chrX	100878119	100882831	**ARMCX3**	+	4712	3.117	458	781	351	2.224	1.153	0.166	0
chr14	24612573	24615855	**PSME2**	-	3282	1.577	159	270	121	2.223	1.153	0.273	0
chr22	43035808	43036607	**ATP5L2**	-	799	1.499	37	64	29	2.223	1.153	0.327	0
chr5	158690088	158713048	**UBLCP1**	+	22960	1.945	1394	2373	1068	2.222	1.152	0.12	0
chr4	15683351	15692070	**FAM200B**	+	8719	3.794	1037	1765	794	2.222	1.152	0.119	0
chr9	19115758	19127604	**PLIN2**	-	11846	2.767	1021	1737	782	2.219	1.15	0.131	0
chr7	127010353	127032767	**ZNF800**	-	22414	3.031	2108	3583	1616	2.217	1.149	0.132	0
chr6	24650204	24667115	**TDP2**	-	16911	2.716	1420	2414	1089	2.217	1.148	0.139	0
chr2	37458773	37476303	**C2orf56**	+	17530	1.168	641	1089	491	2.217	1.148	0.144	0
chr9	136218665	136223361	**SURF1**	-	4696	1.119	161	275	124	2.216	1.148	0.27	0
chr2	224822120	224832431	**MRPL44**	+	10311	2.516	812	1379	622	2.214	1.147	0.133	0
chr1	46016454	46035723	**AKR1A1**	+	19269	1.115	656	1114	503	2.212	1.146	0.188	0
chr15	43477465	43489375	**CCNDBP1**	+	11910	2.586	956	1623	734	2.211	1.145	0.141	0
chr19	48972464	48985571	**CYTH2**	+	13107	4.06	1638	2781	1257	2.211	1.144	0.146	0
chr12	105380097	105388505	**C12orf45**	+	8408	1.386	361	611	277	2.2	1.138	0.208	0
chr2	209100952	209119806	**IDH1**	-	18854	1.254	727	1231	559	2.2	1.137	0.173	0
chr5	139929652	139937678	**SRA1**	-	8026	3.605	901	1525	693	2.198	1.136	0.142	0
chr19	58038692	58052244	**ZNF549**	+	13552	1.343	561	948	432	2.192	1.132	0.187	0

chr2	71357443	71377232	**MPHOSPH10**	+	19789	2.22	1369	2312	1055	2.192	1.132	0.134	0
chr1	85715636	85725355	**C1orf52**	-	9719	3.578	1088	1836	839	2.189	1.13	0.132	0
chr1	205271190	205290883	**NUAK2**	-	19693	2.64	1603	2705	1236	2.187	1.129	0.151	0
chr7	99752042	99756302	**C7orf43**	-	4260	1.286	168	283	129	2.185	1.127	0.288	0
chr11	62327072	62341460	**EEF1G**	-	14388	2.785	1247	2101	962	2.184	1.127	0.141	0
chr1	110026560	110035420	**ATXN7L2**	+	8860	2.825	765	1290	591	2.184	1.127	0.19	0
chr1	28585962	28609002	**SESN2**	+	23040	4.464	3151	5306	2432	2.181	1.125	0.175	0
chr19	52196592	52208443	**LINC00085**	+	11851	4.975	1826	3069	1412	2.174	1.12	0.15	0
chr11	57471186	57479673	**MED19**	-	8487	1.514	398	669	308	2.174	1.12	0.21	0
chr17	33738080	33759543	**SLFN12**	-	21463	1.392	934	1569	722	2.173	1.119	0.145	0
chr4	148538538	148556672	**TMEM184C**	+	18134	1.64	930	1562	719	2.172	1.119	0.145	0
chr2	86426555	86440477	**MRPL35**	+	13922	1.157	503	844	389	2.169	1.117	0.178	0
chr5	139712427	139726188	**HBEGF**	-	13761	1.499	635	1066	491	2.169	1.117	0.195	0
chr7	30174551	30202381	**C7orf41**	+	27830	1.227	1062	1783	822	2.168	1.116	0.15	0
chr6	7390061	7418270	**RIOK1**	+	28209	1.126	988	1656	765	2.165	1.114	0.154	0
chr7	27135712	27139877	**HOTAIRM1**	+	4165	1.769	226	379	175	2.163	1.113	0.279	0
chr2	46844324	46852881	**CRIPT**	+	8557	1.431	383	642	297	2.162	1.113	0.198	0
chr5	14581890	14616287	**FAM105A**	+	34397	1.53	1629	2730	1262	2.162	1.113	0.154	0
chr20	5918485	5931173	**TRMT6**	-	12688	1.398	549	920	426	2.158	1.11	0.196	0
chr17	7788122	7816075	**CHD3**	+	27953	1.772	1540	2576	1195	2.155	1.108	0.15	0
chr17	40811265	40819024	**TUBG2**	+	7759	1.258	303	508	235	2.155	1.108	0.229	0
chr3	195241220	195270224	**PPP1R2**	-	29004	1.974	1792	2995	1391	2.152	1.106	0.141	0
chr2	152104727	152118389	**RBM43**	-	13662	1.315	560	935	434	2.151	1.105	0.185	0
chr8	56980738	56987140	**RPS20**	-	6402	4.524	902	1506	700	2.15	1.104	0.162	0
chr12	57984941	57997211	**PIP4K2C**	+	12270	1.446	550	918	427	2.149	1.104	0.199	0
chr10	90750287	90775542	**FAS**	+	25255	2.829	2228	3716	1731	2.146	1.102	0.15	0
chr12	8071823	8088892	**SLC2A3**	-	17069	2.445	1276	2128	992	2.144	1.101	0.19	0
chr1	109642814	109643234	**SCARNA2**	+	420	10.413	133	222	103	2.143	1.1	0.34	0
chr1	45976706	45988562	**PRDX1**	-	11856	6.4	2370	3951	1843	2.143	1.1	0.15	0
chr9	35681989	35690053	**TPM2**	-	8064	34.526	8638	14396	6718	2.143	1.1	0.225	0
chr5	43289492	43313595	**HMGCS1**	-	24103	2.897	2150	3582	1673	2.141	1.098	0.174	0
chr6	36644236	36655116	**CDKN1A**	+	10880	42.736	14152	23575	11011	2.141	1.098	0.313	0
chr13	114567149	114569805	**LOC100506394**	+	2656	1.41	116	194	90	2.14	1.098	0.305	0
chr16	58147496	58163296	**C16orf80**	-	15800	1.77	863	1438	672	2.14	1.098	0.183	0
chrX	54466852	54471731	**TSR2**	+	4879	1.606	243	405	189	2.139	1.097	0.257	0
chr11	121163387	121184119	**SC5DL**	+	20732	1.397	902	1501	702	2.138	1.096	0.168	0
chr17	1945276	1945525	**OVCA2**	+	249	2.609	19	32	15	2.134	1.094	0.517	0
chr11	4406126	4414926	**TRIM21**	-	8800	1.622	440	730	343	2.129	1.09	0.235	0
chr16	3074031	3077756	**THOC6**	+	3725	4.95	563	935	439	2.129	1.09	0.24	0
chr6	31626074	31628549	**C6orf47**	-	2475	2.406	183	304	143	2.127	1.089	0.302	0
chr1	43849587	43855483	**MED8**	-	5896	4.101	755	1252	589	2.125	1.087	0.174	0
chr9	33461350	33473941	**NOL6**	-	12591	1.914	742	1231	579	2.123	1.086	0.205	0
chr6	26634610	26659980	**ZNF322**	-	25370	1.693	1338	2217	1044	2.122	1.086	0.162	0
chr8	125551342	125562227	**NDUFB9**	+	10885	2.289	775	1284	606	2.12	1.084	0.181	0
chr8	81398447	81434610	**ZBTB10**	+	36163	1.01	1145	1896	894	2.119	1.084	0.152	0
chr10	97803158	97820625	**CCNJ**	+	17467	1.375	746	1236	583	2.118	1.083	0.188	0
chr5	112196884	112228776	**SRP19**	+	31892	1.853	1837	3040	1436	2.117	1.082	0.168	0
chr14	61111416	61116155	**SIX1**	-	4739	2.514	365	605	286	2.117	1.082	0.264	0
chr15	66782665	66790146	**SNAPC5**	-	7481	1.548	361	597	282	2.117	1.082	0.232	0
chr13	107142078	107187388	**EFNB2**	-	45310	2.208	3083	5099	2411	2.115	1.081	0.195	0
chr1	113454469	113498975	**SLC16A1**	-	44506	1.448	2002	3311	1566	2.115	1.08	0.17	0
chr13	111365082	111373421	**ING1**	+	8339	1.409	361	598	283	2.114	1.08	0.259	0
chr8	144915754	144923146	**NRBP2**	-	7392	1.221	278	460	218	2.113	1.08	0.276	0
chr13	28194879	28241559	**POLR1D**	+	46680	1.763	2560	4230	2004	2.111	1.078	0.172	0
chr5	34915819	34925787	**BRIX1**	+	9968	3.018	941	1554	736	2.109	1.077	0.169	0
chr3	52232115	52248343	**ALAS1**	+	16228	1.821	919	1515	720	2.104	1.073	0.184	0
chr1	43232915	43241413	**C1orf50**	+	8498	1.305	347	572	272	2.102	1.072	0.233	0
chr10	126085871	126107545	**OAT**	-	21674	3.017	2031	3347	1592	2.102	1.072	0.175	0
chr10	103867324	103880210	**LDB1**	-	12886	3.463	1382	2277	1083	2.101	1.071	0.182	0
chrX	69506210	69509798	**PDZD11**	-	3588	1.048	117	192	91	2.098	1.069	0.327	0
chr7	112063198	112117258	**IFRD1**	+	54060	3.267	5528	9093	4340	2.095	1.067	0.199	0
chr5	75911306	75919240	**F2RL2**	-	7934	3.286	806	1327	633	2.095	1.067	0.206	0
chr17	33570085	33594761	**SLFN5**	+	24676	6.557	5059	8321	3972	2.095	1.067	0.196	0
chr10	28808845	28821283	**LOC220906**	-	12438	2.747	1069	1759	839	2.094	1.067	0.17	0
chr12	2904107	2914587	**FKBP4**	+	10480	1.001	325	534	255	2.094	1.067	0.266	0
chr8	38034105	38070819	**BAG4**	+	36714	1.052	1207	1984	948	2.094	1.066	0.17	0
chr4	100799494	100815703	**LAMTOR3**	-	16209	2.603	1317	2166	1034	2.093	1.066	0.17	0
chrX	102840418	102842655	**TCEAL4**	+	2237	7.827	548	901	430	2.093	1.065	0.204	0
chr20	20015011	20036690	**CRNKL1**	-	21679	1.65	1120	1841	880	2.092	1.065	0.168	0
chr9	35605280	35610038	**TESK1**	+	4758	1.753	255	419	201	2.087	1.061	0.311	0
chr17	34842472	34851662	**ZNHIT3**	+	9190	1.446	410	672	322	2.084	1.059	0.259	0

chrX	54834170	54842445	**MAGED2**	+	8275	2.77	709	1163	558	2.084	1.059	0.218	0
chr19	44010870	44031396	**ETHE1**	-	20526	1.79	1140	1869	897	2.084	1.059	0.191	0
chr5	96496570	96519005	**RIOK2**	-	22435	1.99	1406	2305	1107	2.082	1.058	0.156	0
chr19	44416775	44439411	**ZNF45**	-	22636	1.087	769	1260	605	2.08	1.057	0.194	0
chr9	132589563	132597572	**C9orf78**	-	8009	1.297	324	530	255	2.08	1.057	0.256	0
chr4	90033967	90036052	**TIGD2**	+	2085	1.852	120	197	95	2.076	1.054	0.319	0
chr6	2833733	2842081	**SERPINB1**	-	8348	2.993	782	1278	616	2.073	1.052	0.195	0
chr7	143078359	143088206	**ZYX**	+	9847	13.738	4189	6846	3304	2.072	1.051	0.218	0
chr19	50058967	50083803	**NOSIP**	-	24836	1.106	855	1396	674	2.07	1.05	0.203	0
chr1	156692412	156697705	**ISG20L2**	-	5293	2.641	434	709	342	2.07	1.05	0.249	0
chr3	51991469	52001482	**PCBP4**	-	10013	2.185	675	1102	533	2.067	1.047	0.233	0
chr9	4679565	4706594	**CDC37L1**	+	27029	1.6	1355	2212	1070	2.066	1.047	0.174	0
chr9	125580375	125590935	**PDCL**	-	10560	2.102	690	1126	545	2.066	1.047	0.218	0
chr2	24300302	24308085	**TP53I3**	-	7783	2.076	503	821	397	2.065	1.046	0.235	0
chr11	10533224	10562774	**RNF141**	-	29550	1.735	1611	2627	1272	2.065	1.046	0.169	0
chr19	12175545	12188626	**ZNF844**	+	13081	1.454	596	972	471	2.063	1.045	0.213	0
chr3	180701497	180707562	**DNAJC19**	-	6065	1.13	213	348	168	2.062	1.044	0.299	0
chr14	20757300	20774153	**TTC5**	-	16853	1.052	549	895	434	2.062	1.044	0.243	0
chr15	34376223	34394053	**C15orf24**	-	17830	1.745	983	1601	777	2.06	1.043	0.169	0
chr12	14927269	14930936	**H2AFJ**	+	3667	3.454	393	640	310	2.06	1.043	0.263	0
chr17	73937588	73975515	**ACOX1**	-	37927	1.882	2236	3640	1767	2.06	1.042	0.179	0
chr7	106809405	106842974	**HBP1**	+	33569	2.625	2766	4502	2188	2.057	1.041	0.18	0
chr13	31710762	31736117	**HSPH1**	-	25355	2.343	1843	2998	1458	2.057	1.04	0.201	0
chr11	116649275	116658739	**ZNF259**	-	9464	2.654	779	1268	617	2.055	1.039	0.219	0
chr5	52393894	52405598	**MOCS2**	-	11704	1.276	466	758	369	2.055	1.039	0.24	0
chr2	73989324	74007284	**DUSP11**	-	17960	2.454	1384	2250	1095	2.055	1.039	0.175	0
chr2	178977181	178994382	**RBM45**	+	17201	1.054	567	922	448	2.054	1.039	0.222	0
chr3	150259779	150264428	**SERP1**	-	4649	5.666	823	1338	651	2.054	1.038	0.204	0
chr13	95226307	95248511	**TGDS**	-	22204	1.824	1262	2052	999	2.053	1.037	0.195	0
chr12	56596287	56615753	**RNF41**	-	19466	2.708	1640	2664	1298	2.052	1.037	0.196	0
chr7	134331530	134364567	**BPGM**	+	33037	2.614	2711	4401	2147	2.05	1.035	0.184	0
chr17	73131343	73150775	**HN1**	-	19432	4.717	2853	4628	2262	2.046	1.033	0.207	0
chr12	120532898	120554643	**RAB35**	-	21745	3.124	2105	3409	1670	2.041	1.029	0.215	0
chr15	89441913	89456663	**MFGE8**	-	14750	4.541	2064	3340	1639	2.037	1.027	0.224	0
chr6	29855382	29858856	**HLA-H**	+	3474	1.163	124	201	98	2.036	1.026	0.379	0
chr9	139567594	139581911	**AGPAT2**	-	14317	1.008	442	715	351	2.036	1.025	0.298	0
chr7	99070514	99085217	**ZNF789**	+	14703	1.167	534	863	424	2.035	1.025	0.249	0
chr4	120217573	120225600	**C4orf3**	-	8027	1.721	435	704	346	2.035	1.025	0.235	0
chr8	22993103	23021540	**TNFRSF10D**	-	28437	5.469	4843	7829	3848	2.035	1.025	0.232	0
chr2	70142172	70170076	**MXD1**	+	27904	1.128	976	1577	775	2.034	1.024	0.222	0
chr17	79202076	79212891	**C17orf56**	-	10815	1.343	446	721	354	2.033	1.024	0.291	0
chr19	48248792	48260323	**GLTSCR2**	+	11531	2.109	754	1219	600	2.032	1.023	0.234	0
chr10	44139306	44144326	**ZNF32**	-	5020	1.177	186	300	148	2.032	1.023	0.3	0
chr6	26158348	26171576	**HIST1H2BD**	+	13228	1.079	448	723	356	2.03	1.022	0.245	0
chr4	184560788	184580331	**RWDD4**	-	19543	1.009	616	995	490	2.03	1.021	0.233	0
chr17	14207056	14209062	**MGC12916**	+	2006	5.551	342	552	272	2.03	1.021	0.313	0
chr6	24403152	24425816	**MRS2**	+	22664	1.189	843	1360	670	2.028	1.02	0.215	0
chr19	56879467	56891196	**ZNF542**	+	11729	1.02	373	603	297	2.028	1.02	0.269	0
chr6	43021766	43027242	**MRPL2**	-	5476	1.628	279	450	222	2.024	1.017	0.289	0
chr6	30851860	30867933	**DDR1**	+	16073	1.355	675	1089	538	2.024	1.017	0.246	0
chr7	92158086	92166823	**RBM48**	+	8737	1.753	483	778	385	2.023	1.016	0.231	0
chr1	145575987	145586546	**PIAS3**	+	10559	4.091	1341	2161	1068	2.022	1.016	0.218	0
chr19	58125829	58133636	**ZNF134**	+	7807	3.362	822	1324	655	2.021	1.015	0.215	0
chr3	187439164	187463513	**BCL6**	-	24349	4.361	3297	5309	2626	2.021	1.015	0.229	0
chr8	182199	197339	**ZNF596**	+	15140	1.049	493	794	393	2.021	1.015	0.267	0
chr3	51967445	51975922	**RRP9**	-	8477	1.256	328	528	262	2.018	1.013	0.313	0
chr2	241065979	241075764	**MYEOV2**	-	9785	1.618	489	786	389	2.018	1.013	0.285	0
chr3	151980404	151987415	**LOC401093**	-	7011	1.653	360	579	287	2.017	1.012	0.289	0
chr14	74416636	74429813	**COQ6**	+	13177	1.208	495	796	394	2.016	1.012	0.268	0
chr1	89318320	89357301	**GTF2B**	-	38981	2.36	2883	4636	2299	2.016	1.011	0.207	0
chr6	64281919	64293489	**PTP4A1**	+	11570	8.692	3127	5027	2494	2.016	1.011	0.226	0
chr6	42989384	42997337	**RRP36**	+	7953	2.141	535	859	426	2.015	1.01	0.24	0
chr6	134274300	134308629	**TBPL1**	+	34329	2.161	2335	3751	1863	2.014	1.01	0.195	0
chr5	140071017	140078890	**HARS2**	+	7873	2.965	728	1170	581	2.013	1.009	0.233	0
chr20	52824501	52836492	**PFDN4**	+	11991	2.076	782	1256	624	2.012	1.009	0.217	0
chr10	22610138	22620414	**BMI1**	+	10276	1.308	420	675	336	2.008	1.005	0.269	0
chr4	3505323	3534224	**LRPAP1**	-	28901	1.354	1205	1932	962	2.007	1.005	0.246	0
chr8	17913924	17942507	**ASAH1**	-	28583	1.085	958	1536	766	2.005	1.004	0.248	0
chr1	193065594	193075244	**GLRX2**	-	9650	1.924	578	926	462	2.004	1.003	0.258	0
chr19	44598481	44612479	**ZNF224**	+	13998	1.633	713	1142	570	2.003	1.002	0.244	0
chr6	10695187	10709970	**PAK1IP1**	+	14783	1.349	629	1007	503	2.002	1.002	0.225	0

chr8	110346551	110358189	**ENY2**	+	11638	3.041	1109	1776	887	2.002	1.001	0.214	0
chr11	6416354	6440644	**APBB1**	-	24290	1.076	817	1309	654	2.002	1.001	0.23	0
chr5	150070351	150080669	**RBM22**	-	10318	3.001	972	1556	777	2.002	1.001	0.215	0
chr11	64018994	64036924	**PLCB3**	+	17930	1.302	721	1155	577	2.001	1.001	0.264	0
chr10	71962585	71993190	**PPA1**	-	30605	1.098	1050	1681	840	2.001	1.001	0.221	0
**Total**					**4942767**								
**Average**					**14203**								

Table S3B. Genes DOWN-regulated >2-fold following 45 min CPT treatment, wash and 15 min Bru (CPT+15m).

chrom	start	end	name	strand	bp	meanRPKM	meanCount	1_CPT15m	2_cont	foldChange	log2FoldCha	pAdjusted	significant
chr6	27861202	27861669	**HIST1H2BO**	+	467	1.175	21	0	28	0	-9999	0.016	1
chr12	11138511	11139511	**TAS2R50**	-	1000	1.054	40	0	53	0	-9999	0.002	1
chr6	26199786	26200216	**HIST1H2BF**	+	430	1.074	16	0	22	0.032	-4.956569	0.06	1
chr11	121986061	121986923	**BLID**	-	862	5.635	180	8	238	0.036	-4.792304	0	1
chr13	28519342	28519710	**ATP5EP2**	+	368	3.314	44	2	58	0.049	-4.345425	0.018	1
chr6	27114907	27115346	**HIST1H2AH**	+	439	2.055	33	2	44	0.049	-4.358723	0.03	1
chr6	27107087	27107457	**HIST1H4I**	+	370	1.385	18	1	24	0.058	-4.095714	0.077	1
chr18	57098170	57364644	**CCBE1**	-	266474	4.757	47854	4315	62366	0.069	-3.853043	0.046	1
chr6	27775976	27776445	**HIST1H2AI**	+	469	1.277	23	2	30	0.072	-3.804948	0.086	1
chr16	56642477	56643409	**MT2A**	+	932	39.833	1332	128	1733	0.074	-3.756491	0	1
chr12	1726221	1756378	**WNT5B**	+	30157	3.76	4176	424	5426	0.078	-3.674724	0	1
chr19	45971252	45978437	**FOSB**	+	7185	17.805	4506	495	5843	0.085	-3.561011	0	1
chr6	26021906	26022278	**HIST1H4A**	+	372	1.862	25	2	32	0.087	-3.519534	0.089	1
chr12	120123594	120315095	**CIT**	-	191501	1.351	9629	1121	12465	0.09	-3.474533	0.002	1
chr5	64444562	64777704	**ADAMTS6**	-	333142	1.964	25496	2992	32997	0.091	-3.462999	0.023	1
chr11	19372270	20143147	**NAV2**	+	770877	1.812	50944	6070	65902	0.092	-3.440553	0.079	1
chr8	70378858	70573147	**SULF1**	+	194289	4.983	38787	4941	50069	0.099	-3.340929	0.068	1
chr1	209848669	209849735	**G0S2**	+	1066	4.617	177	22	228	0.1	-3.318286	0.009	1
chr10	78629358	79397577	**KCNMA1**	-	768219	2.716	76722	10016	98958	0.101	-3.304482	0.195	0
chr9	140657473	140659224	**FLJ40292**	+	1751	1.875	118	15	152	0.103	-3.277587	0.017	1
chr6	26271145	26271612	**HIST1H3G**	-	467	11.05	186	25	240	0.104	-3.259831	0.009	1
chr6	27100816	27101314	**HIST1H2AG**	+	498	3	56	7	72	0.108	-3.205763	0.054	1
chr13	60239722	60738119	**DIAPH3**	-	498397	1.437	27133	3791	34914	0.109	-3.203036	0.036	1
chr8	31497267	32622558	**NRG1**	+	1125291	1.23	52556	7394	67610	0.109	-3.192752	0.122	0
chr1	115828536	115880857	**NGF**	-	52321	5.36	10595	1549	13611	0.114	-3.135177	0.008	1
chr5	140682195	140683612	**SLC25A2**	-	1417	1.671	86	12	110	0.117	-3.097335	0.037	1
chr14	73136659	73360809	**DPF3**	-	224150	1.603	13296	2032	17051	0.119	-3.068238	0.012	1
chr22	46692637	46726707	**GTSE1**	+	34070	1.253	1553	247	1988	0.124	-3.008178	0.001	1
chr7	55086724	55275031	**EGFR**	+	188307	3.995	27565	4448	35271	0.126	-2.987131	0.046	1
chr6	1312674	1314993	**FOXQ1**	+	2319	1.789	145	23	186	0.127	-2.976651	0.02	1
chr6	27833106	27833576	**HIST1H2AL**	+	470	2.257	38	6	49	0.13	-2.945673	0.101	0
chr1	214776531	214837914	**CENPF**	+	61383	2.248	5133	868	6554	0.132	-2.915999	0.004	1
chr1	197053256	197115824	**ASPM**	-	62568	2.827	6648	1154	8480	0.136	-2.876972	0.007	1
chr6	26250369	26250835	**HIST1H3F**	-	466	3.283	56	10	72	0.139	-2.844214	0.083	1
chr13	38210772	38443939	**TRPC4**	-	233167	1.602	14403	2564	18350	0.14	-2.839003	0.027	1
chr19	38397867	38699008	**SIPA1L3**	+	301141	1.428	15929	2889	20275	0.143	-2.810626	0.027	1
chr20	9049700	9461462	**PLCB4**	+	411762	4.242	68810	12706	87511	0.145	-2.783846	0.29	0
chr5	126112314	126172712	**LMNB1**	+	60398	1.879	4218	815	5352	0.152	-2.7145	0.007	1
chr11	123396527	123493518	**GRAMD1B**	+	96991	1.34	4775	929	6057	0.153	-2.704357	0.007	1
chr7	83587658	83824217	**SEMA3A**	-	236559	2.309	21043	4072	26700	0.153	-2.712978	0.06	1
chr15	40453209	40513337	**BUB1B**	+	60128	1.009	2244	441	2846	0.155	-2.688774	0.005	1
chr2	40339285	40739575	**SLC8A1**	-	400290	1.955	30626	6226	38760	0.161	-2.638125	0.121	0
chr7	36429431	36493400	**ANLN**	+	63969	4.628	11071	2280	14002	0.163	-2.61847	0.027	1
chr16	17196180	17564738	**XYLT1**	-	368558	1.591	21558	4451	27260	0.163	-2.614511	0.061	1
chr11	33563876	33695646	**C11orf41**	+	131770	1.432	6951	1438	8789	0.164	-2.610778	0.014	1
chr14	75745480	75748937	**FOS**	+	3457	49.744	6073	1256	7679	0.164	-2.611911	0.009	1
chr2	9346893	9545812	**ASAP2**	+	198919	1.879	13596	2861	17174	0.167	-2.585214	0.032	1
chr1	97543299	98386615	**DPYD**	-	843316	1.445	46420	9863	58606	0.168	-2.570874	0.207	0
chr8	119201694	119634184	**SAMD12**	-	432490	1.127	18440	3902	23286	0.168	-2.57703	0.06	1
chr19	47421932	47508333	**ARHGAP35**	+	86401	3.087	9799	2100	12366	0.17	-2.557252	0.024	1
chr16	86600856	86602537	**FOXC2**	+	1681	2.357	136	29	172	0.17	-2.552352	0.044	1
chr3	27257096	27410912	**NEK10**	-	153816	1.644	9328	2004	11769	0.17	-2.55398	0.023	1
chr10	49654078	49813138	**ARHGAP22**	-	159060	2.897	16605	3590	20944	0.171	-2.544488	0.045	1
chr6	26031816	26032288	**HIST1H3B**	-	472	12.507	216	46	272	0.171	-2.549281	0.037	1
chr4	16503164	16900424	**LDB2**	-	397260	1.055	16225	3504	20465	0.171	-2.546103	0.059	1
chr6	57182421	57513376	**PRIM2**	+	330955	1.541	18752	4040	23656	0.171	-2.549573	0.057	1
chr4	104026962	104119566	**CENPE**	-	92604	1.151	3963	865	4995	0.173	-2.528863	0.011	1
chr12	2966846	2986321	**FOXM1**	-	19475	1.261	891	194	1123	0.173	-2.527486	0.009	1
chr3	193853930	193856401	**HES1**	+	2471	2.71	234	51	295	0.174	-2.519693	0.03	1
chr9	36572904	36677679	**MELK**	+	104775	1.338	5186	1139	6535	0.174	-2.51999	0.014	1
chr11	58390145	58393205	**CNTF**	+	3060	4.035	446	98	561	0.176	-2.506463	0.019	1
chr14	101361106	101373305	**MEG8**	+	12199	2.261	1006	224	1267	0.177	-2.498681	0.009	1
chr16	55600583	55601592	**CAPNS2**	+	1009	1.237	44	10	56	0.178	-2.49221	0.15	0
chr14	51955854	52197444	**FRMD6**	+	241590	13.86	126916	28503	159720	0.178	-2.486326	0.611	0
chr1	156495196	156542396	**IQGAP3**	-	47200	1.55	2678	599	3371	0.178	-2.492731	0.009	1
chr4	1873122	1983934	**WHSC1**	+	110812	2.328	9445	2134	11882	0.18	-2.476726	0.027	1
chr3	71003864	71633140	**FOXP1**	-	629276	1.324	31357	7145	39428	0.181	-2.464096	0.142	0
chr17	27401932	27402627	**TIAF1**	-	695	1.111	28	6	35	0.182	-2.459813	0.218	0

chr22	42334740	42343148	**CENPM**	-	8408	2.299	690	159	868	0.183	-2.448207	0.013	1
chr8	131094983	131097014	**LOC100507117**	-	2031	5.042	379	87	477	0.183	-2.448674	0.03	1
chr3	42132745	42267268	**TRAK1**	+	134523	2.437	11952	2774	15012	0.185	-2.435815	0.038	1
chr15	81293294	81296345	**MESDC1**	+	3051	3.936	419	98	526	0.186	-2.424299	0.02	1
chr1	201617449	201796102	**NAV1**	+	178653	3.288	21848	5094	27433	0.186	-2.428834	0.092	1
chr5	169010637	169031781	**CCDC99**	+	21144	6.27	5001	1181	6274	0.188	-2.408793	0.02	1
chr4	157682762	157892546	**PDGFC**	-	209784	3.614	27966	6591	35091	0.188	-2.412527	0.126	0
chr1	240255184	240638489	**FMN2**	+	383305	5.507	80285	19027	100704	0.189	-2.404	0.424	0
chr17	3627196	3629992	**GSG2**	+	2796	1.502	151	35	189	0.189	-2.405282	0.063	1
chr5	140864740	140867356	**PCDHGC4**	+	2616	1.956	181	42	227	0.189	-2.402499	0.05	1
chr6	160514113	160517244	**LOC729603**	+	3131	1.823	204	48	256	0.19	-2.394005	0.048	1
chr9	113636053	113800365	**LPAR1**	-	164312	6.245	37829	9019	47432	0.19	-2.394767	0.182	0
chr3	10326102	10327430	**GHRLOS2**	+	1328	1.821	86	20	108	0.191	-2.390404	0.106	0
chr18	2571509	2616634	**NDC80**	+	45125	1.167	1951	467	2445	0.191	-2.386058	0.013	1
chr1	149858524	149858961	**HIST2H2AC**	+	437	3.485	56	13	71	0.192	-2.384108	0.162	0
chr10	91461366	91534700	**KIF20B**	+	73334	1.199	3272	791	4099	0.193	-2.372304	0.016	1
chr9	126141932	126692417	**DENND1A**	-	550485	1.265	25358	6173	31753	0.194	-2.362805	0.114	0
chr11	118964584	118966177	**H2AFX**	-	1593	5.989	336	81	421	0.194	-2.368711	0.03	1
chr8	27667137	27695349	**PBK**	-	28212	1.469	1522	369	1907	0.194	-2.366776	0.013	1
chr5	113698015	113832197	**KCNN2**	+	134182	3.412	16899	4171	21142	0.197	-2.341593	0.077	1
chr4	160188997	160281301	**RAPGEF2**	+	92304	1.787	6325	1559	7913	0.197	-2.342799	0.032	1
chr4	20255234	20620788	**SLIT2**	+	365554	3.013	41499	10266	51910	0.198	-2.338105	0.231	0
chr11	64844926	64851615	**CDCA5**	-	6689	2.158	518	128	648	0.199	-2.329209	0.024	1
chr9	128199672	128469513	**MAPKAP1**	-	269841	1.787	17707	4411	22139	0.199	-2.32713	0.08	1
chr2	56411257	56613309	**CCDC85A**	+	202052	7.533	59068	14747	73841	0.2	-2.323946	0.351	0
chr10	129894924	129924468	**MKI67**	-	29544	3.938	4264	1064	5331	0.2	-2.324796	0.019	1
chr8	118811601	119124058	**EXT1**	-	312457	11.583	134746	33941	168347	0.202	-2.310305	0.677	0
chr2	54080049	54087170	**GPR75**	-	7121	1.994	513	130	641	0.203	-2.29816	0.027	1
chr5	43014830	43018913	**LOC648987**	-	4083	2.015	297	75	371	0.203	-2.302154	0.045	1
chr12	64238540	64541613	**SRGAP1**	+	303073	4.561	51802	13142	64689	0.203	-2.299277	0.298	0
chr17	8108048	8113883	**AURKB**	-	5835	4.496	943	240	1178	0.204	-2.290676	0.016	1
chr12	66151800	66220754	**RPSAP52**	-	68954	1.108	2703	689	3374	0.204	-2.291273	0.014	1
chr14	71996041	72206120	**SIPA1L1**	+	210079	3.01	23728	6046	29622	0.204	-2.292526	0.132	0
chr18	55313658	55470327	**ATP8B1**	-	156669	4.336	24990	6381	31193	0.205	-2.289359	0.132	0
chr10	94352824	94415152	**KIF11**	+	62328	1.523	3522	901	4396	0.205	-2.286024	0.02	1
chr5	14143828	14509458	**TRIO**	+	365630	7.75	105066	26836	131143	0.205	-2.288892	0.554	0
chr3	27414213	27498245	**SLC4A7**	-	84032	6.28	19948	5132	24887	0.206	-2.277788	0.115	0
chr6	33359312	33377699	**KIFC1**	+	18387	2.207	1472	382	1835	0.208	-2.262175	0.016	1
chr1	94027348	94147385	**BCAR3**	-	120037	7.441	32503	8492	40507	0.21	-2.25387	0.177	0
chr17	43003447	43025082	**KIF18B**	-	21635	1.949	1536	401	1914	0.21	-2.254319	0.016	1
chr1	163291722	163325553	**NUF2**	+	33831	1.579	1951	513	2430	0.211	-2.244179	0.018	1
chr8	22497883	22499722	**FLJ14107**	-	1839	2.743	181	48	225	0.213	-2.23399	0.078	1
chr3	29322802	30051886	**RBMS3**	+	729084	1.135	31549	8382	39271	0.213	-2.228032	0.207	0
chr9	82186877	82341656	**TLE4**	+	154779	21.373	126507	33646	157461	0.214	-2.226469	0.681	0
chr12	66218239	66360071	**HMGA2**	+	141832	9.589	48053	12878	59778	0.215	-2.214689	0.261	0
chr19	36545782	36596012	**WDR62**	+	50230	1.083	1948	523	2422	0.216	-2.209552	0.017	1
chr9	99148224	99180669	**ZNF367**	-	32445	1.242	1478	397	1839	0.216	-2.209216	0.02	1
chr14	95883830	95942173	**C14orf49**	-	58343	1.262	2622	710	3260	0.218	-2.199007	0.018	1
chr9	128509616	128729655	**PBX3**	+	220039	4.913	39022	10567	48507	0.218	-2.198501	0.227	0
chr12	24962957	25102393	**BCAT1**	-	139436	1.186	6195	1684	7698	0.219	-2.192182	0.041	1
chr17	12569206	12670651	**MYOCD**	+	101445	1.335	5333	1454	6626	0.22	-2.187555	0.049	1
chr7	158424002	158497520	**NCAPG2**	-	73518	1.337	3591	983	4460	0.22	-2.18186	0.026	1
chr9	16409500	16870786	**BNC2**	-	461286	1.612	27476	7553	34117	0.221	-2.175322	0.174	0
chrX	134184962	134186221	**FAM127B**	-	1259	1.531	67	18	84	0.221	-2.179823	0.16	0
chr5	72921982	73237818	**RGNEF**	+	315836	1.197	13734	3770	17056	0.221	-2.177447	0.079	1
chr1	66999824	67210768	**SGIP1**	+	210944	7.749	60393	16552	75007	0.221	-2.180028	0.37	0
chr19	15348300	15391262	**BRD4**	-	42962	3.442	5298	1463	6577	0.222	-2.168272	0.03	1
chr18	55711609	56068772	**NEDD4L**	+	357163	1.395	17855	4912	22169	0.222	-2.174189	0.1	0
chr15	41624891	41673248	**NUSAP1**	+	48357	1.279	2272	628	2819	0.223	-2.165834	0.024	1
chr16	2479394	2508859	**CCNF**	+	29465	1.305	1374	386	1703	0.227	-2.140796	0.02	1
chr11	66099541	66104000	**RIN1**	-	4459	4.475	703	197	871	0.227	-2.140428	0.027	1
chr17	78518624	78940173	**RPTOR**	+	421549	1.515	23016	6466	28533	0.227	-2.141613	0.141	0
chr1	243651534	244006886	**AKT3**	-	355352	1.815	24138	6810	29914	0.228	-2.135078	0.17	0
chr5	140868807	140871444	**PCDHGC5**	+	2637	1.686	157	44	194	0.228	-2.133783	0.098	1
chr6	128289923	128841870	**PTPRK**	-	551947	4.019	81831	23156	101390	0.228	-2.130416	0.491	0
chr13	33677271	34250932	**STARD13**	-	573661	1.243	26400	7490	32704	0.229	-2.126391	0.182	0
chr1	154947117	154951725	**CKS1B**	+	4608	1.521	256	73	318	0.23	-2.122114	0.08	1
chr12	72666528	73059422	**TRHDE**	+	392894	2.334	34338	9769	42528	0.23	-2.122154	0.246	0
chr1	208195587	208417665	**PLXNA2**	-	222078	1.181	9709	2775	12020	0.231	-2.114813	0.07	1
chr2	36583369	36778278	**CRIM1**	+	194909	25.08	184778	53100	228670	0.232	-2.106467	0.932	0
chr15	91509267	91537804	**PRC1**	-	28537	2.113	2192	629	2713	0.232	-2.107143	0.027	1

chrX	96138906	96140450	**RPA4**	+	1544	1.038	59	17	74	0.232	-2.106422	0.224	0
chr17	76210276	76221716	**BIRC5**	+	11440	2.827	1169	336	1446	0.233	-2.102593	0.027	1
chr6	80714321	80752244	**TTK**	+	37923	1.344	1871	540	2315	0.233	-2.099526	0.028	1
chr2	215593274	215674428	**BARD1**	-	81154	2.183	6486	1874	8023	0.234	-2.097768	0.047	1
chr19	18390562	18392432	**JUND**	-	1870	25.634	1658	479	2051	0.234	-2.097342	0.019	1
chr1	210111537	210337633	**SYT14**	+	226096	1.242	9992	2897	12357	0.234	-2.092378	0.061	1
chr20	30326903	30389603	**TPX2**	+	62700	2.005	4609	1334	5701	0.234	-2.095383	0.038	1
chr2	113495443	113522254	**CKAP2L**	-	26811	1.748	1720	502	2126	0.236	-2.081485	0.03	1
chr15	64791618	64978266	**ZNF609**	+	186648	1.039	7182	2095	8878	0.236	-2.083227	0.058	1
chr4	150999425	151178608	**DCLK2**	+	179183	10.458	70983	20766	87722	0.237	-2.078712	0.473	0
chr6	27840925	27841289	**HIST1H4L**	-	364	1.606	21	6	27	0.238	-2.072851	0.38	0
chr14	21668237	21675059	**LOC283624**	-	6822	7.423	1820	535	2248	0.238	-2.068718	0.027	1
chr17	38544772	38574202	**TOP2A**	-	29430	3.59	3909	1148	4829	0.238	-2.072047	0.039	1
chr1	162602227	162750247	**DDR2**	+	148020	4.075	21981	6501	27141	0.24	-2.061641	0.159	0
chr2	225629806	225907330	**DOCK10**	-	277524	4.45	46118	13651	56940	0.24	-2.060407	0.333	0
chr17	64298925	64806862	**PRKCA**	+	507937	3.614	67304	19931	83095	0.24	-2.05972	0.438	0
chr17	61086897	61505067	**TANC2**	+	418170	2.946	46489	13793	57388	0.24	-2.056795	0.345	0
chr10	320129	735608	**DIP2C**	-	415479	1.598	23968	7134	29579	0.241	-2.051656	0.17	0
chr5	137514416	137523404	**KIF20A**	+	8988	2.314	760	227	938	0.242	-2.046071	0.041	1
chr8	25316512	25365425	**CDCA2**	+	48913	1.54	2747	824	3388	0.243	-2.038487	0.035	1
chr11	12132137	12285331	**MICAL2**	+	153194	4.749	26512	7941	32702	0.243	-2.041891	0.197	0
chr4	148653452	148993927	**ARHGAP10**	+	340475	1.734	21637	6500	26683	0.244	-2.037409	0.168	0
chr5	178537851	178772431	**ADAMTS2**	-	234580	2.065	17548	5299	21631	0.245	-2.029132	0.133	0
chr3	172472297	172539263	**ECT2**	+	66966	1.964	4838	1461	5963	0.245	-2.029197	0.047	1
chr17	6917852	6918055	**RNASEK-C17OR**	+	203	1.031	7	2	8	0.245	-2.026423	0.506	0
chr21	36160097	36421595	**RUNX1**	-	261498	4.23	40276	12156	49649	0.245	-2.030026	0.291	0
chr16	81478774	81745367	**CMIP**	+	266593	1.496	14352	4348	17686	0.246	-2.024208	0.108	0
chr5	140810157	140812789	**PCDHGA12**	+	2632	1.453	134	40	166	0.246	-2.02314	0.131	0
chr4	114372187	114683083	**CAMK2D**	-	310896	3.268	37526	11432	46224	0.247	-2.015476	0.289	0
chr2	121554866	121750229	**GLI2**	+	195363	1.497	10649	3243	13117	0.247	-2.016043	0.087	1
chr10	69681655	69835103	**HERC4**	-	153448	8.456	47089	14326	58011	0.247	-2.017651	0.331	0
chr11	9800213	10315754	**SBF2**	-	515541	1.171	22357	6798	27544	0.247	-2.018533	0.184	0
chr12	11285883	11286843	**TAS2R30**	-	960	1.962	68	20	84	0.247	-2.017406	0.219	0
chr1	43824625	43828873	**CDC20**	+	4248	2.745	420	128	517	0.248	-2.012837	0.066	1
chr8	25042286	25270619	**DOCK5**	+	228333	4.254	35940	10980	44260	0.248	-2.011139	0.285	0
chr1	180238797	180243816	**LOC100527964**	-	5019	1.432	255	78	314	0.248	-2.009948	0.089	1
chr18	52889561	53303188	**TCF4**	-	413627	2.902	46034	14045	56697	0.248	-2.013183	0.374	0
chr6	26045638	26046097	**HIST1H3C**	+	459	8.333	137	42	169	0.249	-2.00385	0.144	0
chr8	126104082	126379367	**NSMCE2**	+	275285	1.019	10289	3148	12670	0.249	-2.008679	0.089	1
chr13	30083550	30169825	**SLC7A1**	-	86275	5.161	16115	4931	19843	0.249	-2.008554	0.128	0
chr17	75277491	75496678	**9-Sep**	+	219187	3.08	23943	7373	29467	0.25	-1.998695	0.173	0
chr11	102706527	102714342	**MMP3**	-	7815	1.366	379	116	466	0.25	-1.998654	0.068	1
chr8	128806778	129113499	**PVT1**	+	306721	2.56	28261	8697	34782	0.25	-1.999778	0.215	0
chr3	187871662	188608460	**LPP**	+	736798	2.173	58968	18199	72557	0.251	-1.995236	0.419	0
chr6	144612872	145174170	**UTRN**	+	561298	1.765	36652	11329	45093	0.251	-1.992804	0.292	0
chr3	88188261	88193814	**ZNF654**	+	5553	1.847	382	118	470	0.251	-1.993782	0.089	1
chr10	75910942	76469061	**ADK**	+	558119	1.529	31448	9733	38686	0.252	-1.990862	0.255	0
chr10	69869249	69971773	**MYPN**	+	102524	1.742	6579	2042	8091	0.252	-1.985814	0.068	1
chr12	11148560	11150474	**TAS2R20**	-	1914	1.263	90	27	110	0.252	-1.987466	0.213	0
chr4	72053002	72437804	**SLC4A4**	+	384802	1.149	16807	5234	20664	0.253	-1.981035	0.168	0
chr4	2627158	2734302	**FAM193A**	+	107144	1.548	6013	1883	7389	0.255	-1.972357	0.06	1
chr3	192514604	192635950	**MB21D2**	-	121346	4.158	18442	5788	22660	0.255	-1.968929	0.16	0
chr22	50171537	50173958	**LOC90834**	-	2421	2.117	184	58	226	0.256	-1.963361	0.131	0
chr9	18474078	18910947	**ADAMTSL1**	+	436869	1.304	20354	6428	24997	0.257	-1.959241	0.164	0
chr1	38158158	38175391	**CDCA8**	+	17233	2.865	1782	564	2189	0.258	-1.954832	0.037	1
chr4	48499379	48782316	**FRYL**	-	282937	1.442	15150	4807	18598	0.258	-1.951839	0.149	0
chr11	66624875	66627946	**LRFN4**	+	3071	1.347	144	45	177	0.258	-1.956222	0.139	0
chr19	11257830	11266484	**SPC24**	-	8654	1.157	359	113	440	0.258	-1.952356	0.083	1
chr12	1100403	1605099	**ERC1**	+	504696	1.008	18779	5971	23048	0.259	-1.948602	0.174	0
chr2	234745485	234763212	**HJURP**	-	17727	5.841	3705	1175	4548	0.259	-1.95162	0.044	1
chr10	80828791	81076285	**ZMIZ1**	+	247494	4.638	41017	13032	50346	0.259	-1.949715	0.309	0
chr3	151985828	152183569	**MBNL1**	+	197741	8.668	63872	20380	78369	0.26	-1.943085	0.471	0
chr17	55333930	55757299	**MSI2**	+	423369	1.285	19803	6348	24288	0.261	-1.935728	0.176	0
chrY	14813159	14972768	**USP9Y**	+	159609	1.467	8599	2760	10546	0.262	-1.933887	0.092	1
chr7	139246315	139477693	**HIPK2**	-	231378	1.712	14427	4648	17686	0.263	-1.927861	0.139	0
chr5	9035137	9546233	**SEMA5A**	-	511096	2.287	42618	13729	52248	0.263	-1.928101	0.338	0
chr6	129898239	130031370	**ARHGAP18**	-	133131	3.658	18226	5899	22334	0.264	-1.920626	0.189	0
chr7	137559724	137686846	**CREB3L2**	-	127122	4.961	23199	7493	28435	0.264	-1.924023	0.217	0
chr3	63989697	63997917	**LOC100507062**	+	8220	2.565	776	251	951	0.264	-1.919857	0.06	1
chr12	50382944	50419307	**RACGAP1**	-	36363	1.633	2178	703	2670	0.264	-1.923988	0.046	1
chr4	152041432	152149182	**SH3D19**	-	107750	1.16	4690	1516	5748	0.264	-1.921884	0.072	1

chr1	44173217	44396831	**ST3GAL3**	+	223614	1.077	8753	2831	10727	0.264	-1.921423	0.091	1
chr20	54944444	54967351	**AURKA**	-	22907	1.669	1392	452	1705	0.265	-1.915657	0.046	1
chr3	11178778	11304939	**HRH1**	+	126161	1.281	5806	1886	7112	0.265	-1.914469	0.064	1
chr8	89049459	89339717	**MMP16**	-	290258	1.05	11280	3665	13818	0.265	-1.914642	0.125	0
chr10	33466418	33623833	**NRP1**	-	157415	8.206	47156	15285	57779	0.265	-1.918365	0.374	0
chr6	26027123	26027480	**HIST1H4B**	-	357	21.146	270	88	331	0.266	-1.910253	0.109	0
chr5	119800018	120022964	**PRR16**	+	222946	2.502	20452	6671	25046	0.266	-1.908565	0.196	0
chr22	42556018	42611445	**TCF20**	-	55427	1.045	2106	685	2579	0.266	-1.911438	0.046	1
chr6	130758261	130764210	**TMEM200A**	+	5949	1.104	232	75	284	0.267	-1.907078	0.116	0
chr10	128594022	129250780	**DOCK1**	+	656758	1.131	26843	8796	32859	0.268	-1.901299	0.238	0
chr1	85784167	86044046	**DDAH1**	-	259879	7.762	74880	24629	91631	0.269	-1.89547	0.536	0
chr5	108083522	108523373	**FER**	+	439851	1.262	20479	6744	25057	0.269	-1.893514	0.207	0
chr6	33589155	33664348	**ITPR3**	+	75193	1.555	4102	1354	5018	0.27	-1.889633	0.047	1
chr6	101846860	102517958	**GRIK2**	+	671098	2.028	51715	17128	63244	0.271	-1.8845	0.446	0
chr22	24236564	24237409	**MIF**	+	845	10.855	322	106	394	0.271	-1.885889	0.096	1
chr2	168810529	169104105	**STK39**	-	293576	1.71	18311	6069	22392	0.271	-1.883368	0.183	0
chr12	77415025	77459360	**E2F7**	-	44335	2.403	3836	1281	4687	0.273	-1.870479	0.058	1
chr3	127407908	127542051	**MGLL**	-	134143	1.483	7124	2389	8703	0.274	-1.865134	0.082	1
chr12	11090852	11091806	**TAS2R14**	-	954	1.052	36	12	44	0.274	-1.866067	0.337	0
chr3	149086804	149095568	**TM4SF1**	-	8764	2.653	835	279	1020	0.274	-1.868598	0.058	1
chr15	77516249	77517746	**C15orf5**	-	1497	3.543	198	66	242	0.275	-1.864803	0.174	0
chr15	81071711	81243999	**KIAA1199**	+	172288	9.549	61470	20615	75089	0.275	-1.864861	0.488	0
chr17	13399005	13505244	**HS3ST3A1**	-	106239	1.594	6200	2089	7571	0.276	-1.857335	0.086	1
chr11	58294343	58345639	**LPXN**	-	51296	4.123	7633	2570	9321	0.276	-1.858552	0.092	1
chr8	59717976	60031767	**TOX**	-	313791	2.686	31310	10539	38234	0.276	-1.859078	0.308	0
chr6	16299342	16761721	**ATXN1**	-	462379	2.663	44673	15131	54520	0.278	-1.849275	0.377	0
chr10	60272903	60588845	**BICC1**	+	315942	2.601	30041	10194	36657	0.278	-1.846279	0.289	0
chr7	116312458	116438440	**MET**	+	125982	2.831	13017	4432	15879	0.279	-1.840901	0.15	0
chr3	136055998	136471245	**STAG1**	-	415247	1.612	24684	8403	30111	0.279	-1.841161	0.257	0
chr11	28042162	28129746	**KIF18A**	-	87584	1.795	5817	1984	7094	0.28	-1.837706	0.093	1
chr2	235860627	235964358	**SH3BP4**	+	103731	6.952	25768	8809	31420	0.28	-1.83459	0.245	0
chr2	238395877	238463961	**MLPH**	+	68084	1.646	3956	1354	4823	0.281	-1.832541	0.058	1
chr3	114056946	114866127	**ZBTB20**	-	809181	1.131	33779	11561	41185	0.281	-1.832754	0.327	0
chr22	19033674	19035888	**DGCR11**	-	2214	1.659	131	45	160	0.282	-1.828229	0.195	0
chr3	129033613	129035120	**H1FX**	-	1507	6.705	349	120	426	0.282	-1.824575	0.097	1
chr4	128802015	128820377	**PLK4**	+	18362	1.121	751	258	915	0.283	-1.823064	0.077	1
chr9	112810877	112934791	**AKAP2**	+	123914	6.324	28138	9725	34276	0.284	-1.817405	0.272	0
chr5	1253286	1295162	**TERT**	-	41876	1.758	2575	890	3137	0.284	-1.816535	0.048	1
chr5	148521053	148639999	**ABLIM3**	+	118946	1.758	7535	2613	9175	0.285	-1.811911	0.102	0
chr10	31608100	31818742	**ZEB1**	+	210642	2.13	16465	5721	20046	0.285	-1.808826	0.196	0
chr12	6957971	6960456	**CDCA3**	-	2485	3.177	280	97	341	0.286	-1.808348	0.13	0
chr17	39182278	39183454	**KRTAP1-5**	-	1176	3.363	135	47	165	0.286	-1.804258	0.17	0
chr10	855483	931702	**LARP4B**	-	76219	2.451	6740	2347	8204	0.286	-1.80526	0.098	1
chr12	116396380	116714991	**MED13L**	-	318611	3.751	44501	15481	54174	0.286	-1.807058	0.417	0
chr3	196769430	197025447	**DLG1**	-	256017	2.094	19819	6924	24118	0.287	-1.800404	0.235	0
chr11	61891444	61920635	**INCENP**	+	29191	1.33	1378	481	1677	0.287	-1.800337	0.054	1
chr1	56960418	57045257	**PPAP2B**	-	84839	4.525	13941	4869	16964	0.287	-1.800621	0.168	0
chr16	70721341	70835061	**VAC14**	-	113720	1.717	6835	2389	8318	0.287	-1.799818	0.083	1
chr12	100967488	101018685	**GAS2L3**	+	51197	1.681	3090	1087	3757	0.289	-1.788571	0.066	1
chr7	42000547	42276618	**GLI3**	-	276071	1.321	13155	4639	15993	0.29	-1.785355	0.161	0
chr6	45296053	45518819	**RUNX2**	+	222766	1.753	14248	5043	17316	0.291	-1.779746	0.18	0
chr19	4909509	4962165	**UHRF1**	+	52656	1.579	2959	1048	3596	0.291	-1.778437	0.063	1
chr6	74405507	74538041	**CD109**	+	132534	1.396	6650	2361	8079	0.292	-1.774452	0.102	0
chrX	9431334	9687780	**TBL1X**	+	256446	1.099	10167	3610	12352	0.292	-1.774444	0.138	0
chr6	27805657	27806117	**HIST1H2AK**	-	460	1.71	28	10	34	0.293	-1.770743	0.411	0
chr12	60083125	60175408	**SLC16A7**	+	92283	1.289	4404	1566	5351	0.293	-1.772368	0.097	1
chr4	1723216	1746905	**TACC3**	+	23689	1.856	1565	556	1902	0.293	-1.772546	0.06	1
chr6	33665345	33679504	**C6orf125**	-	14159	1.023	517	184	628	0.294	-1.76616	0.1	0
chr12	2162415	2807115	**CACNA1C**	+	644700	2.551	59109	21101	71778	0.294	-1.766198	0.477	0
chr10	133747959	133770053	**PPP2R2D**	+	22094	1.113	880	314	1068	0.294	-1.764631	0.074	1
chr18	25530929	25757445	**CDH2**	-	226516	5.189	44546	15937	54082	0.295	-1.762763	0.449	0
chr15	99192760	99507759	**IGF1R**	+	314999	1.755	20241	7246	24572	0.295	-1.761666	0.246	0
chr11	95711439	96076344	**MAML2**	-	364905	4.085	55503	19849	67388	0.295	-1.763435	0.492	0
chr17	29421944	29704695	**NF1**	+	282751	1.609	16731	5991	20311	0.295	-1.761387	0.218	0
chr11	12695968	12966284	**TEAD1**	+	270316	4.244	41800	14969	50744	0.295	-1.761247	0.396	0
chr5	169290718	169407744	**FAM196B**	-	117026	2.195	9443	3396	11459	0.296	-1.754372	0.149	0
chr7	8008422	8128709	**GLCCI1**	+	120287	1.18	5155	1853	6255	0.296	-1.755329	0.098	1
chr5	176732500	176739292	**MXD3**	-	6792	1.368	328	118	399	0.296	-1.755634	0.131	0
chr17	76170159	76183285	**TK1**	-	13126	2.742	1274	458	1546	0.296	-1.753954	0.061	1
chr10	27443752	27475848	**MASTL**	+	32096	1.004	1165	419	1413	0.297	-1.753842	0.072	1
chr4	99391517	99579812	**TSPAN5**	-	188295	4.271	29169	10524	35384	0.297	-1.749415	0.313	0

chr10	21823100	22032559	**MLLT10**	+	209459	1.915	14449	5220	17526	0.298	-1.747316	0.184	0
chr4	142949181	143767604	**INPP4B**	-	818423	2.287	67119	24361	81372	0.299	-1.739949	0.523	0
chr6	42192668	42419783	**TRERF1**	-	227115	1.831	14887	5400	18049	0.299	-1.740895	0.186	0
chr5	148206155	148208197	**ADRB2**	+	2042	1.484	106	38	129	0.3	-1.738388	0.233	0
chr8	23386362	23430063	**SLC25A37**	+	43701	1.331	2061	751	2497	0.301	-1.732405	0.066	1
chr17	76000317	76104916	**TNRC6C**	+	104599	1.01	3836	1400	4648	0.301	-1.730317	0.089	1
chr11	58389023	58390145	**ZFP91-CNTF**	+	1122	3.448	141	51	171	0.301	-1.732887	0.233	0
chr10	58117198	58121034	**ZWINT**	-	3836	2.261	308	112	374	0.301	-1.734106	0.143	0
chr11	63742078	63744015	**COX8A**	+	1937	7.355	500	182	605	0.302	-1.729064	0.101	0
chr16	23690200	23701688	**PLK1**	+	11488	3.639	1489	545	1804	0.302	-1.726401	0.068	1
chr15	32907690	32931868	**ARHGAP11A**	+	24178	1.391	1226	450	1485	0.303	-1.720545	0.082	1
chr12	89981825	90049844	**ATP2B1**	-	68019	2.676	6470	2376	7834	0.303	-1.720871	0.108	0
chr12	96051582	96184536	**NTN4**	-	132954	3.208	15806	5796	19143	0.303	-1.723645	0.229	0
chr3	73110809	73112471	**EBLN2**	+	1662	3.565	213	78	258	0.304	-1.715833	0.192	0
chr3	65339905	66024509	**MAGI1**	-	684604	1.271	31929	11761	38652	0.304	-1.716448	0.355	0
chr6	157099063	157531913	**ARID1B**	+	432850	1.708	27109	10019	32805	0.305	-1.711093	0.32	0
chr1	203595914	203713209	**ATP2B4**	+	117295	6.805	28860	10641	34933	0.305	-1.714967	0.319	0
chr5	68462836	68474070	**CCNB1**	+	11234	3.594	1461	540	1768	0.305	-1.710854	0.079	1
chr1	156182778	156209868	**PMF1**	+	27090	10.223	9321	3451	11278	0.306	-1.70851	0.107	0
chr4	177604690	177713895	**VEGFC**	-	109205	11.44	45153	16721	54631	0.306	-1.708046	0.431	0
chr12	27849427	27850566	**REP15**	+	1139	2.243	94	35	114	0.307	-1.703881	0.301	0
chr11	109964086	110042566	**ZC3H12C**	+	78480	1.749	4969	1844	6010	0.307	-1.704384	0.108	0
chr10	125505151	125651500	**CPXM2**	-	146349	1.591	8295	3094	10028	0.309	-1.696139	0.133	0
chr7	127292201	127732659	**SND1**	+	440458	2.537	40508	15141	48964	0.309	-1.693193	0.411	0
chr18	46065426	46389586	**CTIF**	+	324160	2.76	32443	12145	39209	0.31	-1.690733	0.359	0
chr6	56322784	56507694	**DST**	-	184910	6.203	42082	15748	50861	0.31	-1.691387	0.432	0
chr11	119205236	119208022	**RNF26**	+	2786	4.165	405	151	490	0.31	-1.690848	0.123	0
chr10	98741040	98745585	**C10orf12**	+	4545	1.667	275	103	332	0.311	-1.687261	0.181	0
chr2	66662531	66799891	**MEIS1**	+	137360	6.994	35556	13348	42959	0.311	-1.686321	0.402	0
chr16	72816785	73092534	**ZFHX3**	-	275749	2.605	26094	9791	31528	0.311	-1.686999	0.314	0
chr4	17812524	17846487	**NCAPG**	+	33963	2.403	2978	1120	3597	0.312	-1.682484	0.098	1
chr16	3022791	3030540	**PKMYT1**	-	7749	1.813	492	185	594	0.312	-1.679774	0.112	0
chr3	99357453	99515158	**COL8A1**	+	157705	2.548	14936	5648	18032	0.313	-1.674581	0.244	0
chr6	17759413	17987854	**KIF13A**	-	228441	2.808	23431	8848	28292	0.313	-1.676866	0.303	0
chr16	64980682	65155919	**CDH11**	-	175237	4.138	26476	10045	31953	0.314	-1.669396	0.329	0
chr14	65877309	66210839	**FUT8**	+	333530	1.379	16877	6398	20370	0.314	-1.670582	0.251	0
chr1	45205489	45233438	**KIF2C**	+	27949	1.514	1512	573	1825	0.314	-1.671155	0.081	1
chr6	148663728	148873184	**SASH1**	+	209456	2.361	17835	6750	21530	0.314	-1.67338	0.246	0
chr7	92234234	92465941	**CDK6**	-	231707	4.488	38231	14517	46135	0.315	-1.668143	0.421	0
chr15	70946892	71055850	**UACA**	-	108958	4.687	18630	7084	22478	0.315	-1.665926	0.264	0
chr16	75327607	75467387	**CFDP1**	-	139780	1.039	5240	1997	6321	0.316	-1.662263	0.118	0
chr10	126676417	126849624	**CTBP2**	-	173207	2.621	16210	6183	19552	0.316	-1.660894	0.224	0
chr5	139927250	139929163	**EIF4EBP3**	+	1913	1.286	86	32	104	0.316	-1.663811	0.289	0
chr6	158402887	158520207	**SYNJ2**	+	117320	2.478	10322	3933	12452	0.316	-1.662626	0.161	0
chr1	10535002	10690815	**PEX14**	+	155813	1.328	7438	2846	8969	0.317	-1.656004	0.142	0
chr3	81538849	81810950	**GBE1**	-	272101	2.423	24088	9228	29042	0.318	-1.654062	0.316	0
chr8	131064350	131455906	**ASAP1**	-	391556	6.334	89733	34456	108159	0.319	-1.650319	0.675	0
chr5	65222383	65376850	**ERBB2IP**	+	154467	5.322	30157	11639	36330	0.32	-1.642097	0.375	0
chr14	61201458	61435398	**MNAT1**	+	233940	1.281	10927	4209	13166	0.32	-1.645003	0.196	0
chr11	28129797	28355054	**METTL15**	+	225257	1.086	9016	3481	10860	0.321	-1.641238	0.184	0
chr10	22823765	23003503	**PIP4K2A**	-	179738	2.856	18076	6994	21771	0.321	-1.638133	0.24	0
chr11	93454679	93455032	**SCARNA9**	+	353	1.104	14	5	17	0.323	-1.629391	0.609	0
chr20	45838380	45985474	**ZMYND8**	-	147094	1.331	7038	2735	8473	0.323	-1.63091	0.146	0
chr13	110959630	111165373	**COL4A2**	+	205743	2.044	15225	5936	18321	0.324	-1.625756	0.242	0
chr9	80335190	80646219	**GNAQ**	-	311029	1.69	19151	7484	23040	0.325	-1.622202	0.288	0
chr13	97874573	98046374	**MBNL2**	+	171801	6.043	38974	15222	46892	0.325	-1.623178	0.461	0
chr5	16662015	16936385	**MYO10**	-	274370	3.298	32920	12853	39609	0.325	-1.623641	0.391	0
chr2	10262694	10271546	**RRM2**	+	8852	3.824	1227	480	1476	0.325	-1.621241	0.106	0
chr3	176738541	176915048	**TBL1XR1**	-	176507	2.39	15421	6031	18552	0.325	-1.621015	0.254	0
chr2	160175489	160473059	**BAZ2B**	-	297570	1.045	11458	4497	13779	0.326	-1.615383	0.221	0
chr7	132469622	132766828	**CHCHD3**	-	297206	1.048	11167	4377	13430	0.326	-1.617376	0.195	0
chr9	118916070	119164600	**PAPPA**	+	248530	1.15	10415	4085	12526	0.326	-1.616457	0.2	0
chr5	172741725	172756506	**STC2**	-	14781	16.531	8435	3304	10145	0.326	-1.618202	0.138	0
chr1	41154751	41157933	**LOC100130557**	-	3182	1.626	186	73	223	0.327	-1.614008	0.24	0
chr13	76194569	76434006	**LMO7**	+	239437	11.535	102184	40390	122781	0.329	-1.60402	0.755	0
chr15	37183221	37393500	**MEIS2**	-	210279	3.998	30476	12052	36617	0.329	-1.603184	0.381	0
chr5	76986994	77072185	**TBCA**	-	85191	2.09	6383	2521	7671	0.329	-1.605138	0.145	0
chr3	10327437	10335133	**GHRLOS**	+	7696	1.033	280	111	336	0.33	-1.599755	0.187	0
chr9	134735498	134955253	**MED27**	-	219755	1.221	9615	3814	11549	0.33	-1.598329	0.183	0
chr1	170633312	170708541	**PRRX1**	+	75229	4.936	13256	5257	15922	0.33	-1.598613	0.22	0
chr1	178062863	178448648	**RASAL2**	+	385785	1.848	25932	10269	31153	0.33	-1.600959	0.35	0

chr1	103342022	103574052	**COL11A1**	-	232030	6.4	55899	22309	67096	0.332	-1.588605	0.576	0
chr3	123331142	123603149	**MYLK**	-	272007	6.841	65946	26276	79169	0.332	-1.591166	0.57	0
chr8	62413114	62627199	**ASPH**	-	214085	7.826	61018	24402	73223	0.333	-1.585264	0.571	0
chr13	41506054	41593508	**ELF1**	-	87454	1.835	5908	2359	7091	0.333	-1.587488	0.166	0
chr4	40812043	41216635	**APBB2**	-	404592	1.613	23616	9473	28330	0.334	-1.580463	0.334	0
chr10	62538088	62554610	**CDK1**	+	16522	2.757	1639	657	1967	0.334	-1.581832	0.108	0
chr15	75931425	75932664	**IMP3**	-	1239	5.132	223	89	268	0.334	-1.58378	0.216	0
chr10	64926987	65225722	**JMJD1C**	-	298735	2.31	25297	10140	30350	0.334	-1.581614	0.363	0
chr7	31792631	32338383	**PDE1C**	-	545752	2.929	58759	23537	70500	0.334	-1.582688	0.57	0
chr1	21132784	21503381	**EIF4G3**	-	370597	1.499	20263	8144	24303	0.335	-1.577235	0.315	0
chr19	46386865	46389376	**IRF2BP1**	-	2511	1.374	119	48	143	0.335	-1.576979	0.267	0
chr1	120454175	120612317	**NOTCH2**	-	158142	1.683	9615	3876	11528	0.336	-1.572293	0.201	0
chr10	101635333	101769676	**DNMBP**	-	134343	3.218	15655	6322	18765	0.337	-1.569593	0.27	0
chr15	48700502	48937985	**FBN1**	-	237483	5.129	45108	18213	54073	0.337	-1.569938	0.507	0
chr5	130977406	131132756	**FNIP1**	-	155350	3.338	18324	7405	21963	0.337	-1.568546	0.273	0
chr12	27677044	27848497	**PPFIBP1**	+	171453	2.488	15690	6344	18805	0.337	-1.567561	0.288	0
chr20	39807088	39928739	**ZHX3**	-	121651	2.576	11421	4616	13689	0.337	-1.568065	0.231	0
chr3	15708743	15901053	**ANKRD28**	-	192310	3.729	26110	10586	31284	0.338	-1.563207	0.372	0
chr2	202671197	202758263	**CDK15**	+	87066	1.837	5794	2348	6943	0.338	-1.564028	0.159	0
chr8	141668480	142011412	**PTK2**	-	342932	2.232	27836	11303	33347	0.339	-1.56085	0.383	0
chr3	67410883	67705038	**SUCLG2**	-	294155	1.771	18676	7589	22372	0.339	-1.559579	0.292	0
chr8	124332090	124408705	**ATAD2**	-	76615	1.492	4142	1688	4960	0.34	-1.55449	0.144	0
chr2	122095351	122407052	**CLASP1**	-	311701	1.874	21150	8617	25327	0.34	-1.555382	0.325	0
chr1	109419602	109473044	**GPSM2**	+	53442	1.147	2220	905	2658	0.34	-1.554508	0.125	0
chr7	130565750	130598069	**LOC646329**	-	32319	41.217	47413	19318	56779	0.34	-1.555414	0.491	0
chr5	142657495	142815077	**NR3C1**	-	157582	3.623	20982	8535	25132	0.34	-1.557929	0.337	0
chr2	163027199	163100045	**FAP**	-	72846	2.63	6860	2803	8212	0.341	-1.550802	0.168	0
chr19	39897486	39900045	**ZFP36**	+	2559	46.1	4044	1649	4843	0.341	-1.553908	0.106	0
chr1	155112366	155112996	**DPM3**	-	630	1.925	41	17	50	0.342	-1.546202	0.411	0
chr9	127019884	127114719	**NEK6**	+	94835	4.115	13703	5602	16403	0.342	-1.549745	0.229	0
chr22	46067677	46241187	**ATXN10**	+	173510	5.247	32855	13498	39308	0.343	-1.542008	0.42	0
chr17	18874380	18908060	**FAM83G**	-	33680	2.377	2809	1152	3361	0.343	-1.543672	0.109	0
chr15	51236325	51238762	**LOC100132724**	+	2437	1.042	92	37	110	0.343	-1.542947	0.359	0
chr17	28443833	28513486	**NSRP1**	+	69653	2.212	5565	2282	6659	0.343	-1.544491	0.161	0
chr4	7760439	7941653	**AFAP1**	-	181214	2.711	17568	7237	21012	0.344	-1.537705	0.29	0
chr12	93166284	93323107	**EEA1**	-	156823	1.578	8988	3695	10752	0.344	-1.540879	0.213	0
chr8	23154409	23261722	**LOXL2**	-	107313	6.307	24176	9946	28920	0.344	-1.539899	0.344	0
chr3	154797435	154901518	**MME**	+	104083	1.716	6239	2564	7464	0.344	-1.541254	0.145	0
chr12	78225068	78606790	**NAV3**	+	381722	3.375	48117	19824	57548	0.344	-1.537478	0.552	0
chr1	197473878	197744623	**DENND1B**	-	270745	1.234	12329	5081	14745	0.345	-1.537072	0.269	0
chr11	48002109	48192394	**PTPRJ**	+	190285	1.283	8893	3670	10633	0.345	-1.53472	0.218	0
chr1	178694299	178889237	**RALGPS2**	+	194938	2.105	15000	6192	17936	0.345	-1.534267	0.289	0
chr3	158991035	159615155	**SCHIP1**	+	624120	1.012	22837	9421	27310	0.345	-1.535412	0.346	0
chr7	84624871	84751247	**SEMA3D**	-	126376	1.697	7869	3244	9410	0.345	-1.536215	0.21	0
chr12	49716970	49725514	**TROAP**	+	8544	1.305	390	161	466	0.345	-1.534059	0.175	0
chr13	52158483	52336171	**WDFY2**	+	177688	2.417	15427	6370	18446	0.345	-1.533871	0.276	0
chr19	10244021	10305755	**DNMT1**	-	61734	2.098	4587	1899	5483	0.346	-1.529489	0.141	0
chr2	197063976	197457335	**HECW2**	-	393359	1.456	20685	8577	24721	0.347	-1.527156	0.33	0
chr13	114747193	114898095	**RASA3**	-	150902	1.962	10398	4314	12425	0.347	-1.5261	0.204	0
chr13	98795433	99102023	**FARP1**	+	306590	1.749	19390	8070	23163	0.348	-1.521222	0.323	0
chr22	46481876	46509808	**MIRLET7BHG**	+	27932	5.694	5565	2313	6649	0.348	-1.523511	0.144	0
chr9	134305476	134375575	**PRRC2B**	+	70099	2.358	5902	2451	7052	0.348	-1.524502	0.163	0
chr1	41492870	41707815	**SCMH1**	-	214945	1.083	8456	3516	10103	0.348	-1.522413	0.214	0
chr20	42543491	42698254	**TOX2**	+	154763	1.133	6246	2597	7462	0.348	-1.522517	0.168	0
chr4	119201192	119273922	**PRSS12**	-	72730	2.324	6278	2614	7499	0.349	-1.520184	0.207	0
chr17	40724328	40729747	**PSMC3IP**	-	5419	1.164	221	92	265	0.349	-1.519528	0.241	0
chr3	105085556	105295757	**ALCAM**	+	210201	3.931	30698	12814	36659	0.35	-1.51642	0.449	0
chrX	122318095	122624766	**GRIA3**	+	306671	1.218	13519	5648	16143	0.35	-1.515104	0.272	0
chr17	35441926	35766902	**ACACA**	-	324976	1.07	12333	5171	14721	0.351	-1.509234	0.241	0
chr17	1963132	2207069	**SMG6**	-	243937	1.486	12950	5426	15458	0.351	-1.510251	0.254	0
chr14	61447831	61550451	**SLC38A6**	+	102620	1.127	4065	1707	4850	0.352	-1.506321	0.136	0
chr6	26273203	26273640	**HIST1H2BI**	+	437	2.246	35	15	42	0.353	-1.501853	0.499	0
chr5	176560079	176727214	**NSD1**	+	167135	1.465	8866	3733	10577	0.353	-1.502504	0.223	0
chr6	4706392	4955778	**CDYL**	+	249386	1.017	9123	3848	10882	0.354	-1.499539	0.221	0
chr2	189157389	189460652	**GULP1**	+	303263	2.883	32570	13751	38844	0.354	-1.498155	0.471	0
chr3	47892179	48130769	**MAP4**	-	238590	2.799	24299	10266	28976	0.354	-1.496987	0.385	0
chr1	47715810	47779819	**STIL**	-	64009	1.034	2393	1011	2854	0.354	-1.496413	0.144	0
chr8	99466860	99837909	**STK3**	-	371049	1.033	13965	5913	16648	0.355	-1.493248	0.292	0
chr17	65821779	65980494	**BPTF**	+	158715	1.425	8265	3503	9853	0.356	-1.49185	0.229	0
chr3	119540801	119813264	**GSK3B**	-	272463	2.507	24764	10504	29517	0.356	-1.4906	0.39	0
chr6	26188937	26189304	**HIST1H4D**	-	367	3.284	43	18	52	0.356	-1.49173	0.477	0

chr1	180257351	180472022	**ACBD6**	-	214671	1.351	10474	4461	12478	0.358	-1.483861	0.248	0
chr1	53971905	54199877	**GLIS1**	-	227972	1.512	12208	5207	14542	0.358	-1.481634	0.249	0
chr2	20448452	20527144	**PUM2**	-	78692	2.923	8357	3562	9955	0.358	-1.482549	0.229	0
chr5	86564069	86687743	**RASA1**	+	123674	3.233	14508	6189	17281	0.358	-1.48147	0.3	0
chr16	89334034	89556969	**ANKRD11**	-	222935	2.829	22349	9559	26612	0.359	-1.477079	0.348	0
chr15	57210832	57580714	**TCF12**	+	369882	2.253	30561	13064	36394	0.359	-1.478047	0.449	0
chr22	18270415	18507325	**MICAL3**	-	236910	1.391	11640	5000	13854	0.361	-1.47004	0.246	0
chr19	17622431	17632097	**PGLS**	+	9666	1.43	482	207	574	0.361	-1.471199	0.176	0
chr16	87863628	87903100	**SLC7A5**	-	39472	5.243	7175	3087	8537	0.362	-1.46727	0.175	0
chr6	37787306	38122399	**ZFAND3**	+	335093	2.804	34102	14683	40575	0.362	-1.466439	0.469	0
chr8	37553300	37556396	**ZNF703**	+	3096	4.662	486	209	578	0.362	-1.467693	0.15	0
chr13	95672082	95953687	**ABCC4**	-	281605	1.134	11320	4887	13464	0.363	-1.461928	0.249	0
chr11	9160374	9286873	**DENND5A**	-	126499	2.129	9689	4179	11526	0.363	-1.463706	0.246	0
chr4	95373037	95589377	**PDLIM5**	+	216340	4.29	33819	14595	40227	0.363	-1.462639	0.473	0
chr20	33890368	33999945	**UQCC**	-	109577	1.198	4687	2023	5575	0.363	-1.462195	0.171	0
chr4	159587830	159593202	**C4orf46**	-	5372	1.796	342	148	407	0.364	-1.457547	0.233	0
chr1	235824344	236030220	**LYST**	-	205876	1.223	8849	3843	10518	0.365	-1.452308	0.218	0
chr9	97488950	97849500	**C9orf3**	+	360550	1.996	25863	11252	30733	0.366	-1.449612	0.408	0
chr11	72547789	72853143	**FCHSD2**	-	305354	1.938	21759	9458	25859	0.366	-1.45097	0.397	0
chr3	108268717	108308491	**KIAA1524**	-	39774	1.556	2221	965	2640	0.366	-1.451928	0.151	0
chr14	95873603	95876427	**LINC00341**	-	2824	1.252	123	53	146	0.366	-1.449744	0.323	0
chr10	90973325	91011660	**LIPA**	-	38335	2.369	3239	1408	3849	0.366	-1.451144	0.157	0
chr2	54683453	54898583	**SPTBN1**	+	215130	2.346	18115	7874	21529	0.366	-1.451089	0.337	0
chr1	222841354	222885864	**AIDA**	-	44510	1.114	1787	779	2123	0.367	-1.445328	0.156	0
chr9	140446308	140447007	**MRPL41**	+	699	3.552	85	37	101	0.367	-1.446518	0.353	0
chr11	4115923	4160106	**RRM1**	+	44183	1.133	1813	790	2154	0.367	-1.446724	0.16	0
chr17	60556385	60692841	**TLK2**	+	136456	1.458	7237	3155	8597	0.367	-1.445992	0.232	0
chr15	85923870	86292586	**AKAP13**	+	368716	3.11	41468	18134	49246	0.368	-1.44131	0.518	0
chr1	20915443	20945400	**CDA**	+	29957	1.286	1382	604	1642	0.368	-1.441273	0.155	0
chr9	140513443	140730578	**EHMT1**	+	217135	1.365	10478	4583	12443	0.368	-1.440929	0.246	0
chr7	27870192	28220437	**JAZF1**	-	350245	2.415	30760	13440	36533	0.368	-1.4426	0.456	0
chr5	34656432	34832717	**RAI14**	+	176285	2.85	18051	7894	21436	0.368	-1.441176	0.341	0
chr2	201170603	201346986	**SPATS2L**	+	176383	3.056	19637	8591	23318	0.368	-1.440491	0.376	0
chr3	194123402	194188968	**ATP13A3**	-	65566	3.219	7506	3290	8911	0.369	-1.43731	0.218	0
chr20	37554954	37581703	**FAM83D**	+	26749	2.378	2254	988	2676	0.369	-1.436743	0.149	0
chr13	49550047	49783915	**FNDC3A**	+	233868	2.435	20835	9119	24740	0.369	-1.439931	0.39	0
chr6	26217147	26217711	**HIST1H2AE**	+	564	6.078	122	53	145	0.369	-1.437855	0.356	0
chr5	77298149	77590528	**AP3B1**	-	292379	1.457	15539	6829	18442	0.37	-1.433159	0.337	0
chr10	26727265	26856732	**APBB1IP**	+	129467	2.27	10643	4677	12632	0.37	-1.433451	0.279	0
chr15	64457715	64648442	**CSNK1G1**	-	190727	1.077	7413	3254	8799	0.37	-1.434946	0.231	0
chr18	43427573	43547305	**EPG5**	-	119732	2.65	11363	4993	13486	0.37	-1.433274	0.275	0
chr22	35796115	35820495	**MCM5**	+	24380	1.152	992	435	1177	0.37	-1.434388	0.153	0
chr12	63037762	63328665	**PPM1H**	-	290903	1.013	10891	4781	12928	0.37	-1.434941	0.302	0
chr5	137801180	137805004	**EGR1**	+	3824	47.122	6158	2712	7306	0.371	-1.429386	0.166	0
chr6	143072603	143266338	**HIVEP2**	-	193735	1.439	10060	4431	11936	0.371	-1.42939	0.269	0
chr2	114737145	114764887	**LOC440900**	+	27742	1.656	1628	717	1931	0.371	-1.429159	0.149	0
chr12	53645369	53648190	**MFSD5**	+	2821	2.459	240	106	285	0.371	-1.42872	0.256	0
chr5	102201526	102366808	**PAM**	+	165282	3.134	18886	8308	22413	0.371	-1.431657	0.374	0
chr2	43449540	43453745	**ZFP36L2**	-	4205	29.711	4303	1895	5106	0.371	-1.429789	0.15	0
chr16	53737874	54148379	**FTO**	+	410505	1.01	14931	6589	17712	0.372	-1.426523	0.32	0
chr7	77428108	77586821	**PHTF2**	+	158713	1.435	8367	3695	9925	0.372	-1.425373	0.268	0
chr4	122737598	122745088	**CCNA2**	-	7490	4.649	1258	556	1492	0.373	-1.422813	0.17	0
chr2	238232654	238322850	**COL6A3**	-	90196	9.956	31372	13891	37198	0.373	-1.421065	0.432	0
chr5	175815783	175816751	**HIGD2A**	+	968	1.551	51	22	61	0.373	-1.424481	0.429	0
chr1	173900351	173962210	**RC3H1**	-	61859	1.715	3877	1714	4598	0.373	-1.423873	0.201	0
chr5	95220801	95297775	**ELL2**	-	76974	23.734	68632	30449	81359	0.374	-1.417909	0.707	0
chr11	126293395	126870766	**KIRREL3**	-	577371	1.565	32959	14621	39071	0.374	-1.418017	0.49	0
chr2	62900985	63273621	**EHBP1**	+	372636	2.13	29680	13196	35175	0.375	-1.414438	0.496	0
chr2	85764589	85766009	**LOC100630918**	-	1420	1.027	51	22	61	0.375	-1.41327	0.454	0
chr2	61414589	61697849	**USP34**	-	283260	1.626	16671	7401	19761	0.375	-1.416816	0.348	0
chr20	48807119	48809227	**CEBPB**	+	2108	5.139	366	163	434	0.376	-1.41234	0.201	0
chr11	122943032	123066007	**CLMP**	-	122975	2.612	11381	5071	13484	0.376	-1.410689	0.274	0
chr15	40092930	40213093	**GPR176**	-	120163	4.946	21624	9643	25617	0.376	-1.409504	0.406	0
chr5	140805852	140807825	**PCDHGB8P**	+	1973	2.257	155	69	184	0.376	-1.410745	0.319	0
chr5	98104998	98132198	**RGMB**	+	27200	26.183	25431	11334	30131	0.376	-1.410606	0.417	0
chr6	7107829	7252213	**RREB1**	+	144384	3.125	15991	7118	18949	0.376	-1.412523	0.32	0
chr5	304290	438405	**AHRR**	+	134115	4.328	20496	9142	24280	0.377	-1.409103	0.363	0
chr2	196521531	196602426	**SLC39A10**	+	80895	1.114	3226	1445	3820	0.378	-1.402215	0.18	0
chr9	127115751	127121463	**LOC100129034**	+	5712	3.783	757	340	896	0.38	-1.3969	0.175	0
chr3	30647993	30735633	**TGFBR2**	+	87640	3.238	10212	4588	12087	0.38	-1.397455	0.285	0
chr17	3572089	3572962	**TMEM93**	+	873	2.769	84	37	100	0.38	-1.397445	0.393	0

chr15	56119121	56285835	**NEDD4**	-	166714	1.264	7697	3471	9106	0.381	-1.391581	0.27	0
chr1	65730429	65881552	**DNAJC6**	+	151123	1.367	7405	3347	8757	0.382	-1.38728	0.247	0
chr12	11802787	12048325	**ETV6**	+	245538	2.015	17950	8115	21228	0.382	-1.387212	0.382	0
chr1	249104650	249120154	**SH3BP5L**	-	15504	3.34	1823	823	2157	0.382	-1.389582	0.157	0
chr4	170015406	170192249	**SH3RF1**	-	176843	3.946	25375	11457	30015	0.382	-1.389422	0.448	0
chr10	134351352	134596984	**INPP5A**	+	245632	1.038	9063	4107	10715	0.383	-1.383425	0.261	0
chr4	5712923	5816031	**EVC**	+	103108	2.24	8167	3707	9654	0.384	-1.380683	0.246	0
chr4	17844838	18023483	**LCORL**	-	178645	1.412	9051	4113	10697	0.384	-1.379013	0.276	0
chr11	308106	309410	**IFITM2**	+	1304	5.684	259	118	306	0.385	-1.375278	0.285	0
chr13	39917028	40177356	**LHFP**	-	260328	1.712	16112	7321	19042	0.385	-1.378932	0.363	0
chr11	46417963	46612914	**AMBRA1**	-	194951	1.295	9050	4127	10691	0.386	-1.373145	0.278	0
chr11	33097695	33101000	**LINC00294**	+	3305	1.574	189	86	224	0.386	-1.371515	0.367	0
chr12	58120022	58122139	**LOC100130776**	+	2117	1.48	108	49	128	0.386	-1.374199	0.366	0
chr8	141541263	141645646	**EIF2C2**	-	104383	1.655	6040	2762	7133	0.387	-1.368763	0.213	0
chr17	16946073	17095962	**MPRIP**	+	149889	2.41	12759	5827	15069	0.387	-1.3708	0.306	0
chr11	122526397	122685187	**UBASH3B**	+	158790	1.401	7844	3582	9264	0.387	-1.370958	0.244	0
chr16	2570362	2580955	**AMDHD2**	+	10593	2.615	952	436	1123	0.388	-1.364905	0.158	0
chr3	124481794	124606144	**ITGB5**	-	124350	1.265	5663	2596	6685	0.388	-1.364767	0.238	0
chr9	116638561	116818875	**ZNF618**	+	180314	1.563	10027	4594	11838	0.388	-1.365472	0.285	0
chr3	171757417	172118492	**FNDC3B**	+	361075	3.34	43643	20091	51493	0.39	-1.357774	0.576	0
chr2	10443039	10567743	**HPCAL1**	+	124704	1.088	4718	2169	5568	0.39	-1.360181	0.191	0
chr22	29999544	30094589	**NF2**	+	95045	1.655	5642	2593	6658	0.39	-1.360072	0.237	0
chr12	109176465	109251359	**SSH1**	-	74894	8.593	22769	10479	26866	0.39	-1.358296	0.41	0
chr14	38677203	38682268	**SSTR1**	+	5065	25.947	4585	2111	5410	0.39	-1.357285	0.195	0
chr10	3109711	3178997	**PFKP**	+	69286	1.351	3308	1524	3903	0.391	-1.356129	0.19	0
chr9	134452156	134612925	**RAPGEF1**	-	160769	1.308	7399	3413	8727	0.391	-1.35455	0.244	0
chr4	83550689	83720010	**SCD5**	-	169321	1.077	6637	3066	7827	0.392	-1.352075	0.272	0
chr4	139085247	139163503	**SLC7A11**	-	78256	1.597	4607	2134	5431	0.393	-1.347384	0.258	0
chr18	8717368	8832775	**CCDC165**	+	115407	2.781	11349	5266	13377	0.394	-1.344967	0.303	0
chr13	53029494	53050763	**CKAP2**	+	21269	2.87	2202	1022	2595	0.394	-1.343987	0.206	0
chr16	771157	772590	**FAM173A**	+	1433	1.123	55	25	65	0.394	-1.342881	0.449	0
chr9	3824127	4300035	**GLIS3**	-	475908	1.086	18822	8744	22181	0.394	-1.342922	0.418	0
chr17	1958392	1962981	**HIC1**	+	4589	3.671	573	266	676	0.394	-1.343439	0.195	0
chr13	47127295	47327175	**LRCH1**	+	199880	1.347	9658	4481	11384	0.394	-1.345077	0.3	0
chr8	80831094	80942506	**MRPS28**	-	111412	1.101	4440	2064	5233	0.394	-1.34192	0.24	0
chr2	70314584	70316334	**PCBP1**	+	1750	15.708	958	444	1129	0.394	-1.343888	0.178	0
chr6	158244293	158366109	**SNX9**	+	121816	3.322	14283	6629	16834	0.394	-1.344427	0.331	0
chr15	69706626	69740764	**KIF23**	+	34138	3.153	3872	1800	4562	0.395	-1.341285	0.222	0
chr3	11314009	11599139	**ATG7**	+	285130	1.156	11769	5486	13864	0.396	-1.337511	0.32	0
chr22	24666785	24813708	**SPECC1L**	+	146923	1.611	8488	3958	9998	0.396	-1.336788	0.288	0
chr2	173940564	174132737	**ZAK**	+	192173	1.982	13804	6444	16257	0.396	-1.335059	0.365	0
chr7	134464163	134655480	**CALD1**	+	191317	28.537	202705	94747	238691	0.397	-1.332995	1	0
chr12	11126253	11324224	**PRH1-PRR4**	-	197971	1.395	9998	4672	11774	0.397	-1.33355	0.318	0
chr6	125304513	125404661	**RNF217**	+	100148	1.625	5945	2778	7001	0.397	-1.333098	0.277	0
chr12	51985019	52202299	**SCN8A**	+	217280	1.661	13069	6112	15388	0.397	-1.332084	0.357	0
chr1	19665266	19812066	**CAPZB**	-	146800	4.343	22356	10467	26319	0.398	-1.330169	0.411	0
chr7	148504463	148581441	**EZH2**	-	76978	1.283	3539	1658	4167	0.398	-1.328823	0.22	0
chr11	46402617	46405375	**MDK**	+	2758	1.314	125	58	147	0.398	-1.32859	0.374	0
chr7	104756822	105029341	**SRPK2**	-	272519	1.538	15039	7039	17706	0.398	-1.330714	0.37	0
chr6	147525493	147711612	**STXBP5**	+	186119	2.108	14288	6699	16817	0.398	-1.327738	0.381	0
chr7	116164838	116201239	**CAV1**	+	36401	23.234	31005	14573	36482	0.399	-1.323839	0.526	0
chr2	128056244	128100805	**MAP3K2**	-	44561	1.191	1954	918	2299	0.399	-1.323907	0.237	0
chr19	1275519	1279243	**C19orf24**	+	3724	1.401	179	84	210	0.401	-1.317522	0.325	0
chr10	35535952	35860847	**CCNY**	+	324895	1.076	12454	5878	14646	0.401	-1.316985	0.337	0
chr18	12991360	13125051	**CEP192**	+	133691	1.423	6801	3208	7998	0.401	-1.318086	0.272	0
chr3	128444978	128533641	**RAB7A**	+	88663	4.581	14336	6756	16863	0.401	-1.319522	0.344	0
chr13	48877882	49056026	**RB1**	+	178144	1.281	8173	3858	9612	0.401	-1.31701	0.291	0
chr20	34541538	34543281	**SCAND1**	-	1743	1.346	80	37	94	0.401	-1.319858	0.418	0
chr3	160117429	160152741	**SMC4**	+	35312	6.225	7903	3728	9294	0.401	-1.317897	0.292	0
chr14	105475909	105487425	**CDCA4**	-	11516	2.903	1176	556	1383	0.402	-1.314584	0.195	0
chr13	110801309	110959496	**COL4A1**	-	158187	1.766	10011	4735	11770	0.402	-1.313485	0.317	0
chr6	129204285	129837710	**LAMA2**	+	633425	1.69	39141	18498	46023	0.402	-1.31497	0.583	0
chr19	41725107	41767671	**AXL**	+	42564	6.714	10153	4814	11933	0.403	-1.309504	0.313	0
chr1	230202955	230417875	**GALNT2**	+	214920	1.696	12941	6129	15212	0.403	-1.311432	0.341	0
chr12	11213963	11214893	**TAS2R46**	-	930	1.164	39	18	46	0.403	-1.310759	0.564	0
chr6	34214156	34216904	**C6orf1**	-	2748	2.419	229	108	269	0.404	-1.308536	0.313	0
chr3	112323408	112359977	**CCDC80**	-	36569	3.95	5430	2577	6381	0.404	-1.307879	0.317	0
chr12	6309481	6347437	**CD9**	+	37956	1.625	2145	1018	2521	0.404	-1.307922	0.182	0
chr4	186131283	186285120	**SNX25**	+	153837	1.3	7343	3489	8628	0.404	-1.306004	0.315	0
chr8	116420723	116681228	**TRPS1**	-	260505	1.551	14566	6917	17116	0.404	-1.306994	0.382	0
chr2	176987412	176989645	**HOXD9**	+	2233	1.461	114	54	134	0.405	-1.302727	0.408	0

chr9	91003296	91093622	**SPIN1**	+	90326	2.843	9216	4382	10827	0.405	-1.304859	0.314	0
chr6	20100934	20212670	**MBOAT1**	-	111736	1.962	8112	3865	9528	0.406	-1.30142	0.338	0
chr17	17584786	17714765	**RAI1**	+	129979	2.028	9206	4388	10811	0.406	-1.300644	0.287	0
chr22	36134782	36424585	**RBFOX2**	-	289803	2.618	27332	13024	32102	0.406	-1.30145	0.494	0
chr17	26904582	26926056	**SPAG5**	-	21474	1.306	994	474	1168	0.406	-1.30012	0.218	0
chr2	32582095	32843965	**BIRC6**	+	261870	2.035	19315	9225	22678	0.407	-1.297711	0.441	0
chr1	62920396	63153969	**DOCK7**	-	233573	1.299	10890	5203	12786	0.407	-1.296964	0.337	0
chr6	111620233	111804414	**REV3L**	-	184181	3.056	20232	9672	23751	0.407	-1.296117	0.439	0
chr16	28109315	28223190	**XPO6**	-	113875	3.478	14021	6704	16459	0.407	-1.295652	0.359	0
chr1	167905796	168045083	**DCAF6**	+	139287	1.887	9550	4578	11208	0.408	-1.291761	0.339	0
chr12	15773074	15942510	**EPS8**	-	169436	2.928	18089	8659	21232	0.408	-1.293903	0.439	0
chr5	15500304	15939900	**FBXL7**	+	439596	1.675	26816	12848	31472	0.408	-1.292543	0.506	0
chr6	27782821	27783267	**HIST1H2BM**	+	446	1.861	29	14	35	0.408	-1.292735	0.606	0
chr4	54851665	54853449	**RPL21P44**	-	1784	1.218	77	37	91	0.408	-1.292852	0.479	0
chr13	45006278	45150701	**TSC22D1**	-	144423	1.525	7883	3772	9254	0.408	-1.294478	0.3	0
chr13	50556687	50699677	**DLEU2**	-	142990	1.686	8672	4158	10176	0.409	-1.291001	0.316	0
chr16	86508130	86542466	**LOC400550**	-	34336	2.24	2667	1279	3130	0.409	-1.290485	0.194	0
chr21	16333555	16437126	**NRIP1**	-	103571	4.194	15777	7568	18514	0.409	-1.290583	0.413	0
chr1	214522038	214725024	**PTPN14**	-	202986	3.284	24017	11553	28172	0.41	-1.286002	0.477	0
chr21	45285115	45407475	**AGPAT3**	+	122360	1.147	4913	2368	5761	0.411	-1.282256	0.238	0
chr6	17393735	17558023	**CAP2**	+	164288	2.006	12000	5794	14068	0.412	-1.27983	0.382	0
chr20	43595119	43708593	**STK4**	+	113474	1.562	6279	3035	7360	0.412	-1.278003	0.275	0
chr1	35734567	35887545	**ZMYM4**	+	152978	1.407	7727	3735	9058	0.412	-1.2779	0.311	0
chr7	7222245	7288251	**C1GALT1**	+	66006	1.09	2546	1233	2984	0.413	-1.274962	0.22	0
chr14	50885210	50999376	**MAP4K5**	-	114166	2.788	11561	5602	13548	0.413	-1.274075	0.376	0
chr12	105724413	105765296	**C12orf75**	+	40883	19.749	29456	14278	34515	0.414	-1.273422	0.539	0
chr3	56654159	56717135	**FAM208A**	-	62976	1.429	3248	1577	3806	0.414	-1.270954	0.256	0
chr8	120428551	120436678	**NOV**	+	8127	1.328	370	179	433	0.414	-1.270759	0.279	0
chr18	61420276	61472604	**SERPINB7**	+	52328	2.215	4224	2050	4949	0.414	-1.271595	0.284	0
chr4	56298659	56412997	**CLOCK**	-	114338	1.792	7405	3597	8675	0.415	-1.270035	0.317	0
chr14	24608173	24610797	**FAM158A**	-	2624	1.293	119	58	139	0.415	-1.26825	0.427	0
chr7	44836240	44842716	**PPIA**	+	6476	7.015	1599	778	1873	0.415	-1.267482	0.215	0
chr19	43671894	43690688	**PSG5**	-	18794	3.024	2072	1008	2426	0.415	-1.267129	0.256	0
chr16	74655296	74700779	**RFWD3**	-	45483	2.432	3932	1912	4605	0.415	-1.267816	0.248	0
chr9	33817181	33920401	**UBE2R2**	+	103220	2.052	7572	3678	8871	0.415	-1.270115	0.308	0
chr20	30193085	30194317	**ID1**	+	1232	152.547	6472	3151	7579	0.416	-1.266068	0.249	0
chr19	13261281	13265718	**IER2**	+	4437	12.64	1906	929	2232	0.416	-1.264112	0.176	0
chr2	135011829	135212192	**MGAT5**	+	200363	1.256	9121	4441	10681	0.416	-1.266013	0.345	0
chr3	64079525	64211131	**PRICKLE2**	-	131606	1.548	7314	3564	8564	0.416	-1.264832	0.313	0
chr4	99182526	99365012	**RAP1GDS1**	+	182486	1.662	11053	5382	12943	0.416	-1.265774	0.38	0
chr17	47074773	47133507	**IGF2BP1**	+	58734	1.701	3564	1741	4172	0.417	-1.260905	0.251	0
chr5	74666927	74807806	**COL4A3BP**	-	140879	2.097	10514	5146	12304	0.418	-1.257511	0.344	0
chr15	59428562	59665071	**MYO1E**	-	236509	1.729	14468	7091	16927	0.419	-1.255235	0.386	0
chr6	15246526	15522253	**JARID2**	+	275727	2.206	21374	10507	24997	0.42	-1.250317	0.447	0
chr22	20861885	20941919	**MED15**	+	80034	4.616	12994	6381	15199	0.42	-1.252125	0.365	0
chr11	35684352	35830930	**TRIM44**	+	146578	1.287	6756	3322	7901	0.42	-1.250143	0.31	0
chr15	75966662	76005189	**CSPG4**	-	38527	1.812	2419	1190	2829	0.421	-1.248206	0.211	0
chr17	7255207	7258262	**KCTD11**	+	3055	1.639	173	85	202	0.421	-1.247035	0.375	0
chr6	151186814	151423023	**MTHFD1L**	+	236209	2.374	20039	9859	23432	0.421	-1.248963	0.453	0
chr4	101944586	102268628	**PPP3CA**	-	324042	1.517	17808	8769	20821	0.421	-1.247504	0.453	0
chr17	79609348	79615779	**TSPAN10**	+	6431	1.783	391	192	458	0.421	-1.248651	0.279	0
chr11	62518434	62521656	**ZBTB3**	-	3222	1.122	125	61	146	0.421	-1.247263	0.411	0
chr6	485137	693109	**EXOC2**	-	207972	1.018	7545	3719	8821	0.422	-1.245782	0.315	0
chr6	26240653	26241021	**HIST1H4F**	+	368	3.589	46	22	54	0.422	-1.244221	0.541	0
chr22	43265771	43411184	**PACSIN2**	-	145413	1.456	7497	3696	8764	0.422	-1.245458	0.309	0
chr15	67358194	67487533	**SMAD3**	+	129339	6.689	30023	14811	35094	0.422	-1.244515	0.499	0
chr3	16357351	16555222	**RFTN1**	-	197871	1.302	9191	4546	10739	0.423	-1.240081	0.341	0
chr1	155305051	155532324	**ASH1L**	-	227273	1.252	10330	5113	12069	0.424	-1.238947	0.381	0
chr2	25016174	25045245	**CENPO**	+	29071	1.455	1484	735	1734	0.424	-1.23835	0.224	0
chr9	20344967	20622514	**MLLT3**	-	277547	1.247	12620	6254	14742	0.424	-1.23688	0.417	0
chrX	21958714	22012955	**SMS**	+	54241	2.945	5681	2814	6636	0.424	-1.237436	0.291	0
chr5	71515235	71616084	**MRPS27**	-	100849	1.23	4452	2210	5199	0.425	-1.233838	0.288	0
chr2	171847332	172087824	**TLK1**	-	240492	1.507	13062	6489	15252	0.425	-1.232795	0.408	0
chr16	88875876	88878342	**APRT**	-	2466	1.525	129	64	151	0.426	-1.230412	0.412	0
chr8	48685668	48872743	**PRKDC**	-	187075	1.437	9571	4762	11174	0.426	-1.2305	0.349	0
chr2	17845078	17935096	**SMC6**	-	90018	1.004	3242	1611	3786	0.426	-1.232353	0.27	0
chr11	46765083	46867859	**CKAP5**	-	102776	1.79	6602	3292	7706	0.427	-1.226752	0.321	0
chr2	174937174	175113365	**OLA1**	-	176191	1.185	7511	3741	8768	0.427	-1.228532	0.335	0
chr15	77400497	77712446	**PEAK1**	-	311949	2.43	27724	13829	32356	0.427	-1.226344	0.559	0
chr7	151832009	152133090	**MLL3**	-	301081	1.273	13788	6882	16090	0.428	-1.225157	0.418	0
chr14	55034329	55260033	**SAMD4A**	+	225704	4.546	36717	18352	42839	0.428	-1.222935	0.591	0

chr11	65657874	65659106	**CCDC85B**	+	1232	3.474	143	71	167	0.429	-1.221368	0.373	0
chr14	31363004	31495607	**STRN3**	-	132603	1.944	9268	4637	10812	0.429	-1.221184	0.367	0
chr1	227177565	227505826	**CDC42BPA**	-	328261	1.774	21153	10607	24669	0.43	-1.217638	0.502	0
chr3	73431651	73674072	**PDZRN3**	-	242421	1.679	14698	7370	17141	0.43	-1.217605	0.436	0
chr7	102781716	102782850	**RPL19P12**	-	1134	2.444	95	48	111	0.43	-1.219085	0.453	0
chr2	231577556	231685790	**CAB39**	+	108234	5.63	21885	10988	25517	0.431	-1.215464	0.495	0
chr7	23349827	23509995	**IGF2BP3**	-	160168	1.145	6512	3273	7591	0.431	-1.213723	0.314	0
chr5	140787769	140790232	**PCDHGB6**	+	2463	1.406	121	60	141	0.431	-1.21271	0.443	0
chr1	52373627	52456436	**RAB3B**	-	82809	1.671	5023	2525	5856	0.431	-1.213709	0.32	0
chr19	44235300	44259142	**SMG9**	-	23842	1.263	1058	531	1234	0.431	-1.215451	0.248	0
chr18	12446510	12657912	**SPIRE1**	-	211402	1.04	7895	3969	9204	0.431	-1.213485	0.347	0
chr2	242615156	242626383	**DTYMK**	-	11227	1.618	634	319	739	0.432	-1.211017	0.28	0
chr2	238536223	238690290	**LRRFIP1**	+	154067	2.693	14614	7363	17030	0.432	-1.209699	0.407	0
chrX	123094474	123236505	**STAG2**	+	142031	1.426	7284	3665	8491	0.432	-1.212064	0.341	0
chr4	146681887	146859607	**ZNF827**	-	177720	1.536	9915	4990	11557	0.432	-1.21165	0.392	0
chr5	138089106	138270723	**CTNNA1**	+	181617	2.76	17923	9036	20885	0.433	-1.208669	0.457	0
chr3	135684514	135866752	**PPP2R3A**	+	182238	1.958	12982	6552	15125	0.433	-1.20693	0.434	0
chr12	11174270	11175170	**TAS2R19**	-	900	1.62	52	26	61	0.433	-1.207008	0.576	0
chr6	41034530	41040188	**C6orf130**	-	5658	1.048	209	106	244	0.434	-1.205276	0.407	0
chr19	18367905	18385319	**KIAA1683**	-	17414	1.092	665	336	775	0.434	-1.205824	0.28	0
chr6	27777841	27778314	**HIST1H3H**	+	473	3.174	55	27	64	0.435	-1.200866	0.607	0
chr16	67973786	67977376	**LCAT**	-	3590	2.161	267	135	311	0.435	-1.200918	0.349	0
chr4	14113591	14141676	**LOC152742**	+	28085	2.37	2355	1193	2742	0.435	-1.200771	0.258	0
chr12	98909350	98944157	**TMPO**	+	34807	2.164	2684	1360	3125	0.435	-1.200507	0.273	0
chr10	75183336	75193319	**ZMYND17**	-	9983	1.05	375	190	437	0.435	-1.199477	0.37	0
chr1	78030189	78148343	**ZZZ3**	-	118154	3.252	13749	6960	16011	0.435	-1.201818	0.423	0
chr7	27565058	27702620	**HIBADH**	-	137562	1.023	5114	2596	5953	0.436	-1.197465	0.332	0
chr5	52285155	52390609	**ITGA2**	+	105454	1.736	6534	3314	7607	0.436	-1.198543	0.329	0
chr1	198126107	198291548	**NEK7**	+	165441	6.475	39451	20034	45924	0.436	-1.196767	0.66	0
chr19	3359560	3469215	**NFIC**	+	109655	1.81	6926	3517	8062	0.436	-1.196563	0.31	0
chr1	202300784	202311094	**UBE2T**	-	10310	1.301	478	242	557	0.436	-1.199005	0.336	0
chr3	179370932	179507189	**USP13**	+	136257	1.312	6383	3239	7432	0.436	-1.197937	0.329	0
chr12	110810704	110841535	**ANAPC7**	-	30831	1.417	1540	783	1792	0.437	-1.194797	0.253	0
chr6	12012723	12165232	**HIVEP1**	+	152509	1.022	5670	2883	6600	0.437	-1.194657	0.344	0
chr12	81191170	81331694	**LIN7A**	-	140524	1.17	5891	2998	6856	0.437	-1.193406	0.329	0
chr12	46123619	46301819	**ARID2**	+	178200	1.083	6969	3549	8109	0.438	-1.191842	0.357	0
chr1	39547088	39952810	**MACF1**	+	405722	1.833	26737	13649	31099	0.439	-1.188001	0.55	0
chr4	80822770	80994477	**ANTXR2**	-	171707	1.638	10031	5131	11665	0.44	-1.184816	0.386	0
chr12	111890017	112037480	**ATXN2**	-	147463	2.78	14697	7512	17093	0.44	-1.186041	0.447	0
chr3	47057897	47205467	**SETD2**	-	147570	2.25	12008	6143	13963	0.44	-1.184448	0.428	0
chr11	111473169	111597632	**SIK2**	+	124463	1.153	5184	2652	6028	0.44	-1.184814	0.332	0
chr17	74380689	74383941	**SPHK1**	+	3252	8.387	934	477	1086	0.44	-1.184988	0.24	0
chr4	41362803	41702061	**LIMCH1**	+	339258	1.507	18941	9709	22018	0.441	-1.181261	0.529	0
chr3	45636322	45722755	**LIMD1**	+	86433	1.385	4204	2155	4886	0.441	-1.180948	0.288	0
chr16	11439310	11445617	**RMI2**	+	6307	1.025	225	115	261	0.441	-1.182652	0.391	0
chr4	141786724	142054616	**RNF150**	-	267892	1.967	19622	10067	22807	0.441	-1.179845	0.547	0
chr19	47634079	47713893	**SAE1**	+	79814	1.998	5635	2889	6551	0.441	-1.181078	0.313	0
chr1	26210676	26233368	**STMN1**	-	22692	3.726	3001	1539	3489	0.441	-1.180691	0.285	0
chr7	24836163	25019760	**OSBPL3**	-	183597	3.46	22639	11635	26307	0.442	-1.176892	0.511	0
chr13	24144508	24250244	**TNFRSF19**	+	105736	1.222	4645	2386	5397	0.442	-1.177276	0.323	0
chr16	84733554	84813527	**USP10**	+	79973	2.132	6046	3112	7024	0.443	-1.17428	0.329	0
chr12	45609769	45834187	**ANO6**	+	224418	2.4	19374	9981	22505	0.444	-1.172875	0.501	0
chr4	79697531	79833341	**BMP2K**	+	135810	1.86	8991	4638	10442	0.444	-1.170845	0.379	0
chr3	52579367	52719866	**PBRM1**	-	140499	1.355	6821	3519	7921	0.444	-1.170363	0.357	0
chr16	70284133	70285833	**EXOSC6**	-	1700	2.534	145	75	169	0.445	-1.167564	0.407	0
chr4	120980578	120988013	**MAD2L1**	-	7435	1.605	419	216	486	0.445	-1.169129	0.346	0
chr13	28712642	28869475	**PAN3**	+	156833	2.16	12182	6289	14146	0.445	-1.16949	0.431	0
chr21	27252860	27543446	**APP**	-	290586	2.174	22426	11601	26034	0.446	-1.166153	0.509	0
chr11	69455872	69469242	**CCND1**	+	13370	14.992	6929	3585	8043	0.446	-1.16563	0.313	0
chr8	124238428	124238739	**ZHX1-C8ORF76**	-	311	2.738	29	15	33	0.446	-1.163332	0.606	0
chr3	185361526	185542827	**IGF2BP2**	-	181301	2.572	16682	8650	19359	0.447	-1.162189	0.474	0
chr13	99852678	100038753	**UBAC2**	+	186075	1.024	6720	3484	7798	0.447	-1.162246	0.337	0
chr5	71403117	71505397	**MAP1B**	+	102280	6.014	22037	11474	25558	0.449	-1.155382	0.524	0
chr6	30655825	30658769	**NRM**	-	2944	1.128	115	60	133	0.449	-1.15386	0.473	0
chr14	92980124	93155334	**RIN3**	+	175210	1.119	6878	3587	7975	0.45	-1.152723	0.338	0
chr2	71558888	71662189	**ZNF638**	+	103301	1.851	6844	3574	7934	0.451	-1.150234	0.371	0
chr5	31400601	31532282	**DROSHA**	-	131681	1.025	4795	2511	5556	0.452	-1.145596	0.329	0
chr3	57994126	58157982	**FLNB**	+	163856	1.553	9031	4729	10465	0.452	-1.145958	0.39	0
chr2	74699084	74699942	**MRPL53**	-	858	4.18	123	64	142	0.452	-1.144255	0.454	0
chr5	153570294	153800543	**GALNT10**	+	230249	1.306	10857	5694	12578	0.453	-1.14324	0.439	0
chr2	207945528	208030614	**KLF7**	-	85086	8.305	25630	13445	29692	0.453	-1.143023	0.58	0

chr1	47897806	47900313	**MGC12982**	-	2507	1.546	132	69	153	0.453	-1.142665	0.448	0
chr14	24686056	24701576	**NEDD8**	-	15520	1.631	895	470	1036	0.453	-1.140978	0.309	0
chrY	7142012	7249588	**PRKY**	+	107576	1.087	4097	2151	4745	0.453	-1.141527	0.304	0
chr7	39663151	39747723	**RALA**	+	84572	2.526	7569	3971	8769	0.453	-1.142564	0.372	0
chr1	175913966	176176370	**RFWD2**	-	262404	1.382	12993	6816	15052	0.453	-1.142983	0.452	0
chrX	122734411	122866904	**THOC2**	-	132493	1.304	6190	3250	7170	0.453	-1.141391	0.368	0
chr1	165796731	165880855	**UCK2**	+	84124	2.1	6208	3260	7190	0.453	-1.14109	0.337	0
chr5	156904311	157002783	**ADAM19**	-	98472	2.757	9678	5089	11208	0.454	-1.139059	0.41	0
chr15	59397283	59417244	**CCNB2**	+	19961	1.339	941	495	1090	0.454	-1.138534	0.3	0
chr19	13945329	13947100	**LOC284454**	-	1771	38.603	2341	1230	2712	0.454	-1.140552	0.251	0
chr9	37915894	38069210	**SHB**	-	153316	2.874	15517	8163	17968	0.454	-1.138293	0.453	0
chr5	102594441	102614361	**C5orf30**	+	19920	15.307	11163	5875	12926	0.455	-1.137642	0.464	0
chr15	40763159	40765357	**CHST14**	+	2198	1.576	119	63	138	0.455	-1.135543	0.473	0
chr16	84509965	84538288	**KIAA1609**	-	28323	5.067	5038	2655	5832	0.455	-1.135089	0.322	0
chr16	55513080	55540586	**MMP2**	+	27506	5.079	4827	2543	5588	0.455	-1.135528	0.3	0
chr17	19808749	19881129	**AKAP10**	-	72380	1.217	3149	1661	3646	0.456	-1.13426	0.321	0
chr5	140782519	140785006	**PCDHGA9**	+	2487	1.634	141	74	163	0.456	-1.132764	0.463	0
chr20	34359922	34538288	**PHF20**	+	178366	1.184	7525	3974	8708	0.456	-1.131616	0.384	0
chr22	41641614	41682216	**RANGAP1**	-	40602	3.063	4349	2295	5034	0.456	-1.133144	0.311	0
chr20	48429249	48508772	**SLC9A8**	+	79523	1.323	3696	1951	4278	0.456	-1.132783	0.308	0
chr7	17830384	17980131	**SNX13**	-	149747	1.375	7309	3858	8460	0.456	-1.132548	0.376	0
chr6	27858092	27858570	**HIST1H3J**	-	478	3.503	58	30	67	0.457	-1.12861	0.57	0
chr1	179923907	180084015	**CEP350**	+	160108	1.318	7589	4022	8777	0.458	-1.125671	0.402	0
chr7	5659671	5821361	**RNF216**	-	161690	1.546	8896	4712	10290	0.458	-1.126649	0.405	0
chr10	16632616	16859453	**RSU1**	-	226837	1.591	12845	6805	14858	0.458	-1.126577	0.45	0
chr18	74069636	74207146	**ZNF516**	-	137510	2.15	10370	5496	11994	0.458	-1.125704	0.403	0
chr13	33078642	33083532	**CG030**	-	4890	4.004	693	368	801	0.459	-1.122544	0.338	0
chr5	80715671	81047072	**SSBP2**	-	331401	1.128	13628	7234	15759	0.459	-1.123277	0.496	0
chr1	119425665	119532179	**TBX15**	-	106514	1.514	5737	3044	6634	0.459	-1.123788	0.36	0
chr13	30338544	30424820	**UBL3**	-	86276	3.001	9324	4953	10781	0.459	-1.121978	0.429	0
chr6	100956607	101329224	**ASCC3**	-	372617	2.482	33302	17710	38500	0.46	-1.120305	0.639	0
chr6	117803819	117891020	**DCBLD1**	+	87201	3.098	9497	5049	10979	0.46	-1.120578	0.398	0
chr13	24995068	25086948	**PARP4**	-	91880	1.511	4892	2601	5656	0.46	-1.120361	0.334	0
chr2	218664511	218808796	**TNS1**	-	144285	1.901	9755	5185	11278	0.46	-1.120918	0.419	0
chr6	75794041	75915623	**COL12A1**	-	121582	8.704	38549	20556	44547	0.461	-1.115713	0.681	0
chr18	21572736	21715574	**TTC39C**	+	142838	1.889	9537	5082	11021	0.461	-1.116725	0.41	0
chr7	129470572	129592800	**UBE2H**	-	122228	2.134	9332	4970	10786	0.461	-1.117847	0.423	0
chr7	156931654	157062066	**UBE3C**	+	130412	1.744	8068	4296	9325	0.461	-1.118049	0.392	0
chr2	69685126	69870977	**AAK1**	-	185851	2.689	17878	9541	20657	0.462	-1.114293	0.512	0
chr15	101459459	101610317	**LRRK1**	+	150858	1.32	6984	3731	8068	0.462	-1.11269	0.367	0
chr18	56530060	56653709	**ZNF532**	+	123649	2.091	9298	4960	10744	0.462	-1.114918	0.432	0
chr1	17866329	18024370	**ARHGEF10L**	+	158041	1.077	5914	3163	6831	0.463	-1.110657	0.336	0
chr20	62152132	62153524	**PPDPF**	+	1392	9.358	445	238	514	0.463	-1.109375	0.338	0
chr7	32535175	32623779	**AVL9**	+	88604	2.225	6991	3748	8072	0.464	-1.106806	0.386	0
chr11	47487488	47574792	**CELF1**	-	87304	2.926	9024	4836	10420	0.464	-1.107339	0.409	0
chr7	2767740	2883959	**GNA12**	-	116219	1.619	6601	3538	7622	0.464	-1.107164	0.365	0
chr2	135809834	135928279	**RAB3GAP1**	+	118445	2.663	11333	6077	13086	0.464	-1.106562	0.457	0
chr2	235401685	235405693	**ARL4C**	-	4008	1.552	215	115	248	0.465	-1.106205	0.432	0
chr12	14518610	14651697	**ATF7IP**	+	133087	1.686	8087	4337	9337	0.465	-1.106176	0.424	0
chr12	122755980	122907116	**CLIP1**	-	151136	1.678	9052	4858	10451	0.465	-1.105109	0.424	0
chr13	51935700	52027275	**INTS6**	-	91575	1.602	5265	2824	6079	0.465	-1.10616	0.379	0
chr2	192110106	192290115	**MYO1B**	+	180009	5.458	35972	19292	41532	0.465	-1.106168	0.679	0
chr5	149887673	149937773	**NDST1**	+	50100	3.257	5726	3074	6611	0.465	-1.104771	0.351	0
chr11	68228185	68382801	**PPP6R3**	+	154616	2.462	13558	7275	15652	0.465	-1.10533	0.473	0
chr2	31456879	31491260	**EHD3**	+	34381	1.001	1184	637	1366	0.466	-1.101473	0.283	0
chr1	68167148	68299155	**GNG12**	-	132007	3.646	17097	9195	19731	0.466	-1.101539	0.505	0
chr10	103825123	103827795	**HPS6**	+	2672	2.585	236	126	272	0.466	-1.103022	0.407	0
chr2	182321618	182402468	**ITGA4**	+	80850	2.078	6128	3298	7071	0.466	-1.10036	0.42	0
chr17	26369687	26523404	**NLK**	+	153717	2.176	12128	6518	13998	0.466	-1.102678	0.486	0
chr4	85590692	85887544	**WDFY3**	-	296852	1.608	17194	9249	19843	0.466	-1.101217	0.523	0
chr6	26033319	26033796	**HIST1H2AB**	-	477	3.087	51	27	59	0.467	-1.098964	0.617	0
chr13	33160563	33352158	**PDS5B**	+	191595	1.003	6928	3731	7994	0.467	-1.099406	0.416	0
chr13	50018428	50069139	**SETDB2**	+	50711	1.092	1944	1048	2243	0.467	-1.097885	0.307	0
chr17	18218593	18231370	**SMCR8**	+	12777	2.45	1078	581	1244	0.467	-1.09886	0.289	0
chr2	69240275	69476459	**ANTXR1**	+	236184	2.117	17684	9553	20394	0.468	-1.094126	0.507	0
chr14	71374121	71582099	**PCNX**	+	207978	1.742	12999	7010	14995	0.468	-1.096832	0.483	0
chr4	39824482	39979576	**PDS5A**	-	155094	1.645	9097	4913	10491	0.468	-1.094416	0.429	0
chr11	6340175	6341740	**PRKCDBP**	-	1565	14.033	747	403	862	0.468	-1.095841	0.294	0
chr1	210001311	210030910	**DIEXF**	+	29599	3.322	3455	1870	3984	0.469	-1.091138	0.328	0
chr6	160390130	160527583	**IGF2R**	+	137453	2.022	9651	5217	11129	0.469	-1.092994	0.4	0
chr7	66018552	66043498	**LOC493754**	-	24946	2.13	1873	1012	2160	0.469	-1.093167	0.313	0

chr10	95066185	95242074	**MYOF**	-	175889	8.753	54636	29550	62999	0.469	-1.092139	0.736	0
chr7	65670258	65825438	**TPST1**	+	155180	1.087	5980	3233	6896	0.469	-1.092638	0.381	0
chr19	1911888	1913446	**ADAT3**	+	1558	1.867	99	53	114	0.47	-1.088938	0.499	0
chr19	47567446	47617009	**ZC3H4**	-	49563	1.529	2651	1436	3056	0.47	-1.089185	0.313	0
chr7	100026412	100031749	**MEPCE**	+	5337	3.324	609	331	702	0.471	-1.086173	0.329	0
chr15	52599479	52821247	**MYO5A**	-	221768	1.146	9072	4920	10457	0.471	-1.087542	0.437	0
chr9	107543283	107690527	**ABCA1**	-	147244	1.351	6775	3681	7807	0.472	-1.084444	0.336	0
chr19	13106583	13209610	**NFIX**	+	103027	2.06	7351	3996	8469	0.472	-1.083574	0.373	0
chr12	80167342	80329235	**PPP1R12A**	-	161893	4.581	27023	14693	31132	0.472	-1.083284	0.637	0
chr15	56382730	56535483	**RFX7**	-	152753	1.667	9133	4966	10523	0.472	-1.083231	0.449	0
chr16	85645028	85709812	**KIAA0182**	+	64784	1.144	2565	1398	2954	0.473	-1.078757	0.305	0
chr9	125703287	125867147	**RABGAP1**	+	163860	1.338	7809	4250	8995	0.473	-1.081439	0.42	0
chr15	50849351	50979012	**TRPM7**	-	129661	1.785	8216	4474	9463	0.473	-1.080751	0.421	0
chr12	7260903	7274447	**MATL2963**	+	13544	1.12	529	288	609	0.474	-1.076998	0.374	0
chr12	121200312	121342155	**SPPL3**	-	141843	1.145	5779	3151	6655	0.474	-1.078498	0.391	0
chr9	100961279	101018003	**TBC1D2**	-	56724	1.928	3795	2073	4369	0.474	-1.075879	0.324	0
chr21	44263203	44299678	**WDR4**	-	36475	1.472	1849	1009	2129	0.474	-1.076998	0.291	0
chr3	124944512	125094198	**ZNF148**	-	149686	2.122	11442	6247	13174	0.474	-1.076502	0.484	0
chr4	81187741	81212171	**FGF5**	+	24430	15.895	13650	7458	15714	0.475	-1.075105	0.473	0
chr17	66031847	66042970	**KPNA2**	+	11123	5.708	2245	1228	2584	0.475	-1.072715	0.333	0
chr6	52128811	52149582	**MCM3**	-	20771	1.402	1023	559	1178	0.475	-1.07391	0.331	0
chr9	123617930	123639606	**PHF19**	-	21676	1.462	1106	604	1273	0.475	-1.074142	0.318	0
chr9	33921690	34048947	**UBAP2**	-	127257	1.295	5836	3190	6718	0.475	-1.074074	0.388	0
chr4	114821439	114900878	**ARSJ**	-	79439	3.472	9942	5444	11441	0.476	-1.071421	0.471	0
chr2	189896640	190044605	**COL5A2**	-	147965	7.55	41009	22453	47195	0.476	-1.07174	0.725	0
chr17	17115526	17140502	**FLCN**	-	24976	2.72	2318	1269	2668	0.476	-1.071587	0.288	0
chr4	100869243	100871512	**H2AFZ**	-	2269	3.744	299	164	344	0.476	-1.07006	0.449	0
chr10	71029755	71161637	**HK1**	+	131882	1.541	7052	3860	8117	0.476	-1.072301	0.377	0
chr2	227596032	227663506	**IRS1**	-	67474	3.102	7437	4071	8559	0.476	-1.072001	0.419	0
chr9	21454266	21559697	**MIR31HG**	-	105431	2.327	8671	4753	9977	0.476	-1.069555	0.428	0
chr15	40226346	40327797	**EIF2AK4**	+	101451	1.715	6230	3421	7166	0.477	-1.06662	0.417	0
chr15	33010204	33026870	**GREM1**	+	16666	77.894	45749	25161	52612	0.478	-1.064181	0.703	0
chr19	2428163	2456958	**LMNB2**	-	28795	1.35	1350	743	1553	0.478	-1.063734	0.313	0
chr21	26934456	26947480	**MIR155HG**	+	13024	4.262	1967	1081	2262	0.478	-1.065203	0.339	0
chr16	70557690	70611571	**SF3B3**	+	53881	2.444	4652	2556	5351	0.478	-1.066087	0.374	0
chr1	52497776	52499472	**KTI12**	-	1696	4.396	256	141	294	0.479	-1.062528	0.432	0
chr16	71678851	71748704	**PHLPP2**	-	69853	1.145	2867	1582	3295	0.48	-1.058044	0.375	0
chr3	57743173	57914894	**SLMAP**	+	171721	1.015	6234	3440	7165	0.48	-1.058548	0.419	0
chr18	21111462	21166581	**NPC1**	-	55119	3.201	6131	3387	7046	0.481	-1.056709	0.376	0
chr4	184020462	184241929	**WWC2**	+	221467	2.373	18952	10476	21777	0.481	-1.055743	0.573	0
chr7	73150424	73153190	**ABHD11**	-	2766	1.508	142	78	163	0.482	-1.052197	0.485	0
chr11	65684282	65686531	**C11orf68**	-	2249	7.185	555	307	638	0.482	-1.054168	0.361	0
chr12	6603297	6641132	**NCAPD2**	+	37835	1.165	1569	868	1802	0.482	-1.053573	0.355	0
chr5	159848864	159855746	**PTTG1**	+	6882	2.385	585	324	672	0.482	-1.051652	0.416	0
chr2	161128661	161350318	**RBMS1**	-	221657	4.869	38932	21573	44718	0.482	-1.051641	0.705	0
chr14	77972339	78083110	**SPTLC2**	-	110771	3.064	12232	6778	14049	0.482	-1.051468	0.509	0
chr19	7745706	7747748	**TRAPPC5**	+	2042	1.753	122	68	141	0.482	-1.053249	0.506	0
chr19	1103935	1106787	**GPX4**	+	2852	2.32	227	126	261	0.483	-1.049907	0.45	0
chr15	63900816	64126147	**HERC1**	-	225331	1.322	10694	5928	12282	0.483	-1.050796	0.489	0
chr14	24836144	24848810	**NFATC4**	+	12666	2.205	956	530	1098	0.483	-1.048923	0.314	0
chr10	105353783	105615164	**SH3PXD2A**	-	261381	1.777	16160	8965	18559	0.483	-1.049759	0.5	0
chr11	313990	315272	**IFITM1**	+	1282	1.357	60	33	69	0.484	-1.04696	0.606	0
chr2	39476421	39664219	**MAP4K3**	-	187798	2.848	19147	10643	21981	0.484	-1.046369	0.57	0
chr6	20402136	20493945	**E2F3**	+	91809	2.78	8981	4995	10310	0.485	-1.0453	0.443	0
chr10	86088409	86278276	**FAM190B**	+	189867	1.901	12779	7109	14669	0.485	-1.045122	0.494	0
chr8	42128819	42190171	**IKBKB**	+	61352	1.326	2850	1587	3271	0.485	-1.043254	0.345	0
chr13	111293756	111358480	**CARS2**	-	64724	2.716	6064	3382	6958	0.486	-1.040827	0.376	0
chr12	70636773	70748773	**CNOT2**	+	112000	2.584	10333	5761	11857	0.486	-1.04136	0.481	0
chr3	130569368	130735555	**ATP2C1**	+	166187	2.345	13995	7815	16055	0.487	-1.038634	0.53	0
chr10	92980368	93044021	**PCGF5**	+	63653	1.302	2981	1664	3420	0.487	-1.039084	0.395	0
chr20	34213952	34236846	**CPNE1**	-	22894	2.594	2079	1163	2384	0.488	-1.034971	0.341	0
chr5	92745064	92917003	**FLJ42709**	-	171939	1.393	8640	4834	9909	0.488	-1.035345	0.481	0
chr1	89149921	89301938	**PKN2**	+	152017	1.658	8997	5036	10317	0.488	-1.034509	0.47	0
chr6	35310334	35395968	**PPARD**	+	85634	1.775	5315	2974	6095	0.488	-1.035033	0.387	0
chr8	9413444	9639856	**TNKS**	+	226412	1.466	11824	6618	13559	0.488	-1.034801	0.5	0
chr21	38739858	38887679	**DYRK1A**	+	147821	5.383	28376	15901	32535	0.489	-1.03284	0.645	0
chr14	63841354	64010079	**PPP2R5E**	-	168725	1.35	8136	4559	9328	0.489	-1.032818	0.458	0
chr11	64085559	64089295	**PRDX5**	+	3736	1.872	240	134	275	0.489	-1.032723	0.453	0
chr6	163835674	163999628	**QKI**	+	163954	2.849	16774	9409	19229	0.489	-1.031199	0.568	0
chr14	96722546	96731100	**BDKRB1**	+	8554	3.109	942	529	1080	0.49	-1.028752	0.382	0
chr8	25276773	25282556	**GNRH1**	-	5783	1.345	276	155	317	0.49	-1.029594	0.496	0

chr13	45694630	45858239	**GTF2F2**	+	163609	1.18	6891	3867	7899	0.49	-1.030467	0.448	0
chr6	52362199	52441862	**TRAM2**	-	79663	7.88	22131	12433	25363	0.49	-1.028564	0.583	0
chr14	103398715	103523742	**CDC42BPB**	-	125027	2.086	9101	5120	10428	0.491	-1.026198	0.444	0
chr16	27325250	27376099	**IL4R**	+	50849	1.385	2437	1371	2792	0.491	-1.02561	0.334	0
chr1	112162404	112256101	**RAP1A**	+	93697	2.594	8654	4874	9914	0.492	-1.024444	0.469	0
chr2	128458596	128461407	**SFT2D3**	+	2811	1.402	137	77	157	0.492	-1.022541	0.538	0
chr1	46085715	46089731	**CCDC17**	-	4016	1.141	157	88	180	0.493	-1.02021	0.503	0
chr22	19318223	19419219	**HIRA**	-	100996	1.129	3978	2247	4555	0.493	-1.019408	0.375	0
chr6	26225382	26225844	**HIST1H3E**	+	462	4.452	69	39	79	0.493	-1.019563	0.575	0
chr11	65837823	66012218	**PACS1**	+	174395	1.179	7308	4126	8369	0.493	-1.020179	0.451	0
chr9	35490006	35561895	**RUSC2**	+	71889	4.603	11869	6697	13593	0.493	-1.021266	0.516	0
chr4	159690181	159827954	**FNIP2**	+	137773	2.092	10088	5703	11549	0.494	-1.017806	0.463	0
chr9	108210314	108311385	**FSD1L**	+	101071	1.177	4213	2384	4822	0.494	-1.016002	0.405	0
chr6	143929316	144152322	**PHACTR2**	+	223006	1.7	13578	7680	15544	0.494	-1.017082	0.538	0
chr11	128328655	128457453	**ETS1**	-	128798	4.184	18833	10676	21552	0.495	-1.013394	0.555	0
chr2	75719443	75796848	**FAM176A**	-	77405	1.734	4786	2713	5477	0.495	-1.013316	0.424	0
chr14	73741917	73925286	**NUMB**	-	183369	1.347	8775	4974	10042	0.495	-1.013459	0.473	0
chr2	128698790	128784869	**SAP130**	-	86079	1.026	3097	1756	3544	0.495	-1.013196	0.376	0
chrX	53559062	53713673	**HUWE1**	-	154611	2.053	11238	6380	12857	0.496	-1.010937	0.499	0
chr5	36876860	37065921	**NIPBL**	+	189061	1.942	13234	7514	15140	0.496	-1.010651	0.551	0
chr6	34204576	34214008	**HMGA1**	+	9432	2.144	695	395	795	0.497	-1.007266	0.372	0
chr13	23902964	24007841	**SACS**	-	104877	3.064	11479	6531	13129	0.497	-1.00727	0.514	0
chr2	175424301	175547627	**WIPF1**	-	123326	1.749	7724	4388	8835	0.497	-1.009481	0.474	0
chr17	79523912	79604138	**NPLOC4**	-	80226	3.498	9782	5573	11185	0.498	-1.004976	0.46	0
chr1	109822175	109825790	**PSRC1**	-	3615	1.739	221	126	253	0.498	-1.004631	0.512	0
chr14	69340839	69446083	**ACTN1**	-	105244	12.546	46065	26256	52668	0.499	-1.004262	0.723	0
chr8	143781528	143785584	**LY6K**	+	4056	1.785	247	141	282	0.499	-1.002114	0.461	0
chr5	109025155	109203429	**MAN2A1**	+	178274	1.241	7842	4474	8964	0.499	-1.002622	0.464	0
chr20	49575362	49577820	**MOCS3**	+	2458	2.255	189	108	216	0.499	-1.00365	0.493	0
chr10	104847773	104953063	**NT5C2**	-	105290	2.805	10630	6062	12153	0.499	-1.003288	0.52	0
chr5	102465256	102538909	**PPIP5K2**	+	73653	1.311	3444	1964	3938	0.499	-1.003153	0.414	0
chr16	30565084	30569642	**ZNF764**	-	4558	1.344	209	119	239	0.499	-1.001561	0.481	0
chr11	35160416	35253949	**CD44**	+	93533	24.339	80535	46039	92033	0.5	-0.999282	0.863	0
chr1	145611035	145688776	**RNF115**	+	77741	2.254	6250	3572	7143	0.5	-0.99949	0.453	0
chr2	230631929	230786655	**TRIP12**	-	154726	3.103	17048	9746	19482	0.5	-0.999268	0.57	0
chr3	149235021	149421060	**WWTR1**	-	186039	1.483	9616	5496	10989	0.5	-0.999455	0.463	0
**Total**					**1.2E+08**								
**Average size**					**139848**								
