# Supplementary material for: Genome-Wide Transcriptional Effects of the Anti-Cancer Agent Camptothecin
Source: PLoS One. 2013 Oct 23;8(10):e78190. doi: 10.1371/journal.pone.0078190 (PMC3806802; doi:10.1371/journal.pone.0078190)
Supplement: Table S4 — Up and down regulation of genes following CPT treatment and 30 min recovery. (PDF) [file pone.0078190.s005.pdf]

Table S4. Up and down regulation of genes following CPT treatment and 30 min recovery.

Pages 1-5: Table S4A - UP-regulated genes
Pages 6-17: Table S4B - DOWN-regulated genes

Table S4A. Genes UP-regulated >2-fold following 45 min CPT treatment, wash and 30 min recovery with Bru last 15 min (CPT+30m).

chrom	start	end	name	strand	bp	meanRPKM	meanCount	1_CPT30m	2_cont	foldChange	log2FoldCha	pAdjusted	significant
chr1	89517986	89531043	**GBP1**	-	13057	1.289	536	1472	224	6.564	2.715	0	1
chrX	53111541	53117728	**TSPYL2**	+	6187	2.901	570	1521	253	5.992	2.583	0	1
chr10	112257624	112271302	**DUSP5**	+	13678	10.455	4520	11266	2272	4.959	2.31	0.001	1
chr14	96846021	96853563	**C14orf129**	+	7542	1.331	323	781	171	4.566	2.191	0.004	1
chr17	72858618	72869156	**FDXR**	-	10538	1.582	532	1284	281	4.555	2.188	0.003	1
chrX	135228860	135293518	**FHL1**	+	64658	2.425	5118	12238	2745	4.458	2.156	0.002	1
chr1	8071778	8086393	**ERRFI1**	-	14615	2.434	1140	2704	619	4.365	2.126	0.001	1
chr7	141463896	141464997	**TAS2R3**	+	1101	1.071	37	88	20	4.27	2.094	0.076	1
chr20	10618331	10654694	**JAG1**	-	36363	1.686	1971	4560	1107	4.117	2.042	0.002	1
chr5	72848024	72861511	**ANKRA2**	-	13487	1.226	539	1236	306	4.034	2.012	0.005	1
chr10	30722949	30750762	**MAP3K8**	+	27813	1.099	982	2212	572	3.862	1.949	0.005	1
chr8	95938199	95961615	**TP53INP1**	-	23416	4.695	3531	7922	2067	3.832	1.938	0.007	1
chr13	107142078	107187388	**EFNB2**	-	45310	2.645	3841	8584	2259	3.799	1.926	0.008	1
chr6	72124148	72130448	**LINC00472**	-	6300	4.055	825	1831	489	3.739	1.903	0.007	1
chr6	121756744	121770873	**GJA1**	+	14129	1.882	865	1916	514	3.721	1.896	0.006	1
chr12	4430358	4469190	**C12orf5**	+	38832	2.5	3135	6942	1866	3.719	1.895	0.007	1
chr10	90694830	90751147	**ACTA2**	-	56317	2.212	4107	9089	2446	3.716	1.894	0.006	1
chr18	57567191	57571538	**PMAIP1**	+	4347	3.959	549	1214	327	3.712	1.892	0.014	1
chr10	60094738	60130513	**UBE2D1**	+	35775	1.324	1534	3384	918	3.685	1.882	0.006	1
chr11	10772810	10801290	**CTR9**	+	28480	1.92	1781	3928	1065	3.685	1.882	0.005	1
chr6	16129316	16148478	**MYLIP**	+	19162	1.35	824	1803	498	3.617	1.855	0.012	1
chr12	77252495	77272799	**CSRP2**	-	20304	2.278	1505	3211	937	3.425	1.776	0.01	1
chr4	17578926	17609590	**LAP3**	+	30664	1.302	1281	2733	798	3.425	1.776	0.013	1
chr9	22002901	22009312	**CDKN2B**	-	6411	1.708	353	749	221	3.384	1.759	0.033	1
chr4	89181531	89205888	**PPM1K**	-	24357	1.118	886	1873	557	3.36	1.749	0.014	1
chr1	113454469	113498975	**SLC16A1**	-	44506	1.611	2321	4883	1467	3.328	1.734	0.014	1
chrX	37698088	37706889	**DYNLT3**	-	8801	1.112	321	674	203	3.317	1.73	0.033	1
chr15	44092618	44094769	**C15orf63**	+	2151	2.393	167	349	106	3.275	1.711	0.067	1
chr17	33738080	33759543	**SLFN12**	-	21463	1.512	1060	2210	676	3.266	1.708	0.014	1
chr5	53751430	53752214	**HSPB3**	+	784	2.772	69	145	44	3.241	1.697	0.136	0
chr6	71998476	72011973	**OGFRL1**	+	13497	3.131	1384	2871	888	3.234	1.693	0.014	1
chr5	43289492	43313595	**HMGCS1**	-	24103	3.123	2424	4992	1568	3.183	1.67	0.022	1
chr8	134249413	134309547	**NDRG1**	-	60134	1.1	2129	4384	1378	3.18	1.669	0.022	1
chr1	163038395	163046592	**RGS4**	+	8197	1.344	347	715	224	3.18	1.669	0.065	1
chr13	77566058	77576652	**CLN5**	+	10594	1.351	465	957	301	3.178	1.668	0.035	1
chr1	85623355	85666728	**SYDE2**	-	43373	1.086	1547	3183	1002	3.176	1.667	0.015	1
chr10	90750287	90775542	**FAS**	+	25255	3.041	2502	5141	1622	3.169	1.664	0.019	1
chr14	80663867	80697397	**DIO2**	-	33530	1.248	1356	2785	880	3.163	1.661	0.02	1
chr13	49063098	49107316	**RCBTB2**	-	44218	1.042	1499	3072	975	3.15	1.656	0.019	1
chrX	24001832	24045303	**KLHL15**	-	43471	1.387	1961	4012	1278	3.14	1.651	0.02	1
chr9	91605777	91611057	**C9orf47**	+	5280	1.939	332	679	216	3.135	1.649	0.052	1
chr1	192778168	192781407	**RGS2**	+	3239	2.547	266	543	173	3.125	1.644	0.066	1
chr14	45366506	45376460	**C14orf28**	+	9954	1.59	513	1046	335	3.121	1.642	0.038	1
chr8	40010986	40012827	**C8orf4**	+	1841	9.146	551	1124	360	3.121	1.642	0.031	1
chr11	121163387	121184119	**SC5DL**	+	20732	1.492	1007	2053	658	3.12	1.642	0.022	1
chr6	52529198	52533951	**LOC730101**	+	4753	2.909	451	918	295	3.109	1.637	0.04	1
chr21	19161283	19191703	**C21orf91**	-	30420	1.306	1287	2616	844	3.097	1.631	0.024	1
chr19	47724078	47736023	**BBC3**	-	11945	2.948	1127	2290	740	3.095	1.63	0.03	1
chr2	208576263	208620896	**CCNYL1**	+	44633	1.352	1949	3947	1283	3.076	1.621	0.026	1
chr2	3501689	3523350	**ADI1**	-	21661	2.754	1940	3925	1278	3.069	1.618	0.024	1
chr1	185265521	185286461	**IVNS1ABP**	-	20940	5.424	3713	7492	2453	3.054	1.61	0.029	1
chr1	85109389	85156240	**SSX2IP**	-	46851	2.771	4264	8517	2846	2.992	1.581	0.034	1
chr4	75230859	75254477	**EREG**	+	23618	10.069	7588	15007	5115	2.934	1.553	0.082	1
chr2	36581891	36582713	**LOC100288911**	-	822	2.094	55	109	37	2.926	1.549	0.202	0
chr1	110009099	110024764	**SYPL2**	+	15665	1.257	636	1256	430	2.921	1.546	0.052	1
chr7	30052883	30066268	**FKBP14**	-	13385	1.031	452	891	306	2.907	1.539	0.057	1
chr19	2037469	2051243	**MKNK2**	-	13774	1.331	587	1154	397	2.901	1.537	0.064	1
chr19	18496967	18499986	**GDF15**	+	3019	8.128	779	1527	529	2.884	1.528	0.063	1
chr12	8071823	8088892	**SLC2A3**	-	17069	2.497	1366	2674	930	2.876	1.524	0.047	1
chr18	3262110	3278282	**MYL12B**	+	16172	8.181	4368	8537	2978	2.866	1.519	0.042	1

chr1	179851419	179889211	**TOR1AIP1**	+	37792	2.308	2845	5558	1941	2.863	1.517	0.041	1
chr5	139929652	139937678	**SRA1**	-	8026	3.604	948	1841	650	2.832	1.502	0.041	1
chr10	69644426	69678147	**SIRT1**	+	33721	2.132	2358	4559	1624	2.806	1.489	0.041	1
chr8	57124314	57131176	**CHCHD7**	+	6862	2.178	487	941	335	2.802	1.486	0.071	1
chr10	112631552	112659764	**PDCD4**	+	28212	1.554	1443	2787	996	2.798	1.485	0.039	1
chr11	5710816	5732093	**TRIM22**	+	21277	3.444	2411	4648	1665	2.791	1.481	0.041	1
chr10	1085963	1095061	**IDI1**	-	9098	2.983	879	1689	608	2.775	1.473	0.06	1
chr2	70142172	70170076	**MXD1**	+	27904	1.16	1048	2011	726	2.767	1.469	0.057	1
chr4	140374960	140397069	**RAB33B**	+	22109	1.193	857	1644	595	2.763	1.466	0.059	1
chr6	160100148	160114353	**SOD2**	-	14205	2.8	1281	2451	891	2.751	1.46	0.061	1
chr1	33402049	33430286	**RNF19B**	-	28237	1.321	1211	2313	844	2.741	1.455	0.056	1
chr1	203274663	203278729	**BTG2**	+	4066	5.608	728	1390	507	2.738	1.453	0.082	1
chr17	58677543	58743640	**PPM1D**	+	66097	2.452	5321	10133	3717	2.726	1.447	0.071	1
chr8	81398447	81434610	**ZBTB10**	+	36163	1.009	1200	2285	838	2.725	1.446	0.048	1
chr13	114567149	114569805	**LOC100506394**	+	2656	1.399	121	230	85	2.714	1.44	0.17	0
chr3	195241220	195270224	**PPP1R2**	-	29004	1.952	1862	3536	1303	2.712	1.44	0.05	1
chr17	43224683	43229468	**HEXIM1**	+	4785	3.454	535	1016	375	2.707	1.437	0.087	1
chr8	59465727	59495419	**SDCBP**	+	29692	4.305	4138	7845	2902	2.703	1.434	0.077	1
chr7	106809405	106842974	**HBP1**	+	33569	2.641	2911	5495	2050	2.68	1.422	0.061	1
chr8	104426941	104455680	**DCAF13**	+	28739	1.562	1478	2788	1042	2.674	1.419	0.054	1
chr7	25158269	25164980	**CYCS**	-	6711	3.571	783	1475	552	2.671	1.417	0.07	1
chr1	89318320	89357301	**GTF2B**	-	38981	2.394	3054	5751	2155	2.669	1.416	0.065	1
chr21	28208605	28217728	**ADAMTS1**	-	9123	75.664	22172	41682	15669	2.66	1.411	0.228	0
chr5	14581890	14616287	**FAM105A**	+	34397	1.492	1671	3136	1183	2.65	1.406	0.065	1
chr8	22993103	23021540	**TNFRSF10D**	-	28437	5.504	5092	9553	3606	2.649	1.406	0.09	1
chr17	33570085	33594761	**SLFN5**	+	24676	6.494	5253	9846	3722	2.645	1.403	0.086	1
chr10	102033712	102046439	**BLOC1S2**	-	12727	2.52	1055	1976	748	2.642	1.402	0.062	1
chr5	112196884	112228776	**SRP19**	+	31892	1.822	1895	3545	1345	2.635	1.398	0.066	1
chr10	64893006	64914786	**NRBF2**	+	21780	1.783	1277	2385	908	2.626	1.393	0.061	1
chr6	138409641	138428660	**PERP**	-	19019	1.495	928	1730	660	2.621	1.39	0.073	1
chr12	91497231	91505542	**LUM**	-	8311	4.472	1212	2258	864	2.612	1.385	0.07	1
chr12	75874512	75895716	**GLIPR1**	+	21204	2.716	1895	3521	1353	2.602	1.379	0.065	1
chr13	31710762	31736117	**HSPH1**	-	25355	2.318	1908	3535	1366	2.587	1.372	0.078	1
chr15	66782665	66790146	**SNAPC5**	-	7481	1.508	369	684	264	2.585	1.37	0.123	0
chr7	17338275	17385775	**AHR**	+	47500	3.288	5057	9364	3622	2.585	1.37	0.107	0
chr17	73937588	73975515	**ACOX1**	-	37927	1.858	2312	4280	1656	2.584	1.37	0.071	1
chr5	92919042	92929786	**NR2F1**	+	10744	1.7	592	1095	424	2.579	1.367	0.106	0
chr9	33025208	33039062	**DNAJA1**	+	13854	4.593	2086	3856	1496	2.577	1.366	0.071	1
chr7	112063198	112117258	**IFRD1**	+	54060	3.194	5670	10480	4067	2.577	1.366	0.101	0
chr1	85731459	85742587	**BCL10**	-	11128	4.537	1655	3057	1188	2.573	1.364	0.071	1
chr12	69201970	69239212	**MDM2**	+	37242	12.127	14811	27315	10643	2.566	1.36	0.178	0
chr11	65265232	65273939	**MALAT1**	+	8707	625.155	179865	331673	129262	2.566	1.359	0.506	0
chr1	231154703	231175995	**FAM89A**	-	21292	1.008	697	1281	503	2.546	1.348	0.102	0
chr13	28194879	28241559	**POLR1D**	+	46680	1.704	2600	4767	1878	2.538	1.344	0.086	1
chr5	55230924	55290821	**IL6ST**	-	59897	3.579	7039	12902	5085	2.537	1.343	0.121	0
chr5	53813588	53842416	**SNX18**	+	28828	4.081	3877	7106	2801	2.537	1.343	0.089	1
chr3	141595469	141645382	**ATP1B3**	+	49913	3.002	4876	8932	3525	2.534	1.341	0.111	0
chr13	48807273	48836232	**ITM2B**	+	28959	3.107	2966	5412	2150	2.517	1.332	0.085	1
chr1	47073386	47082563	**MOB3C**	-	9177	1.638	489	891	354	2.513	1.329	0.129	0
chr2	86830515	86851000	**RNF103**	-	20485	1.874	1257	2289	914	2.504	1.324	0.087	1
chr3	187439164	187463513	**BCL6**	-	24349	4.282	3385	6157	2461	2.501	1.323	0.107	0
chrX	70521627	70525204	**ITGB1BP2**	+	3577	1.06	124	227	90	2.498	1.321	0.21	0
chr22	38597938	38612517	**MAFF**	+	14579	3.884	1826	3316	1329	2.494	1.319	0.104	0
chr6	116832807	116839709	**FAM26E**	+	6902	1.242	284	516	207	2.49	1.316	0.15	0
chr17	7387697	7417935	**POLR2A**	+	30238	7.092	6973	12651	5080	2.49	1.316	0.147	0
chr1	110276553	110283660	**GSTM3**	-	7107	1.41	331	599	241	2.48	1.31	0.148	0
chr12	14927269	14930936	**H2AFJ**	+	3667	3.336	398	718	291	2.466	1.302	0.154	0
chr3	28363844	28390618	**AZI2**	-	26774	2.111	1861	3356	1363	2.461	1.299	0.09	1
chr18	3247527	3256234	**MYL12A**	+	8707	13.3	3821	6882	2800	2.458	1.297	0.103	0
chr5	37812778	37839782	**GDNF**	-	27004	2.605	2279	4102	1671	2.454	1.295	0.111	0
chr8	126010719	126034525	**SQLE**	+	23806	1.822	1406	2529	1031	2.452	1.294	0.108	0
chr11	104813593	104839325	**CASP4**	-	25732	1.613	1348	2416	991	2.437	1.285	0.11	0
chr1	28099693	28150963	**STX12**	+	51270	2.436	4114	7371	3028	2.434	1.284	0.114	0
chr2	62423261	62451866	**B3GNT2**	+	28605	1.491	1383	2477	1018	2.433	1.283	0.112	0
chr17	10600926	10614875	**C17orf48**	+	13949	2.819	1313	2351	967	2.429	1.281	0.083	1
chr14	39865576	39901704	**FBXO33**	-	36128	1.582	1881	3366	1386	2.428	1.28	0.099	1
chr7	134127106	134143888	**AKR1B1**	-	16782	1.703	923	1651	680	2.428	1.28	0.127	0
chr6	7390061	7418270	**RIOK1**	+	28209	1.051	972	1737	717	2.422	1.276	0.108	0
chr4	110736665	110745893	**GAR1**	+	9228	1.53	467	834	344	2.422	1.276	0.138	0
chr11	2661767	2721228	**KCNQ1OT1**	-	59461	1.606	3115	5556	2301	2.414	1.271	0.12	0
chr4	120217573	120225600	**C4orf3**	-	8027	1.652	438	781	324	2.41	1.269	0.146	0

chr14	61176255	61190852	**SIX4**	-	14597	1.651	783	1395	579	2.41	1.269	0.134	0
chr18	47007547	47013644	**C18orf32**	-	6097	1.653	331	590	245	2.409	1.268	0.169	0
chr4	108852716	108874613	**CYP2U1**	+	21897	1.157	830	1479	614	2.408	1.268	0.119	0
chr4	148538538	148556672	**TMEM184C**	+	18134	1.52	910	1619	674	2.403	1.265	0.108	0
chr8	103216728	103251346	**RRM2B**	-	34618	2.054	2331	4145	1726	2.401	1.263	0.111	0
chr6	26634610	26659980	**ZNF322**	-	25370	1.588	1321	2348	979	2.398	1.262	0.108	0
chr2	68385004	68403094	**PNO1**	+	18090	1.44	854	1518	633	2.398	1.262	0.119	0
chr1	68150859	68154021	**GADD45A**	+	3162	15.847	1630	2889	1210	2.387	1.255	0.12	0
chr7	91741462	91764059	**CYP51A1**	-	22597	2.027	1481	2624	1100	2.384	1.253	0.129	0
chr7	92167788	92169079	**MGC16142**	+	1291	1.166	49	88	37	2.383	1.253	0.316	0
chr5	162864576	162872022	**CCNG1**	+	7446	8.808	2159	3820	1605	2.379	1.25	0.111	0
chr13	73329539	73356344	**DIS3**	-	26805	3.444	3038	5371	2261	2.376	1.248	0.119	0
chr21	35467161	35478561	**SLC5A3**	+	11400	2.77	1029	1816	766	2.37	1.245	0.128	0
chr1	111489811	111506566	**LRIF1**	-	16755	2.056	1131	1996	843	2.367	1.243	0.117	0
chr14	51100359	51135023	**SAV1**	-	34664	2.118	2433	4292	1814	2.366	1.243	0.108	0
chr6	153308399	153323925	**MTRF1L**	-	15526	1.91	976	1721	728	2.364	1.241	0.119	0
chr3	160939098	160969795	**NMD3**	+	30697	2.878	2914	5130	2176	2.358	1.238	0.12	0
chr2	54014067	54045956	**ERLEC1**	+	31889	1.871	1963	3455	1466	2.356	1.237	0.116	0
chr1	231664398	231702269	**TSNAX**	+	37871	1.306	1632	2863	1221	2.344	1.229	0.115	0
chr7	10971579	10979813	**NDUFA4**	-	8234	2.893	790	1386	591	2.342	1.228	0.123	0
chr11	117070039	117075508	**TAGLN**	+	5469	14.25	2534	4441	1898	2.339	1.226	0.143	0
chr9	4709556	4742043	**AK3**	-	32487	1.339	1431	2508	1072	2.338	1.225	0.12	0
chr1	110527386	110566364	**AHCYL1**	+	38978	3.06	3936	6894	2950	2.336	1.224	0.138	0
chr13	76099349	76111991	**COMMD6**	-	12642	1.474	613	1074	460	2.334	1.223	0.15	0
chr2	26568953	26618759	**EPT1**	+	49806	1.306	2145	3753	1609	2.332	1.222	0.12	0
chr16	84599203	84651669	**COTL1**	-	52466	3.263	5594	9786	4196	2.332	1.222	0.168	0
chr5	74632992	74657926	**HMGCR**	+	24934	2.948	2395	4187	1797	2.329	1.22	0.142	0
chr1	205055269	205091150	**RBBP5**	-	35881	1.553	1825	3188	1371	2.325	1.217	0.13	0
chr12	107349543	107367813	**C12orf23**	+	18270	9.406	5694	9937	4280	2.321	1.215	0.153	0
chr4	76567952	76598667	**G3BP2**	-	30715	3.368	3403	5933	2560	2.318	1.213	0.143	0
chr1	165631448	165667900	**ALDH9A1**	-	36452	1.643	1954	3406	1470	2.316	1.211	0.14	0
chr9	4679565	4706594	**CDC37L1**	+	27029	1.493	1330	2312	1003	2.305	1.205	0.128	0
chr7	30174551	30202381	**C7orf41**	+	27830	1.117	1021	1774	770	2.302	1.203	0.14	0
chr11	105878628	105892954	**KIAA1826**	-	14326	1.445	679	1180	512	2.301	1.202	0.156	0
chr5	96496570	96519005	**RIOK2**	-	22435	1.845	1374	2383	1037	2.298	1.2	0.121	0
chr2	64319785	64371605	**PELI1**	-	51820	1.089	1870	3242	1412	2.295	1.198	0.124	0
chr7	43622691	43666978	**STK17A**	+	44287	6.022	8764	15192	6622	2.294	1.198	0.207	0
chr1	94352589	94375012	**GCLM**	-	22423	2.014	1498	2594	1132	2.291	1.196	0.123	0
chr9	35732316	35737005	**CREB3**	+	4689	2.093	320	554	242	2.29	1.196	0.218	0
chr17	67240575	67323323	**ABCA5**	-	82748	1.021	2784	4817	2107	2.286	1.193	0.143	0
chr4	139936942	139967095	**CCRN4L**	+	30153	3.226	3185	5509	2411	2.285	1.192	0.153	0
chr6	36644236	36655116	**CDKN1A**	+	10880	39.123	13636	23581	10321	2.285	1.192	0.293	0
chr4	113152894	113191211	**AP1AR**	+	38317	1.156	1433	2475	1086	2.278	1.187	0.16	0
chr6	44225902	44233525	**NFKBIE**	-	7623	1.048	257	445	195	2.277	1.187	0.261	0
chr7	127010353	127032767	**ZNF800**	-	22414	2.71	1997	3444	1514	2.274	1.185	0.144	0
chr20	7961715	8000393	**TMX4**	-	38678	1.401	1776	3062	1347	2.272	1.184	0.148	0
chr11	10533224	10562774	**RNF141**	-	29550	1.608	1571	2706	1192	2.27	1.183	0.135	0
chr10	115881973	115934364	**C10orf118**	-	52391	1.076	1867	3204	1421	2.255	1.173	0.139	0
chr8	110346551	110358189	**ENY2**	+	11638	2.853	1091	1870	831	2.25	1.17	0.153	0
chr20	33292147	33301237	**TP53INP2**	+	9090	3.669	1076	1845	820	2.249	1.17	0.178	0
chr12	49782956	49786116	**LOC100335030**	+	3160	1.237	127	218	97	2.246	1.167	0.308	0
chr4	103998781	104021024	**BDH2**	-	22243	1.156	845	1446	644	2.243	1.165	0.163	0
chr18	21032786	21063099	**RIOK3**	+	30313	2.618	2621	4481	2001	2.239	1.163	0.151	0
chr1	45976706	45988562	**PRDX1**	-	11856	5.782	2262	3867	1727	2.239	1.163	0.15	0
chr6	146864827	146876086	**RAB32**	+	11259	3.61	1334	2280	1019	2.237	1.162	0.153	0
chr10	18948312	18966940	**ARL5B**	+	18628	2.61	1600	2733	1222	2.236	1.161	0.151	0
chr2	165754708	165812035	**SLC38A11**	-	57327	2.276	4308	7357	3291	2.235	1.16	0.17	0
chr8	118532964	118552501	**MED30**	+	19537	1.239	796	1359	608	2.234	1.159	0.168	0
chr2	86668270	86719839	**KDM3A**	+	51569	3.192	5437	9281	4155	2.233	1.159	0.184	0
chr1	116915794	116947396	**ATP1A1**	+	31602	3.362	3491	5954	2670	2.229	1.157	0.17	0
chr5	131817300	131826465	**IRF1**	-	9165	1.959	582	992	445	2.227	1.155	0.208	0
chr5	137673223	137685418	**FAM53C**	+	12195	3.26	1291	2197	988	2.223	1.152	0.175	0
chr7	105888731	105925638	**NAMPT**	-	36907	5.054	6127	10426	4694	2.221	1.151	0.205	0
chr5	68389775	68426899	**SLC30A5**	+	37124	1.511	1850	3147	1418	2.218	1.15	0.153	0
chr2	209100952	209119806	**IDH1**	-	18854	1.115	684	1162	524	2.217	1.149	0.2	0
chr2	198318230	198339851	**COQ10B**	+	21621	3.986	2829	4809	2169	2.217	1.149	0.169	0
chr5	33987090	34008220	**AMACR**	-	21130	1.653	1154	1961	885	2.215	1.147	0.154	0
chr14	50234786	50249856	**KLHDC2**	+	15070	1.548	776	1317	595	2.212	1.146	0.161	0
chr7	134331530	134364567	**BPGM**	+	33037	2.402	2620	4445	2012	2.209	1.143	0.161	0
chr5	158690088	158713048	**UBLCP1**	+	22960	1.712	1303	2210	1000	2.209	1.143	0.151	0
chr12	6571403	6579843	**VAMP1**	-	8440	2.44	673	1141	517	2.205	1.141	0.198	0

chr5	34929697	34959069	**DNAJC21**	+	29372	1.989	1928	3264	1482	2.202	1.139	0.159	0
chr4	129190391	129209984	**PGRMC2**	-	19593	4.556	2990	5061	2299	2.201	1.138	0.15	0
chr12	105380097	105388505	**C12orf45**	+	8408	1.226	338	572	260	2.2	1.138	0.24	0
chr4	15683351	15692070	**FAM200B**	+	8719	3.332	967	1637	744	2.2	1.137	0.153	0
chr1	145507556	145513535	**RBM8A**	+	5979	1.541	302	511	232	2.198	1.136	0.25	0
chr12	100594573	100618201	**ACTR6**	+	23628	1.121	875	1480	673	2.197	1.135	0.17	0
chr10	72163860	72188374	**EIF4EBP2**	+	24514	3.223	2594	4385	1997	2.196	1.135	0.174	0
chr20	4666796	4682234	**PRNP**	+	15438	5.68	2868	4846	2209	2.194	1.133	0.183	0
chr15	45003684	45010357	**B2M**	+	6673	13.73	3021	5102	2327	2.193	1.133	0.173	0
chr8	38846326	38854041	**TM2D2**	-	7715	1.931	490	828	377	2.191	1.132	0.208	0
chr2	24290453	24299314	**SF3B14**	-	8861	1.498	439	742	339	2.19	1.131	0.208	0
chr1	39491966	39500308	**NDUFS5**	+	8342	2.006	551	929	425	2.186	1.128	0.207	0
chr14	54976586	55005334	**CGRRF1**	+	28748	1.028	967	1629	746	2.184	1.127	0.185	0
chr16	19513014	19533450	**GDE1**	-	20436	1.24	832	1401	642	2.181	1.125	0.189	0
chr10	71962585	71993190	**PPA1**	-	30605	1.017	1019	1717	787	2.181	1.125	0.182	0
chr15	45694518	45713616	**SPATA5L1**	+	19098	1.116	699	1177	540	2.18	1.124	0.2	0
chr11	85955805	85989785	**EED**	+	33980	2.302	2585	4351	1996	2.179	1.124	0.17	0
chr9	107509968	107522403	**NIPSNAP3A**	+	12435	2.841	1171	1969	905	2.175	1.121	0.163	0
chr20	16710608	16722417	**SNRPB2**	+	11809	2.334	910	1530	703	2.175	1.121	0.177	0
chr9	94171326	94186144	**NFIL3**	-	14818	3.329	1615	2715	1249	2.174	1.12	0.179	0
chr7	143078359	143088206	**ZYX**	+	9847	12.467	4000	6712	3096	2.168	1.116	0.208	0
chr6	64281919	64293489	**PTP4A1**	+	11570	7.994	3018	5060	2337	2.165	1.114	0.201	0
chr4	79839093	79860582	**PAQR3**	-	21489	1.371	964	1614	747	2.159	1.11	0.198	0
chr14	103592663	103603776	**TNFAIP2**	+	11113	1.167	421	704	326	2.154	1.107	0.262	0
chr6	109783718	109804440	**ZBTB24**	-	20722	1.901	1286	2150	998	2.154	1.107	0.196	0
chr6	134274300	134308629	**TBPL1**	+	34329	1.979	2248	3756	1745	2.152	1.106	0.176	0
chr6	122720695	122754264	**HSF2**	+	33569	1.243	1373	2292	1066	2.15	1.104	0.182	0
chr11	9002122	9025596	**NRIP3**	-	23474	1.114	864	1442	671	2.15	1.104	0.19	0
chr11	65190268	65194003	**NEAT1**	+	3735	125.271	15485	25848	12031	2.148	1.103	0.308	0
chr11	89933596	89956532	**CHORDC1**	-	22936	2.986	2248	3750	1748	2.145	1.101	0.193	0
chr20	5986738	6020697	**CRLS1**	+	33959	1.804	2010	3350	1564	2.142	1.099	0.193	0
chr11	74699949	74718743	**NEU3**	+	18794	1.385	849	1415	660	2.141	1.099	0.208	0
chr5	75911306	75919240	**F2RL2**	-	7934	2.949	763	1271	593	2.141	1.098	0.215	0
chr1	115110180	115124265	**BCAS2**	-	14085	1.077	502	837	391	2.14	1.098	0.217	0
chrX	134021661	134049297	**MOSPD1**	-	27636	1.587	1427	2377	1110	2.14	1.098	0.202	0
chr6	56911383	56920023	**KIAA1586**	+	8640	2.755	786	1307	612	2.136	1.095	0.201	0
chr4	106603784	106629881	**INTS12**	-	26097	1.554	1350	2246	1051	2.135	1.094	0.17	0
chr7	12250847	12276890	**TMEM106B**	+	26043	1.557	1345	2238	1048	2.135	1.094	0.175	0
chr13	111365082	111373421	**ING1**	+	8339	1.256	340	564	265	2.128	1.09	0.289	0
chr11	14479048	14521441	**COPB1**	-	42393	4.321	6072	10076	4737	2.127	1.089	0.224	0
chr9	91606361	91619925	**S1PR3**	+	13564	1.478	653	1084	509	2.127	1.089	0.234	0
chr9	124101352	124132545	**STOM**	-	31193	6.329	6505	10784	5079	2.123	1.086	0.241	0
chr6	83874592	83903655	**PGM3**	-	29063	3.398	3253	5391	2540	2.122	1.086	0.205	0
chr8	73921096	73959987	**TERF1**	+	38891	1.172	1508	2494	1180	2.113	1.08	0.189	0
chr6	24650204	24667115	**TDP2**	-	16911	2.351	1304	2155	1020	2.112	1.079	0.202	0
chr6	151685249	151712677	**ZBTB2**	-	27428	2.615	2364	3906	1851	2.11	1.077	0.201	0
chr6	144324033	144329867	**HYMAI**	-	5834	1.322	254	419	198	2.109	1.076	0.296	0
chr13	45967453	45992516	**SLC25A30**	-	25063	1.201	989	1632	774	2.108	1.076	0.208	0
chr1	241792166	241799232	**CHML**	-	7066	3.469	824	1361	646	2.108	1.076	0.179	0
chr6	43543877	43588260	**POLH**	+	44383	1.655	2424	4000	1898	2.107	1.075	0.201	0
chr1	222885905	222906106	**BROX**	+	20201	2.825	1878	3098	1471	2.106	1.074	0.202	0
chr1	15943952	15986363	**DDI2**	+	42411	1.125	1568	2587	1228	2.106	1.074	0.202	0
chr3	155544300	155572248	**SLC33A1**	-	27948	1.127	1037	1710	812	2.105	1.074	0.204	0
chr17	78109012	78120982	**EIF4A3**	-	11970	3.368	1320	2176	1034	2.104	1.073	0.208	0
chr21	26955086	26955536	**LINC00515**	-	450	2.324	33	55	26	2.103	1.072	0.478	0
chr11	62559597	62572964	**NXF1**	-	13367	3.237	1408	2320	1104	2.101	1.071	0.215	0
chr15	43477465	43489375	**CCNDBP1**	+	11910	2.233	877	1445	688	2.101	1.071	0.208	0
chr5	34915819	34925787	**BRIX1**	+	9968	2.67	880	1449	690	2.099	1.069	0.208	0
chr12	22199158	22218602	**CMAS**	+	19444	1.294	832	1369	652	2.099	1.069	0.208	0
chr6	122764492	122792952	**SERINC1**	-	28460	5.609	5273	8679	4137	2.098	1.069	0.233	0
chr5	159828647	159846168	**SLU7**	-	17521	1.601	934	1535	733	2.094	1.066	0.199	0
chr5	110074753	110098484	**SLC25A46**	+	23731	2.17	1694	2786	1331	2.093	1.066	0.205	0
chr7	149128453	149158053	**ZNF777**	-	29600	1.244	1205	1981	947	2.093	1.065	0.21	0
chr9	35681989	35690053	**TPM2**	-	8064	30.272	8010	13152	6295	2.089	1.063	0.279	0
chr13	27844463	27847827	**RASL11A**	+	3364	1.515	165	271	130	2.089	1.063	0.366	0
chr12	10851675	10875953	**CSDA**	-	24278	5.344	4252	6979	3343	2.088	1.062	0.237	0
chr6	146119271	146135921	**FBXO30**	-	16650	4.993	2759	4529	2170	2.087	1.061	0.202	0
chr4	144434615	144478642	**SMARCA5**	+	44027	2.997	4342	7122	3415	2.085	1.06	0.233	0
chr6	106534194	106557814	**PRDM1**	+	23620	2.699	2120	3473	1669	2.08	1.057	0.198	0
chr6	24705089	24719403	**C6orf62**	-	14314	8.624	4078	6677	3212	2.079	1.056	0.225	0
chr8	104410865	104427468	**SLC25A32**	-	16603	3.823	2105	3446	1658	2.078	1.055	0.202	0

chr1	180942175	180992046	**STX6**	-	49871	2.354	3875	6339	3053	2.076	1.054	0.226	0
chr11	125439297	125454575	**EI24**	+	15278	1.647	823	1347	649	2.076	1.054	0.234	0
chrX	69353317	69386173	**IGBP1**	+	32856	1.36	1471	2405	1159	2.075	1.053	0.208	0
chr19	37717365	37734574	**ZNF383**	+	17209	1.01	572	935	451	2.072	1.051	0.248	0
chr1	211748380	211752099	**SLC30A1**	-	3719	6.175	740	1210	584	2.072	1.051	0.27	0
chr6	30509154	30525371	**GNL1**	-	16217	1.536	814	1329	642	2.069	1.049	0.24	0
chr14	58666832	58702353	**ACTR10**	+	35521	1.507	1780	2906	1405	2.068	1.048	0.2	0
chr12	44152746	44183346	**IRAK4**	+	30600	1.364	1374	2242	1085	2.066	1.047	0.209	0
chr4	109571740	109588980	**OSTC**	+	17240	2.396	1374	2239	1085	2.064	1.045	0.202	0
chr7	120590816	120615711	**ING3**	+	24895	1.832	1508	2458	1191	2.064	1.045	0.208	0
chr7	117824085	117844093	**NAA38**	+	20008	1.977	1309	2134	1034	2.063	1.045	0.208	0
chr1	90460677	90494094	**ZNF326**	+	33417	3.177	3529	5750	2789	2.061	1.044	0.216	0
chr14	57735605	57756797	**MUDENG**	+	21192	1.886	1329	2165	1050	2.061	1.043	0.202	0
chr1	87794150	87814607	**LMO4**	+	20457	1.058	707	1151	559	2.06	1.043	0.256	0
chr2	150426146	150444330	**MMADHC**	-	18184	5.522	3323	5410	2627	2.059	1.042	0.222	0
chr3	101547075	101579869	**NFKBIZ**	+	32794	9.461	10140	16507	8018	2.059	1.042	0.323	0
chr14	34902143	34931468	**SPTSSA**	-	29325	1.713	1660	2700	1313	2.056	1.04	0.209	0
chr3	113367232	113415493	**KIAA2018**	-	48261	1.455	2315	3766	1831	2.056	1.04	0.218	0
chr12	10365488	10375724	**GABARAPL1**	+	10236	9.203	3170	5156	2507	2.056	1.04	0.2	0
chr5	34905365	34915780	**RAD1**	-	10415	3.279	1124	1828	890	2.055	1.039	0.222	0
chr6	160182988	160200087	**ACAT2**	+	17099	2.042	1135	1844	898	2.053	1.038	0.245	0
chr6	2833733	2842081	**SERPINB1**	-	8348	2.646	729	1185	577	2.052	1.037	0.237	0
chrX	135579237	135594503	**HTATSF1**	+	15266	1.279	647	1050	513	2.048	1.034	0.24	0
chr12	59265936	59314262	**LRIG3**	-	48326	1.845	2942	4774	2331	2.048	1.034	0.228	0
chr13	50571142	50592603	**TRIM13**	+	21461	2.253	1609	2610	1275	2.046	1.033	0.208	0
chr7	86825477	86849031	**C7orf23**	-	23554	2.039	1575	2550	1249	2.041	1.029	0.231	0
chr11	59341870	59383617	**OSBP**	-	41747	2.559	3511	5685	2786	2.04	1.029	0.245	0
chr1	109606997	109618624	**TAF13**	-	11627	3.521	1355	2193	1075	2.039	1.028	0.216	0
chr8	90914095	90940095	**OSGIN2**	+	26000	2.779	2398	3882	1904	2.039	1.028	0.215	0
chr20	5918485	5931173	**TRMT6**	-	12688	1.208	503	814	399	2.039	1.028	0.279	0
chr4	25378847	25420120	**ANAPC4**	+	41273	1.445	1973	3192	1567	2.036	1.026	0.218	0
chr7	121713597	121784344	**AASS**	-	70747	1.011	2378	3838	1891	2.03	1.021	0.216	0
chr2	170655388	170668575	**SSB**	+	13187	1.548	677	1093	538	2.029	1.021	0.245	0
chr5	98264837	98266713	**LOC100289230**	+	1876	2.262	139	224	110	2.028	1.02	0.387	0
chr6	126307575	126360420	**TRMT11**	+	52845	1.468	2555	4121	2033	2.027	1.019	0.237	0
chr14	20779528	20801457	**CCNB1IP1**	-	21929	1.952	1412	2277	1124	2.025	1.018	0.228	0
chr1	114239823	114301777	**PHTF1**	-	61954	1.215	2477	3993	1972	2.025	1.018	0.237	0
chrX	131337052	131352189	**RAP2C**	-	15137	1.7	852	1371	679	2.019	1.014	0.239	0
chr3	143690639	143711210	**C3orf58**	+	20571	1.125	759	1221	605	2.018	1.013	0.265	0
chr4	166248817	166264314	**MSMO1**	+	15497	2.397	1206	1940	961	2.018	1.013	0.263	0
chr16	56518258	56554008	**BBS2**	-	35750	1.084	1272	2044	1014	2.015	1.011	0.243	0
chr15	34376223	34394053	**C15orf24**	-	17830	1.532	912	1466	728	2.014	1.01	0.224	0
chr9	132589563	132597572	**C9orf78**	-	8009	1.136	299	481	239	2.013	1.01	0.326	0
chr13	20207787	20247599	**MPHOSPH8**	+	39812	1.127	1476	2371	1177	2.013	1.009	0.238	0
chr14	35514112	35552589	**FAM177A1**	+	38477	1.476	1870	3005	1492	2.013	1.009	0.237	0
chr6	19837616	19840915	**ID4**	+	3299	3.474	374	601	298	2.012	1.009	0.326	0
chr4	56719815	56771244	**EXOC1**	+	51429	1.474	2520	4042	2012	2.008	1.006	0.23	0
chr17	34136487	34174238	**TAF15**	+	37751	2.581	3243	5196	2592	2.005	1.003	0.237	0
chr13	76123926	76180068	**UCHL3**	+	56142	1.237	2304	3692	1842	2.005	1.003	0.233	0
chr14	45393526	45431179	**KLHL28**	-	37653	1.601	1991	3191	1592	2.004	1.003	0.237	0
chr4	78078356	78091213	**CCNG2**	+	12857	3.425	1437	2302	1149	2.003	1.002	0.26	0
chr21	30428647	30446010	**CCT8**	-	17363	2.606	1504	2406	1203	2	1	0.227	0
chr9	125001833	125027143	**RBM18**	-	25310	2.015	1686	2697	1348	2	1	0.237	0
chr1	93811477	93828148	**DR1**	+	16671	3.807	2108	3373	1687	2	1	0.231	0
**Total**					**7563405**								
**Average size**					**23130**								

Table S4B. Genes DOWN-regulated >2-fold following 45 min CPT treatment, wash and 30 min recovery with Bru last 15 min (CPT+30m).

chrom	start	end	name	strand	bp	meanRPKM	meanCount	1_CPT30m	2_cont	foldChange	log2FoldChange	pAdjusted	significant
chr12	27849427	27850566	**REP15**	+	1139	2.006	81	2	107	0.026	-5.29174	0.003	1
chr6	26271145	26271612	**HIST1H3G**	-	467	10.928	170	7	225	0.032	-4.947603	0	1
chr6	26043454	26043885	**HIST1H2BB**	-	431	1.325	19	0	26	0.035	-4.851723	0.105	0
chr6	27840925	27841289	**HIST1H4L**	-	364	1.488	19	0	25	0.036	-4.800716	0.117	0
chr6	1312674	1314993	**FOXQ1**	+	2319	1.762	132	8	174	0.047	-4.409974	0.002	1
chr12	1726221	1756378	**WNT5B**	+	30157	3.788	3878	257	5085	0.051	-4.305523	0	1
chr5	140682195	140683612	**SLC25A2**	-	1417	1.654	78	5	103	0.053	-4.240848	0.012	1
chr6	26031816	26032288	**HIST1H3B**	-	472	12.07	195	13	255	0.054	-4.223076	0.001	1
chr6	27858092	27858570	**HIST1H3J**	-	478	2.995	48	3	63	0.058	-4.113252	0.034	1
chr18	57098170	57364644	**CCBE1**	-	266474	4.834	44746	3659	58441	0.063	-3.997126	0.061	1
chr13	28519342	28519710	**ATP5EP2**	+	368	3.407	41	3	54	0.067	-3.90394	0.057	1
chr6	27833106	27833576	**HIST1H2AL**	+	470	2.241	35	3	46	0.078	-3.67388	0.089	1
chr16	86600856	86602537	**FOXC2**	+	1681	2.308	124	13	161	0.085	-3.562034	0.013	1
chr10	78629358	79397577	**KCNMA1**	-	768219	2.744	71606	8201	92741	0.088	-3.499227	0.246	0
chr14	73136659	73360809	**DPF3**	-	224150	1.613	12385	1607	15978	0.101	-3.312927	0.009	1
chr12	6957971	6960456	**CDCA3**	-	2485	2.967	248	32	319	0.103	-3.284366	0.008	1
chr12	120123594	120315095	**CIT**	-	191501	1.381	9071	1242	11681	0.106	-3.23332	0.006	1
chr5	64444562	64777704	**ADAMTS6**	-	333142	2.006	24012	3304	30914	0.107	-3.225923	0.05	1
chr11	19372270	20143147	**NAV2**	+	770877	1.852	47996	6691	61765	0.108	-3.206413	0.16	0
chr6	26045638	26046097	**HIST1H3C**	+	459	7.919	123	17	158	0.109	-3.196891	0.029	1
chr5	43039181	43040447	**C5orf39**	-	1266	4.008	167	23	215	0.11	-3.182051	0.017	1
chr8	70378858	70573147	**SULF1**	+	194289	5.074	36463	5151	46900	0.11	-3.186686	0.122	0
chr16	56642477	56643409	**MT2A**	+	932	41.084	1263	179	1624	0.111	-3.176862	0.001	1
chr1	115828536	115880857	**NGF**	-	52321	5.428	9927	1447	12753	0.113	-3.139439	0.009	1
chr17	8108048	8113883	**AURKB**	-	5835	4.372	859	127	1104	0.116	-3.112146	0.001	1
chr22	46692637	46726707	**GTSE1**	+	34070	1.265	1452	216	1864	0.116	-3.108353	0.001	1
chr7	55086724	55275031	**EGFR**	+	188307	4.035	25792	4003	33055	0.121	-3.045482	0.066	1
chr6	160514113	160517244	**LOC729603**	+	3131	1.789	187	29	240	0.122	-3.04006	0.021	1
chr8	31497267	32622558	**NRG1**	+	1125291	1.252	49439	7707	63350	0.122	-3.039047	0.208	0
chr16	3022791	3030540	**PKMYT1**	-	7749	1.69	435	70	557	0.126	-2.988324	0.007	1
chr11	64844926	64851615	**CDCA5**	-	6689	2.113	474	77	607	0.128	-2.970147	0.006	1
chr13	60239722	60738119	**DIAPH3**	-	498397	1.466	25584	4191	32715	0.128	-2.964389	0.081	1
chr11	66099541	66104000	**RIN1**	-	4459	4.334	639	105	817	0.129	-2.949557	0.004	1
chr1	180238797	180243816	**LOC100527964**	-	5019	1.373	230	38	294	0.13	-2.944383	0.02	1
chr6	26199786	26200216	**HIST1H2BF**	+	430	1.139	16	2	20	0.131	-2.930065	0.307	0
chr19	47421932	47508333	**ARHGAP35**	+	86401	3.067	9076	1540	11588	0.133	-2.911324	0.013	1
chr11	65657874	65659106	**CCDC85B**	+	1232	3.107	122	20	156	0.134	-2.900675	0.041	1
chr8	119201694	119634184	**SAMD12**	-	432490	1.12	17100	2945	21818	0.135	-2.888822	0.047	1
chr1	43824625	43828873	**CDC20**	+	4248	2.637	380	66	485	0.137	-2.865221	0.013	1
chr2	54756358	54756978	**RPL23AP32**	+	620	1.95	41	7	53	0.138	-2.862154	0.178	0
chr14	95873603	95876427	**LINC00341**	-	2824	1.145	107	19	137	0.139	-2.8448	0.062	1
chr12	49716970	49725514	**TROAP**	+	8544	1.204	343	61	437	0.14	-2.840312	0.014	1
chr11	66624875	66627946	**LRFN4**	+	3071	1.292	131	23	166	0.142	-2.81436	0.052	1
chr2	40339285	40739575	**SLC8A1**	-	400290	1.953	28518	5144	36309	0.142	-2.819207	0.134	0
chr19	38397867	38699008	**SIPA1L3**	+	301141	1.441	14927	2710	18999	0.143	-2.80935	0.04	1
chr1	156495196	156542396	**IQGAP3**	-	47200	1.541	2484	459	3159	0.145	-2.781144	0.004	1
chr6	26027123	26027480	**HIST1H4B**	-	357	20.217	244	45	310	0.147	-2.767227	0.029	1
chr6	26188937	26189304	**HIST1H4D**	-	367	3.012	38	7	49	0.149	-2.75016	0.208	0
chr20	9049700	9461462	**PLCB4**	+	411762	4.281	64525	12173	81975	0.149	-2.751458	0.389	0
chr12	11148560	11150474	**TAS2R20**	-	1914	1.213	81	15	103	0.149	-2.743542	0.114	0
chr11	123396527	123493518	**GRAMD1B**	+	96991	1.35	4471	856	5676	0.151	-2.728702	0.008	1
chr16	1821895	1823140	**MRPS34**	-	1245	2.306	95	18	120	0.151	-2.725204	0.087	1
chr11	33563876	33695646	**C11orf41**	+	131770	1.436	6489	1249	8236	0.152	-2.720713	0.014	1
chr9	140657473	140659224	**FLJ40292**	+	1751	1.936	112	21	143	0.153	-2.710655	0.073	1
chr17	43003447	43025082	**KIF18B**	-	21635	1.914	1414	274	1794	0.153	-2.708346	0.005	1
chr6	27860476	27860963	**HIST1H2AM**	-	487	3.122	51	10	65	0.154	-2.703563	0.162	0
chr6	149721494	149722182	**SUMO4**	+	688	2.189	51	10	64	0.155	-2.688459	0.163	0
chr15	77516249	77517746	**C15orf5**	-	1497	3.379	179	36	227	0.16	-2.640127	0.065	1
chr16	2479394	2508859	**CCNF**	+	29465	1.276	1261	255	1596	0.16	-2.644144	0.006	1
chr14	101361106	101373305	**MEG8**	+	12199	2.261	938	190	1187	0.16	-2.639748	0.008	1
chr3	42132745	42267268	**TRAK1**	+	134523	2.427	11113	2248	14068	0.16	-2.645729	0.035	1
chr2	121554866	121750229	**GLI2**	+	195363	1.449	9712	1973	12292	0.161	-2.638901	0.03	1
chr2	9346893	9545812	**ASAP2**	+	198919	1.89	12728	2627	16095	0.163	-2.61493	0.045	1
chr22	24236564	24237409	**MIF**	+	845	10.429	292	60	369	0.163	-2.619151	0.03	1
chr16	69363899	69364498	**PDF**	-	599	1.164	22	4	28	0.163	-2.620796	0.288	0
chr10	49654078	49813138	**ARHGAP22**	-	159060	2.909	15530	3235	19629	0.165	-2.600793	0.061	1
chr3	27257096	27410912	**NEK10**	-	153816	1.651	8727	1819	11030	0.165	-2.599861	0.029	1

chr22	42334740	42343148	**CENPM**	-	8408	2.299	644	136	813	0.168	-2.572323	0.013	1
chr17	79609348	79615779	**TSPAN10**	+	6431	1.614	340	72	429	0.168	-2.575056	0.026	1
chr17	76210276	76221716	**BIRC5**	+	11440	2.764	1073	228	1355	0.169	-2.565957	0.009	1
chr11	119205236	119208022	**RNF26**	+	2786	3.943	364	77	459	0.169	-2.567958	0.026	1
chr19	18699494	18703147	**C19orf60**	+	3653	1.34	162	35	204	0.174	-2.522368	0.069	1
chr1	155112366	155112996	**DPM3**	-	630	1.801	37	8	47	0.174	-2.519866	0.232	0
chr19	17416476	17417652	**MRPL34**	+	1176	1.06	41	9	52	0.174	-2.525312	0.218	0
chr13	38210772	38443939	**TRPC4**	-	233167	1.637	13643	2995	17192	0.174	-2.520674	0.073	1
chr1	201617449	201796102	**NAV1**	+	178653	3.29	20405	4504	25705	0.175	-2.51269	0.117	0
chr5	178537851	178772431	**ADAMTS2**	-	234580	2.014	16104	3604	20271	0.178	-2.491649	0.084	1
chr6	33359312	33377699	**KIFC1**	+	18387	2.189	1366	306	1720	0.178	-2.488937	0.01	1
chr16	17196180	17564738	**XYLT1**	-	368558	1.611	20297	4550	25546	0.178	-2.488879	0.116	0
chr6	26033319	26033796	**HIST1H2AB**	-	477	2.737	44	10	56	0.179	-2.483133	0.228	0
chr9	36572904	36677679	**MELK**	+	104775	1.348	4867	1097	6123	0.179	-2.480673	0.02	1
chr19	11257830	11266484	**SPC24**	-	8654	1.122	328	73	413	0.179	-2.483758	0.04	1
chr1	214776531	214837914	**CENPF**	+	61383	2.311	4883	1106	6142	0.18	-2.473114	0.02	1
chr6	27839622	27840099	**HIST1H3I**	-	477	3.732	60	13	75	0.181	-2.466059	0.185	0
chr22	50171537	50173958	**LOC90834**	-	2421	2.056	168	38	212	0.181	-2.469325	0.082	1
chr20	44441254	44445596	**UBE2C**	+	4342	3.249	476	108	599	0.181	-2.465878	0.026	1
chr19	36545782	36596012	**WDR62**	+	50230	1.071	1805	411	2270	0.181	-2.464772	0.01	1
chr1	197053256	197115824	**ASPM**	-	62568	2.903	6320	1442	7946	0.182	-2.461402	0.029	1
chr19	18390562	18392432	**JUND**	-	1870	25.225	1530	352	1923	0.183	-2.449626	0.009	1
chr5	176732500	176739292	**MXD3**	-	6792	1.307	297	68	374	0.183	-2.451572	0.045	1
chr6	57182421	57513376	**PRIM2**	+	330955	1.557	17644	4067	22169	0.183	-2.446359	0.104	0
chr4	16503164	16900424	**LDB2**	-	397260	1.066	15270	3563	19172	0.186	-2.427786	0.105	0
chr6	42858002	42858554	**C6orf226**	-	552	1.72	31	7	38	0.187	-2.415714	0.287	0
chr5	113698015	113832197	**KCNN2**	+	134182	3.41	15785	3708	19811	0.187	-2.417567	0.099	1
chr1	38158158	38175391	**CDCA8**	+	17233	2.789	1635	388	2051	0.189	-2.40055	0.014	1
chr15	40453209	40513337	**BUB1B**	+	60128	1.03	2127	508	2667	0.191	-2.38977	0.015	1
chr15	41624891	41673248	**NUSAP1**	+	48357	1.265	2108	505	2642	0.191	-2.386795	0.016	1
chr14	71996041	72206120	**SIPA1L1**	+	210079	3.001	22138	5289	27755	0.191	-2.391517	0.16	0
chr2	56411257	56613309	**CCDC85A**	+	202052	7.522	55204	13297	69173	0.192	-2.379012	0.434	0
chr10	80828791	81076285	**ZMIZ1**	+	247494	4.52	37654	9068	47183	0.192	-2.379269	0.267	0
chr11	118964584	118966177	**H2AFX**	-	1593	6.016	315	76	395	0.194	-2.367915	0.049	1
chr7	36429431	36493400	**ANLN**	+	63969	4.713	10479	2557	13120	0.195	-2.359136	0.068	1
chr19	15348300	15391262	**BRD4**	-	42962	3.413	4923	1200	6164	0.195	-2.360593	0.026	1
chr6	30655825	30658769	**NRM**	-	2944	1.015	100	24	125	0.196	-2.349756	0.15	0
chr9	128199672	128469513	**MAPKAP1**	-	269841	1.79	16580	4081	20746	0.197	-2.345814	0.116	0
chr14	71954577	71956420	**LOC145474**	+	1843	2.717	176	43	221	0.198	-2.336994	0.111	0
chr15	81293294	81296345	**MESDC1**	+	3051	3.973	394	97	493	0.198	-2.339657	0.039	1
chr6	27107087	27107457	**HIST1H4I**	+	370	1.484	18	4	22	0.199	-2.332178	0.402	0
chr12	2966846	2986321	**FOXM1**	-	19475	1.281	842	211	1053	0.201	-2.315403	0.022	1
chr6	27100816	27101314	**HIST1H2AG**	+	498	3.143	54	13	68	0.201	-2.316216	0.256	0
chr8	21995532	21999448	**REEP4**	-	3916	1.271	166	41	208	0.201	-2.31159	0.104	0
chr17	74380689	74383941	**SPHK1**	+	3252	7.609	814	204	1018	0.201	-2.317801	0.02	1
chr19	46386865	46389376	**IRF2BP1**	-	2511	1.303	107	27	134	0.204	-2.294833	0.15	0
chr6	27806439	27806888	**HIST1H2BN**	+	449	1.867	28	7	35	0.205	-2.283634	0.355	0
chr14	95883830	95942173	**C14orf49**	-	58343	1.259	2449	628	3055	0.206	-2.281942	0.02	1
chr9	126141932	126692417	**DENND1A**	-	550485	1.275	23858	6161	29757	0.207	-2.271901	0.193	0
chr4	2627158	2734302	**FAM193A**	+	107144	1.518	5551	1430	6924	0.207	-2.274842	0.04	1
chr16	81698958	81700879	**LOC100129617**	+	1921	1.606	105	27	131	0.207	-2.270166	0.166	0
chr10	98741040	98745585	**C10orf12**	+	4545	1.595	249	64	311	0.208	-2.265731	0.09	1
chr1	240255184	240638489	**FMN2**	+	383305	5.564	75679	19667	94350	0.208	-2.262225	0.578	0
chr7	83587658	83824217	**SEMA3A**	-	236559	2.374	20062	5205	25015	0.208	-2.264637	0.182	0
chr5	137514416	137523404	**KIF20A**	+	8988	2.283	705	183	879	0.209	-2.261635	0.034	1
chr2	234745485	234763212	**HJURP**	-	17727	5.728	3420	895	4262	0.21	-2.250855	0.027	1
chr4	1873122	1983934	**WHSC1**	+	110812	2.366	8936	2339	11134	0.21	-2.250954	0.067	1
chr1	46085715	46089731	**CCDC17**	-	4016	1.016	135	35	168	0.211	-2.247599	0.139	0
chr5	126112314	126172712	**LMNB1**	+	60398	1.935	4027	1065	5015	0.212	-2.235225	0.035	1
chr9	127115751	127121463	**LOC100129034**	+	5712	3.524	674	177	840	0.212	-2.239866	0.033	1
chr17	78518624	78940173	**RPTOR**	+	421549	1.508	21473	5673	26740	0.212	-2.236681	0.176	0
chr1	97543299	98386615	**DPYD**	-	843316	1.477	44103	11684	54909	0.213	-2.232454	0.385	0
chr3	71003864	71633140	**FOXP1**	-	629276	1.345	29674	7869	36943	0.213	-2.230929	0.266	0
chr18	2571509	2616634	**NDC80**	+	45125	1.18	1841	488	2291	0.213	-2.229174	0.026	1
chr15	91509267	91537804	**PRC1**	-	28537	2.098	2042	541	2543	0.213	-2.230904	0.025	1
chr5	169010637	169031781	**CCDC99**	+	21144	6.35	4723	1257	5879	0.214	-2.224871	0.045	1
chr17	12569206	12670651	**MYOCD**	+	101445	1.331	4987	1329	6206	0.214	-2.22273	0.061	1
chr15	40763159	40765357	**CHST14**	+	2198	1.428	104	28	129	0.218	-2.199352	0.175	0
chr17	3627196	3629992	**GSG2**	+	2796	1.525	143	39	177	0.22	-2.181312	0.15	0
chr4	1723216	1746905	**TACC3**	+	23689	1.799	1435	392	1782	0.22	-2.18453	0.026	1
chr6	27782821	27783267	**HIST1H2BM**	+	446	1.713	26	7	32	0.222	-2.172705	0.413	0

chr16	67973786	67977376	**LCAT**	-	3590	1.977	235	64	291	0.222	-2.172041	0.101	0
chr8	27667137	27695349	**PBK**	-	28212	1.49	1440	401	1787	0.225	-2.154988	0.032	1
chr5	68462836	68474070	**CCNB1**	+	11234	3.468	1336	374	1657	0.226	-2.144581	0.033	1
chr1	208195587	208417665	**PLXNA2**	-	222078	1.178	9084	2546	11263	0.226	-2.145152	0.094	1
chr5	14143828	14509458	**TRIO**	+	365630	7.827	99143	27917	122885	0.227	-2.138081	0.712	0
chr3	63989697	63997917	**LOC100507062**	+	8220	2.52	719	203	891	0.228	-2.132519	0.051	1
chr15	64791618	64978266	**ZNF609**	+	186648	1.035	6713	1895	8319	0.228	-2.134192	0.073	1
chr12	50382944	50419307	**RACGAP1**	-	36363	1.606	2019	571	2502	0.229	-2.12948	0.036	1
chr17	61086897	61505067	**TANC2**	+	418170	2.926	43403	12312	53767	0.229	-2.126665	0.415	0
chr6	26250369	26250835	**HIST1H3F**	-	466	3.422	54	15	67	0.23	-2.122324	0.296	0
chr10	129894924	129924468	**MKI67**	-	29544	3.991	4034	1149	4995	0.23	-2.12016	0.048	1
chr6	34214156	34216904	**C6orf1**	-	2748	2.249	204	58	252	0.231	-2.115049	0.122	0
chr2	54080049	54087170	**GPR75**	-	7121	2.018	485	138	601	0.231	-2.116507	0.065	1
chr1	45205489	45233438	**KIF2C**	+	27949	1.459	1382	395	1710	0.231	-2.11185	0.034	1
chr19	45971252	45978437	**FOSB**	+	7185	19.026	4426	1271	5478	0.232	-2.10744	0.04	1
chr8	37553300	37556396	**ZNF703**	+	3096	4.423	438	125	542	0.232	-2.10803	0.059	1
chr5	139927250	139929163	**EIF4EBP3**	+	1913	1.241	79	22	97	0.233	-2.102053	0.236	0
chr16	2570362	2580955	**AMDHD2**	+	10593	2.451	851	247	1053	0.235	-2.091738	0.038	1
chr11	63742078	63744015	**COX8A**	+	1937	7.142	459	133	567	0.235	-2.092093	0.067	1
chr10	128594022	129250780	**DOCK1**	+	656758	1.114	24905	7239	30793	0.235	-2.088681	0.252	0
chr12	64238540	64541613	**SRGAP1**	+	303073	4.622	49019	14240	60612	0.235	-2.089577	0.458	0
chr10	320129	735608	**DIP2C**	-	415479	1.592	22424	6533	27720	0.236	-2.085016	0.226	0
chr11	102706527	102714342	**MMP3**	-	7815	1.356	353	103	437	0.236	-2.085755	0.087	1
chr17	55333930	55757299	**MSI2**	+	423369	1.268	18412	5368	22760	0.236	-2.084059	0.2	0
chr1	156182778	156209868	**PMF1**	+	27090	9.941	8558	2507	10575	0.237	-2.076604	0.073	1
chr1	209848669	209849735	**G0S2**	+	1066	4.913	173	51	214	0.238	-2.069476	0.15	0
chr9	140004991	140009195	**DPP7**	-	4204	1.052	145	42	179	0.239	-2.065597	0.164	0
chr8	131094983	131097014	**LOC100507117**	-	2031	5.164	362	106	447	0.239	-2.067309	0.102	0
chr9	82186877	82341656	**TLE4**	+	154779	21.567	119440	35205	147518	0.239	-2.067034	0.839	0
chr1	202300784	202311094	**UBE2T**	-	10310	1.191	423	124	522	0.239	-2.064374	0.09	1
chr2	225629806	225907330	**DOCK10**	-	277524	4.441	43216	12810	53351	0.24	-2.058249	0.434	0
chr2	242615156	242626383	**DTYMK**	-	11227	1.489	561	166	693	0.241	-2.05465	0.065	1
chr5	43014830	43018913	**LOC648987**	-	4083	2.047	281	83	347	0.241	-2.051328	0.116	0
chr4	160188997	160281301	**RAPGEF2**	+	92304	1.819	6006	1785	7413	0.241	-2.053718	0.092	1
chr8	118811601	119124058	**EXT1**	-	312457	11.776	127863	38216	157745	0.242	-2.045324	0.862	0
chr13	100003673	100004281	**FKSG29**	+	608	1.025	21	6	26	0.242	-2.046083	0.475	0
chr17	1958392	1962981	**HIC1**	+	4589	3.443	513	153	634	0.242	-2.049073	0.062	1
chr4	150999425	151178608	**DCLK2**	+	179183	10.46	66658	20075	82186	0.244	-2.033491	0.592	0
chr8	128806778	129113499	**PVT1**	+	306721	2.549	26437	7956	32597	0.244	-2.034571	0.279	0
chr1	154947117	154951725	**CKS1B**	+	4608	1.528	241	72	298	0.245	-2.03088	0.147	0
chr12	53645369	53648190	**MFSD5**	+	2821	2.327	217	65	267	0.245	-2.026849	0.142	0
chr12	24962957	25102393	**BCAT1**	-	139436	1.198	5853	1772	7213	0.246	-2.025307	0.089	1
chr6	26020717	26021186	**HIST1H3A**	+	469	3.227	51	15	63	0.246	-2.025821	0.327	0
chr14	21668237	21675059	**LOC283624**	-	6822	7.435	1710	518	2107	0.246	-2.021833	0.043	1
chr19	1911888	1913446	**ADAT3**	+	1558	1.7	86	26	107	0.247	-2.018376	0.234	0
chr2	235401685	235405693	**ARL4C**	-	4008	1.414	188	57	232	0.247	-2.018367	0.157	0
chr10	855483	931702	**LARP4B**	-	76219	2.403	6241	1899	7688	0.247	-2.01693	0.086	1
chr3	29322802	30051886	**RBMS3**	+	729084	1.149	29866	9088	36792	0.247	-2.017348	0.352	0
chr19	7745706	7747748	**TRAPPC5**	+	2042	1.587	107	32	132	0.248	-2.011651	0.218	0
chr12	72666528	73059422	**TRHDE**	+	392894	2.346	32355	9877	39848	0.248	-2.012228	0.366	0
chr17	38544772	38574202	**TOP2A**	-	29430	3.597	3676	1128	4525	0.249	-2.004173	0.067	1
chr3	10326102	10327430	**GHRLOS2**	+	1328	1.866	82	25	102	0.25	-1.999537	0.262	0
chr1	163291722	163325553	**NUF2**	+	33831	1.604	1852	576	2277	0.253	-1.982604	0.054	1
chr22	42556018	42611445	**TCF20**	-	55427	1.036	1965	611	2417	0.253	-1.984116	0.055	1
chr17	75277491	75496678	**9-Sep**	+	219187	3.078	22468	7018	27617	0.254	-1.976311	0.254	0
chr9	18474078	18910947	**ADAMTSL1**	+	436869	1.298	19057	5946	23427	0.254	-1.978153	0.222	0
chr15	76551629	76552493	**TYRO3P**	-	864	2.138	61	19	75	0.254	-1.975694	0.312	0
chr14	51955854	52197444	**FRMD6**	+	241590	14.313	121808	38287	149648	0.256	-1.966622	0.861	0
chr17	76170159	76183285	**TK1**	-	13126	2.688	1180	373	1449	0.258	-1.954867	0.054	1
chr9	16409500	16870786	**BNC2**	-	461286	1.634	26044	8266	31970	0.259	-1.951393	0.319	0
chr5	72921982	73237818	**RGNEF**	+	315836	1.213	13021	4134	15984	0.259	-1.950999	0.176	0
chr11	62518434	62521656	**ZBTB3**	-	3222	1.045	111	35	137	0.259	-1.946696	0.233	0
chr20	30326903	30389603	**TPX2**	+	62700	2.022	4357	1401	5342	0.262	-1.930406	0.086	1
chr9	139971952	139978990	**UAP1L1**	+	7038	4.126	949	307	1163	0.264	-1.92016	0.058	1
chr6	655938	656964	**HUS1B**	-	1026	1.815	61	20	75	0.265	-1.913916	0.324	0
chr15	75931425	75932664	**IMP3**	-	1239	4.963	205	66	251	0.265	-1.91817	0.176	0
chr20	49575362	49577820	**MOCS3**	+	2458	2.04	165	53	203	0.265	-1.91801	0.198	0
chr4	122737598	122745088	**CCNA2**	-	7490	4.413	1142	372	1398	0.266	-1.910379	0.071	1
chr2	113495443	113522254	**CKAP2L**	-	26811	1.764	1626	530	1992	0.266	-1.908149	0.067	1
chr2	27008881	27017455	**CENPA**	+	8574	1.834	526	172	644	0.267	-1.903174	0.1	1
chr16	771157	772590	**FAM173A**	+	1433	1.061	50	16	61	0.268	-1.90149	0.366	0

chr9	128509616	128729655	**PBX3**	+	220039	5.005	37156	12240	45462	0.269	-1.893064	0.421	0
chr16	23690200	23701688	**PLK1**	+	11488	3.571	1381	454	1691	0.269	-1.89658	0.064	1
chr18	46065426	46389586	**CTIF**	+	324160	2.698	30036	9920	36741	0.27	-1.888894	0.376	0
chr11	842823	867116	**TSPAN4**	+	24293	1.412	1136	375	1390	0.27	-1.887458	0.063	1
chr9	127019884	127114719	**NEK6**	+	94835	3.976	12573	4173	15373	0.271	-1.88118	0.183	0
chr4	157682762	157892546	**PDGFC**	-	209784	3.734	26888	8901	32883	0.271	-1.885313	0.35	0
chr3	187871662	188608460	**LPP**	+	736798	2.181	55615	18494	67989	0.272	-1.878214	0.568	0
chr16	70721341	70835061	**VAC14**	-	113720	1.699	6379	2126	7796	0.273	-1.874139	0.106	0
chr18	55313658	55470327	**ATP8B1**	-	156669	4.452	23928	8020	29230	0.274	-1.865785	0.324	0
chr8	25316512	25365425	**CDCA2**	+	48913	1.553	2599	870	3175	0.274	-1.866123	0.08	1
chr7	158424002	158497520	**NCAPG2**	-	73518	1.362	3421	1143	4180	0.274	-1.869935	0.087	1
chr13	33677271	34250932	**STARD13**	-	573661	1.262	25085	8408	30644	0.274	-1.865692	0.347	0
chr9	113636053	113800365	**LPAR1**	-	164312	6.456	36396	12239	44448	0.275	-1.860642	0.44	0
chr1	94027348	94147385	**BCAR3**	-	120037	7.624	31085	10460	37960	0.276	-1.85953	0.387	0
chr6	27861202	27861669	**HIST1H2BO**	+	467	1.334	21	7	26	0.276	-1.857856	0.543	0
chr20	37554954	37581703	**FAM83D**	+	26749	2.273	2055	694	2508	0.277	-1.853857	0.073	1
chr20	34541538	34543281	**SCAND1**	-	1743	1.272	72	24	88	0.277	-1.851511	0.316	0
chr3	73110809	73112471	**EBLN2**	+	1662	3.504	198	67	242	0.278	-1.846119	0.213	0
chr9	131483148	131486408	**ZDHHC12**	-	3260	1.308	142	48	173	0.278	-1.84621	0.242	0
chr11	61891444	61920635	**INCENP**	+	29191	1.318	1288	437	1571	0.279	-1.844234	0.071	1
chr8	59717976	60031767	**TOX**	-	313791	2.673	29371	10011	35824	0.279	-1.839352	0.398	0
chrY	14813159	14972768	**USP9Y**	+	159609	1.47	8103	2766	9882	0.28	-1.83703	0.16	0
chr12	2162415	2807115	**CACNA1C**	+	644700	2.521	55168	18876	67266	0.281	-1.833279	0.569	0
chr16	81478774	81745367	**CMIP**	+	266593	1.511	13598	4669	16575	0.282	-1.827711	0.215	0
chr10	91461366	91534700	**KIF20B**	+	73334	1.241	3151	1081	3841	0.282	-1.828479	0.101	0
chr11	68771861	68780850	**MRGPRF**	-	8989	1.092	325	112	396	0.283	-1.82004	0.157	0
chr6	128289923	128841870	**PTPRK**	-	551947	4.092	77971	26854	95009	0.283	-1.822888	0.693	0
chr4	104026962	104119566	**CENPE**	-	92604	1.203	3843	1329	4681	0.284	-1.81577	0.109	0
chr2	36583369	36778278	**CRIM1**	+	194909	25.497	175892	60844	214242	0.284	-1.816042	1	0
chr15	81071711	81243999	**KIAA1199**	+	172288	9.518	57753	19954	70353	0.284	-1.817884	0.608	0
chr1	47897806	47900313	**MGC12982**	-	2507	1.435	118	41	143	0.285	-1.809447	0.267	0
chr12	66151800	66220754	**RPSAP52**	-	68954	1.141	2597	900	3162	0.285	-1.812928	0.078	1
chr16	72816785	73092534	**ZFHX3**	-	275749	2.559	24266	8434	29544	0.285	-1.808548	0.355	0
chr10	94352824	94415152	**KIF11**	+	62328	1.57	3384	1180	4119	0.286	-1.803536	0.107	0
chr6	26217147	26217711	**HIST1H2AE**	+	564	5.826	112	39	136	0.287	-1.798624	0.3	0
chr11	12132137	12285331	**MICAL2**	+	153194	4.813	25191	8828	30645	0.288	-1.795485	0.365	0
chr4	72053002	72437804	**SLC4A4**	+	384802	1.158	15913	5573	19359	0.288	-1.796455	0.295	0
chr6	80714321	80752244	**TTK**	+	37923	1.368	1783	625	2169	0.288	-1.794183	0.094	1
chr10	125505151	125651500	**CPXM2**	-	146349	1.567	7726	2712	9398	0.289	-1.792889	0.155	0
chr20	30193085	30194317	**ID1**	+	1232	143.871	5841	2053	7104	0.289	-1.790318	0.12	0
chr14	38677203	38682268	**SSTR1**	+	5065	24.709	4169	1467	5070	0.289	-1.788924	0.104	0
chr19	11039423	11040916	**C19orf52**	+	1493	1.669	82	29	100	0.29	-1.784069	0.339	0
chr8	126104082	126379367	**NSMCE2**	+	275285	1.03	9765	3444	11873	0.29	-1.785256	0.2	0
chr19	4909509	4962165	**UHRF1**	+	52656	1.568	2772	978	3370	0.29	-1.78415	0.093	1
chr19	1275519	1279243	**C19orf24**	+	3724	1.331	162	57	197	0.291	-1.781551	0.234	0
chr11	9800213	10315754	**SBF2**	-	515541	1.185	21238	7527	25809	0.292	-1.777598	0.345	0
chr20	62329994	62339355	**ARFRP1**	-	9361	1.595	493	176	599	0.293	-1.768802	0.134	0
chr6	42192668	42419783	**TRERF1**	-	227115	1.814	13926	4962	16915	0.293	-1.769257	0.242	0
chr1	243651534	244006886	**AKT3**	-	355352	1.856	23081	8236	28029	0.294	-1.766876	0.375	0
chr19	2428163	2456958	**LMNB2**	-	28795	1.242	1198	427	1455	0.294	-1.766898	0.091	1
chr17	26904582	26926056	**SPAG5**	-	21474	1.236	901	321	1094	0.294	-1.765644	0.106	0
chr8	25042286	25270619	**DOCK5**	+	228333	4.312	34171	12267	41472	0.296	-1.757323	0.465	0
chr11	6340175	6341740	**PRKCDBP**	-	1565	12.997	665	238	808	0.296	-1.758128	0.108	0
chr4	20255234	20620788	**SLIT2**	+	365554	3.127	40075	14389	48637	0.296	-1.757053	0.519	0
chr20	62152132	62153524	**PPDPF**	+	1392	8.681	397	143	482	0.297	-1.752761	0.153	0
chr2	235860627	235964358	**SH3BP4**	+	103731	6.952	24272	8751	29446	0.297	-1.750483	0.364	0
chr12	7260903	7274447	**MATL2963**	+	13544	1.033	470	170	570	0.299	-1.743186	0.153	0
chr1	66999824	67210768	**SGIP1**	+	210944	7.962	57961	20985	70286	0.299	-1.743877	0.622	0
chr4	148653452	148993927	**ARHGAP10**	+	340475	1.763	20625	7491	25003	0.3	-1.73888	0.347	0
chr1	149858524	149858961	**HIST2H2AC**	+	437	3.633	54	20	66	0.301	-1.730701	0.449	0
chr11	67776047	67796743	**ALDH3B1**	+	20696	1.594	1098	401	1330	0.302	-1.729293	0.101	0
chr20	54944444	54967351	**AURKA**	-	22907	1.683	1319	482	1598	0.302	-1.728327	0.108	0
chr2	238395877	238463961	**MLPH**	+	68084	1.649	3732	1366	4520	0.302	-1.726449	0.114	0
chr17	64298925	64806862	**PRKCA**	+	507937	3.685	64273	23493	77866	0.302	-1.72875	0.655	0
chr13	114747193	114898095	**RASA3**	-	150902	1.91	9614	3522	11645	0.302	-1.72526	0.2	0
chr1	203595914	203713209	**ATP2B4**	+	117295	6.742	27029	9909	32736	0.303	-1.723942	0.406	0
chr7	42000547	42276618	**GLI3**	-	276071	1.317	12375	4539	14988	0.303	-1.723347	0.244	0
chr3	127407908	127542051	**MGLL**	-	134143	1.49	6735	2473	8156	0.303	-1.721465	0.163	0
chr10	75910942	76469061	**ADK**	+	558119	1.551	29943	11019	36251	0.304	-1.717936	0.449	0
chr6	101846860	102517958	**GRIK2**	+	671098	2.039	48947	18040	59249	0.304	-1.715582	0.604	0
chr12	58120022	58122139	**LOC100130776**	+	2117	1.419	99	36	120	0.304	-1.719345	0.324	0

chr10	133747959	133770053	**PPP2R2D**	+	22094	1.108	827	304	1001	0.304	-1.717418	0.121	0
chr18	52889561	53303188	**TCF4**	-	413627	2.949	43877	16168	53114	0.304	-1.715962	0.579	0
chr2	43449540	43453745	**ZFP36L2**	-	4205	28.687	3953	1455	4786	0.304	-1.71737	0.113	0
chr6	26240653	26241021	**HIST1H4F**	+	368	3.386	42	15	50	0.305	-1.715007	0.469	0
chr5	1253286	1295162	**TERT**	-	41876	1.761	2429	897	2940	0.305	-1.712223	0.101	0
chr10	62538088	62554610	**CDK1**	+	16522	2.7	1523	564	1843	0.306	-1.707163	0.113	0
chr4	17812524	17846487	**NCAPG**	+	33963	2.375	2785	1030	3370	0.306	-1.709645	0.127	0
chr21	36160097	36421595	**RUNX1**	-	261498	4.31	38454	14237	46526	0.306	-1.708365	0.505	0
chr19	51226604	51228981	**CLEC11A**	+	2377	3.486	272	101	330	0.307	-1.705248	0.208	0
chr10	103825123	103827795	**HPS6**	+	2672	2.404	211	78	255	0.307	-1.70301	0.234	0
chr19	42580289	42585720	**ZNF574**	+	5431	2.23	400	148	484	0.307	-1.705827	0.171	0
chr11	126293395	126870766	**KIRREL3**	-	577371	1.505	30272	11260	36609	0.308	-1.700928	0.457	0
chr5	108083522	108523373	**FER**	+	439851	1.272	19422	7250	23479	0.309	-1.695283	0.359	0
chr1	53971905	54199877	**GLIS1**	-	227972	1.468	11275	4215	13628	0.309	-1.692927	0.234	0
chr12	31944118	31945175	**H3F3C**	-	1057	1.586	56	20	67	0.309	-1.694959	0.443	0
chr9	99148224	99180669	**ZNF367**	-	32445	1.283	1425	533	1723	0.31	-1.691549	0.119	0
chr16	70284133	70285833	**EXOSC6**	-	1700	2.382	131	49	158	0.311	-1.685807	0.289	0
chr10	33466418	33623833	**NRP1**	-	157415	8.299	44820	16848	54144	0.311	-1.684198	0.564	0
chr5	9035137	9546233	**SEMA5A**	-	511096	2.315	40526	15220	48961	0.311	-1.68563	0.532	0
chr17	42634811	42638630	**FZD2**	+	3819	4.699	594	224	718	0.312	-1.678919	0.148	0
chr10	58117198	58121034	**ZWINT**	-	3836	2.253	290	109	350	0.312	-1.680259	0.221	0
chr1	20915443	20945400	**CDA**	+	29957	1.243	1274	482	1539	0.313	-1.673542	0.124	0
chr7	139246315	139477693	**HIPK2**	-	231378	1.734	13726	5182	16573	0.313	-1.677073	0.286	0
chr8	23386362	23430063	**SLC25A37**	+	43701	1.326	1938	732	2340	0.313	-1.676453	0.112	0
chr11	64002055	64006736	**VEGFB**	+	4681	1.263	195	73	236	0.313	-1.676965	0.261	0
chr6	16299342	16761721	**ATXN1**	-	462379	2.681	42329	16044	51091	0.314	-1.671057	0.549	0
chr6	33589155	33664348	**ITPR3**	+	75193	1.571	3896	1475	4703	0.314	-1.672416	0.128	0
chr12	60083125	60175408	**SLC16A7**	+	92283	1.288	4154	1576	5013	0.314	-1.668873	0.166	0
chr4	185615218	185655286	**MLF1IP**	-	40068	1.04	1413	537	1705	0.315	-1.666891	0.121	0
chr19	1241748	1244824	**ATP5D**	+	3076	1.51	153	58	184	0.316	-1.661171	0.292	0
chr1	212208918	212278187	**DTL**	+	69269	1.044	2463	943	2970	0.317	-1.655495	0.135	0
chr12	66218239	66360071	**HMGA2**	+	141832	9.934	46461	17753	56031	0.317	-1.658168	0.556	0
chr12	52626953	52642709	**KRT7**	+	15756	1.131	636	243	767	0.317	-1.656438	0.197	0
chr2	135011829	135212192	**MGAT5**	+	200363	1.19	8299	3172	10008	0.317	-1.657312	0.229	0
chr12	1100403	1605099	**ERC1**	+	504696	1.025	17917	6877	21597	0.318	-1.650901	0.36	0
chr5	169290718	169407744	**FAM196B**	-	117026	2.193	8905	3409	10737	0.318	-1.655173	0.237	0
chr2	168810529	169104105	**STK39**	-	293576	1.729	17410	6692	20983	0.319	-1.648651	0.348	0
chr11	12695968	12966284	**TEAD1**	+	270316	4.245	39456	15173	47550	0.319	-1.647854	0.543	0
chr1	1309109	1310818	**AURKAIP1**	-	1709	2.175	122	47	147	0.322	-1.634726	0.33	0
chr11	65684282	65686531	**C11orf68**	-	2249	6.666	496	192	598	0.322	-1.636448	0.175	0
chr11	95711439	96076344	**MAML2**	-	364905	4.09	52442	20353	63138	0.322	-1.633266	0.637	0
chrX	9431334	9687780	**TBL1X**	+	256446	1.102	9614	3728	11575	0.322	-1.634548	0.242	0
chr11	832951	838835	**CD151**	+	5884	3.798	741	288	892	0.323	-1.630564	0.148	0
chr15	99192760	99507759	**IGF1R**	+	314999	1.758	19129	7442	23025	0.323	-1.629282	0.379	0
chr17	40724328	40729747	**PSMC3IP**	-	5419	1.14	206	80	248	0.323	-1.629851	0.276	0
chr1	44173217	44396831	**ST3GAL3**	+	223614	1.094	8349	3243	10052	0.323	-1.632075	0.226	0
chr9	140135710	140138159	**TUBB4B**	+	2449	14.454	1171	455	1410	0.323	-1.632164	0.121	0
chr16	85645028	85709812	**KIAA0182**	+	64784	1.064	2300	896	2768	0.324	-1.626968	0.129	0
chr1	162602227	162750247	**DDR2**	+	148020	4.187	21141	8262	25434	0.325	-1.622064	0.391	0
chr6	144612872	145174170	**UTRN**	+	561298	1.804	35127	13749	42253	0.325	-1.619686	0.528	0
chr6	26021906	26022278	**HIST1H4A**	+	372	2.054	25	10	30	0.326	-1.618495	0.57	0
chr19	43671894	43690688	**PSG5**	-	18794	2.873	1890	740	2273	0.326	-1.6183	0.156	0
chr4	152041432	152149182	**SH3D19**	-	107750	1.179	4477	1753	5385	0.326	-1.618599	0.2	0
chr4	159587830	159593202	**C4orf46**	-	5372	1.75	317	124	381	0.327	-1.611416	0.243	0
chr3	11178778	11304939	**HRH1**	+	126161	1.302	5543	2176	6665	0.327	-1.61441	0.185	0
chr15	75628373	75632614	**COMMD4**	+	4241	1.31	184	72	222	0.328	-1.606116	0.296	0
chr2	215593274	215674428	**BARD1**	-	81154	2.253	6258	2477	7519	0.329	-1.601973	0.208	0
chr6	26225382	26225844	**HIST1H3E**	+	462	4.126	62	24	74	0.329	-1.604759	0.435	0
chr1	120454175	120612317	**NOTCH2**	-	158142	1.659	8992	3562	10802	0.33	-1.600521	0.254	0
chr3	114056946	114866127	**ZBTB20**	-	809181	1.142	32123	12721	38591	0.33	-1.600989	0.515	0
chr9	139006426	139010731	**C9orf69**	-	4305	1.859	264	104	317	0.331	-1.596175	0.254	0
chr17	80009762	80015346	**GPS1**	+	5584	1.556	288	114	347	0.331	-1.594478	0.25	0
chr3	65339905	66024509	**MAGI1**	-	684604	1.271	30157	11975	36217	0.331	-1.596604	0.497	0
chr6	56322784	56507694	**DST**	-	184910	6.19	39698	15818	47657	0.332	-1.591081	0.575	0
chr4	2932287	2936586	**MFSD10**	-	4299	1.577	222	88	266	0.332	-1.592545	0.272	0
chr3	123331142	123603149	**MYLK**	-	272007	6.769	61811	24651	74198	0.332	-1.589712	0.674	0
chr6	33665345	33679504	**C6orf125**	-	14159	1.029	491	196	589	0.334	-1.58094	0.208	0
chr20	3764497	3767337	**CENPB**	-	2840	3.659	343	137	412	0.334	-1.58177	0.229	0
chr3	172472297	172539263	**ECT2**	+	66966	2.019	4657	1865	5588	0.334	-1.582516	0.2	0
chr14	105475909	105487425	**CDCA4**	-	11516	2.79	1080	434	1296	0.335	-1.578073	0.151	0
chr22	46481876	46509808	**MIRLET7BHG**	+	27932	5.606	5198	2094	6232	0.336	-1.572949	0.185	0

chr20	3776400	3786761	**CDC25B**	+	10361	3.657	1261	510	1511	0.338	-1.565715	0.148	0
chr1	210111537	210337633	**SYT14**	+	226096	1.285	9665	3915	11582	0.338	-1.564808	0.259	0
chr10	69681655	69835103	**HERC4**	-	153448	8.701	45376	18412	54365	0.339	-1.561995	0.608	0
chr22	50354142	50357720	**PIM3**	+	3578	3.344	390	158	468	0.339	-1.560326	0.214	0
chr7	137559724	137686846	**CREB3L2**	-	127122	5.068	22244	9046	26644	0.34	-1.558442	0.446	0
chr3	192514604	192635950	**MB21D2**	-	121346	4.264	17732	7225	21234	0.34	-1.555191	0.388	0
chr11	28129797	28355054	**METTL15**	+	225257	1.081	8498	3462	10176	0.34	-1.555194	0.279	0
chr9	140172279	140177093	**C9orf167**	+	4814	2.623	413	168	494	0.341	-1.551239	0.212	0
chr12	116396380	116714991	**MED13L**	-	318611	3.794	42389	17284	50758	0.341	-1.554174	0.611	0
chr20	45838380	45985474	**ZMYND8**	-	147094	1.325	6631	2705	7940	0.341	-1.55357	0.231	0
chr13	30083550	30169825	**SLC7A1**	-	86275	5.314	15541	6380	18595	0.343	-1.543238	0.363	0
chr8	22497883	22499722	**FLJ14107**	-	1839	2.872	176	72	211	0.345	-1.536611	0.34	0
chr5	148521053	148639999	**ABLIM3**	+	118946	1.783	7192	2973	8598	0.346	-1.532132	0.247	0
chr6	129898239	130031370	**ARHGAP18**	-	133131	3.744	17507	7251	20926	0.347	-1.528985	0.42	0
chr3	88188261	88193814	**ZNF654**	+	5553	1.902	369	153	441	0.347	-1.525931	0.292	0
chr5	140868807	140871444	**PCDHGC5**	+	2637	1.755	153	63	182	0.349	-1.517288	0.358	0
chr7	127292201	127732659	**SND1**	+	440458	2.549	38433	16081	45883	0.35	-1.512583	0.586	0
chr1	26210676	26233368	**STMN1**	-	22692	3.538	2738	1145	3269	0.35	-1.513147	0.191	0
chr10	60272903	60588845	**BICC1**	+	315942	2.65	28774	12048	34350	0.351	-1.511463	0.519	0
chr2	66662531	66799891	**MEIS1**	+	137360	7.02	33725	14147	40251	0.351	-1.508527	0.573	0
chr6	31126302	31131992	**TCF19**	+	5690	1.201	230	96	274	0.352	-1.505609	0.324	0
chr19	56088890	56092211	**ZNF579**	-	3321	2.455	264	111	316	0.352	-1.506955	0.278	0
chr4	114372187	114683083	**CAMK2D**	-	310896	3.378	36303	15272	43314	0.353	-1.503907	0.584	0
chr16	88875876	88878342	**APRT**	-	2466	1.462	118	50	141	0.354	-1.49949	0.391	0
chr11	35639734	35642421	**FJX1**	+	2687	1.021	90	38	108	0.354	-1.496297	0.435	0
chr7	116312458	116438440	**MET**	+	125982	2.886	12478	5273	14880	0.354	-1.496622	0.355	0
chr1	164528596	164821060	**PBX1**	+	292464	1.002	9992	4218	11916	0.354	-1.49803	0.312	0
chr19	10244021	10305755	**DNMT1**	-	61734	2.079	4311	1828	5139	0.356	-1.49086	0.208	0
chr22	29702996	29708774	**GAS2L1**	+	5778	1.154	221	93	264	0.356	-1.490808	0.325	0
chr6	27775976	27776445	**HIST1H2AI**	+	469	1.439	23	10	28	0.356	-1.488421	0.664	0
chr5	43065288	43067073	**LOC100132356**	-	1785	4.858	285	121	340	0.356	-1.490364	0.289	0
chr1	10535002	10690815	**PEX14**	+	155813	1.332	7051	2989	8405	0.356	-1.491435	0.266	0
chr19	2233154	2236328	**PLEKHJ1**	-	3174	2.052	217	92	258	0.356	-1.489634	0.331	0
chr2	10262694	10271546	**RRM2**	+	8852	3.821	1161	492	1383	0.356	-1.490646	0.2	0
chr10	134351352	134596984	**INPP5A**	+	245632	1.013	8427	3586	10041	0.357	-1.485567	0.288	0
chr10	21823100	22032559	**MLLT10**	+	209459	1.938	13782	5858	16424	0.357	-1.487176	0.37	0
chr7	31792631	32338383	**PDE1C**	-	545752	2.916	55433	23559	66058	0.357	-1.487402	0.702	0
chr13	110959630	111165373	**COL4A2**	+	205743	2.045	14410	6138	17167	0.358	-1.483713	0.382	0
chr3	151985828	152183569	**MBNL1**	+	197741	8.925	61653	26322	73430	0.358	-1.480084	0.733	0
chr3	27414213	27498245	**SLC4A7**	-	84032	6.631	19574	8344	23317	0.358	-1.482472	0.464	0
chr6	27834569	27835359	**HIST1H1B**	-	790	7.315	200	85	239	0.359	-1.479712	0.381	0
chr22	35796115	35820495	**MCM5**	+	24380	1.131	926	395	1103	0.359	-1.479151	0.198	0
chr6	3259161	3268300	**PSMG4**	+	9139	1.493	452	193	538	0.359	-1.478615	0.245	0
chr4	142949181	143767604	**INPP4B**	-	818423	2.315	64053	27429	76261	0.36	-1.475218	0.732	0
chr13	97874573	98046374	**MBNL2**	+	171801	6.045	36916	15874	43930	0.361	-1.468538	0.622	0
chr8	120428551	120436678	**NOV**	+	8127	1.283	341	146	406	0.361	-1.470106	0.272	0
chr12	53894704	53900215	**TARBP2**	+	5511	1.015	184	79	219	0.361	-1.471245	0.354	0
chr11	46402617	46405375	**MDK**	+	2758	1.278	116	50	138	0.363	-1.463352	0.41	0
chr8	131064350	131455906	**ASAP1**	-	391556	6.366	85237	36886	101354	0.364	-1.458265	0.844	0
chr11	2423522	2425106	**TSSC4**	+	1584	2.566	134	58	160	0.364	-1.456438	0.398	0
chr9	89559276	89562104	**GAS1**	-	2828	5.925	543	236	646	0.365	-1.452257	0.219	0
chr19	1103935	1106787	**GPX4**	+	2852	2.182	205	89	244	0.365	-1.453144	0.352	0
chr17	39182278	39183454	**KRTAP1-5**	-	1176	3.431	130	56	154	0.365	-1.453345	0.381	0
chr5	119800018	120022964	**PRR16**	+	222946	2.575	19744	8569	23470	0.365	-1.453588	0.461	0
chr7	84624871	84751247	**SEMA3D**	-	126376	1.686	7417	3215	8817	0.365	-1.455254	0.31	0
chr5	304290	438405	**AHRR**	+	134115	4.248	19150	8337	22755	0.366	-1.448503	0.443	0
chr22	50941375	50946135	**LMF2**	-	4760	1.301	205	89	243	0.366	-1.448809	0.354	0
chr16	86544132	86548070	**FOXF1**	+	3938	6.035	770	335	915	0.367	-1.447914	0.197	0
chr7	27870192	28220437	**JAZF1**	-	350245	2.376	28811	12547	34232	0.367	-1.447978	0.552	0
chr4	40812043	41216635	**APBB2**	-	404592	1.612	22353	9768	26547	0.368	-1.442385	0.488	0
chr17	17584786	17714765	**RAI1**	+	129979	1.968	8530	3724	10132	0.368	-1.443884	0.296	0
chr1	109822175	109825790	**PSRC1**	-	3615	1.623	199	87	237	0.369	-1.437367	0.385	0
chr4	99391517	99579812	**TSPAN5**	-	188295	4.34	27931	12250	33158	0.369	-1.436604	0.54	0
chr16	86612114	86615304	**FOXL1**	+	3190	3.583	375	165	445	0.37	-1.432787	0.279	0
chr3	129033613	129035120	**H1FX**	-	1507	6.862	336	147	399	0.37	-1.435794	0.286	0
chr2	70314584	70316334	**PCBP1**	+	1750	15.332	891	391	1058	0.37	-1.436036	0.208	0
chr19	2425621	2427875	**TIMM13**	-	2254	2.024	149	65	177	0.37	-1.434096	0.388	0
chr4	128802015	128820377	**PLK4**	+	18362	1.148	722	318	857	0.371	-1.430105	0.25	0
chr17	1963132	2207069	**SMG6**	-	243937	1.476	12206	5368	14486	0.371	-1.432219	0.375	0
chr9	140083053	140084822	**SSNA1**	+	1769	1.484	86	38	103	0.371	-1.429243	0.468	0
chr8	145648999	145653927	**VPS28**	-	4928	1.207	196	86	233	0.371	-1.430665	0.366	0

chr9	140098534	140100090	**TMEM203**	-	1556	2.198	113	50	134	0.372	-1.42707	0.446	0
chr10	69869249	69971773	**MYPN**	+	102524	1.809	6393	2826	7582	0.373	-1.423725	0.299	0
chr22	41641614	41682216	**RANGAP1**	-	40602	2.916	3978	1759	4717	0.373	-1.42313	0.234	0
chr6	45296053	45518819	**RUNX2**	+	222766	1.789	13684	6058	16226	0.373	-1.42135	0.404	0
chr16	87863628	87903100	**SLC7A5**	-	39472	5.194	6747	2983	8002	0.373	-1.423532	0.266	0
chr14	75745480	75748937	**FOS**	+	3457	53.708	6072	2694	7197	0.374	-1.417303	0.252	0
chr14	65877309	66210839	**FUT8**	+	333530	1.393	16100	7138	19087	0.374	-1.41899	0.449	0
chr4	120980578	120988013	**MAD2L1**	-	7435	1.534	384	170	456	0.374	-1.419083	0.306	0
chr16	89334034	89556969	**ANKRD11**	-	222935	2.805	21043	9350	24940	0.375	-1.415334	0.473	0
chr15	32907690	32931868	**ARHGAP11A**	+	24178	1.412	1174	522	1391	0.375	-1.413323	0.23	0
chr6	27114907	27115346	**HIST1H2AH**	+	439	2.349	34	15	41	0.375	-1.414447	0.611	0
chr2	74699084	74699942	**MRPL53**	-	858	3.994	112	50	133	0.375	-1.413386	0.437	0
chr20	62571181	62587800	**UCKL1**	-	16619	1.229	672	299	796	0.375	-1.413571	0.229	0
chr17	7306292	7307450	**C17orf61**	-	1158	1.108	42	19	50	0.376	-1.410595	0.585	0
chr4	48499379	48782316	**FRYL**	-	282937	1.495	14708	6555	17426	0.376	-1.410511	0.44	0
chr1	149859018	149859466	**HIST2H2AB**	-	448	1.225	18	8	21	0.376	-1.410775	0.692	0
chr1	227918889	227923112	**JMJD4**	-	4223	1.611	223	99	264	0.376	-1.412006	0.354	0
chr11	67798083	67804114	**NDUFS8**	+	6031	1.552	313	139	371	0.376	-1.411376	0.327	0
chr16	64980682	65155919	**CDH11**	-	175237	4.183	25274	11274	29941	0.377	-1.409106	0.537	0
chr7	92234234	92465941	**CDK6**	-	231707	4.539	36515	16376	43228	0.379	-1.400336	0.63	0
chr10	126676417	126849624	**CTBP2**	-	173207	2.65	15478	6943	18323	0.379	-1.400039	0.426	0
chr17	13399005	13505244	**HS3ST3A1**	-	106239	1.641	5993	2688	7094	0.379	-1.399845	0.302	0
chr9	116638561	116818875	**ZNF618**	+	180314	1.533	9372	4206	11094	0.379	-1.399238	0.35	0
chrX	134184962	134186221	**FAM127B**	-	1259	1.614	66	30	79	0.38	-1.394492	0.51	0
chr6	17759413	17987854	**KIF13A**	-	228441	2.844	22403	10078	26511	0.38	-1.395319	0.519	0
chr3	136055998	136471245	**STAG1**	-	415247	1.657	23839	10716	28214	0.38	-1.396653	0.535	0
chr16	31102174	31106276	**VKORC1**	-	4102	1.472	200	90	237	0.38	-1.395677	0.385	0
chr22	18270415	18507325	**MICAL3**	-	236910	1.381	10973	4943	12983	0.381	-1.392972	0.366	0
chr11	48002109	48192394	**PTPRJ**	+	190285	1.281	8422	3798	9964	0.381	-1.391307	0.355	0
chr6	74405507	74538041	**CD109**	+	132534	1.428	6401	2889	7572	0.382	-1.390025	0.299	0
chr6	33384318	33386065	**CUTA**	-	1747	6.806	395	178	467	0.382	-1.387341	0.307	0
chr13	76194569	76434006	**LMO7**	+	239437	11.601	97254	43896	115040	0.382	-1.389954	0.926	0
chr15	37183221	37393500	**MEIS2**	-	210279	4.023	29012	13107	34314	0.382	-1.388423	0.576	0
chr22	46067677	46241187	**ATXN10**	+	173510	5.252	31166	14161	36835	0.384	-1.379095	0.591	0
chr1	36787631	36789755	**FAM176B**	-	2124	1.861	128	58	151	0.384	-1.380157	0.428	0
chr3	47892179	48130769	**MAP4**	-	238590	2.788	22977	10453	27151	0.385	-1.377076	0.534	0
chr5	16662015	16936385	**MYO10**	-	274370	3.328	31404	14271	37115	0.385	-1.378927	0.596	0
chr15	75966662	76005189	**CSPG4**	-	38527	1.759	2244	1024	2651	0.386	-1.372308	0.217	0
chr17	76352858	76356158	**SOCS3**	-	3300	24.274	2605	1188	3077	0.386	-1.372736	0.208	0
chr9	140513443	140730578	**EHMT1**	+	217135	1.354	9875	4518	11661	0.387	-1.367968	0.365	0
chr8	89049459	89339717	**MMP16**	-	290258	1.089	10962	5006	12948	0.387	-1.370762	0.403	0
chr1	249104650	249120154	**SH3BP5L**	-	15504	3.294	1711	782	2021	0.387	-1.369362	0.225	0
chr1	103342022	103574052	**COL11A1**	-	232030	6.438	53240	24385	62858	0.388	-1.366103	0.751	0
chr2	172964165	172967478	**DLX2**	-	3313	5.882	639	292	754	0.388	-1.366622	0.254	0
chr11	28042162	28129746	**KIF18A**	-	87584	1.85	5631	2581	6647	0.388	-1.364457	0.324	0
chr6	20100934	20212670	**MBOAT1**	-	111736	1.911	7560	3462	8926	0.388	-1.36615	0.378	0
chr3	49057907	49060926	**NDUFAF3**	+	3019	2.479	246	113	291	0.388	-1.364878	0.368	0
chr6	157099063	157531913	**ARID1B**	+	432850	1.741	26047	11970	30739	0.389	-1.36066	0.572	0
chr4	185308875	185395726	**IRF2**	-	86851	1.005	2964	1362	3498	0.389	-1.360564	0.259	0
chr22	44888449	44894005	**LDOC1L**	-	5556	3.399	625	287	738	0.389	-1.361461	0.267	0
chr3	149086804	149095568	**TM4SF1**	-	8764	2.742	810	372	956	0.389	-1.361928	0.26	0
chr19	18367905	18385319	**KIAA1683**	-	17414	1.055	615	283	726	0.39	-1.356982	0.28	0
chr19	3359560	3469215	**NFIC**	+	109655	1.747	6403	2945	7555	0.39	-1.358941	0.305	0
chr9	131707808	131710012	**DOLK**	-	2204	1.925	140	64	165	0.391	-1.356254	0.445	0
chr1	41154751	41157933	**LOC100130557**	-	3182	1.643	177	82	209	0.391	-1.352951	0.442	0
chr16	67182004	67184902	**B3GNT9**	-	2898	2.936	282	130	332	0.392	-1.352188	0.366	0
chr13	98795433	99102023	**FARP1**	+	306590	1.751	18408	8518	21705	0.392	-1.349471	0.494	0
chr18	55711609	56068772	**NEDD4L**	+	357163	1.477	17620	8148	20778	0.392	-1.350411	0.464	0
chr2	122095351	122407052	**CLASP1**	-	311701	1.883	20135	9338	23733	0.393	-1.345611	0.517	0
chr19	5785152	5791249	**DUS3L**	-	6097	1.988	399	185	470	0.393	-1.345713	0.318	0
chr9	123617930	123639606	**PHF19**	-	21676	1.39	1012	468	1193	0.393	-1.348022	0.248	0
chr2	54683453	54898583	**SPTBN1**	+	215130	2.332	17115	7935	20175	0.393	-1.346217	0.479	0
chr17	76000317	76104916	**TNRC6C**	+	104599	1.033	3694	1711	4355	0.393	-1.347354	0.283	0
chr7	132469622	132766828	**CHCHD3**	-	297206	1.06	10679	4957	12586	0.394	-1.344168	0.393	0
chr5	176560079	176727214	**NSD1**	+	167135	1.464	8409	3900	9911	0.394	-1.345448	0.375	0
chr18	25530929	25757445	**CDH2**	-	226516	5.324	43007	20033	50666	0.395	-1.338636	0.713	0
chr16	46723557	46732306	**ORC6**	+	8749	1.178	350	163	412	0.395	-1.338806	0.37	0
chr20	42543491	42698254	**TOX2**	+	154763	1.136	5936	2764	6993	0.395	-1.339082	0.316	0
chr2	238232654	238322850	**COL6A3**	-	90196	9.885	29603	13818	34864	0.396	-1.335149	0.578	0
chr15	64457715	64648442	**CSNK1G1**	-	190727	1.069	7000	3264	8245	0.396	-1.336994	0.354	0
chr3	56654159	56717135	**FAM208A**	-	62976	1.392	3027	1412	3566	0.396	-1.335996	0.288	0

chrX	122318095	122624766	**GRIA3**	+	306671	1.221	12851	6024	15127	0.398	-1.328168	0.448	0
chr6	27805657	27806117	**HIST1H2AK**	-	460	1.758	27	12	32	0.398	-1.329033	0.674	0
chr11	1968501	1977839	**MRPL23**	+	9338	1.001	307	144	362	0.398	-1.330673	0.358	0
chr8	141668480	142011412	**PTK2**	-	342932	2.249	26553	12471	31247	0.399	-1.325103	0.587	0
chr22	21982377	21984340	**YDJC**	-	1963	1.716	110	51	130	0.399	-1.323887	0.481	0
chr12	89981825	90049844	**ATP2B1**	-	68019	2.741	6243	2944	7343	0.401	-1.318196	0.324	0
chr5	159848864	159855746	**PTTG1**	+	6882	2.261	536	252	630	0.401	-1.319475	0.344	0
chr19	17622431	17632097	**PGLS**	+	9666	1.43	457	216	538	0.402	-1.315962	0.321	0
chr15	70946892	71055850	**UACA**	-	108958	4.776	17913	8464	21063	0.402	-1.315293	0.512	0
chr17	79993756	79995573	**DCXR**	-	1817	1.184	71	33	83	0.403	-1.310763	0.55	0
chr2	218664511	218808796	**TNS1**	-	144285	1.822	8990	4255	10568	0.403	-1.312404	0.393	0
chr2	189157389	189460652	**GULP1**	+	303263	2.889	30974	14719	36392	0.404	-1.305871	0.644	0
chr3	67410883	67705038	**SUCLG2**	-	294155	1.787	17844	8479	20966	0.404	-1.305955	0.502	0
chr4	54851665	54853449	**RPL21P44**	-	1784	1.193	72	34	85	0.405	-1.30368	0.582	0
chr1	21132784	21503381	**EIF4G3**	-	370597	1.516	19390	9244	22772	0.406	-1.300703	0.537	0
chr6	27100094	27100575	**HIST1H2BJ**	-	481	2.205	36	17	42	0.406	-1.299493	0.674	0
chr1	85784167	86044046	**DDAH1**	-	259879	8.089	73119	34905	85857	0.407	-1.298493	0.852	0
chr10	135122422	135126666	**ZNF511**	+	4244	1.248	177	84	207	0.408	-1.292816	0.451	0
chr13	95672082	95953687	**ABCC4**	-	281605	1.135	10754	5161	12618	0.409	-1.289832	0.417	0
chr6	27777841	27778314	**HIST1H3H**	+	473	3.074	51	24	60	0.409	-1.289309	0.643	0
chr1	1288070	1293915	**MXRA8**	-	5845	4.522	866	415	1017	0.409	-1.290111	0.266	0
chr3	105085556	105295757	**ALCAM**	+	210201	3.955	29284	14097	34346	0.41	-1.28469	0.639	0
chr17	16946073	17095962	**MPRIP**	+	149889	2.389	12039	5789	14122	0.41	-1.286532	0.438	0
chr16	31085742	31094833	**ZNF646**	+	9091	1.148	346	166	407	0.41	-1.286143	0.376	0
chr6	26273203	26273640	**HIST1H2BI**	+	437	2.258	34	16	39	0.411	-1.282302	0.674	0
chr17	41166621	41174459	**VAT1**	-	7838	5.745	1480	714	1736	0.411	-1.282063	0.254	0
chr10	31608100	31818742	**ZEB1**	+	210642	2.206	16017	7716	18784	0.411	-1.283549	0.508	0
chr11	66081957	66084515	**CD248**	-	2558	14.111	1195	577	1401	0.412	-1.27918	0.265	0
chr9	134735498	134955253	**MED27**	-	219755	1.241	9232	4458	10823	0.412	-1.279417	0.403	0
chr9	134305476	134375575	**PRRC2B**	+	70099	2.378	5637	2721	6609	0.412	-1.280068	0.348	0
chr13	53029494	53050763	**CKAP2**	+	21269	2.837	2075	1006	2432	0.414	-1.272471	0.305	0
chr17	73126319	73127890	**NT5C**	-	1571	3.145	161	78	188	0.415	-1.268858	0.455	0
chr20	48807119	48809227	**CEBPB**	+	2108	5.134	348	169	407	0.416	-1.265046	0.357	0
chr8	62413114	62627199	**ASPH**	-	214085	7.951	58609	28594	68614	0.417	-1.262783	0.796	0
chr8	23154409	23261722	**LOXL2**	-	107313	6.379	23151	11300	27102	0.417	-1.262002	0.575	0
chr6	129204285	129837710	**LAMA2**	+	633425	1.667	36852	18041	43122	0.418	-1.257134	0.693	0
chr15	48700502	48937985	**FBN1**	-	237483	5.206	43307	21242	50662	0.419	-1.253969	0.732	0
chr15	59397283	59417244	**CCNB2**	+	19961	1.295	873	429	1021	0.42	-1.249894	0.316	0
chr3	196769430	197025447	**DLG1**	-	256017	2.174	19319	9482	22598	0.42	-1.252956	0.569	0
chr5	172261222	172379688	**ERGIC1**	+	118466	1.605	6431	3158	7523	0.42	-1.252212	0.376	0
chr17	79849598	79858363	**ANAPC11**	+	8765	1.79	527	259	617	0.421	-1.247756	0.361	0
chr4	1205227	1242908	**CTBP1**	-	37681	1.853	2303	1134	2693	0.421	-1.247517	0.279	0
chr20	814355	826922	**FAM110A**	+	12567	1.162	487	239	569	0.421	-1.248563	0.363	0
chr19	47567446	47617009	**ZC3H4**	-	49563	1.47	2450	1206	2864	0.421	-1.247409	0.302	0
chr19	4675243	4723855	**DPP9**	-	48612	1.748	2828	1396	3306	0.422	-1.24366	0.298	0
chr16	21529229	21531765	**SLC7A5P2**	-	2536	1.904	159	78	185	0.422	-1.244976	0.48	0
chr19	1576677	1592652	**MBD3**	-	15975	1.074	570	281	666	0.423	-1.240996	0.346	0
chr2	15307031	15701454	**NBAS**	-	394423	1.005	13600	6717	15894	0.423	-1.242463	0.498	0
chr16	58028572	58033762	**ZNF319**	-	5190	1.223	210	103	245	0.423	-1.239973	0.452	0
chr1	17866329	18024370	**ARHGEF10L**	+	158041	1.04	5480	2714	6402	0.424	-1.238199	0.354	0
chr16	88709696	88717457	**CYBA**	-	7761	1.041	265	131	309	0.424	-1.237821	0.42	0
chr17	66031847	66042970	**KPNA2**	+	11123	5.474	2073	1026	2422	0.424	-1.23802	0.315	0
chr11	65485143	65488409	**RNASEH2C**	-	3266	1.108	119	59	139	0.424	-1.239345	0.521	0
chr6	130758261	130764210	**TMEM200A**	+	5949	1.157	228	113	266	0.424	-1.239351	0.449	0
chr9	112810877	112934791	**AKAP2**	+	123914	6.583	27501	13639	32122	0.425	-1.235743	0.615	0
chr11	308106	309410	**IFITM2**	+	1304	5.666	246	122	287	0.425	-1.233899	0.448	0
chr8	143738873	143751401	**JRK**	-	12528	2.964	1227	609	1433	0.425	-1.234793	0.286	0
chr17	36886509	36891858	**CISD3**	+	5349	1.137	203	101	237	0.426	-1.231291	0.471	0
chr11	72547789	72853143	**FCHSD2**	-	305354	1.946	20750	10313	24228	0.426	-1.232144	0.595	0
chr22	20861885	20941919	**MED15**	+	80034	4.537	12201	6073	14244	0.426	-1.229871	0.467	0
chr20	278203	280963	**ZCCHC3**	+	2760	6.168	563	279	657	0.426	-1.231466	0.348	0
chr4	7760439	7941653	**AFAP1**	-	181214	2.75	16869	8404	19691	0.427	-1.228371	0.531	0
chr19	5891286	5904024	**NDUFA11**	-	12738	1.353	570	284	666	0.427	-1.227213	0.35	0
chr2	202671197	202758263	**CDK15**	+	87066	1.869	5575	2785	6506	0.428	-1.223976	0.388	0
chr3	10327437	10335133	**GHRLOS**	+	7696	1.055	270	134	315	0.428	-1.224968	0.44	0
chr10	3109711	3178997	**PFKP**	+	69286	1.344	3134	1564	3658	0.428	-1.225834	0.324	0
chr17	3572089	3572962	**TMEM93**	+	873	2.77	80	40	93	0.428	-1.224377	0.581	0
chr15	85923870	86292586	**AKAP13**	+	368716	3.125	39564	19816	46146	0.429	-1.219554	0.716	0
chr15	40674921	40686489	**C15orf23**	+	11568	1.487	589	294	687	0.429	-1.222058	0.381	0
chr9	97488950	97849500	**C9orf3**	+	360550	2.007	24686	12344	28800	0.429	-1.222286	0.611	0
chr1	197473878	197744623	**DENND1B**	-	270745	1.252	11845	5932	13816	0.429	-1.219627	0.507	0

chr19	4791727	4795571	**FEM1A**	+	3844	1.625	209	104	244	0.429	-1.221958	0.475	0
chr7	100026412	100031749	**MEPCE**	+	5337	3.205	564	282	658	0.429	-1.220372	0.351	0
chr5	148206155	148208197	**ADRB2**	+	2042	1.537	103	51	121	0.43	-1.218905	0.544	0
chr9	132396882	132404448	**ASB6**	-	7566	1.75	438	219	511	0.43	-1.217297	0.379	0
chr5	102594441	102614361	**C5orf30**	+	19920	14.805	10384	5204	12110	0.43	-1.218384	0.496	0
chr12	78225068	78606790	**NAV3**	+	381722	3.424	46226	23158	53915	0.43	-1.219151	0.771	0
chr3	176738541	176915048	**TBL1XR1**	-	176507	2.447	14905	7469	17383	0.43	-1.218683	0.534	0
chr19	13945329	13947100	**LOC284454**	-	1771	37.52	2180	1096	2542	0.431	-1.213697	0.293	0
chr9	140446308	140447007	**MRPL41**	+	699	3.576	81	41	95	0.431	-1.213776	0.564	0
chr17	29421944	29704695	**NF1**	+	282751	1.67	16325	8203	19032	0.431	-1.214155	0.552	0
chr17	35441926	35766902	**ACACA**	-	324976	1.084	11836	5954	13796	0.432	-1.212277	0.47	0
chr9	134452156	134612925	**RAPGEF1**	-	160769	1.303	7017	3530	8179	0.432	-1.212199	0.396	0
chr9	100961279	101018003	**TBC1D2**	-	56724	1.857	3513	1769	4095	0.432	-1.210868	0.332	0
chr12	98909350	98944157	**TMPO**	+	34807	2.115	2512	1264	2928	0.432	-1.212362	0.341	0
chr2	62900985	63273621	**EHBP1**	+	372636	2.134	28280	14259	32953	0.433	-1.208557	0.674	0
chr2	197063976	197457335	**HECW2**	-	393359	1.479	19884	10039	23166	0.433	-1.206345	0.583	0
chr19	42460832	42463528	**RABAC1**	-	2696	3.489	310	156	362	0.433	-1.206695	0.427	0
chr1	178062863	178448648	**RASAL2**	+	385785	1.89	25055	12644	29192	0.433	-1.207019	0.627	0
chr5	98104998	98132198	**RGMB**	+	27200	26.253	24232	12222	28236	0.433	-1.20799	0.611	0
chr15	57210832	57580714	**TCF12**	+	369882	2.274	29265	14761	34100	0.433	-1.207918	0.671	0
chr1	44440601	44443972	**ATP6V0B**	+	3371	3.569	395	199	460	0.434	-1.205714	0.389	0
chr19	55996556	55998935	**NAT14**	+	2379	1.069	82	41	96	0.434	-1.202836	0.567	0
chr11	122943032	123066007	**CLMP**	-	122975	2.621	10852	5497	12636	0.435	-1.200778	0.467	0
chr8	145064225	145067583	**GRINA**	+	3358	3.943	435	220	507	0.435	-1.200095	0.385	0
chr17	76967334	76976061	**LGALS3BP**	-	8727	3.053	878	445	1023	0.435	-1.201076	0.318	0
chr9	118916070	119164600	**PAPPA**	+	248530	1.18	10080	5109	11737	0.435	-1.199717	0.48	0
chr17	78234666	78370086	**RNF213**	+	135420	1.613	7377	3740	8590	0.435	-1.199585	0.42	0
chr3	49758931	49761384	**GMPPB**	-	2453	1.579	129	65	150	0.436	-1.197996	0.537	0
chr14	61201458	61435398	**MNAT1**	+	233940	1.317	10596	5373	12337	0.436	-1.199094	0.488	0
chr19	45596430	45650543	**PPP1R37**	+	54113	1.527	2725	1383	3173	0.436	-1.197614	0.314	0
chr19	42724491	42732353	**ZNF526**	+	7862	1.258	329	166	383	0.436	-1.198993	0.434	0
chr11	58294343	58345639	**LPXN**	-	51296	4.324	7505	3815	8735	0.437	-1.194862	0.43	0
chr9	80335190	80646219	**GNAQ**	-	311029	1.736	18558	9464	21590	0.438	-1.189742	0.584	0
chr2	206547223	206662857	**NRP2**	+	115634	1.015	4055	2068	4718	0.438	-1.189794	0.389	0
chr17	60556385	60692841	**TLK2**	+	136456	1.47	6924	3528	8056	0.438	-1.191052	0.446	0
chr6	37787306	38122399	**ZFAND3**	+	335093	2.833	32680	16661	38020	0.438	-1.190275	0.683	0
chr1	109419602	109473044	**GPSM2**	+	53442	1.169	2141	1092	2491	0.439	-1.189017	0.354	0
chr4	177604690	177713895	**VEGFC**	-	109205	11.845	44010	22452	51195	0.439	-1.189141	0.741	0
chr19	39897486	39900045	**ZFP36**	+	2559	47.037	3902	1990	4540	0.439	-1.189309	0.321	0
chr16	75327607	75467387	**CFDP1**	-	139780	1.072	5093	2604	5923	0.44	-1.185342	0.394	0
chr12	100967488	101018685	**GAS2L3**	+	51197	1.754	3028	1548	3521	0.44	-1.185347	0.353	0
chr17	40687950	40696466	**NAGLU**	+	8516	2.624	734	376	853	0.441	-1.180657	0.345	0
chr19	41725107	41767671	**AXL**	+	42564	6.675	9623	4946	11182	0.442	-1.176709	0.47	0
chr11	43702142	43878169	**HSD17B12**	+	176027	1.034	6206	3194	7210	0.443	-1.174367	0.427	0
chr4	41362803	41702061	**LIMCH1**	+	339258	1.472	17754	9135	20627	0.443	-1.175027	0.614	0
chr22	43265771	43411184	**PACSIN2**	-	145413	1.437	7070	3642	8213	0.443	-1.173083	0.435	0
chr1	230202955	230417875	**GALNT2**	+	214920	1.687	12273	6326	14256	0.444	-1.172098	0.507	0
chr11	4115923	4160106	**RRM1**	+	44183	1.145	1738	899	2018	0.445	-1.166717	0.369	0
chr16	2255177	2259418	**MLST8**	+	4241	1.572	220	114	255	0.446	-1.165938	0.488	0
chr10	105206542	105212162	**CALHM2**	-	5620	2.921	550	285	638	0.447	-1.161383	0.399	0
chr19	36031639	36036931	**LOC100506469**	-	5292	1.329	231	120	269	0.447	-1.160579	0.481	0
chr12	96051582	96184536	**NTN4**	-	132954	3.336	15456	8014	17936	0.447	-1.1622	0.582	0
chr1	41492870	41707815	**SCMH1**	-	214945	1.104	8157	4228	9467	0.447	-1.162917	0.471	0
chr8	99466860	99837909	**STK3**	-	371049	1.05	13442	6970	15600	0.447	-1.162242	0.549	0
chr3	15708743	15901053	**ANKRD28**	-	192310	3.818	25271	13142	29314	0.448	-1.157444	0.652	0
chr19	51300960	51307974	**C19orf48**	-	7014	2.992	698	362	810	0.448	-1.159721	0.375	0
chr11	65543377	65547822	**DKFZp761E198**	-	4445	3.091	451	234	523	0.448	-1.159383	0.4	0
chr8	145137523	145141119	**GPAA1**	+	3596	1.58	187	97	217	0.448	-1.158727	0.508	0
chr16	29817854	29822504	**MAZ**	+	4650	1.643	256	133	296	0.448	-1.15723	0.488	0
chr11	64085559	64089295	**PRDX5**	+	3736	1.803	222	115	258	0.448	-1.157218	0.488	0
chr3	30647993	30735633	**TGFBR2**	+	87640	3.257	9764	5078	11326	0.448	-1.157065	0.493	0
chr19	36036501	36038429	**TMEM147**	+	1928	1.808	115	60	134	0.448	-1.158521	0.569	0
chr19	56154985	56156989	**ZNF581**	+	2004	1.325	87	45	101	0.448	-1.158238	0.591	0
chr19	2100992	2151556	**AP3D1**	-	50564	1.632	2717	1417	3150	0.45	-1.152533	0.341	0
chrX	153672472	153679002	**FAM50A**	+	6530	1.089	237	124	275	0.45	-1.15296	0.496	0
chr9	136207754	136214972	**MED22**	-	7218	1.435	342	178	396	0.45	-1.150404	0.449	0
chr17	18218593	18231370	**SMCR8**	+	12777	2.381	1006	525	1166	0.45	-1.151067	0.345	0
chr7	97910978	97922275	**BRI3**	+	11297	9.954	3693	1930	4281	0.451	-1.148914	0.357	0
chr16	87439851	87525460	**ZCCHC14**	-	85609	1.021	2894	1513	3355	0.451	-1.149047	0.353	0
chr10	75183336	75193319	**ZMYND17**	-	9983	1.033	353	185	410	0.451	-1.147675	0.484	0
chr8	145149959	145152428	**CYC1**	+	2469	1.728	141	73	163	0.452	-1.145413	0.546	0

chr6	148663728	148873184	**SASH1**	+	209456	2.447	17412	9121	20176	0.452	-1.145246	0.584	0
chr4	5712923	5816031	**EVC**	+	103108	2.253	7809	4095	9047	0.453	-1.143351	0.452	0
chrX	38660684	38665783	**MID1IP1**	+	5099	2.277	380	199	441	0.453	-1.142952	0.433	0
chr4	141786724	142054616	**RNF150**	-	267892	1.927	18446	9687	21365	0.453	-1.141134	0.64	0
chr1	1246964	1260046	**CPSF3L**	-	13082	1.284	555	291	642	0.454	-1.139002	0.397	0
chr12	11802787	12048325	**ETV6**	+	245538	2.028	17178	9038	19891	0.454	-1.137951	0.6	0
chr17	18874380	18908060	**FAM83G**	-	33680	2.434	2720	1430	3150	0.454	-1.139414	0.357	0
chr12	6643656	6647536	**GAPDH**	+	3880	19.376	2486	1305	2879	0.454	-1.14061	0.35	0
chr8	143781528	143785584	**LY6K**	+	4056	1.715	228	120	265	0.454	-1.138389	0.49	0
chr17	79825596	79829282	**ARHGDIA**	-	3686	14.234	1734	912	2008	0.455	-1.137463	0.343	0
chr14	73957643	73960105	**C14orf169**	+	2462	1.838	148	78	172	0.455	-1.136402	0.54	0
chr6	4706392	4955778	**CDYL**	+	249386	1.037	8807	4635	10197	0.455	-1.137381	0.488	0
chr1	6650783	6662929	**KLHL21**	-	12146	4.748	1888	995	2186	0.455	-1.134522	0.333	0
chr20	62371210	62375403	**SLC2A4RG**	+	4193	1.263	173	91	200	0.455	-1.137128	0.52	0
chr15	69706626	69740764	**KIF23**	+	34138	3.159	3693	1948	4275	0.456	-1.134044	0.408	0
chr10	27443752	27475848	**MASTL**	+	32096	1.05	1144	604	1324	0.456	-1.131602	0.379	0
chr17	29302352	29303017	**DPRXP4**	+	665	1.673	37	20	43	0.457	-1.128675	0.726	0
chr16	84509965	84538288	**KIAA1609**	-	28323	4.955	4723	2497	5465	0.457	-1.129717	0.409	0
chr11	61560108	61564714	**FEN1**	+	4606	1.139	175	93	203	0.458	-1.126758	0.548	0
chr22	29999544	30094589	**NF2**	+	95045	1.663	5394	2859	6239	0.458	-1.125878	0.446	0
chr19	30156326	30166383	**PLEKHF1**	+	10057	1.132	372	196	430	0.458	-1.127609	0.438	0
chr10	105353783	105615164	**SH3PXD2A**	-	261381	1.72	15035	7960	17393	0.458	-1.127539	0.554	0
chr1	19665266	19812066	**CAPZB**	-	146800	4.353	21329	11318	24666	0.459	-1.123934	0.613	0
chr14	105155942	105185947	**INF2**	+	30005	1.822	1807	959	2090	0.459	-1.123257	0.353	0
chr9	34634718	34637768	**SIGMAR1**	-	3050	2.298	231	123	268	0.459	-1.122685	0.505	0
chr11	46417963	46612914	**AMBRA1**	-	194951	1.304	8666	4608	10018	0.46	-1.120391	0.495	0
chr17	65821779	65980494	**BPTF**	+	158715	1.454	7986	4249	9232	0.46	-1.119239	0.503	0
chr21	45553493	45565605	**C21orf33**	+	12112	1.526	609	324	703	0.461	-1.11656	0.397	0
chr8	145582224	145584946	**GPR172A**	+	2722	1.785	159	84	183	0.461	-1.11727	0.542	0
chr11	71713910	71791573	**NUMA1**	-	77663	2.114	5601	2984	6473	0.461	-1.116781	0.449	0
chr12	27677044	27848497	**PPFIBP1**	+	171453	2.559	15244	8118	17619	0.461	-1.117895	0.599	0
chr14	105219469	105225996	**SIVA1**	+	6527	2.517	545	290	630	0.461	-1.11596	0.42	0
chr12	109176465	109251359	**SSH1**	-	74894	8.646	21787	11616	25178	0.461	-1.116038	0.628	0
chr11	67806461	67818366	**TCIRG1**	+	11905	1.855	725	386	839	0.461	-1.117589	0.379	0
chr17	47074773	47133507	**IGF2BP1**	+	58734	1.691	3384	1806	3910	0.462	-1.113937	0.409	0
chr11	67033904	67054029	**ADRBK1**	+	20125	1.273	842	450	973	0.463	-1.111564	0.375	0
chr22	43547534	43559248	**TSPO**	+	11714	3.394	1309	700	1512	0.463	-1.111106	0.355	0
chr16	30565084	30569642	**ZNF764**	-	4558	1.295	194	103	224	0.463	-1.110962	0.528	0
chr11	47430045	47438051	**SLC39A13**	+	8006	3.331	876	470	1011	0.465	-1.103467	0.375	0
chr3	11314009	11599139	**ATG7**	+	285130	1.162	11258	6059	12992	0.466	-1.100396	0.541	0
chr2	160175489	160473059	**BAZ2B**	-	297570	1.082	11186	6015	12910	0.466	-1.101796	0.565	0
chr10	101635333	101769676	**DNMBP**	-	134343	3.317	15235	8187	17584	0.466	-1.102742	0.591	0
chrX	140269930	140271310	**LDOC1**	-	1380	3.865	177	95	205	0.466	-1.100129	0.556	0
chr9	37915894	38069210	**SHB**	-	153316	2.819	14591	7850	16838	0.466	-1.100929	0.576	0
chr7	134464163	134655480	**CALD1**	+	191317	28.643	193821	104422	223621	0.467	-1.09862	0.996	0
chr17	40949651	40950704	**CCDC56**	-	1053	1.543	54	29	62	0.467	-1.098289	0.681	0
chr2	172950207	172954401	**DLX1**	+	4194	2.252	315	169	363	0.467	-1.099681	0.493	0
chr5	95220801	95297775	**ELL2**	-	76974	24.072	66089	35702	76218	0.468	-1.094122	0.923	0
chr6	26234439	26235216	**HIST1H1D**	-	777	7.79	212	114	245	0.468	-1.095843	0.595	0
chr1	47715810	47779819	**STIL**	-	64009	1.058	2319	1253	2674	0.468	-1.09403	0.411	0
chr1	32681797	32687926	**TMEM234**	-	6129	1.036	212	114	245	0.468	-1.094657	0.543	0
chr19	14063318	14072256	**DCAF15**	+	8938	1.068	315	170	363	0.469	-1.093428	0.488	0
chr6	31694816	31698039	**DDAH2**	-	3223	1.142	121	65	139	0.469	-1.091671	0.591	0
chr15	43619973	43622820	**LCMT2**	-	2847	1.928	182	98	209	0.469	-1.091505	0.551	0
chr20	306238	310867	**SOX12**	+	4629	1.644	252	136	291	0.469	-1.091184	0.519	0
chr10	26727265	26856732	**APBB1IP**	+	129467	2.309	10269	5567	11837	0.47	-1.088127	0.549	0
chr1	56960418	57045257	**PPAP2B**	-	84839	4.773	13791	7473	15897	0.47	-1.088892	0.583	0
chr4	83550689	83720010	**SCD5**	-	169321	1.085	6361	3443	7334	0.47	-1.09064	0.495	0
chr6	7107829	7252213	**RREB1**	+	144384	3.173	15410	8364	17758	0.471	-1.086261	0.586	0
chr17	79976578	79980773	**STRA13**	-	4195	1.038	144	78	166	0.471	-1.086738	0.58	0
chr22	20067754	20099400	**DGCR8**	+	31646	2.858	3019	1641	3478	0.472	-1.083358	0.398	0
chr14	24836144	24848810	**NFATC4**	+	12666	2.144	893	486	1029	0.472	-1.08291	0.386	0
chr19	12778880	12780465	**WDR83OS**	-	1585	2.532	134	72	154	0.472	-1.084164	0.596	0
chr5	65222383	65376850	**ERBB2IP**	+	154467	5.538	29556	16099	34042	0.473	-1.080297	0.727	0
chr21	45285115	45407475	**AGPAT3**	+	122360	1.149	4690	2561	5399	0.474	-1.075783	0.437	0
chr19	10828728	10942586	**DNM2**	+	113858	1.551	5910	3227	6804	0.474	-1.076115	0.457	0
chr12	93166284	93323107	**EEA1**	-	156823	1.626	8749	4772	10075	0.474	-1.078045	0.531	0
chr16	88781745	88851372	**PIEZO1**	-	69627	2.041	4691	2560	5401	0.474	-1.076792	0.423	0
chr5	76986994	77072185	**TBCA**	-	85191	2.167	6244	3409	7189	0.474	-1.07646	0.475	0
chr19	45394476	45406946	**TOMM40**	+	12470	1.297	537	293	619	0.474	-1.076491	0.449	0
chr16	447191	450754	**NME4**	+	3563	2.45	288	157	332	0.475	-1.074127	0.507	0

chr3	52027643	52029958	**RPL29**	-	2315	4.403	341	186	393	0.475	-1.072637	0.506	0
chr5	172741725	172756506	**STC2**	-	14781	17.159	8260	4512	9509	0.475	-1.075482	0.467	0
chr19	10982252	11033448	**CARM1**	+	51196	1.143	1933	1061	2224	0.477	-1.067259	0.385	0
chr13	41506054	41593508	**ELF1**	-	87454	1.902	5775	3169	6644	0.477	-1.068026	0.504	0
chr3	73431651	73674072	**PDZRN3**	-	242421	1.668	13962	7664	16061	0.477	-1.067332	0.608	0
chr21	46825096	46933634	**COL18A1**	+	108538	1.299	4752	2611	5466	0.478	-1.066004	0.451	0
chr19	3572942	3579081	**HMG20B**	+	6139	1.191	240	132	276	0.478	-1.06458	0.528	0
chr6	158402887	158520207	**SYNJ2**	+	117320	2.587	10146	5574	11670	0.478	-1.065994	0.529	0
chr2	10443039	10567743	**HPCAL1**	+	124704	1.101	4538	2497	5218	0.479	-1.062993	0.434	0
chr16	86508130	86542466	**LOC400550**	-	34336	2.25	2552	1406	2934	0.479	-1.060994	0.394	0
chr19	45682002	45685058	**BLOC1S3**	+	3056	2.185	218	120	250	0.48	-1.059193	0.537	0
chr10	88728187	88730497	**C10orf116**	+	2310	2.029	155	85	178	0.48	-1.060124	0.588	0
chr8	141541263	141645646	**EIF2C2**	-	104383	1.677	5817	3210	6685	0.48	-1.058443	0.459	0
chr20	33890368	33999945	**UQCC**	-	109577	1.227	4546	2508	5225	0.48	-1.058379	0.458	0
chr10	35535952	35860847	**CCNY**	+	324895	1.084	11944	6600	13725	0.481	-1.056277	0.575	0
chr17	42422490	42430470	**GRN**	+	7980	2.743	719	397	827	0.481	-1.056977	0.418	0
chr5	140810157	140812789	**PCDHGA12**	+	2632	1.564	135	74	155	0.481	-1.057163	0.595	0
chr22	20105023	20114704	**RANBP1**	+	9681	1.126	366	202	421	0.481	-1.057309	0.511	0
chr13	49550047	49783915	**FNDC3A**	+	233868	2.488	20179	11172	23181	0.482	-1.052963	0.674	0
chr4	773936	775636	**LOC100129917**	-	1700	2.806	159	88	183	0.482	-1.052155	0.599	0
chr15	67358194	67487533	**SMAD3**	+	129339	6.687	28634	15863	32892	0.482	-1.052034	0.69	0
chr3	48507228	48509044	**TREX1**	+	1816	1.287	77	42	88	0.483	-1.050014	0.66	0
chr7	128502856	128505903	**ATP6V1F**	+	3047	1.916	191	106	220	0.484	-1.046357	0.566	0
chr14	96722546	96731100	**BDKRB1**	+	8554	3.02	881	489	1012	0.484	-1.047668	0.449	0
chr3	81538849	81810950	**GBE1**	-	272101	2.533	23705	13182	27213	0.484	-1.045749	0.694	0
chr20	2639040	2644843	**IDH3B**	-	5803	2.165	419	233	482	0.484	-1.046228	0.496	0
chr12	51985019	52202299	**SCN8A**	+	217280	1.677	12557	6972	14419	0.484	-1.048324	0.602	0
chr7	100271362	100276792	**GNB2**	+	5430	2.716	486	270	559	0.485	-1.045334	0.468	0
chr19	13261281	13265718	**IER2**	+	4437	12.684	1823	1015	2092	0.485	-1.043654	0.378	0
chr17	7760002	7761172	**LSMD1**	-	1170	1.522	58	32	67	0.485	-1.042767	0.688	0
chr2	61414589	61697849	**USP34**	-	283260	1.659	16131	8973	18517	0.485	-1.045192	0.632	0
chr17	42148097	42153712	**G6PC3**	+	5615	1.917	357	199	410	0.486	-1.040067	0.511	0
chr14	74178485	74181128	**PNMA1**	-	2643	5.453	478	267	548	0.487	-1.037628	0.481	0
chr2	201170603	201346986	**SPATS2L**	+	176383	3.127	19046	10636	21849	0.487	-1.038559	0.674	0
chr16	2034149	2037750	**GFER**	+	3601	1.199	141	79	162	0.488	-1.03547	0.602	0
chr16	2009516	2011976	**NDUFB10**	+	2460	1.619	133	74	153	0.488	-1.034175	0.623	0
chr1	101702304	101707076	**S1PR1**	+	4772	2.905	472	264	542	0.488	-1.035509	0.516	0
chr1	26606212	26608013	**SH3BGRL3**	+	1801	14.275	854	477	979	0.488	-1.035508	0.434	0
chr15	90328125	90358072	**ANPEP**	-	29947	5.23	5188	2909	5948	0.489	-1.031736	0.463	0
chr6	17393735	17558023	**CAP2**	+	164288	2.016	11498	6449	13182	0.489	-1.031264	0.608	0
chr6	15246526	15522253	**JARID2**	+	275727	2.211	20435	11460	23427	0.489	-1.031546	0.655	0
chr3	99357453	99515158	**COL8A1**	+	157705	2.675	14741	8281	16894	0.49	-1.028552	0.646	0
chr18	43427573	43547305	**EPG5**	-	119732	2.712	11025	6187	12637	0.49	-1.030442	0.581	0
chr12	53491435	53496128	**IGFBP6**	+	4693	2.192	344	193	394	0.49	-1.028632	0.529	0
chr20	39807088	39928739	**ZHX3**	-	121651	2.675	11191	6286	12827	0.49	-1.028879	0.595	0
chr19	3506294	3536755	**FZR1**	+	30461	1.948	1956	1100	2241	0.491	-1.025756	0.406	0
chr1	156084460	156109878	**LMNA**	+	25418	19.846	16680	9379	19114	0.491	-1.027173	0.613	0
chr22	19163093	19166301	**SLC25A1**	-	3208	1.924	202	114	232	0.491	-1.026098	0.569	0
chr9	140509783	140513308	**C9orf37**	-	3525	1.107	129	72	148	0.492	-1.024015	0.624	0
chr19	1086577	1095391	**POLR2E**	-	8814	1.101	318	179	365	0.492	-1.023645	0.522	0
chr5	180650262	180662808	**TRIM41**	+	12546	1.07	446	251	511	0.492	-1.022245	0.5	0
chr5	156904311	157002783	**ADAM19**	-	98472	2.726	9171	5179	10502	0.493	-1.019924	0.571	0
chr19	2164147	2232577	**DOT1L**	+	68430	1.865	4219	2382	4832	0.493	-1.020513	0.449	0
chr22	41972889	41985871	**PMM1**	-	12982	1.918	820	463	939	0.493	-1.020165	0.436	0
chr3	154797435	154901518	**MME**	+	104083	1.778	6111	3455	6996	0.494	-1.017668	0.485	0
chr11	109964086	110042566	**ZC3H12C**	+	78480	1.843	4920	2784	5632	0.494	-1.016497	0.5	0
chr6	485137	693109	**EXOC2**	-	207972	1.021	7221	4088	8266	0.495	-1.015663	0.538	0
chr11	43918852	43921424	**LOC729799**	+	2572	1.811	154	87	176	0.495	-1.014742	0.611	0
chr7	73113534	73134017	**STX1A**	-	20483	1.084	738	418	845	0.495	-1.014035	0.455	0
chr18	8717368	8832775	**CCDC165**	+	115407	2.824	10955	6213	12536	0.496	-1.012682	0.58	0
chr11	65686727	65689048	**DRAP1**	+	2321	3.586	276	156	316	0.496	-1.012467	0.556	0
chr5	142657495	142815077	**NR3C1**	-	157582	3.766	20579	11671	23548	0.496	-1.01258	0.696	0
chrY	7142012	7249588	**PRKY**	+	107576	1.078	3887	2207	4447	0.496	-1.010304	0.468	0
chr12	56360555	56366568	**CDK2**	+	6013	2.941	596	339	682	0.497	-1.007985	0.496	0
chr11	58390145	58393205	**CNTF**	+	3060	4.509	460	261	526	0.497	-1.00862	0.509	0
chr6	34204576	34214008	**HMGA1**	+	9432	2.089	651	370	745	0.497	-1.009012	0.464	0
chr15	58983301	58985324	**HSP90AB4P**	-	2023	1.082	75	42	86	0.497	-1.007575	0.721	0
chr11	62414319	62420774	**INTS5**	-	6455	1.062	226	128	258	0.497	-1.007452	0.573	0
chr4	186131283	186285120	**SNX25**	+	153837	1.314	7068	4021	8084	0.497	-1.007529	0.576	0
chr5	86564069	86687743	**RASA1**	+	123674	3.336	14161	8063	16193	0.498	-1.006042	0.634	0
chr19	8575461	8579048	**ZNF414**	-	3587	1.342	157	89	179	0.498	-1.007223	0.595	0

chr15	75494220	75504510	**C15orf39**	+	10290	1.951	658	375	752	0.5	-1.001407	0.456	0
chr17	7482804	7485429	**CD68**	+	2625	2.303	201	114	230	0.5	-1.001129	0.595	0
chr6	26017259	26018040	**HIST1H1A**	-	781	1.111	30	17	34	0.5	-1.000703	0.831	0
chr8	116420723	116681228	**TRPS1**	-	260505	1.569	14032	8012	16039	0.5	-1.001199	0.635	0
chr19	56152391	56154836	**ZNF580**	+	2445	1.265	102	58	116	0.5	-0.998566	0.65	0
**Total**					**86755364**								
**Average size**					**110940**								
